# Association of dietary live microbe intake with various cognitive domains in US adults aged 60 years or older

**DOI:** 10.1038/s41598-024-51520-x

**Published:** 2024-03-08

**Authors:** Jia-jie Lv, Xin-yu Li, Jing-bing Wang, Xi-tao Yang, Min-yi Yin, Cheng-hao Yang

**Affiliations:** 1 Department of Vascular Surgery, Shanghai Putuo People’s Hospital, School of Medicine, Tongji University, No.1291 Jiangning Road, Huangpu District, Shanghai, 200011 China; 2 Department of Vascular Surgery, Shanghai Ninth People’s Hospital, School of Medicine, Shanghai Jiao Tong University, No.639 Zhizaoju Road, Huangpu District, Shanghai, 200011 China; 3 Department of Interventional Therapy, Multidisciplinary Team of Vascular Anomalies, Shanghai Ninth People’s Hospital, Shanghai Jiao Tong University, No.639 Zhizaoju Road, Huangpu District, Shanghai, 200011 China; 4 Department of Plastic and Reconstructive Surgery, Shanghai Ninth People’s Hospital, Shanghai Jiao Tong University School of Medicine, No.639 Zhizaoju Road, Huangpu District, Shanghai, 200011 China

**Keywords:** Microbiology, Diseases, Risk factors

## Abstract

The purpose of this study was to explore whether dietary live microbe intake is associated with various cognitive domains using data from the National Health and Nutrition Examination Survey (NHANES) from 2011 to 2014. And the specific relationship between low, medium and high dietary live microbe intake groups and cognitive ability of the elderly. Dietary live microbe intake was calculated from 24-h diet recall interviews. Cognitive function was assessed using the number symbol substitution test (DSST, which measures processing speed), the animal fluency test (AFT, which measures executive function), the Alzheimer's Registry sub-test (CERAD, which measures memory), and the Composite Z-score, which adds the Z-values of individual tests. Multiple linear regression models and restricted cubic bar graphs were used to investigate the relationship between live microbe intake and cognitive performance. A total of 2,450 participants aged 60 or older were included. Live microbe intake was positively correlated with cognitive ability on the whole. Specifically, when the intake of low, medium and high live microbe was > 2640 g, > 39 g and > 0 g respectively, the CERAD, DSST, AFT and compositive-Z score of the subjects increased with the increase of microbial intake (P < 0.05). In American adults age 60 or older, higher intakes of live microbes were associated with better cognitive performance, especially after a certain amount was reached.

## Introduction

Live microorganisms in the diet, or probiotics, are beneficial bacteria or yeasts that can be ingested to improve digestive health and enhance immunity ^1,2^. These microorganisms exist naturally in certain foods, such as yogurt, kefir, and sauerkraut, and are also available in supplement form ^2–4^. Probiotics function by colonizing the gut with beneficial bacteria, which helps to reestablish the microbiome's equilibrium and promote healthy digestion. Moreover, they aid in enhancing the immune system by improving gut health and reducing inflammation ^1,5^. Studies have revealed that probiotics may have potential benefits for various health conditions, such as irritable bowel syndrome, diarrhea, and eczema ^5–8^. However, more extensive research is required to fully comprehend the potential advantages of dietary live microbes, including the optimal strain and dosage for various health conditions ^9,10^.

Several reports have explored the physiological and pathological mechanisms of active microorganisms in the diet and their effects on brain function ^11–13^. Some research suggests that the gut-brain axis, a bidirectional communication system between the gut microbiota and the brain, plays a crucial role in linking peripheral gut function with emotional and cognitive brain centers through neuro-immune-endocrine mediators ^14,15^. Gut microbial dysbiosis can lead to the secretion of beta-amyloid (Aβ) and lipopolysaccharide (LPS), which disturb gastrointestinal permeability and the blood–brain barrier, modulating inflammatory signaling pathways and promoting neuroinflammation, nerve injury, and ultimately neuronal death in Alzheimer's disease (AD) ^14,16^. Consuming probiotic-rich foods to alter the gut microbial composition can serve as a preventive/therapeutic approach for AD. Currently, active microbes are considered one of the best preventive measures against cognitive decline in Alzheimer's disease ^17^. Numerous in vivo studies and more recently clinical trials have shown the effectiveness of selected bacterial strains in slowing the progression of Alzheimer's disease ^18,19^. Active microbes can modulate inflammatory processes, counteract oxidative stress, and modify gut microbial communities ^20,21^.

To the best of our knowledge, no studies have investigated the impact of dietary live microbe intake on cognitive function in the elderly, especially regarding the effects of low, medium, and high dietary live microbe intake on cognitive performance in the elderly population of the United States. To fill this gap in knowledge, we conducted a secondary analysis of data from the National Health and Nutrition Examination Survey (NHANES), controlling for multiple confounding factors. Our goal was to examine the relationship between dietary live microbe intake and cognitive function in the United States population, specifically in the elderly population, and to explore the potential link between dietary live microbe intake and cognitive performance across various classifications.

## Materials and methods

### Study population

**Figure 1:**
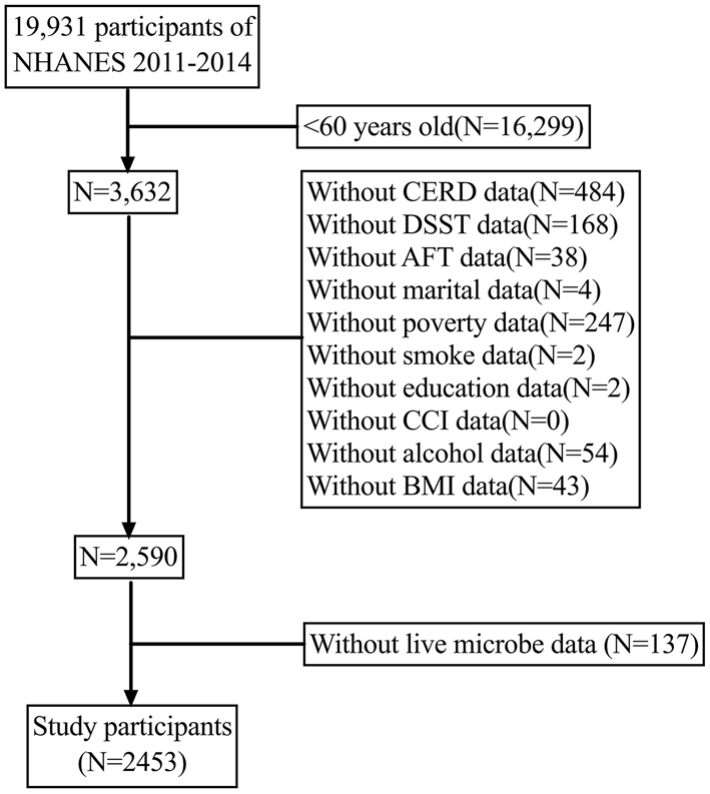
Flow chart of the study.

The National Health and Nutrition Examination Survey (NHANES) is a comprehensive and interdisciplinary survey program initiated by the Centers for Disease Control and Prevention (CDC) with the aim of evaluating the health and nutrition status of United States residents. The NHANES survey has been conducted annually since the 1960s and includes individuals of all ages from across the country. The overarching objective of the NHANES program is to collect, analyze, and publish data on the health, nutrition, and environmental exposures of U.S. residents. For our analysis, we combined two survey periods (2011–2012 and 2013–2014) to obtain more precise estimates with reduced sampling error. We restricted our study sample to individuals over 60 years of age, and we excluded those with missing data on important variables. The inclusion and exclusion criteria for our study are summarized in Fig. 1. The NHANES studies have been approved by the Ethics Review Committee of the National Center for Health Statistics and Research. Informed consent was obtained from every participant in the survey. The NHANES database is publicly accessible without the need for ethical or administrative approval. Data described in the manuscript, code book, and analytic code will be made publicly and freely available without restriction at https://www.cdc.gov/nchs/nhanes/index.htm.

### Exposure: dietary live microbe intake

In order to estimate the amounts of live microbes present in different food types, a group of four experts in the field (Maria L Marco, Mary E Sanders, Robert Hutkins, and Colin Hill) determined the estimated quantities of live microbes (per gram) for 9388 food codes contained in 48 subgroups within the NHANES database. Since the numbers of living microorganisms in each food type were expected to vary, the foods were categorized into three levels: low [Lo; < 10^4 colony-forming units per gram(CFU/g)], medium (Med; 10^4–10^7 CFU/g), or high (Hi; > 10^7 CFU/g) levels of live microbes. These levels were selected to reflect the approximate numbers of viable microbes expected to be present in pasteurized foods (< 10^4 CFU/g), fresh fruits and vegetables eaten unpeeled (10^4–10^7 CFU/g), and unpasteurized fermented foods and probiotic supplements (> 10^7 CFU/g) ^22^. In the initial phase, three researchers (MLM, MES, and RH) identified food subgroups presumed to encompass solely food codes with a microbial count of less than 10^4 CFU/g (refer to Supplementary Table 1). These evaluations were grounded on reported values in primary literature, authoritative reviews, or extrapolated values derived from the known impacts of food processing techniques, such as pasteurization, on microbial viability ^23–27^. Subsequently, 2 duos of experts scrutinized the remaining 6,317 food codes distributed across 25 food categories ^22,28,29^. Team1 (RH and CH) and Team 2 (MLM and MES) assessed 2,856 and 3,461 food codes, respectively, inclusive of the subgroups delineated in Supplementary Table 1^30^. For these evaluations, a panel of four experts (RH, CH, MLM and MES) drew upon a comprehensive review of the literature, authoritative critiques, and established knowledge regarding the impact of food processing techniques, such as pasteurization, on microbial viability. Discrepancies were resolved through intra- and inter-team reconciliation, supplemented by external consultation with Fred Breidt, a Microbiologist from the USDA Agricultural Research Service ^31^. The dietary intake of relevant live microbes was estimated using data from 24-h dietary recalls collected through face-to-face interviews that asked participants for detailed information about all foods and beverages consumed during the previous day (midnight to midnight). A complete description of the NHANES dietary interview methodology can be found in other literature ^32^. Initially, we utilize the NHANES 24-h dietary recall data to ascertain the consumption of various foods and the nutritional content relevant to our research subjects. Subsequently, we categorize these foods based on their corresponding active microbial content, as detailed in Supplementary Table 2. This categorization is aimed at classifying the dietary intake of active microbes by the subjects. Following this methodology, the subjects were ultimately segregated into three distinct groups. These include: LO (Low), encompassing subjects whose diet exclusively consisted of foods classified as having low viable microbial content; MED (Medium), comprising individuals who consumed foods categorized as medium in viable microbial content, but not high; and HI (High), referring to subjects who consumed foods deemed to have a high viable microbial content. Our analysis based on the above panel of experts estimate that active microbial content of food. Supplementary Table 2 lists the NHANES food codes and the assigned categories (the table is derived from the study of the expert panel formed by Maria et al.) ^22^.

### Outcome: cognitive function

In the NHANES 2011–2014 survey, a battery of cognitive function tests was administered to individuals aged 60 years and above. These tests included the word learning sub-test from the Consortium to Establish a Registry for Alzheimer's disease (CERAD), the Animal Fluency Test (AFT), and the Digit Symbol Substitution Test (DSST). The CERAD word learning subtest is a tool employed to evaluate immediate and delayed memory ^33^. It entails three successive learning trials and a delayed recall trial, in which the subject is expected to memorize a set of words across multiple learning and recall sessions, and to retrieve as many of these words as feasible during the final recall evaluation. During the learning trials, the participants were required to recite 10 dissimilar words aloud, and the sequence of the words varied across the trials. Following completion of the initial two cognitive function tests, the DSST and AFT, the delayed recall test necessitated the participant to remember these 10 words used during the prior learning trial. The DSST evaluates processing speed by requiring participants to accurately match numbers in 133 boxes to symbols that are paired with them on a paper form within a specific timeframe of 120 s^34^. This test measures reaction time and attention as the subject must complete as many digit symbol replacement tasks as possible within the given time, and the total number of correct matches is the result. The AFT is a language and cognitive assessment that measures absolute fluency in language, which is a component of executive function^35^. In this test, participants were instructed to name as many animals as they could as quickly as possible. Each correctly named animal is given one point, and the final score represents the total number of correctly named animals. Furthermore, a composite-z score was created by adding the z scores [(individual test score—mean score)/SD] of these three individual tests (DSST, AFT, CERAD). Higher scores on all the tests are indicative of better cognitive performance.

### Covariates

Based on previous research and clinical experience, the sociodemographic characteristics considered in this study included age, sex, race (Mexican American, white, black, and other), education level (less than high school, high school, and college or higher), marital status (living with a peer, single, and married), poverty income ratio (PIR), smoking status (former, never, and current), drinking status (never, former, light, moderate, and heavy), body mass index (BMI), metabolic equivalent (MET), and comorbidity index (CCI) ^32,36^. PIR, the ratio of total household income to the poverty line, was used as a measure of socioeconomic status, with low (PIR < 1.35), medium (1.35 ≤ PIR < 3.0), and high (PIR ≥ 3.0) categories. Participants who reported having never smoked or smoked fewer than 100 cigarettes in their lifetime were classified as never smokers, while those who reported having smoked 100 cigarettes in their lifetime but were not current smokers were classified as former smokers. Current smokers were defined as individuals who reported smoking 100 cigarettes per day on some days or during their lifetime ^16^. In terms of alcohol consumption, never drinkers were defined as individuals who reported consuming fewer than 12 drinks in their lifetime, while former drinkers were those who reported having drunk more than 12 times in their lifetime but had not consumed any alcohol in the previous year. Current drinkers were further classified into mild, moderate, or heavy drinkers. Heavy drinking was defined as consuming 3 or more drinks per day for women and 4 or more drinks per day for men, with binge drinking on 5 or more days per month. Moderate drinkers were defined as those who consumed up to 2 drinks per day for women and up to 3 drinks per day for men, with binge drinking on 2 or fewer days per month. Comorbidities considered in the study included conditions such as diabetes, congestive heart failure, coronary artery disease, chronic obstructive pulmonary disease (chronic bronchitis and/or emphysema), hypertension, and cancer. A comorbidity index (CCI) was calculated based on the number and severity of a range of conditions a patient has, each of which was assigned a score. Higher scores indicate more severe disease and a greater impact on patient survival and prognosis.

### Statistical analysis

The data analysis was conducted using appropriate NHANES sample weights. Mean values along with standard error (SE) were reported for continuous variables, while categorical variables were presented as numbers in weighted percentages. A weighted t-test was utilized to evaluate continuous variables, and a weighted χ2 test was performed for categorical variables. Subjects were categorized into three diet groups based on the content of viable microbes in their food intake: LO (all foods eaten were classified as low in viable microbes content); MED (foods eaten were classified as moderate but not high in viable microbes content); and, HI (foods eaten were considered as high in viable microbes content). Our objective was to investigate the potential relationship between the consumption of live microbe and cognitive function among the elderly population. First, we stratified the continuous variable of the composite-z score into three tertile concentrations. Categorical variables were analyzed using weighted chi-square, while continuous variables were assessed using weighted linear regression models across tertiles. Secondly, we developed multiple linear regression models to evaluate the impact of microbial intake with different densities on cognitive function. The association was examined using the original model (model 1), minimally adjusted model (model 2), and fully adjusted model (model 3) to determine the linear relationship between the consumption of live microbe and cognitive function . Finally, we conducted subgroup analyses utilizing hierarchical multiple linear regression to determine the subgroup associations between composite live microbe intake and cognitive function. To further investigate the relationship between different groups of live microbe intake and cognitive function, we constructed a fully adjusted model using a restricted spline model, and performed smoothed curve fitting to explore the trend of change among the groups. All statistical analyses were performed with R, version 4.2.3 (R Project for Statistical Computing).In all tests, P < 0.05 (2-sided) was considered to indicate statistical significance.

## Results

### Population characteristics

Table 1 displays the population characteristics of the study based on the composite z-scorequantile. The analysis included a total of 2,453 adults aged 60 years or older, with a mean age of 68.9 ± 6.7 years old, and 48.8% male. The results showed that participants with higher levels of cognition were more likely to have a high metabolic equivalent and a high intake of High Dietary Live Microbe (P < 0.05). Additionally, although there was no statistically significant difference, women, non-Hispanic whites, those with lower comorbidities, higher education levels, higher household income to poverty ratio, married, never smoked, and light alcohol use were more likely to have better cognitive function.

**Table 1:** Characteristics of the overall target population according to z-score tertiles (n = 2453).

Variable	Tertiles of composite-z score				*P*
	Total	Q1	Q2	Q3	
Age(years)	68.90 ± 0.21	72.56 ± 0.34	70.11 ± 0.40	66.48 ± 0.16	
Gender, n (%)					
Female	1255 (51.16)	367 (49.47)	403 (49.74)	485 (57.87)	
Male	1198 (48.84)	452 (50.53)	413 (50.26)	333 (42.13)	
Race/Ethnicity, n (%)					
Mexican American	210 (8.56)	92 (6.35)	67 (3.23)	51 (1.73)	
Non-Hispanic Black	570 (23.24)	246 (15.45)	194 (8.46)	130 (3.88)	
Non-Hispanic White	1232 (50.22)	302 (65.46)	397 (78.75)	533 (89.97)	
Other Hispanic	240 (9.78)	124 (7.87)	71 (3.23)	45 (1.31)	
Other race/ethnicity	201 (8.19)	55 (4.87)	87 (6.34)	59 (3.12)	
PIR	3.16 ± 0.08	2.23 ± 0.07	2.94 ± 0.09	3.72 ± 0.09	
BMI (kg/m^2^)	29.21 ± 0.24	28.84 ± 0.34	29.37 ± 0.27	29.26 ± 0.36	0.38
CCI	1.87 ± 0.05	2.26 ± 0.09	1.89 ± 0.11	1.68 ± 0.05	
MET	2898.26 ± 157.76	2412.04 ± 233.15	2777.74 ± 213.08	3124.59 ± 191.35	0.01
Marital status, n (%)					
Living with partner	65 (2.65)	18 (2.24)	22 (3.21)	25 (2.31)	0.43
Married	2185 (89.07)	719 (90.83)	727 (91.56)	739 (93.09)	
Single	203 (8.28)	82 (6.93)	67 (5.24)	54 (4.60)	
Education level, n (%)					
High school	570 (23.24)	202 (28.10)	221 (27.12)	147 (14.42)	
Less than high school	577 (23.52)	368 (35.07)	167 (17.10)	42 (3.71)	
More than high school	1306 (53.24)	249 (36.83)	428 (55.78)	629 (81.87)	
Low Dietary Live Microbe Group (g)	3003.34 ± 55.48	2671.25 ± 55.04	3034.58 ± 80.69	3132.06 ± 76.75	
Medium Dietary Live Microbe Group (g)	120.31 ± 5.03	87.33 ± 8.47	111.53 ± 7.40	140.74 ± 7.24	
High Dietary Live Microbe Group (g)	27.27 ± 2.83	17.41 ± 2.68	25.83 ± 3.23	32.60 ± 4.39	0.01
CERAD	5.02 ± 0.08	3.16 ± 0.05	4.73 ± 0.05	6.04 ± 0.08	
DSST	52.89 ± 0.56	33.57 ± 0.81	48.55 ± 0.54	64.36 ± 0.38	
AFT	18.40 ± 0.20	12.68 ± 0.18	16.47 ± 0.15	22.21 ± 0.25	
Smoking status, n (%)					
Former	956 (38.97)	325 (41.16)	321 (43.04)	310 (37.86)	0.13
Never	1195 (48.72)	378 (46.54)	393 (45.48)	424 (52.56)	
Now	302 (12.31)	116 (12.30)	102 (11.48)	84 (9.58)	
Alcohol consumption, n (%)					
Former	700 (28.54)	308 (34.98)	225 (26.41)	167 (15.93)	
Heavy	179 (7.3)	67 (6.87)	62 (6.38)	50 (5.36)	
Mild	975 (39.75)	230 (33.44)	342 (43.59)	403 (54.42)	
Moderate	240 (9.78)	58 (6.02)	68 (9.84)	114 (16.02)	
Never	359 (14.64)	156 (18.69)	119 (13.78)	84 (8.27)	

^a^PIR, Poverty to income ratio; BMI, Body mass index; CCI, Co-morbidity index; MET, metabolic equivalent.

^b^Mean and SE for continuous variables: *P* value was calculated by weighted t test. % for Categorical variables: *P* value was calculated by weighted χ2 test.

^c^Tertiles of composite-z score: Q1 represents the lowest level of cognition, Q1 represents the highest level of cognition.

### Association between different dietary live microbe groups score and cognitive function

Table 2 displays the associations between different groups of live microbe intake and cognitive function. The intake of different live microbe groups was divided into three quintiles, and three multiple linear regression models (as described previously) were constructed to determine the relationship between live microbe intake and cognitive performance (Table 2). In unadjusted model 1, the highest quintile of the Low Dietary Live Microbe Group was significantly associated with higher CERAD score (B:0.4; 95%CI: 0.21, 0.59; P < 0.001), DSST score (B: 6.28; 95%CI: 4.36, 8.21; P < 0.001), AFT score (B: 2.3; 95%CI: 1.48, 3.12; P < 0.001), and composite z-score(B: 1.02; 95%CI: 0.78, 1.29; P < 0.001) compared to the lowest quintile. In partially adjusted Model 2, the highest quintile of the Low Dietary Live Microbe Group was significantly associated with higher CERAD score (B: 0.27; 95%CI: 0.07,0.48; P < 0.05), DSST score (B: 4.04; 95%CI: 2.46, 5.62; P < 0.001), AFT score (B: 1.49; 95%CI: 0.7, 2.28; P < 0.01), and composite z-score(B: 0.67; 95%CI: 0.42, 0.92; P < 0.001). Similarly, in fully adjusted model 3, significant associations were observed between the highest quintile of the Low Dietary Live Microbe Group and higher scores in CERAD (B: 0.25; 95%CI: 0.03, 0.47; *P* < 0.05), DSST (B: 3.79; 95%CI: 1.97, 5.61; *P* < 0.001), AFT (B: 1.31; 95%CI: 0.38, 2.23; *P* < 0.01), and composite z-score (B: 0.55; 95%CI: 0.27, 0.83; *P* < 0.001). Furthermore, a linear trend was observed in all these associations. Similarly, in the Medium Dietary Live Microbe Group and the High Dietary Live Microbe Group, the highest intakes in each group were compared to the lowest intakes in each group, and significant differences were observed in CERAD score, AFT score, DSST score, and composite-z score (P < 0.05).

**Table 2:** Regression coefficients and 95% confidence intervals of different dietary live microbe group for cognitive function scores (n = 2453).

Exposure	Cognitive function			
	CERAD	DSST	AFT	Composite z-score
	Β(95% CI)	Β(95% CI)	Β(95% CI)	Β(95% CI)
Low dietary live microbe group				
Model 1				
Q1	0 (Reference)	0 (Reference)	0 (Reference)	0 (Reference)
Q2	0.29 (0.05, 0.53)*	5.38 (3.18, 7.58)***	1.69(0.68, 2.71)**	0.79 (0.40, 1.18)***
Q3	0.4 (0.21, 0.59)***	6.28 (4.36, 8.21)***	2.3(1.48, 3.12)***	1.02 (0.75, 1.29)***
P_trend_	< 0.001	< 0.0001	< 0.0001	< 0.0001
Model 2				
Q1	0 (Reference)	0 (Reference)	0 (Reference)	0 (Reference)
Q2	0.2 (− 0.02, 0.43)	3.88 (2.05, 5.71)***	1.18 (0.26, 2.11)*	0.56 (0.22, 0.90)**
Q3	0.27 (0.07, 0.48)*	4.04 (2.46, 5.62)***	1.49 (0.70, 2.28)**	0.67 (0.42, 0.92)***
P_trend_	0.01	< 0.0001	< 0.001	< 0.0001
Model 3				
Q1	0 (Reference)	0 (Reference)	0 (Reference)	0 (Reference)
Q2	0.14 (− 0.08, 0.36)	2.11 (0.44, 3.77)*	0.75 (− 0.15, 1.65)	0.34 (0.02, 0.66)*
Q3	0.2 (0.01, 0.39)*	1.89 (0.41, 3.37)*	0.95 (0.08, 1.82)*	0.39 (0.14, 0.64)**
P_trend_	0.04	0.02	0.04	0.004
Medium dietary live microbe group				
Model 1				
Q1	0 (Reference)	0 (Reference)	0 (Reference)	0 (Reference)
Q2	0.23 (− 0.01, 0.47)	4.14 (2.10, 6.17)***	1.26 (0.55, 1.97)**	0.61 (0.33, 0.88)***
Q3	0.35 (0.16, 0.55)**	6.89 (4.71, 9.07)***	1.93 (1.15, 2.71)***	0.96 (0.69, 1.24)***
P_trend_	< 0.001	< 0.0001	< 0.0001	< 0.0001
Model 2				
Q1	0 (Reference)	0 (Reference)	0 (Reference)	0 (Reference)
Q2	0.26 (0.03, 0.48)*	4.63 (2.60, 6.66)***	1.45 (0.76, 2.15)***	0.69 (0.43, 0.94)***
Q3	0.34 (0.16, 0.52)**	6.81 (4.85, 8.76)***	2.02 (1.32, 2.71)***	0.96 (0.74, 1.19)***
P_trend_	< 0.001	< 0.0001	< 0.0001	< 0.0001
Model 3				
Q1	0 (Reference)	0 (Reference)	0 (Reference)	0 (Reference)
Q2	0.17 (− 0.06, 0.39)	1.7 (− 0.38, 3.78)	0.66 (− 0.04, 1.36)	0.3 (0.05, 0.55)*
Q3	0.2 (0.03, 0.38)*	2.92 (0.92, 4.93)*	0.98 (0.24, 1.72)*	0.44 (0.19, 0.70)**
P_trend_	0.03	0.01	0.01	0.002
High dietary live microbe group				
Model 1				
Q1	0 (Reference)	0 (Reference)	0 (Reference)	0 (Reference)
Q2	0.3 (0.05, 0.54)*	4.61 (2.11, 7.11)**	1.52 (0.40, 2.64)*	0.72 (0.40, 1.04)***
Q3	0.28 (− 0.01, 0.58)	6.11 (3.55, 8.68)***	1.33 (0.14, 2.52)*	0.77 (0.40, 1.14)**
P_trend_	< 0.001	< 0.0001	0.01	< 0.0001
Model 2				
Q1	0 (Reference)	0 (Reference)	0 (Reference)	0 (Reference)
Q2	0.32 (0.06, 0.58)*	4.61 (2.32, 6.90)**	1.44 (0.35, 2.53)*	0.71 (0.39, 1.04)**
Q3	0.26 (− 0.04, 0.56)	5.15 (2.64, 7.66)**	1.16 (0.10, 2.23)*	0.64 (0.29, 0.98)**
P_trend_	< 0.001	< 0.0001	0.01	< 0.0001
Model 3				
Q1	0 (Reference)	0 (Reference)	0 (Reference)	0 (Reference)
Q2	0.32 (0.06, 0.58)*	1.64 (− 0.52, 3.80)	1.46 (0.37, 2.54)*	0.31 (− 0.01, 0.63)*
Q3	0.27(− 0.03, 0.57)	1.65 (− 0.65, 3.96)	1 (− 0.01, 2.01)*	0.18 (− 0.17, 0.53)
P_trend_	< 0.001	0.05	0.01	0.04

^a^B: unstandardized regression coefficient,***P < 0.001, **P < 0.01, *P < 0.05.

^b^The composite-z score was calculated by summing the z scores ((test score—mean score)/SD) of the three individual tests.

^c^Model 1: no covariates were adjusted. Model 2: adjusted for age; gender. Model 3: adjusted for age; gender; race; Education level; Marital status; PIR; BMI; CCI; smoking status; alcohol consumption.

^d^Tertiles of different dietary live microbe group: they were grouped according to the triquels of each category.

### Subgroup analyses and nonlinearity analysis

**Figure 2:**
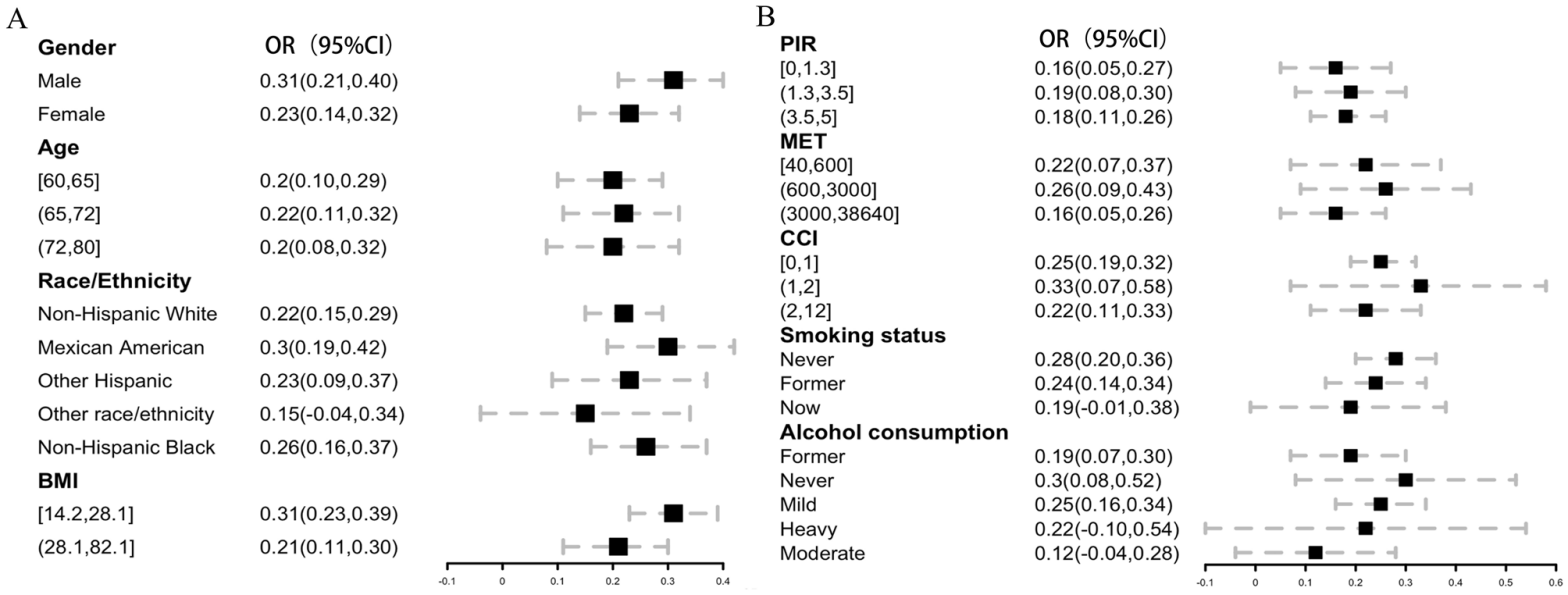
Forest plot of Composite z-score across Composite live microbe with subgroup, NHANES 2011–2014. OR: Odds ratio; CI: Confidence interval.

Figure 2 presents a subgroup analysis of the association between composite live microbe intake and cognitive ability, as measured by the compositive-z score. The analysis was stratified by various demographic and lifestyle factors, including sex, age, race, body mass index (BMI), household income ratio (PIR), metabolic equivalent (MET), comorbidity index (CCI), smoking status, and alcohol intake. The results indicate that women, Mexican Americans, never-smokers, individuals with low BMIs, and those with moderate MET had higher cognitive function.

**Figure 3:**
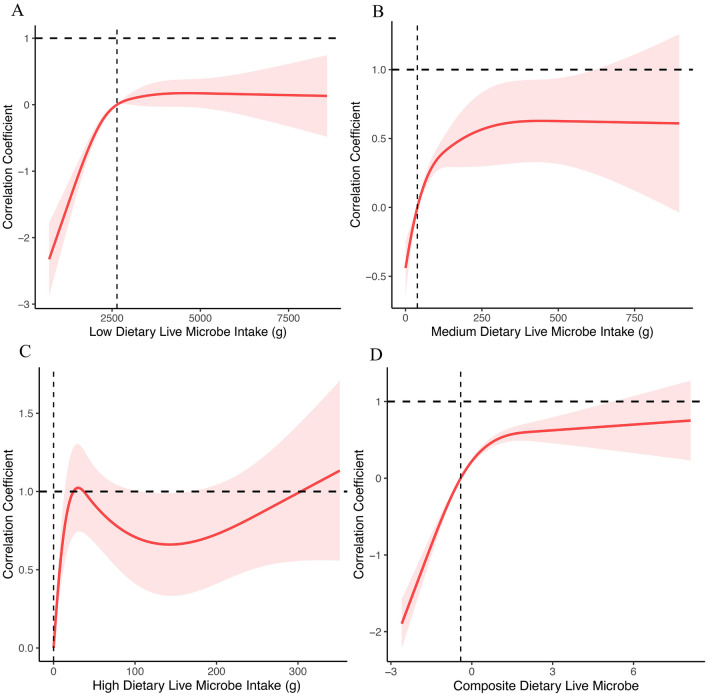
Correlation coefficient between Composite Z-score and different dietary live microbe intake during 2011–2014. Adjusted for age, gender, race, education, marital status, PIR, BMI, smoking status and alcohol consumption. The shaded area represents the 95% confidence interval.

To further investigate the relationship between each live microbe intake group and cognitive function, we used restricted cubic bar graphs to examine the low, medium, and high intake groups and their respective cognitive performances. We also employed a smooth curve to capture their trends (Fig. 3). After adjusting for potential confounders such as sex, age, race, education, marital status, poverty income ratio, BMI, smoking status, CCI, and alcohol intake, we found a positive correlation between live microbe intake and cognitive ability, as measured by the composite z-score(P < 0.05, Fig. 3D). More specifically, we analyzed the relationship between each live microbe intake group and cognitive function. For the low-live microbe intake group, we found a negative correlation with cognitive ability when the intake was less than 2640 g. However, this negative trend gradually decreased with the increase of intake. When the intake was greater than 2640 g, the low live microbe intake group was positively correlated with cognitive ability, and this positive trend remained stable with further increases in intake (Fig. 3A). In the moderate live microbe intake group, we observed a negative correlation between intake and cognitive ability when intake was less than 39 g. However, as the intake increased, the moderate live microbe intake group showed a positive correlation with cognitive ability, which was strengthened with further increases in intake and plateaued when the intake exceeded 250 g (Fig. 3B). Finally, for the high-live microbe intake group, we observed a positive correlation with cognitive ability that fluctuated within a certain range (Fig. 3C).

## Discussion

In this population-based study of older US adults, we aimed to investigate potential associations between active microbial intake and cognitive performance based on a 24-h dietary recall interview. Our findings reveal two important insights. Firstly, our analysis showed a significant association between dietary active microbial intake and cognitive performance in older age, even after adjusting for various potential confounders. In other words, overall, active microbial intake was positively correlated with cognitive function in older age. Secondly, we investigated different categories of active microorganisms in the diet. Our results indicated that when the intake of low active microorganisms was > 2640 g, the intake of medium active microorganisms was > 39 g, and the intake of high active microorganisms was > 0 g, cognitive performance tended to be enhanced with an increase in intake. To the best of our understanding, this study is the inaugural exploration of the nonlinear correlations between the intake of live microbes from dietary sources and distinct cognitive functions in a comprehensive, nationally representative cohort of older adults in the United States.

Recent studies have highlighted the gut microbiota as a pivotal intermediary in the relationship between diet and brain health, though investigations into the effects of live microbes on neurocognitive outcomes are still in their nascent stages ^37–39^. Evidence from animal research suggests that disruptions in gut microbial compositions can influence the production of neuroactive substances, instigate neuroinflammation, compromise the blood–brain barrier's integrity, and exacerbate the progression of dementia-related pathology ^40–42^. Our findings are in concordance with these preclinical observations, indicating that increasing microbial levels via the consumption of dietary live microbes could mitigate these detrimental effects. We hypothesize that reaching certain threshold levels of intake encourages the proliferation of beneficial bacteria, inhibits pathogenic organisms, upholds the function of the intestinal barrier, reduces inflammatory responses, and generates bioactive metabolites, all contributing to the preservation of neurological health ^43,44^. According to Maria et al. ^45^, these studies have produced evidence through human microbiota investigations, randomized controlled trials involving specific microorganisms (i.e., probiotics), and collaborative studies of fermented dairy product consumption that suggest regular consumption of safe live bacteria can provide cognitive benefits. One such study on elderly participants found that long-term consumption of probiotic-containing yogurt can improve spatial memory and attention ^46^. Another study corroborated the positive effects of dietary probiotics on cognitive performance in older adults, which is also impacted by dietary habits ^47^. Moreover, some research has suggested that dietary components like dietary fiber and polyphenols may also promote cognitive function in older adults and can function as prebiotics, stimulating the growth of intestinal microbiota ^48,49^.

In our cognitive test battery, we observed domain-specific correlations that varied according to the type of microbes consumed. For example, a greater consumption of low microbe varieties was associated with targeted improvements in memory functions, whereas medium microbe varieties predominantly boosted processing speed. Although further research is needed to fully elucidate the underlying mechanisms, it is plausible that certain microbial strains may exert differential effects on neurotransmitter systems, synaptic plasticity, and neurogenesis, each influencing specific cognitive abilities ^50,51^. Moreover, unique metabolites derived from these microbes might also have distinct neuromodulatory roles ^52^. Intriguingly, high microbe consumption appeared to enhance overall cognition without specific domain preference, which could be attributed to the wide variety of strains and functional metabolites present in higher microbial loads ^53,54^. These pioneering findings pave the way for future research aimed at customizing live microbe regimens to maximize neurological benefits across different functional domains.

Notably, our subgroup analyses provide vital insights on population segments garnering the greatest cognitive payoffs from live microbes. We found more pronounced associations between total microbe intake and cognitive performance in women compared to men. These sex-specific differences could reflect the immunomodulatory properties of estrogen interacting with gut-microbiota-brain signaling ^55^. Higher composite cognition scores were also seen for Mexican Americans versus other racial-ethnic groups as live microbe consumption increased. Traditional Mexican diets rich in microbe-containing items like corn, beans, vegetables, herbs, fruits and fermented foods could potentiate neurological effects ^56^. Moreover, never smokers demonstrated a steeper positive relationship between microbes and overall cognition compared to former/current smokers. Cigarette smoke dysregulates intestinal permeability and gut microbial profiles, which may hamper microbe-mediated cognitive gains ^57^. Those with moderate MET activity also had heightened cognition at higher intakes, possibly due to regular exercise optimizing gut microbial richness and metabolite production ^58^. These nuanced findings provide unique public health insights to better leverage dietary live microbes for brain health promotion across subpopulations.

The present study benefits from the use of a well-documented cohort with experts in nutrition and clinical medicine to examine the association between dietary active microbial intake and cognitive performance. Furthermore, the data was weighted to ensure the results are generalizable to the broader U.S. population. However, there are several limitations that need to be acknowledged. Firstly, the cross-sectional design of the study does not allow for the establishment of a causal relationship between live microbial intake and cognitive performance, despite some medical plausibility. Therefore, future longitudinal studies or clinical trials are necessary to confirm the associations reported here. Secondly, the estimates of live microbes in different food types were based on expert opinion, literature reviews, and knowledge of food processing rather than direct testing or culture. There was no external validation of the accuracy of these estimates, which introduces uncertainty. The categorization into low, medium, and high levels of live microbes was fairly broad and may not capture more nuanced differences. More precise quantification through culturing or molecular biology techniques could improve accuracy.

Additionally, while we adjusted for several potential confounding factors, the influence of other factors cannot be completely ruled out. Finally, we recognize the limitation of the timeliness of our analysis of associations between active microbes and cognition using data up to 2014, but the NHANES surveys provide a solid foundation for our study because they are comprehensive and nationally representative. The broad coverage and detailed demographic characteristics of this dataset make it a powerful tool for exploring the relationship between dietary active microbes and cognitive function. Therefore, larger-scale multicenter clinical studies are warranted to confirm the association between dietary active microbial intake and cognitive performance, as well as to elucidate its mechanisms and clinical applicability.

In summary, this nationally generalizable study found significantly higher performance across a spectrum of cognitive domains among older U.S. adults consuming greater quantities of dietary live microbes. Our analyses revealed threshold intake levels necessary for favorable neurocognitive effects within low, medium and high microbe groups. We also identified particularly receptive population subgroups based on sex, race-ethnicity, smoking status and physical activity patterns. These insights fill crucial gaps regarding nonlinear microbe-cognition dose-responses and effect modification by intrinsic biological and behavioral traits. Our findings motivate further experimental research on mechanisms and clinical trials establishing optimal microbe-based prevention protocols for age-related cognitive decline.

## Conclusion

Our study in the United States from 2011 to 2014 showed that there was a positive association between the intake of live microbe and cognitive performance in the elderly, but only when consumed in certain amounts. While the findings are reasonable, the study has some limitations, and therefore, further validation is needed through a large prospective cohort study.

### Supplementary Information

Supplementary Table 1.

Supplementary Table 2.

## Data Availability

Data described in the manuscript, code book, and analytic code will be made publicly and freely available without restriction at https://www.cdc.gov/nchs/nhanes/index.htm.
