# Supplementary material for: Association of dietary live microbe intake with various cognitive domains in US adults aged 60 years or older
Source: Sci Rep. 2024 Mar 8;14:5714. doi: 10.1038/s41598-024-51520-x (PMC10923796; doi:10.1038/s41598-024-51520-x)
Supplement: Supplementary file 1 — Supplementary Table 1. [file 41598_2024_51520_MOESM1_ESM.docx]

Supplementary Table 1. Food subgroups assigned to Teams 1 and 2 for live dietary microbe assignment. FC, food codes

| Subgroups | Team 1  (# FC: 2856) | Team 2  (# FC: 3461) | <10^4^ (unassigned)  (# FC: 3071) | FC/  Subgroup  (# FC: 9388) |
| --- | --- | --- | --- | --- |
| 100% Juice |  | x |  | 81 |
| Alcoholic Beverages |  |  | x | 95 |
| Baby Beverages |  |  | x | 20 |
| Baby Foods | x |  |  | 203 |
| Breads, Rolls, Tortillas |  |  | x | 242 |
| Candy |  |  | x | 133 |
| Cheese | x |  |  | 90 |
| Coffee and Tea |  |  | x | 161 |
| Condiments and Sauces |  | x |  | 204 |
| Cooked Cereals |  |  | x | 158 |
| Cooked Grains |  |  | x | 94 |
| Crackers |  |  | x | 88 |
| Cured Meats/Poultry | x |  |  | 176 |
| Dairy Drinks and Substitutes |  |  | x | 48 |
| Diet Beverages |  |  | x | 40 |
| Eggs | x |  |  | 219 |
| Fats and Oils | x |  |  | 184 |
| Flavored Milk |  |  | x | 72 |
| Flavored or Enhanced Water |  |  | x | 12 |
| Fruits |  | x |  | 242 |
| Infant Formulas | x |  |  | 213 |
| Meats | x |  |  | 215 |
| Milk | x |  |  | 37 |
| Mixed Dishes - Asian |  | x |  | 148 |
| Mixed Dishes - Bean/Vegetable-based |  | x |  | 145 |
| Mixed Dishes - Grain-based |  | x |  | 422 |
| Mixed Dishes - Meat, Poultry, Seafood |  | x |  | 551 |
| Mixed Dishes - Mexican | x |  |  | 163 |
| Mixed Dishes - Pizza |  |  | x | 119 |
| Mixed Dishes - Sandwiches (single code) | x |  |  | 482 |
| Mixed Dishes - Soups |  | x |  | 277 |
| Other |  | x |  | 86 |
| Other Desserts | x |  |  | 219 |
| Plain Water |  |  | x | 3 |
| Plant-based Protein Foods | x |  |  | 279 |
| Poultry |  |  | x | 396 |
| Protein and Nutritional Powders |  |  | x | 17 |
| Quick Breads and Bread Products |  |  | x | 142 |
| Ready-to-Eat Cereals |  |  | x | 238 |
| Savory Snacks |  |  | x | 163 |
| Seafood | x |  |  | 329 |
| Snack/Meal Bars |  |  | x | 93 |
| Sugars |  |  | x | 75 |
| Sweet Bakery Products |  |  | x | 472 |
| Sweetened Beverages |  | x |  | 208 |
| Vegetables, excluding Potatoes |  | x |  | 1097 |
| White Potatoes |  |  | x | 190 |
| Yogurt | x |  |  | 47 |
