# Supplementary material for: Association of dietary live microbe intake with various cognitive domains in US adults aged 60 years or older
Source: Sci Rep. 2024 Mar 8;14:5714. doi: 10.1038/s41598-024-51520-x (PMC10923796; doi:10.1038/s41598-024-51520-x)
Supplement: Supplementary file 2 — Supplementary Table 2. [file 41598_2024_51520_MOESM2_ESM.docx]

Supplementary Table 2. NHANES food codes and the assigned categories.

| **Foodcode** | **Description** | **Assigned** **category** | **Subgroup** |
| --- | --- | --- | --- |
| 11100000 | Milk, NFS | Lo | Milk |
| 11111000 | Milk, whole | Lo | Milk |
| 11111100 | Milk, low sodium, whole | Lo | Milk |
| 11111150 | Milk, calcium fortified, whole | Lo | Milk |
| 11111160 | Milk, calcium fortified, low fat (1%) | Lo | Milk |
| 11111170 | Milk, calcium fortified, fat free (skim | Lo | Milk |
| 11112000 | Milk, cow's, fluid, other than whole, | Lo | Milk |
| 11112110 | Milk, reduced fat (2%) | Lo | Milk |
| 11112210 | Milk, low fat (1%) | Lo | Milk |
| 11113000 | Milk, fat free (skim) | Lo | Milk |
| 11114300 | Milk, lactose free, low fat (1%) | Lo | Milk |
| 11114320 | Milk, lactose free, fat free (skim) | Lo | Milk |
| 11114330 | Milk, lactose free, reduced fat (2%) | Lo | Milk |
| 11114350 | Milk, lactose free, whole | Lo | Milk |
| 11116000 | Goat's milk, whole | Lo | Milk |
| 11120000 | Milk, dry, reconstituted, NS as to fa | Lo | Milk |
| 11121100 | Milk, dry, reconstituted, whole | Lo | Milk |
| 11121210 | Milk, dry, reconstituted, low fat (1% | Lo | Milk |
| 11121300 | Milk, dry, reconstituted, fat free (ski | Lo | Milk |
| 11210000 | Milk, evaporated, NS as to fat conte | Lo | Milk |
| 11210050 | Milk, evaporated, NS as to fat conte | Lo | Milk |
| 11210100 | Milk, evaporated, NS as to fat conte | Lo | Milk |
| 11211000 | Milk, evaporated, whole, NS as to d | Lo | Milk |
| 11211050 | Milk, evaporated, whole | Lo | Milk |
| 11211100 | Milk, evaporated, whole, undiluted | Lo | Milk |
| 11211200 | Milk, evaporated, whole, diluted | Lo | Milk |
| 11211400 | Milk, evaporated, reduced fat (2%) | Lo | Milk |
| 11212000 | Milk, evaporated, skim, NS as to dil | Lo | Milk |
| 11212050 | Milk, evaporated, fat free (skim) | Lo | Milk |
| 11212100 | Milk, evaporated, skim, undiluted | Lo | Milk |
| 11220000 | Milk, condensed, sweetened | Lo | Flavored Milk |
| 11220100 | Milk, condensed, sweetened, undilu | Lo | Flavored Milk |
| 11220200 | Milk, condensed, sweetened, dilute | Lo | Flavored Milk |
| 11310000 | Milk, imitation, fluid, soy based | Lo | Dairy Drinks and Substitutes |
| 11320000 | Soy milk | Lo | Dairy Drinks and Substitutes |
| 11320100 | Soy milk, light | Lo | Dairy Drinks and Substitutes |
| 11320200 | Soy milk, nonfat | Lo | Dairy Drinks and Substitutes |
| 11321000 | Soy milk, chocolate | Lo | Dairy Drinks and Substitutes |
| 11321100 | Soy milk, light, chocolate | Lo | Dairy Drinks and Substitutes |
| 11321200 | Soy milk, nonfat, chocolate | Lo | Dairy Drinks and Substitutes |
| 11330000 | Milk, soy, dry, reconstituted, not ba | Lo | Dairy Drinks and Substitutes |
| 11340000 | Imitation milk, non-soy, sweetened | Lo | Dairy Drinks and Substitutes |
| 11350000 | Almond milk, sweetened | Lo | Dairy Drinks and Substitutes |
| 11350010 | Almond milk, sweetened, chocolate | Lo | Dairy Drinks and Substitutes |
| 11350020 | Almond milk, unsweetened | Lo | Dairy Drinks and Substitutes |
| 11350030 | Almond milk, unsweetened, chocol | Lo | Dairy Drinks and Substitutes |
| 11360000 | Rice milk | Lo | Dairy Drinks and Substitutes |
| 11370000 | Coconut milk | Lo | Dairy Drinks and Substitutes |
| 11511000 | Chocolate milk, NFS | Lo | Flavored Milk |

| 11511100 | Chocolate milk, ready to drink, who | Lo | Flavored Milk |
| --- | --- | --- | --- |
| 11511200 | Chocolate milk, ready to drink, redu | Lo | Flavored Milk |
| 11511300 | Chocolate milk, ready to drink, fat fr | Lo | Flavored Milk |
| 11511400 | Chocolate milk, ready to drink, low | Lo | Flavored Milk |
| 11511550 | Chocolate milk, ready to drink, redu | Lo | Flavored Milk |
| 11511600 | Chocolate milk, ready to drink, low | Lo | Flavored Milk |
| 11511610 | Chocolate milk, ready to drink, fat fr | Lo | Flavored Milk |
| 11511700 | Chocolate milk, ready to drink, low | Lo | Flavored Milk |
| 11512000 | Cocoa, hot chocolate, not from dry | Lo | Flavored Milk |
| 11512010 | Hot chocolate / Cocoa, ready to dri | Lo | Flavored Milk |
| 11512020 | Hot chocolate / Cocoa, ready to dri | Lo | Flavored Milk |
| 11512030 | Hot chocolate / Cocoa, ready to dri | Lo | Dairy Drinks and Substitutes |
| 11512100 | Hot chocolate / Cocoa, ready to dri | Lo | Flavored Milk |
| 11512110 | Hot chocolate / Cocoa, ready to dri | Lo | Flavored Milk |
| 11512120 | Hot chocolate / Cocoa, ready to dri | Lo | Dairy Drinks and Substitutes |
| 11512500 | Hot chocolate, Puerto Rican style, | Lo | Flavored Milk |
| 11512510 | Hot chocolate, Puerto Rican style, | Lo | Flavored Milk |
| 11513000 | Chocolate milk, made from dry mix | Lo | Flavored Milk |
| 11513100 | Chocolate milk, made from dry mix | Lo | Flavored Milk |
| 11513150 | Chocolate milk, made from dry mix | Lo | Flavored Milk |
| 11513200 | Chocolate milk, made from dry mix | Lo | Flavored Milk |
| 11513300 | Chocolate milk, made from dry mix | Lo | Flavored Milk |
| 11513310 | Chocolate milk, made from dry mix | Lo | Dairy Drinks and Substitutes |
| 11513355 | Chocolate milk, made from reduced | Lo | Flavored Milk |
| 11513360 | Chocolate milk, made from reduced | Lo | Flavored Milk |
| 11513365 | Chocolate milk, made from reduced | Lo | Flavored Milk |
| 11513380 | Chocolate milk, made from dry mix | Lo | Flavored Milk |
| 11513381 | Chocolate milk, made from dry mix | Lo | Flavored Milk |
| 11513382 | Chocolate milk, made from dry mix | Lo | Flavored Milk |
| 11513383 | Chocolate milk, made from dry mix | Lo | Flavored Milk |
| 11513384 | Chocolate milk, made from dry mix | Lo | Flavored Milk |
| 11513391 | Chocolate milk, made from no suga | Lo | Flavored Milk |
| 11513392 | Chocolate milk, made from no suga | Lo | Flavored Milk |
| 11513393 | Chocolate milk, made from no suga | Lo | Flavored Milk |
| 11513394 | Chocolate milk, made from no suga | Lo | Flavored Milk |
| 11513395 | Chocolate milk, made from no suga | Lo | Dairy Drinks and Substitutes |
| 11513400 | Chocolate milk, made from syrup, N | Lo | Flavored Milk |
| 11513500 | Chocolate milk, made from syrup w | Lo | Flavored Milk |
| 11513550 | Chocolate milk, made from syrup w | Lo | Flavored Milk |
| 11513600 | Chocolate milk, made from syrup w | Lo | Flavored Milk |
| 11513700 | Chocolate milk, made from syrup w | Lo | Flavored Milk |
| 11513750 | Chocolate milk, made from syrup w | Lo | Dairy Drinks and Substitutes |
| 11513801 | Chocolate milk, made from light syr | Lo | Flavored Milk |
| 11513802 | Chocolate milk, made from light syr | Lo | Flavored Milk |
| 11513803 | Chocolate milk, made from light syr | Lo | Flavored Milk |
| 11513804 | Chocolate milk, made from light syr | Lo | Flavored Milk |
| 11513805 | Chocolate milk, made from light syr | Lo | Dairy Drinks and Substitutes |
| 11513851 | Chocolate milk, made from sugar fr | Lo | Flavored Milk |
| 11513853 | Chocolate milk, made from sugar fr | Lo | Flavored Milk |
| 11514100 | Hot chocolate / Cocoa, made with d | Lo | Flavored Milk |
| 11514110 | Hot chocolate / Cocoa, made with d | Lo | Flavored Milk |

| 11514120 | Hot chocolate / Cocoa, made with d | Lo | Flavored Milk |
| --- | --- | --- | --- |
| 11514130 | Hot chocolate / Cocoa, made with d | Lo | Flavored Milk |
| 11514140 | Hot chocolate / Cocoa, made with d | Lo | Flavored Milk |
| 11514150 | Hot chocolate / Cocoa, made with d | Lo | Dairy Drinks and Substitutes |
| 11514300 | Cocoa with nonfat dry milk and low | Lo | Flavored Milk |
| 11514310 | Hot chocolate / Cocoa, made with n | Lo | Flavored Milk |
| 11514320 | Hot chocolate / Cocoa, made with n | Lo | Flavored Milk |
| 11514330 | Hot chocolate / Cocoa, made with n | Lo | Flavored Milk |
| 11514340 | Hot chocolate / Cocoa, made with n | Lo | Flavored Milk |
| 11514350 | Hot chocolate / Cocoa, made with n | Lo | Flavored Milk |
| 11514360 | Hot chocolate / Cocoa, made with n | Lo | Dairy Drinks and Substitutes |
| 11514500 | Cocoa, whey, and low calorie swee | Lo | Dairy Drinks and Substitutes |
| 11516000 | Cocoa, whey, and low-calorie swee | Lo | Flavored Milk |
| 11518000 | Milk beverage with nonfat dry milk a | Lo | Dairy Drinks and Substitutes |
| 11519000 | Milk beverage, made with whole mi | Lo | Flavored Milk |
| 11519040 | Strawberry milk, NFS | Lo | Flavored Milk |
| 11519050 | Strawberry milk, whole | Lo | Flavored Milk |
| 11519105 | Strawberry milk, reduced fat | Lo | Flavored Milk |
| 11519200 | Strawberry milk, low fat | Lo | Flavored Milk |
| 11519205 | Strawberry milk, fat free | Lo | Flavored Milk |
| 11519210 | Strawberry milk, reduced sugar | Lo | Flavored Milk |
| 11519215 | Strawberry milk, non-dairy | Lo | Dairy Drinks and Substitutes |
| 11520000 | Milk, malted, unfortified, NS as to fl | Lo | Flavored Milk |
| 11521000 | Milk, malted, unfortified, chocolate, | Lo | Flavored Milk |
| 11522000 | Milk, malted, unfortified, natural flav | Lo | Flavored Milk |
| 11525000 | Milk, malted, natural flavor, made w | Lo | Flavored Milk |
| 11526000 | Milk, malted | Lo | Flavored Milk |
| 11527000 | Milk, malted, fortified, NS as to flav | Lo | Flavored Milk |
| 11531000 | Eggnog | Lo | Dairy Drinks and Substitutes |
| 11531500 | Eggnog, lowfat / light | Lo | Dairy Drinks and Substitutes |
| 11541000 | Milk shake, NS as to flavor or type | Lo | Dairy Drinks and Substitutes |
| 11541100 | Milk shake, homemade or fountain- | Lo | Dairy Drinks and Substitutes |
| 11541110 | Milk shake, home recipe, chocolate | Lo | Dairy Drinks and Substitutes |
| 11541120 | Milk shake, home recipe, flavors ot | Lo | Dairy Drinks and Substitutes |
| 11541130 | Milk shake, home recipe, chocolate | Lo | Dairy Drinks and Substitutes |
| 11541135 | Milk shake, home recipe, flavors ot | Lo | Dairy Drinks and Substitutes |
| 11541400 | Milk shake with malt | Lo | Dairy Drinks and Substitutes |
| 11541500 | Milk shake, made with skim milk, c | Lo | Dairy Drinks and Substitutes |
| 11541510 | Milk shake, made with skim milk, fl | Lo | Dairy Drinks and Substitutes |
| 11542100 | Milk shake, fast food, chocolate | Lo | Dairy Drinks and Substitutes |
| 11542200 | Milk shake, fast food, flavors other | Lo | Dairy Drinks and Substitutes |
| 11543000 | Milk shake, bottled, chocolate | Lo | Dairy Drinks and Substitutes |
| 11543010 | Milk shake, bottled, flavors other th | Lo | Dairy Drinks and Substitutes |
| 11551050 | Licuado or Batido | Lo | Sweetened Beverages |
| 11552200 | Orange Julius | Lo | Sweetened Beverages |
| 11553130 | Fruit smoothie juice drink, with dair | Lo | Sweetened Beverages |
| 11560000 | Chocolate milk drink | Lo | Dairy Drinks and Substitutes |
| 11560020 | Flavored milk drink, whey- and milk | Lo | Dairy Drinks and Substitutes |
| 11561000 | Cafe con leche | Lo | Coffee and Tea |
| 11561010 | Cafe con leche prepared with suga | Lo | Coffee and Tea |
| 11611000 | Instant breakfast, fluid, canned | Lo | Sweetened Beverages |

| 11612000 | Instant breakfast, powder, milk add | Lo | Sweetened Beverages |
| --- | --- | --- | --- |
| 11613000 | Instant breakfast, powder, sweeten | Lo | Sweetened Beverages |
| 11623000 | Meal supplement or replacement, c | Lo | Sweetened Beverages |
| 11631000 | High calorie beverage, canned or p | Lo | Sweetened Beverages |
| 11641000 | Meal supplement or replacement, m | Lo | Sweetened Beverages |
| 11641020 | Meal replacement or supplement, m | Lo | Sweetened Beverages |
| 11710000 | Infant formula, NFS | Lo | Infant Formulas |
| 11710050 | Infant formula, NS as to form (Simil | Lo | Infant Formulas |
| 11710051 | Infant formula, ready-to-feed (Simil | Lo | Infant Formulas |
| 11710053 | Infant formula, powder, made with | Lo | Infant Formulas |
| 11710054 | Infant formula, powder, made with t | Lo | Infant Formulas |
| 11710055 | Infant formula, powder, made with | Lo | Infant Formulas |
| 11710056 | Infant formula, powder, made with | Lo | Infant Formulas |
| 11710061 | Similac Alimentum Advance, with ir | Lo | Infant Formulas |
| 11710063 | Similac Alimentum Advance, with ir | Lo | Infant Formulas |
| 11710100 | Enfamil, low iron, infant formula, NS | Lo | Infant Formulas |
| 11710102 | Enfamil, low iron, infant formula, pr | Lo | Infant Formulas |
| 11710103 | Enfamil, low iron, infant formula, pr | Lo | Infant Formulas |
| 11710200 | Similac, low iron, infant formula, NS | Lo | Infant Formulas |
| 11710201 | Similac, low iron, infant formula, rea | Lo | Infant Formulas |
| 11710202 | Similac, low iron, infant formula, pre | Lo | Infant Formulas |
| 11710203 | Similac, low iron, infant formula, pre | Lo | Infant Formulas |
| 11710250 | Similac Natural Care Advance, low | Lo | Infant Formulas |
| 11710350 | Infant formula, NS as to form (Simil | Lo | Infant Formulas |
| 11710351 | Infant formula, ready-to-feed (Simil | Lo | Infant Formulas |
| 11710352 | Infant formula, liquid concentrate, m | Lo | Infant Formulas |
| 11710353 | Infant formula, powder, made with | Lo | Infant Formulas |
| 11710354 | Infant formula, liquid concentrate, m | Lo | Infant Formulas |
| 11710355 | Infant formula, liquid concentrate, m | Lo | Infant Formulas |
| 11710356 | Infant formula, liquid concentrate, m | Lo | Infant Formulas |
| 11710357 | Infant formula, powder, made with t | Lo | Infant Formulas |
| 11710358 | Infant formula, powder, made with | Lo | Infant Formulas |
| 11710359 | Infant formula, powder, made with | Lo | Infant Formulas |
| 11710367 | Infant formula, powder, made with t | Lo | Infant Formulas |
| 11710369 | Infant formula, powder, made with | Lo | Infant Formulas |
| 11710370 | Infant formula, NS as to form (Simil | Lo | Infant Formulas |
| 11710371 | Infant formula, ready-to-feed (Simil | Lo | Infant Formulas |
| 11710373 | Infant formula, powder, made with | Lo | Infant Formulas |
| 11710374 | Infant formula, liquid concentrate, m | Lo | Infant Formulas |
| 11710376 | Infant formula, liquid concentrate, m | Lo | Infant Formulas |
| 11710377 | Infant formula, powder, made with t | Lo | Infant Formulas |
| 11710378 | Infant formula, powder, made with | Lo | Infant Formulas |
| 11710379 | Infant formula, powder, made with | Lo | Infant Formulas |
| 11710380 | Infant formula, NS as to form (Simil | Lo | Infant Formulas |
| 11710381 | Infant formula, ready-to-feed (Simil | Lo | Infant Formulas |
| 11710383 | Infant formula, powder, made with | Lo | Infant Formulas |
| 11710387 | Similac Sensitive for Spit-Up, infant | Lo | Infant Formulas |
| 11710388 | Similac Sensitive for Spit-Up, infant | Lo | Infant Formulas |
| 11710389 | Similac Sensitive for Spit-Up, infant | Lo | Infant Formulas |
| 11710400 | Similac, with iron, infant formula, N | Lo | Infant Formulas |
| 11710401 | Similac, with iron, infant formula, re | Lo | Infant Formulas |

| 11710402 | Similac, with iron, infant formula, pr | Lo | Infant Formulas |
| --- | --- | --- | --- |
| 11710403 | Similac, with iron, infant formula, pr | Lo | Infant Formulas |
| 11710451 | Similac Special Care Advance 24, | Lo | Infant Formulas |
| 11710470 | Infant formula, NS as to form (Simil | Lo | Infant Formulas |
| 11710471 | Infant formula, ready-to-feed (Simil | Lo | Infant Formulas |
| 11710473 | Infant formula, powder, made with | Lo | Infant Formulas |
| 11710477 | Infant formula, powder, made with t | Lo | Infant Formulas |
| 11710478 | Infant formula, powder, made with | Lo | Infant Formulas |
| 11710479 | Infant formula, powder, made with | Lo | Infant Formulas |
| 11710480 | Infant formula, NS as to form (Simil | Lo | Infant Formulas |
| 11710481 | Infant formula, powder, made with | Lo | Infant Formulas |
| 11710482 | Similac Go and Grow, infant formul | Lo | Infant Formulas |
| 11710484 | Similac Go and Grow, infant formul | Lo | Infant Formulas |
| 11710600 | Enfamil, with iron, infant formula, N | Lo | Infant Formulas |
| 11710601 | Enfamil, with iron, infant formula, re | Lo | Infant Formulas |
| 11710602 | Enfamil, with iron, infant formula, pr | Lo | Infant Formulas |
| 11710603 | Enfamil, with iron, infant formula, pr | Lo | Infant Formulas |
| 11710627 | Infant formula, powder, made with t | Lo | Infant Formulas |
| 11710628 | Infant formula, powder, made with | Lo | Infant Formulas |
| 11710629 | Infant formula, powder, made with | Lo | Infant Formulas |
| 11710631 | Infant formula, ready-to-feed (Enfa | Lo | Infant Formulas |
| 11710633 | Infant formula, liquid concentrate, m | Lo | Infant Formulas |
| 11710634 | Infant formula, liquid concentrate, m | Lo | Infant Formulas |
| 11710635 | Infant formula, liquid concentrate, m | Lo | Infant Formulas |
| 11710637 | Infant formula, powder, made with t | Lo | Infant Formulas |
| 11710638 | Infant formula, powder, made with | Lo | Infant Formulas |
| 11710639 | Infant formula, powder, made with | Lo | Infant Formulas |
| 11710640 | Enfamil PREMIUM LIPIL, infant for | Lo | Infant Formulas |
| 11710643 | Enfamil PREMIUM LIPIL, infant for | Lo | Infant Formulas |
| 11710644 | Enfamil PREMIUM LIPIL, infant for | Lo | Infant Formulas |
| 11710646 | Enfamil PREMIUM LIPIL, infant for | Lo | Infant Formulas |
| 11710647 | Enfamil PREMIUM LIPIL, infant for | Lo | Infant Formulas |
| 11710648 | Enfamil PREMIUM LIPIL, infant for | Lo | Infant Formulas |
| 11710649 | Enfamil PREMIUM LIPIL, infant for | Lo | Infant Formulas |
| 11710650 | Enfamil LIPIL, infant formula, NS a | Lo | Infant Formulas |
| 11710651 | Enfamil LIPIL, infant formula, ready | Lo | Infant Formulas |
| 11710652 | Enfamil LIPIL, infant formula, prepa | Lo | Infant Formulas |
| 11710653 | Enfamil LIPIL, infant formula, prepa | Lo | Infant Formulas |
| 11710654 | Enfamil LIPIL, infant formula, prepa | Lo | Infant Formulas |
| 11710656 | Enfamil LIPIL, infant formula, prepa | Lo | Infant Formulas |
| 11710657 | Enfamil LIPIL, infant formula, prepa | Lo | Infant Formulas |
| 11710658 | Enfamil LIPIL, infant formula, prepa | Lo | Infant Formulas |
| 11710659 | Enfamil LIPIL, infant formula, prepa | Lo | Infant Formulas |
| 11710660 | Infant formula, NS as to form (Enfa | Lo | Infant Formulas |
| 11710661 | Infant formula, ready-to-feed (Enfa | Lo | Infant Formulas |
| 11710663 | Infant formula, powder, made with | Lo | Infant Formulas |
| 11710664 | Infant formula, powder, made with t | Lo | Infant Formulas |
| 11710667 | Infant formula, powder, made with | Lo | Infant Formulas |
| 11710668 | Infant formula, powder, made with | Lo | Infant Formulas |
| 11710669 | Infant formula, powder, made with | Lo | Infant Formulas |
| 11710671 | Infant formula, ready-to-feed (Enfa | Lo | Infant Formulas |

| 11710673 | Infant formula, powder, made with | Lo | Infant Formulas |
| --- | --- | --- | --- |
| 11710674 | Infant formula, powder, made with t | Lo | Infant Formulas |
| 11710675 | Infant formula, powder, made with | Lo | Infant Formulas |
| 11710677 | Infant formula, powder, made with t | Lo | Infant Formulas |
| 11710678 | Infant formula, powder, made with | Lo | Infant Formulas |
| 11710679 | Infant formula, powder, made with | Lo | Infant Formulas |
| 11710681 | Infant formula, ready-to-feed (Enfa | Lo | Infant Formulas |
| 11710683 | Infant formula, powder, made with | Lo | Infant Formulas |
| 11710687 | Infant formula, powder, made with t | Lo | Infant Formulas |
| 11710688 | Infant formula, powder, made with | Lo | Infant Formulas |
| 11710689 | Infant formula, powder, made with | Lo | Infant Formulas |
| 11710690 | Infant formula, NS as to form (Enfa | Lo | Infant Formulas |
| 11710697 | Infant formula, powder, made with t | Lo | Infant Formulas |
| 11710698 | Infant formula, powder, made with | Lo | Infant Formulas |
| 11710699 | Infant formula, powder, made with | Lo | Infant Formulas |
| 11710710 | Lactofree, with iron, infant formula, | Lo | Infant Formulas |
| 11710711 | Lactofree, with iron, infant formula, | Lo | Infant Formulas |
| 11710712 | Lactofree, with iron, infant formula, | Lo | Infant Formulas |
| 11710713 | Lactofree, with iron, infant formula, | Lo | Infant Formulas |
| 11710720 | Enfamil LactoFree Lipil, with iron, in | Lo | Infant Formulas |
| 11710721 | Enfamil LactoFree Lipil, with iron, in | Lo | Infant Formulas |
| 11710722 | Enfamil LactoFree Lipil, with iron, in | Lo | Infant Formulas |
| 11710723 | Enfamil LactoFree Lipil, with iron, in | Lo | Infant Formulas |
| 11710800 | Infant formula, NS as to form (Pedi | Lo | Infant Formulas |
| 11710801 | Infant formula, ready-to-feed (Pedia | Lo | Infant Formulas |
| 11710805 | Infant formula, with fiber, NS as to f | Lo | Infant Formulas |
| 11710806 | Infant formula, with fiber, ready-to-f | Lo | Infant Formulas |
| 11710900 | Good Start Supreme, with iron, infa | Lo | Infant Formulas |
| 11710901 | Good Start Supreme, with iron, infa | Lo | Infant Formulas |
| 11710902 | Good Start Supreme, with iron, infa | Lo | Infant Formulas |
| 11710903 | Good Start Supreme, with iron, infa | Lo | Infant Formulas |
| 11710910 | Infant formula, NS as to form (Gerb | Lo | Infant Formulas |
| 11710911 | Infant formula, ready-to-feed (Gerb | Lo | Infant Formulas |
| 11710912 | Infant formula, liquid concentrate, m | Lo | Infant Formulas |
| 11710913 | Infant formula, powder, made with | Lo | Infant Formulas |
| 11710916 | Infant formula, liquid concentrate, m | Lo | Infant Formulas |
| 11710917 | Infant formula, powder, made with t | Lo | Infant Formulas |
| 11710918 | Infant formula, powder, made with | Lo | Infant Formulas |
| 11710919 | Infant formula, powder, made with | Lo | Infant Formulas |
| 11710927 | Infant formula, powder, made with t | Lo | Infant Formulas |
| 11710928 | Infant formula, powder, made with | Lo | Infant Formulas |
| 11710929 | Infant formula, powder, made with | Lo | Infant Formulas |
| 11710930 | Infant formula, NS as to form (Gerb | Lo | Infant Formulas |
| 11710937 | Gerber Good Start 2 Gentle Plus, in | Lo | Infant Formulas |
| 11710938 | Gerber Good Start 2 Gentle Plus, in | Lo | Infant Formulas |
| 11710949 | Gerber Good Start 2 Protect Plus, i | Lo | Infant Formulas |
| 11710951 | Good Start 2 Essentials, with iron, i | Lo | Infant Formulas |
| 11710953 | Good Start 2 Essentials, with iron, i | Lo | Infant Formulas |
| 11710962 | Infant formula, powder, made with | Lo | Infant Formulas |
| 11710963 | Infant formula, ready-to-feed (Store | Lo | Infant Formulas |
| 11710964 | Infant formula, liquid concentrate, m | Lo | Infant Formulas |

| 11710966 | Infant formula, liquid concentrate, m | Lo | Infant Formulas |
| --- | --- | --- | --- |
| 11710967 | Infant formula, powder, made with t | Lo | Infant Formulas |
| 11710968 | Infant formula, powder, made with | Lo | Infant Formulas |
| 11710969 | Infant formula, powder, made with | Lo | Infant Formulas |
| 11720300 | Enfamil ProSobee, with iron, infant | Lo | Infant Formulas |
| 11720301 | Enfamil ProSobee, with iron, infant | Lo | Infant Formulas |
| 11720302 | Enfamil ProSobee, with iron, infant | Lo | Infant Formulas |
| 11720303 | Enfamil ProSobee, with iron, infant | Lo | Infant Formulas |
| 11720311 | Infant formula, ready-to-feed (Enfa | Lo | Infant Formulas |
| 11720312 | Infant formula, liquid concentrate, m | Lo | Infant Formulas |
| 11720313 | Infant formula, powder, made with | Lo | Infant Formulas |
| 11720316 | Infant formula, liquid concentrate, m | Lo | Infant Formulas |
| 11720317 | Infant formula, powder, made with t | Lo | Infant Formulas |
| 11720318 | Infant formula, powder, made with | Lo | Infant Formulas |
| 11720319 | Infant formula, powder, made with | Lo | Infant Formulas |
| 11720323 | Infant formula, powder, made with | Lo | Infant Formulas |
| 11720328 | Enfamil Enfagrow Soy Next Step LI | Lo | Infant Formulas |
| 11720329 | Enfamil Enfagrow Soy Next Step LI | Lo | Infant Formulas |
| 11720400 | Similac Isomil, with iron, infant form | Lo | Infant Formulas |
| 11720401 | Similac Isomil, with iron, infant form | Lo | Infant Formulas |
| 11720402 | Similac Isomil, with iron, infant form | Lo | Infant Formulas |
| 11720403 | Similac Isomil, with iron, infant form | Lo | Infant Formulas |
| 11720411 | Infant formula, ready-to-feed (Simil | Lo | Infant Formulas |
| 11720412 | Infant formula, liquid concentrate, m | Lo | Infant Formulas |
| 11720413 | Infant formula, powder, made with | Lo | Infant Formulas |
| 11720414 | Infant formula, liquid concentrate, m | Lo | Infant Formulas |
| 11720416 | Infant formula, liquid concentrate, m | Lo | Infant Formulas |
| 11720417 | Infant formula, powder, made with t | Lo | Infant Formulas |
| 11720418 | Infant formula, powder, made with | Lo | Infant Formulas |
| 11720419 | Infant formula, powder, made with | Lo | Infant Formulas |
| 11720430 | Infant formula, NS as to form (Simil | Lo | Infant Formulas |
| 11720431 | Infant formula, ready-to-feed (Simil | Lo | Infant Formulas |
| 11720600 | Good Start Essentials Soy, with iro | Lo | Infant Formulas |
| 11720601 | Good Start Essentials Soy, with iro | Lo | Infant Formulas |
| 11720602 | Good Start Essentials Soy, with iro | Lo | Infant Formulas |
| 11720603 | Good Start Essentials Soy, with iro | Lo | Infant Formulas |
| 11720612 | Infant formula, liquid concentrate, m | Lo | Infant Formulas |
| 11720613 | Infant formula, powder, made with | Lo | Infant Formulas |
| 11720615 | Infant formula, liquid concentrate, m | Lo | Infant Formulas |
| 11720617 | Infant formula, powder, made with t | Lo | Infant Formulas |
| 11720618 | Infant formula, powder, made with | Lo | Infant Formulas |
| 11720619 | Infant formula, powder, made with | Lo | Infant Formulas |
| 11720620 | Infant formula, NS as to form (Gerb | Lo | Infant Formulas |
| 11720628 | Gerber Good Start 2 Soy Plus, infa | Lo | Infant Formulas |
| 11720629 | Gerber Good Start 2 Soy Plus, infa | Lo | Infant Formulas |
| 11720703 | Next Step Prosobee, with iron, infa | Lo | Infant Formulas |
| 11720803 | Infant formula, powder, made with | Lo | Infant Formulas |
| 11720807 | Infant formula, powder, made with t | Lo | Infant Formulas |
| 11720808 | Infant formula, powder, made with | Lo | Infant Formulas |
| 11720809 | Infant formula, powder, made with | Lo | Infant Formulas |
| 11740301 | Nutramigen, with iron, infant formul | Lo | Infant Formulas |

| 11740302 | Nutramigen, with iron, infant formul | Lo | Infant Formulas |
| --- | --- | --- | --- |
| 11740303 | Nutramigen, with iron, infant formul | Lo | Infant Formulas |
| 11740310 | Infant formula, NS as to form (Enfa | Lo | Infant Formulas |
| 11740311 | Infant formula, ready-to-feed (Enfa | Lo | Infant Formulas |
| 11740312 | Infant formula, liquid concentrate, m | Lo | Infant Formulas |
| 11740313 | Infant formula, powder, made with | Lo | Infant Formulas |
| 11740317 | Enfamil Nutramigen LIPIL, infant fo | Lo | Infant Formulas |
| 11740318 | Enfamil Nutramigen LIPIL, infant fo | Lo | Infant Formulas |
| 11740319 | Enfamil Nutramigen LIPIL, infant fo | Lo | Infant Formulas |
| 11740323 | Infant formula, powder, made with | Lo | Infant Formulas |
| 11740329 | Enfamil Nutramigen AA LIPIL, infan | Lo | Infant Formulas |
| 11740400 | Infant formula, NS as to form (Enfa | Lo | Infant Formulas |
| 11740403 | Infant formula, powder, made with | Lo | Infant Formulas |
| 11740407 | Enfmail Pregestimil LIPIL, infant for | Lo | Infant Formulas |
| 11740520 | Enfamil Premature LIPIL 20, with ir | Lo | Infant Formulas |
| 11810000 | Milk, dry, not reconstituted, NS as t | Lo | Other |
| 11811000 | Milk, dry, not reconstituted, whole | Lo | Other |
| 11812000 | Milk, dry, not reconstituted, low fat ( | Lo | Other |
| 11813000 | Milk, dry, not reconstituted, fat free | Lo | Other |
| 11825000 | Whey, sweet, dry | Lo | Other |
| 11830100 | Hot chocolate / Cocoa, dry mix, not | Lo | Other |
| 11830110 | Cocoa powder with nonfat dry milk | Lo | Other |
| 11830115 | Hot chocolate / Cocoa, dry mix, no | Lo | Other |
| 11830120 | Cocoa, whey, and low calorie swee | Lo | Other |
| 11830140 | Chocolate, instant, dry mix, fortified | Lo | Other |
| 11830150 | Cocoa powder, not reconstituted | Lo | Other |
| 11830160 | Chocolate beverage powder, dry m | Lo | Other |
| 11830165 | Chocolate beverage powder, light, | Lo | Other |
| 11830170 | Cocoa (or chocolate) flavored beve | Lo | Other |
| 11830200 | Milk, malted, dry mix, unfortified, no | Lo | Other |
| 11830210 | Milk, malted, dry mix, fortified, not r | Lo | Other |
| 11830260 | Milk, malted, dry mix, not reconstitu | Lo | Other |
| 11830400 | Strawberry beverage powder, dry m | Lo | Other |
| 11830500 | Milk beverage, powder, with nonfat | Lo | Other |
| 11830550 | Milk beverage, powder, with nonfat | Lo | Other |
| 11830800 | Instant breakfast, powder, not reco | Lo | Other |
| 11830810 | Instant breakfast, powder, sweeten | Lo | Other |
| 11830850 | High calorie milk beverage, powder | Lo | Other |
| 11830900 | Protein supplement, milk-based, po | Lo | Other |
| 11830940 | Meal replacement, high protein, mil | Lo | Other |
| 11830970 | Meal replacement, protein type, mil | Lo | Other |
| 11830990 | Nutrient supplement, milk-based, p | Lo | Other |
| 11831500 | Nutrient supplement, milk-based, h | Lo | Other |
| 11832000 | Meal replacement, protein type, mil | Lo | Other |
| 11836000 | Protein supplement, milk-based, M | Lo | Other |
| 11836100 | Protein supplement, milk-based, M | Lo | Other |
| 12100100 | Cream, NS as to light, heavy, or ha | Lo | Fats and Oils |
| 12110100 | Cream, light | Lo | Fats and Oils |
| 12110300 | Cream, light, whipped, unsweetene | Lo | Fats and Oils |
| 12120100 | Cream, half and half | Lo | Fats and Oils |
| 12120105 | Cream, half and half, low fat | Lo | Fats and Oils |

| 12120106 | Cream, half and half, flavored | Lo | Fats and Oils |
| --- | --- | --- | --- |
| 12120110 | Cream, half and half, fat free | Lo | Fats and Oils |
| 12130100 | Cream, heavy | Lo | Fats and Oils |
| 12130200 | Cream, heavy, whipped, unsweeten | Lo | Fats and Oils |
| 12140000 | Cream, whipped | Lo | Fats and Oils |
| 12140100 | Cream, whipped, pressurized conta | Lo | Fats and Oils |
| 12140105 | Cream, whipped, pressurized conta | Lo | Fats and Oils |
| 12140110 | Whipped topping, dairy based, fat f | Lo | Fats and Oils |
| 12200100 | Coffee creamer, NFS | Lo | Fats and Oils |
| 12210100 | Cream substitute, frozen | Lo | Fats and Oils |
| 12210200 | Coffee creamer, liquid | Lo | Fats and Oils |
| 12210210 | Coffee creamer, liquid, flavored | Lo | Fats and Oils |
| 12210250 | Cream substitute, light, liquid | Lo | Fats and Oils |
| 12210255 | Cream substitute, light, flavored, liq | Lo | Fats and Oils |
| 12210260 | Coffee creamer, liquid, fat free | Lo | Fats and Oils |
| 12210270 | Coffee creamer, liquid, fat free, flav | Lo | Fats and Oils |
| 12210280 | Coffee creamer, liquid, fat free, sug | Lo | Fats and Oils |
| 12210305 | Cream substitute, sugar free, liquid | Lo | Fats and Oils |
| 12210310 | Coffee creamer, liquid, sugar free, f | Lo | Fats and Oils |
| 12210400 | Coffee creamer, powder | Lo | Fats and Oils |
| 12210410 | Cream substitute, light, powdered | Lo | Fats and Oils |
| 12210420 | Coffee creamer, powder, flavored | Lo | Fats and Oils |
| 12210430 | Coffee creamer, powder, fat free | Lo | Fats and Oils |
| 12210440 | Coffee creamer,powder, fat free, fla | Lo | Fats and Oils |
| 12210500 | Cream substitute, sugar free, powd | Lo | Fats and Oils |
| 12210505 | Coffee creamer,powder, sugar free | Lo | Fats and Oils |
| 12210520 | Coffee creamer, soy, liquid | Lo | Fats and Oils |
| 12220000 | Whipped topping, nondairy, NS as t | Lo | Fats and Oils |
| 12220100 | Whipped topping, nondairy, pressu | Lo | Fats and Oils |
| 12220200 | Whipped topping | Lo | Fats and Oils |
| 12220250 | Whipped topping, nondairy, frozen, | Lo | Fats and Oils |
| 12220270 | Whipped topping, fat free | Lo | Fats and Oils |
| 12220280 | Whipped topping, sugar free | Lo | Fats and Oils |
| 12220300 | Whipped cream substitute, nondair | Lo | Fats and Oils |
| 12220400 | Whipped cream substitute, nondair | Lo | Fats and Oils |
| 13110000 | Ice cream, NFS | Lo | Other Desserts |
| 13110100 | Ice cream, vanilla | Lo | Other Desserts |
| 13110102 | Ice cream, vanilla, with additional in | Lo | Other Desserts |
| 13110110 | Ice cream, chocolate | Lo | Other Desserts |
| 13110112 | Ice cream, chocolate, with additiona | Lo | Other Desserts |
| 13110120 | Ice cream, rich, flavors other than c | Lo | Other Desserts |
| 13110130 | Ice cream, rich, chocolate | Lo | Other Desserts |
| 13110140 | Ice cream, rich, NS as to flavor | Lo | Other Desserts |
| 13110200 | Ice cream, soft serve, vanilla | Lo | Other Desserts |
| 13110210 | Ice cream, soft serve, chocolate | Lo | Other Desserts |
| 13110220 | Ice cream, soft serve, NS as to flav | Lo | Other Desserts |
| 13110310 | Ice cream, no sugar added, NS as t | Lo | Other Desserts |
| 13110320 | Ice cream, no sugar added, flavors | Lo | Other Desserts |
| 13110330 | Ice cream, no sugar added, chocol | Lo | Other Desserts |
| 13110460 | Gelato, vanilla | Lo | Other Desserts |
| 13110470 | Gelato, chocolate | Lo | Other Desserts |

| 13120050 | Ice cream bar, vanilla | Lo | Other Desserts |
| --- | --- | --- | --- |
| 13120100 | Ice cream bar, vanilla, chocolate co | Lo | Other Desserts |
| 13120110 | Ice cream candy bar | Lo | Other Desserts |
| 13120120 | Ice cream bar or stick, rich chocola | Lo | Other Desserts |
| 13120121 | Ice cream bar or stick, rich ice crea | Lo | Other Desserts |
| 13120130 | Ice cream bar or stick, rich ice crea | Lo | Other Desserts |
| 13120140 | Ice cream bar, chocolate | Lo | Other Desserts |
| 13120300 | Ice cream bar, cake covered | Lo | Other Desserts |
| 13120310 | Ice cream bar, stick or nugget, with | Lo | Other Desserts |
| 13120400 | Ice cream bar or stick with fruit | Lo | Other Desserts |
| 13120500 | Ice cream sandwich, vanilla | Lo | Other Desserts |
| 13120510 | Ice cream sandwich, chocolate | Lo | Other Desserts |
| 13120550 | Ice cream cookie sandwich | Lo | Other Desserts |
| 13120700 | Ice cream cone with nuts, flavors ot | Lo | Other Desserts |
| 13120710 | Ice cream cone, chocolate covered | Lo | Other Desserts |
| 13120720 | Ice cream cone, chocolate covered | Lo | Other Desserts |
| 13120730 | Ice cream cone, scooped, vanilla | Lo | Other Desserts |
| 13120735 | Ice cream cone, scooped, vanilla, w | Lo | Other Desserts |
| 13120740 | Ice cream cone, NFS | Lo | Other Desserts |
| 13120750 | Ice cream cone with nuts, chocolat | Lo | Other Desserts |
| 13120760 | Ice cream cone, chocolate covered | Lo | Other Desserts |
| 13120770 | Ice cream cone, scooped, chocolat | Lo | Other Desserts |
| 13120775 | Ice cream cone, scooped, chocolat | Lo | Other Desserts |
| 13120780 | Ice cream cone, chocolate covered | Lo | Other Desserts |
| 13120782 | Ice cream cone, soft serve, vanilla | Lo | Other Desserts |
| 13120784 | Ice cream cone, soft serve, chocola | Lo | Other Desserts |
| 13120786 | Ice cream cone, soft serve, vanilla, | Lo | Other Desserts |
| 13120788 | Ice cream cone, soft serve, chocola | Lo | Other Desserts |
| 13120790 | Ice cream cone, vanilla, prepackag | Lo | Other Desserts |
| 13120792 | Ice cream cone, chocolate, prepack | Lo | Other Desserts |
| 13120800 | Ice cream soda, flavors other than | Lo | Dairy Drinks and Substitutes |
| 13120810 | Ice cream soda, chocolate | Lo | Dairy Drinks and Substitutes |
| 13121000 | Ice cream sundae, NFS | Lo | Other Desserts |
| 13121100 | Ice cream sundae, fruit topping | Lo | Other Desserts |
| 13121120 | Banana split | Lo | Other Desserts |
| 13121300 | Ice cream sundae, hot fudge toppin | Lo | Other Desserts |
| 13121400 | Ice cream sundae, caramel topping | Lo | Other Desserts |
| 13122100 | Ice cream pie, no crust | Lo | Other Desserts |
| 13122500 | Ice cream pie, with cookie crust, fu | Lo | Other Desserts |
| 13126000 | Ice cream, fried | Lo | Other Desserts |
| 13127000 | Dippin' Dots, flash frozen ice cream | Lo | Other Desserts |
| 13127010 | Dippin' Dots, flash frozen ice cream | Lo | Other Desserts |
| 13130100 | Light ice cream, NFS | Lo | Other Desserts |
| 13130300 | Light ice cream, vanilla | Lo | Other Desserts |
| 13130310 | Light ice cream, chocolate | Lo | Other Desserts |
| 13130320 | Light ice cream, no sugar added, N | Lo | Other Desserts |
| 13130330 | Light ice cream, no sugar added, fl | Lo | Other Desserts |
| 13130340 | Light ice cream, no sugar added, c | Lo | Other Desserts |
| 13130590 | Light ice cream, soft serve, NS as t | Lo | Other Desserts |
| 13130600 | Light ice cream, soft serve, flavors | Lo | Other Desserts |
| 13130610 | Light ice cream, soft serve, chocola | Lo | Other Desserts |

| 13130620 | Light ice cream, soft serve cone, fla | Lo | Other Desserts |
| --- | --- | --- | --- |
| 13130630 | Light ice cream, soft serve cone, ch | Lo | Other Desserts |
| 13130640 | Light ice cream, soft serve cone, N | Lo | Other Desserts |
| 13130700 | Soft serve, blended with candy or c | Lo | Other Desserts |
| 13135000 | Light ice cream sandwich, vanilla | Lo | Other Desserts |
| 13135010 | Light ice cream sandwich, chocolat | Lo | Other Desserts |
| 13136000 | Ice cream sandwich, made with ligh | Lo | Other Desserts |
| 13140000 | Light ice cream bar, vanilla | Lo | Other Desserts |
| 13140100 | Light ice cream bar, vanilla, chocola | Lo | Other Desserts |
| 13140110 | Light ice cream, bar or stick, choco | Lo | Other Desserts |
| 13140115 | Light ice cream bar, chocolate | Lo | Other Desserts |
| 13140450 | Light ice cream, cone, NFS | Lo | Other Desserts |
| 13140500 | Light ice cream, cone, flavors other | Lo | Other Desserts |
| 13140550 | Light ice cream, cone, chocolate | Lo | Other Desserts |
| 13140575 | Light ice cream, no sugar added, c | Lo | Other Desserts |
| 13140580 | Light ice cream, no sugar added, c | Lo | Other Desserts |
| 13140650 | Light ice cream, sundae, soft serve | Lo | Other Desserts |
| 13140660 | Light ice cream, sundae, soft serve | Lo | Other Desserts |
| 13140670 | Light ice cream, sundae, soft serve | Lo | Other Desserts |
| 13140680 | Light ice cream, sundae, soft serve | Lo | Other Desserts |
| 13140700 | Creamsicle | Lo | Other Desserts |
| 13140710 | Creamsicle, light | Lo | Other Desserts |
| 13140900 | Fudgesicle | Lo | Other Desserts |
| 13142000 | Milk dessert bar or stick, frozen, wit | Lo | Other Desserts |
| 13142100 | Light ice cream cone, vanilla, prepa | Lo | Other Desserts |
| 13142110 | Light ice cream cone, chocolate, pr | Lo | Other Desserts |
| 13150000 | Sherbet, all flavors | Lo | Other Desserts |
| 13160150 | Fat free ice cream, no sugar added | Lo | Other Desserts |
| 13160160 | Fat free ice cream, no sugar added | Lo | Other Desserts |
| 13160400 | Fat free ice cream, flavors other tha | Lo | Other Desserts |
| 13160410 | Fat free ice cream, chocolate | Lo | Other Desserts |
| 13161000 | Milk dessert bar, frozen, made from | Lo | Other Desserts |
| 13161500 | Milk dessert sandwich bar, frozen, | Lo | Other Desserts |
| 13161520 | Milk dessert sandwich bar, frozen, | Lo | Other Desserts |
| 13161600 | Fudgesicle, light | Lo | Other Desserts |
| 13161630 | Light ice cream, bar or stick, with lo | Lo | Other Desserts |
| 13170000 | Baked Alaska | Lo | Other Desserts |
| 13200110 | Pudding, chocolate, NFS | Lo | Other Desserts |
| 13210110 | Pudding, bread | Lo | Other Desserts |
| 13210180 | Pudding, Mexican bread | Lo | Other Desserts |
| 13210220 | Pudding, chocolate, NS as to from | Lo | Other Desserts |
| 13210250 | Pudding, chocolate, low calorie, co | Lo | Other Desserts |
| 13210270 | Custard, Puerto Rican style | Lo | Other Desserts |
| 13210280 | Pudding, flavors other than chocola | Lo | Other Desserts |
| 13210290 | Pudding, flavors other than chocola | Lo | Other Desserts |
| 13210300 | Custard | Lo | Other Desserts |
| 13210350 | Flan | Lo | Other Desserts |
| 13210370 | Creme brulee | Lo | Other Desserts |
| 13210410 | Pudding, rice | Lo | Other Desserts |
| 13210450 | Firni, Indian pudding | Lo | Other Desserts |
| 13210500 | Pudding, tapioca, made from home | Lo | Other Desserts |

| 13210520 | Pudding, tapioca, made from dry m | Lo | Other Desserts |
| --- | --- | --- | --- |
| 13210610 | Pudding, coconut | Lo | Other Desserts |
| 13210710 | Pudding, cornmeal, milk, and mola | Lo | Other Desserts |
| 13210750 | Pudding, pumpkin | Lo | Other Desserts |
| 13210810 | Pumpkin pudding, Puerto Rican sty | Lo | Other Desserts |
| 13210820 | Fresh corn custard, Puerto Rican st | Lo | Other Desserts |
| 13220110 | Pudding, flavors other than chocola | Lo | Other Desserts |
| 13220120 | Pudding, chocolate, made from dry | Lo | Other Desserts |
| 13220210 | Pudding, flavors other than chocola | Lo | Other Desserts |
| 13220220 | Pudding, chocolate, made from dry | Lo | Other Desserts |
| 13220230 | Pudding, ready-to-eat, chocolate, re | Lo | Other Desserts |
| 13220235 | Pudding, ready-to-eat, chocolate, fa | Lo | Other Desserts |
| 13220240 | Pudding, ready-to-eat, flavors other | Lo | Other Desserts |
| 13220245 | Pudding, ready-to-eat, flavors other | Lo | Other Desserts |
| 13230110 | Pudding, flavors other than chocola | Lo | Other Desserts |
| 13230120 | Pudding, flavors other than chocola | Lo | Other Desserts |
| 13230130 | Pudding, chocolate, ready-to-eat | Lo | Other Desserts |
| 13230140 | Pudding, chocolate, ready-to-eat, s | Lo | Other Desserts |
| 13230200 | Pudding, ready-to-eat, chocolate an | Lo | Other Desserts |
| 13230500 | Pudding, tapioca, ready-to-eat | Lo | Other Desserts |
| 13230510 | Pudding, ready-to-eat, tapioca, fat f | Lo | Other Desserts |
| 13241000 | Banana pudding | Lo | Other Desserts |
| 13250000 | Mousse | Lo | Other Desserts |
| 13250100 | Mousse, not chocolate | Lo | Other Desserts |
| 13250200 | Mousse, chocolate, lowfat, reduced | Lo | Other Desserts |
| 13252100 | Coconut custard, Puerto Rican styl | Lo | Other Desserts |
| 13252200 | Milk dessert or milk candy, Puerto | Lo | Other Desserts |
| 13252500 | Barfi or Burfi, Indian dessert | Lo | Other Desserts |
| 13252590 | Trifle | Lo | Other Desserts |
| 13252600 | Tiramisu | Lo | Sweet Bakery Products |
| 13310000 | Custard pudding, flavor other than | Lo | Baby Foods |
| 13311000 | Custard pudding, baby food, flavor | Lo | Baby Foods |
| 13312000 | Custard pudding, baby food, flavor | Lo | Baby Foods |
| 13411000 | White sauce or gravy | Lo | Condiments and Sauces |
| 13412000 | Milk gravy, quick gravy | Lo | Condiments and Sauces |
| 14120010 | Cheese, Mexican blend | Lo | Cheese |
| 14120020 | Cheese, Mexican blend, reduced fa | Lo | Cheese |
| 14131000 | Queso Anejo, aged Mexican chees | Lo | Cheese |
| 14131500 | Queso Asadero | Lo | Cheese |
| 14132000 | Queso Chihuahua | Lo | Cheese |
| 14133000 | Queso Fresco | Lo | Cheese |
| 14134000 | Queso cotija | Lo | Cheese |
| 14200100 | Cheese, cottage, NFS | Lo | Cheese |
| 14201010 | Cheese, cottage, creamed, large or | Lo | Cheese |
| 14201200 | Cottage cheese, farmer's | Lo | Cheese |
| 14201500 | Cheese, Ricotta | Lo | Cheese |
| 14202010 | Cheese, cottage, with fruit | Lo | Cheese |
| 14202020 | Cheese, cottage, with vegetables | Lo | Cheese |
| 14203010 | Cheese, cottage, dry curd | Lo | Cheese |
| 14203020 | Cheese, cottage, salted, dry curd | Lo | Cheese |
| 14203510 | Puerto Rican white cheese | Lo | Cheese |

| 14204010 | Cheese, cottage, low fat | Lo | Cheese |
| --- | --- | --- | --- |
| 14204020 | Cheese, cottage, lowfat, with fruit | Lo | Cheese |
| 14206010 | Cheese, cottage, lowfat, low sodium | Lo | Cheese |
| 14207010 | Cheese, cottage, lowfat, lactose red | Lo | Cheese |
| 14301010 | Cream cheese, regular, plain | Lo | Fats and Oils |
| 14301100 | Cream cheese, regular, flavored | Lo | Fats and Oils |
| 14303010 | Cream cheese, light | Lo | Fats and Oils |
| 14410100 | Cheese, American and Swiss blend | Lo | Cheese |
| 14410110 | Cheese, American | Lo | Cheese |
| 14410120 | Cheese, American, reduced fat | Lo | Cheese |
| 14410130 | Cheese, American, nonfat or fat fre | Lo | Cheese |
| 14410200 | Cheese, processed, American or C | Lo | Cheese |
| 14410210 | Cheese, American, reduced sodium | Lo | Cheese |
| 14410300 | Cheese, processed, American or C | Lo | Cheese |
| 14410330 | Cheese spread, American or Ched | Lo | Cheese |
| 14410350 | Cheese, processed, American or C | Lo | Cheese |
| 14410380 | Cream cheese spread, fat free | Lo | Fats and Oils |
| 14410400 | Cheese, processed, Swiss | Lo | Cheese |
| 14410420 | Cheese, processed, Swiss, lowfat | Lo | Cheese |
| 14410500 | Cheese, processed cheese food | Lo | Cheese |
| 14410600 | Cheese, processed, with vegetable | Lo | Cheese |
| 14420000 | Cheese spread, NFS | Lo | Cheese |
| 14420100 | Cheese spread, American or Ched | Lo | Cheese |
| 14420160 | Cheese spread, Swiss cheese bas | Lo | Cheese |
| 14420200 | Cheese spread, cream cheese, reg | Lo | Fats and Oils |
| 14420210 | Cheese spread, cream cheese, ligh | Lo | Fats and Oils |
| 14420300 | Cheese spread, pressurized can | Lo | Cheese |
| 14502000 | Imitation cheese | Lo | Cheese |
| 14502010 | Imitation cheese, American or ched | Lo | Cheese |
| 14502040 | Imitation cheese, American or ched | Lo | Cheese |
| 14504010 | Imitation mozzarella cheese | Lo | Cheese |
| 14610200 | Cheese, cottage cheese, with gelat | Lo | Cheese |
| 14610210 | Cheese, cottage cheese, with gelat | Lo | Cheese |
| 14610250 | Cheese, cottage cheese, with gelat | Lo | Cheese |
| 14610520 | Cheese ball | Lo | Cheese |
| 14620110 | Artichoke dip | Lo | Condiments and Sauces |
| 14620130 | Seafood dip | Lo | Condiments and Sauces |
| 14620150 | Cheese dip with chili pepper | Lo | Condiments and Sauces |
| 14620200 | Cheese dip | Lo | Condiments and Sauces |
| 14620300 | Topping from cheese pizza | Lo | Mixed Dishes - Pizza |
| 14620310 | Topping from vegetable pizza | Lo | Mixed Dishes - Pizza |
| 14620320 | Topping from meat pizza | Lo | Mixed Dishes - Pizza |
| 14620330 | Topping from meat and vegetable p | Lo | Mixed Dishes - Pizza |
| 14630100 | Cheese fondue | Lo | Condiments and Sauces |
| 14630200 | Cheese souffle | Lo | Eggs |
| 14630300 | Welsh rarebit | Lo | Condiments and Sauces |
| 14640002 | Cheese sandwich, American chees | Lo | Mixed Dishes - Sandwiches (single code) |
| 14640004 | Cheese sandwich, American chees | Lo | Mixed Dishes - Sandwiches (single code) |
| 14640006 | Cheese sandwich, American chees | Lo | Mixed Dishes - Sandwiches (single code) |
| 14640014 | Cheese sandwich, reduced fat Ame | Lo | Mixed Dishes - Sandwiches (single code) |
| 14640018 | Cheese sandwich, reduced fat Ame | Lo | Mixed Dishes - Sandwiches (single code) |

| 14640026 | Cheese sandwich, American chees | Lo | Mixed Dishes - Sandwiches (single code) |
| --- | --- | --- | --- |
| 14640028 | Cheese sandwich, American chees | Lo | Mixed Dishes - Sandwiches (single code) |
| 14640030 | Cheese sandwich, American chees | Lo | Mixed Dishes - Sandwiches (single code) |
| 14640042 | Cheese sandwich, reduced fat Ame | Lo | Mixed Dishes - Sandwiches (single code) |
| 14640050 | Cheese sandwich, American chees | Lo | Mixed Dishes - Sandwiches (single code) |
| 14640052 | Cheese sandwich, American chees | Lo | Mixed Dishes - Sandwiches (single code) |
| 14640054 | Cheese sandwich, American chees | Lo | Mixed Dishes - Sandwiches (single code) |
| 14640062 | Cheese sandwich, reduced fat Ame | Lo | Mixed Dishes - Sandwiches (single code) |
| 14640100 | Grilled cheese sandwich, NFS | Lo | Mixed Dishes - Sandwiches (single code) |
| 14640105 | Grilled cheese sandwich, American | Lo | Mixed Dishes - Sandwiches (single code) |
| 14640110 | Grilled cheese sandwich, American | Lo | Mixed Dishes - Sandwiches (single code) |
| 14640115 | Grilled cheese sandwich, American | Lo | Mixed Dishes - Sandwiches (single code) |
| 14640125 | Grilled cheese sandwich, Cheddar | Lo | Mixed Dishes - Sandwiches (single code) |
| 14640130 | Grilled cheese sandwich, Cheddar | Lo | Mixed Dishes - Sandwiches (single code) |
| 14640135 | Grilled cheese sandwich, Cheddar | Lo | Mixed Dishes - Sandwiches (single code) |
| 14640155 | Grilled cheese sandwich, reduced f | Lo | Mixed Dishes - Sandwiches (single code) |
| 14640160 | Grilled cheese sandwich, reduced f | Lo | Mixed Dishes - Sandwiches (single code) |
| 14640165 | Grilled cheese sandwich, reduced f | Lo | Mixed Dishes - Sandwiches (single code) |
| 14640185 | Grilled cheese sandwich, reduced f | Lo | Mixed Dishes - Sandwiches (single code) |
| 14640190 | Grilled cheese sandwich, reduced f | Lo | Mixed Dishes - Sandwiches (single code) |
| 14640195 | Grilled cheese sandwich, reduced f | Lo | Mixed Dishes - Sandwiches (single code) |
| 14650100 | Cheese sauce | Lo | Condiments and Sauces |
| 14650150 | Cheese sauce made with lowfat ch | Lo | Condiments and Sauces |
| 14650160 | Alfredo sauce | Lo | Condiments and Sauces |
| 14660200 | Mozzarella sticks, breaded, baked, | Lo | Cheese |
| 14710100 | Cheddar cheese soup, home recipe | Lo | Mixed Dishes - Soups |
| 14710200 | Beer cheese soup, made with milk | Lo | Mixed Dishes - Soups |
| 20000000 | Meat, NFS | Lo | Meats |
| 20000090 | Meat sticks, baby food, NS as to ty | Lo | Baby Foods |
| 20000200 | Ground meat, NFS | Lo | Meats |
| 21000100 | Beef, NS as to cut, cooked, NS as t | Lo | Meats |
| 21000110 | Beef, NS as to cut, cooked, lean an | Lo | Meats |
| 21000120 | Beef, NS as to cut, cooked, lean on | Lo | Meats |
| 21001000 | Steak, NS as to type of meat, cook | Lo | Meats |
| 21001010 | Steak, NS as to type of meat, cook | Lo | Meats |
| 21001020 | Steak, NS as to type of meat, cook | Lo | Meats |
| 21002000 | Beef, pickled | Lo | Meats |
| 21003000 | Beef, NS as to cut, fried, NS to fat e | Lo | Meats |
| 21101000 | Beef steak, NS as to cooking meth | Lo | Meats |
| 21101010 | Beef steak, NS as to cooking meth | Lo | Meats |
| 21101020 | Beef steak, NS as to cooking meth | Lo | Meats |
| 21101110 | Beef steak, broiled or baked, NS as | Lo | Meats |
| 21101120 | Beef steak, broiled or baked, lean a | Lo | Meats |
| 21101130 | Beef steak, broiled or baked, lean o | Lo | Meats |
| 21102110 | Beef steak, fried, NS as to fat eaten | Lo | Meats |
| 21102120 | Beef steak, fried, lean and fat eaten | Lo | Meats |
| 21102130 | Beef steak, fried, lean only eaten | Lo | Meats |
| 21103110 | Beef steak, breaded or floured, bak | Lo | Meats |
| 21103120 | Beef steak, breaded or floured, bak | Lo | Meats |
| 21103130 | Beef steak, breaded or floured, bak | Lo | Meats |
| 21104110 | Beef steak, battered, fried, NS as to | Lo | Meats |

| 21104120 | Beef steak, battered, fried, lean and | Lo | Meats |
| --- | --- | --- | --- |
| 21104130 | Beef steak, battered, fried, lean onl | Lo | Meats |
| 21105110 | Beef steak, braised, NS as to fat ea | Lo | Meats |
| 21105120 | Beef steak, braised, lean and fat ea | Lo | Meats |
| 21105130 | Beef steak, braised, lean only eaten | Lo | Meats |
| 21301000 | Beef, oxtails, cooked | Lo | Meats |
| 21302000 | Beef, neck bones, cooked | Lo | Meats |
| 21304000 | Beef, shortribs, cooked, NS as to fa | Lo | Meats |
| 21304110 | Beef, shortribs, cooked, lean and fa | Lo | Meats |
| 21304120 | Beef, shortribs, cooked, lean only e | Lo | Meats |
| 21304200 | Beef, shortribs, barbecued, with sa | Lo | Meats |
| 21304210 | Beef, shortribs, barbecued, with sa | Lo | Meats |
| 21304220 | Beef, shortribs, barbecued, with sa | Lo | Meats |
| 21305000 | Beef, cow head, cooked | Lo | Meats |
| 21401000 | Beef, roast, roasted, NS as to fat e | Lo | Meats |
| 21401110 | Beef, roast, roasted, lean and fat e | Lo | Meats |
| 21401120 | Beef, roast, roasted, lean only eate | Lo | Meats |
| 21401400 | Beef, roast, canned | Lo | Meats |
| 21407000 | Beef, pot roast, braised or boiled, N | Lo | Meats |
| 21407110 | Beef, pot roast, braised or boiled, le | Lo | Meats |
| 21407120 | Beef, pot roast, braised or boiled, le | Lo | Meats |
| 21410000 | Beef, stew meat, cooked, NS as to | Lo | Meats |
| 21410110 | Beef, stew meat, cooked, lean and | Lo | Meats |
| 21410120 | Beef, stew meat, cooked, lean only | Lo | Meats |
| 21416000 | Corned beef, cooked, NS as to fat | Lo | Cured Meats/Poultry |
| 21416110 | Corned beef, cooked, lean and fat | Lo | Cured Meats/Poultry |
| 21416120 | Corned beef, cooked, lean only eat | Lo | Cured Meats/Poultry |
| 21416150 | Corned beef, canned, ready-to-eat | Lo | Cured Meats/Poultry |
| 21417100 | Beef brisket, cooked, NS as to fat e | Lo | Meats |
| 21417110 | Beef brisket, cooked, lean and fat e | Lo | Meats |
| 21417120 | Beef brisket, cooked, lean only eate | Lo | Meats |
| 21420100 | Beef, sandwich steak, flaked, forme | Lo | Meats |
| 21500100 | Ground beef, cooked | Lo | Meats |
| 21500110 | Ground beef, meatballs, meat only, | Lo | Meats |
| 21500200 | Ground beef or patty, breaded, coo | Lo | Meats |
| 21500300 | Ground beef patty, cooked (for fast | Lo | Meats |
| 21500310 | Ground beef patty, cooked | Lo | Meats |
| 21501000 | Ground beef, less than 80% lean, c | Lo | Meats |
| 21501200 | Ground beef, 80% - 84% lean, cook | Lo | Meats |
| 21501300 | Ground beef, 85% - 89% lean, cook | Lo | Meats |
| 21501350 | Ground beef, 90% - 94% lean, cook | Lo | Meats |
| 21501360 | Ground beef, 95% or more lean, co | Lo | Meats |
| 21540100 | Ground beef with textured vegetabl | Lo | Meats |
| 21601000 | Beef, bacon, cooked | Lo | Cured Meats/Poultry |
| 21601010 | Beef, bacon, reduced sodium, cook | Lo | Cured Meats/Poultry |
| 21601250 | Beef, bacon, cooked, lean only eate | Lo | Cured Meats/Poultry |
| 21601500 | Beef, bacon, formed, lean meat ad | Lo | Cured Meats/Poultry |
| 21602000 | Beef, dried, chipped, uncooked | Lo | Cured Meats/Poultry |
| 21602010 | Beef, dried, chipped, cooked in fat | Lo | Cured Meats/Poultry |
| 21602100 | Beef jerky | Lo | Cured Meats/Poultry |
| 21603000 | Beef, pastrami (beef, smoked, spic | Lo | Cured Meats/Poultry |

| 21701010 | Beef, baby food, strained | Lo | Baby Foods |
| --- | --- | --- | --- |
| 21701020 | Beef, baby food, junior | Lo | Baby Foods |
| 22000100 | Pork, NS as to cut, cooked, NS as t | Lo | Meats |
| 22000110 | Pork, NS as to cut, cooked, lean an | Lo | Meats |
| 22000120 | Pork, NS as to cut, cooked, lean on | Lo | Meats |
| 22000200 | Pork, NS as to cut, fried, NS as to f | Lo | Meats |
| 22000210 | Pork, NS as to cut, fried, lean and f | Lo | Meats |
| 22000220 | Pork, NS as to cut, fried, lean only | Lo | Meats |
| 22000300 | Pork, NS as to cut, breaded or flour | Lo | Meats |
| 22000310 | Pork, NS as to cut, breaded or flour | Lo | Meats |
| 22000320 | Pork, NS as to cut, breaded or flour | Lo | Meats |
| 22001000 | Pork, pickled, NS as to cut | Lo | Meats |
| 22002000 | Pork, ground or patty, cooked | Lo | Meats |
| 22002100 | Pork, ground or patty, breaded, coo | Lo | Meats |
| 22002800 | Pork jerky | Lo | Cured Meats/Poultry |
| 22101000 | Pork chop, NS as to cooking metho | Lo | Meats |
| 22101010 | Pork chop, NS as to cooking metho | Lo | Meats |
| 22101020 | Pork chop, NS as to cooking metho | Lo | Meats |
| 22101100 | Pork chop, broiled or baked, NS as | Lo | Meats |
| 22101110 | Pork chop, broiled or baked, lean a | Lo | Meats |
| 22101120 | Pork chop, broiled or baked, lean o | Lo | Meats |
| 22101130 | Pork chop, breaded or floured, broil | Lo | Meats |
| 22101140 | Pork chop, breaded or floured, broil | Lo | Meats |
| 22101150 | Pork chop, breaded or floured, broil | Lo | Meats |
| 22101200 | Pork chop, fried, NS as to fat eaten | Lo | Meats |
| 22101210 | Pork chop, fried, lean and fat eaten | Lo | Meats |
| 22101220 | Pork chop, fried, lean only eaten | Lo | Meats |
| 22101300 | Pork chop, breaded or floured, fried | Lo | Meats |
| 22101310 | Pork chop, breaded or floured, fried | Lo | Meats |
| 22101320 | Pork chop, breaded or floured, fried | Lo | Meats |
| 22101400 | Pork chop, battered, fried, NS as to | Lo | Meats |
| 22101410 | Pork chop, battered, fried, lean and | Lo | Meats |
| 22101420 | Pork chop, battered, fried, lean only | Lo | Meats |
| 22101500 | Pork chop, stewed, NS as to fat eat | Lo | Meats |
| 22101510 | Pork chop, stewed, lean and fat eat | Lo | Meats |
| 22101520 | Pork chop, stewed, lean only eaten | Lo | Meats |
| 22107000 | Pork chop, smoked or cured, cooke | Lo | Cured Meats/Poultry |
| 22107010 | Pork chop, smoked or cured, cooke | Lo | Cured Meats/Poultry |
| 22107020 | Pork chop, smoked or cured, cooke | Lo | Cured Meats/Poultry |
| 22201000 | Pork steak or cutlet, NS as to cooki | Lo | Meats |
| 22201010 | Pork steak or cutlet, NS as to cooki | Lo | Meats |
| 22201020 | Pork steak or cutlet, NS as to cooki | Lo | Meats |
| 22201050 | Pork steak or cutlet, battered, fried, | Lo | Meats |
| 22201060 | Pork steak or cutlet, battered, fried, | Lo | Meats |
| 22201070 | Pork steak or cutlet, battered, fried, | Lo | Meats |
| 22201100 | Pork steak or cutlet, broiled or bake | Lo | Meats |
| 22201110 | Pork steak or cutlet, broiled or bake | Lo | Meats |
| 22201120 | Pork steak or cutlet, broiled or bake | Lo | Meats |
| 22201200 | Pork steak or cutlet, fried, NS as to | Lo | Meats |
| 22201210 | Pork steak or cutlet, fried, lean and | Lo | Meats |
| 22201220 | Pork steak or cutlet, fried, lean only | Lo | Meats |

| 22201300 | Pork steak or cutlet, breaded or flo | Lo | Meats |
| --- | --- | --- | --- |
| 22201310 | Pork steak or cutlet, breaded or flo | Lo | Meats |
| 22201320 | Pork steak or cutlet, breaded or flo | Lo | Meats |
| 22201400 | Pork steak or cutlet, breaded or flo | Lo | Meats |
| 22201410 | Pork steak or cutlet, breaded or flo | Lo | Meats |
| 22201420 | Pork steak or cutlet, breaded or flo | Lo | Meats |
| 22210300 | Pork, tenderloin, cooked, NS as to | Lo | Meats |
| 22210310 | Pork, tenderloin, breaded, fried | Lo | Meats |
| 22210350 | Pork, tenderloin, braised | Lo | Meats |
| 22210400 | Pork, tenderloin, baked | Lo | Meats |
| 22210450 | Pork, tenderloin, battered, fried | Lo | Meats |
| 22300120 | Ham, fried, NS as to fat eaten | Lo | Cured Meats/Poultry |
| 22300130 | Ham, fried, lean and fat eaten | Lo | Cured Meats/Poultry |
| 22300140 | Ham, fried, lean only eaten | Lo | Cured Meats/Poultry |
| 22300150 | Ham, breaded or floured, fried, NS | Lo | Cured Meats/Poultry |
| 22300160 | Ham, breaded or floured, fried, lean | Lo | Cured Meats/Poultry |
| 22300170 | Ham, breaded or floured, fried, lean | Lo | Cured Meats/Poultry |
| 22301000 | Ham, fresh, cooked, NS as to fat e | Lo | Meats |
| 22301110 | Ham, fresh, cooked, lean and fat ea | Lo | Meats |
| 22301120 | Ham, fresh, cooked, lean only eate | Lo | Meats |
| 22311000 | Ham, smoked or cured, cooked, NS | Lo | Cured Meats/Poultry |
| 22311010 | Ham, smoked or cured, cooked, lea | Lo | Cured Meats/Poultry |
| 22311020 | Ham, smoked or cured, cooked, lea | Lo | Cured Meats/Poultry |
| 22311220 | Ham, smoked or cured, low sodium | Lo | Cured Meats/Poultry |
| 22311450 | Ham, prosciutto | Lo | Cured Meats/Poultry |
| 22311500 | Ham, smoked or cured, canned, N | Lo | Cured Meats/Poultry |
| 22311510 | Ham, smoked or cured, canned, le | Lo | Cured Meats/Poultry |
| 22311520 | Ham, smoked or cured, canned, le | Lo | Cured Meats/Poultry |
| 22321110 | Ham, smoked or cured, ground pat | Lo | Cured Meats/Poultry |
| 22400100 | Pork roast, NS as to cut, cooked, N | Lo | Meats |
| 22400110 | Pork roast, NS as to cut, cooked, le | Lo | Meats |
| 22400120 | Pork roast, NS as to cut, cooked, le | Lo | Meats |
| 22401000 | Pork roast, loin, cooked, NS as to f | Lo | Meats |
| 22401010 | Pork roast, loin, cooked, lean and f | Lo | Meats |
| 22401020 | Pork roast, loin, cooked, lean only e | Lo | Meats |
| 22411000 | Pork roast, shoulder, cooked, NS a | Lo | Meats |
| 22411010 | Pork roast, shoulder, cooked, lean | Lo | Meats |
| 22411020 | Pork roast, shoulder, cooked, lean | Lo | Meats |
| 22421000 | Pork roast, smoked or cured, cooke | Lo | Cured Meats/Poultry |
| 22421010 | Pork roast, smoked or cured, cooke | Lo | Cured Meats/Poultry |
| 22421020 | Pork roast, smoked or cured, cooke | Lo | Cured Meats/Poultry |
| 22431000 | Pork roll, cured, fried | Lo | Cured Meats/Poultry |
| 22501010 | Canadian bacon, cooked | Lo | Cured Meats/Poultry |
| 22600100 | Bacon, NS as to type of meat, cook | Lo | Cured Meats/Poultry |
| 22600200 | Pork bacon, NS as to fresh, smoke | Lo | Cured Meats/Poultry |
| 22600210 | Pork bacon, NS as to fresh, smoke | Lo | Cured Meats/Poultry |
| 22601000 | Pork bacon, smoked or cured, cook | Lo | Cured Meats/Poultry |
| 22601020 | Pork bacon, smoked or cured, cook | Lo | Cured Meats/Poultry |
| 22601040 | Bacon or side pork, fresh, cooked | Lo | Cured Meats/Poultry |
| 22602010 | Pork bacon, smoked or cured, redu | Lo | Cured Meats/Poultry |
| 22605010 | Pork bacon, formed, lean meat add | Lo | Cured Meats/Poultry |

| 22621000 | Salt pork, cooked | Lo | Cured Meats/Poultry |
| --- | --- | --- | --- |
| 22621100 | Fat back, cooked | Lo | Fats and Oils |
| 22701000 | Pork, spareribs, cooked, NS as to f | Lo | Meats |
| 22701010 | Pork, spareribs, cooked, lean and f | Lo | Meats |
| 22701020 | Pork, spareribs, cooked, lean only e | Lo | Meats |
| 22701030 | Pork, spareribs, barbecued, with sa | Lo | Meats |
| 22701040 | Pork, spareribs, barbecued, with sa | Lo | Meats |
| 22701050 | Pork, spareribs, barbecued, with sa | Lo | Meats |
| 22704010 | Pork, cracklings, cooked | Lo | Meats |
| 22705010 | Pork ears, tail, head, snout, miscell | Lo | Meats |
| 22706010 | Pork, neck bones, cooked | Lo | Meats |
| 22707010 | Pork, pig's feet, cooked | Lo | Meats |
| 22707020 | Pork, pig's feet, pickled | Lo | Meats |
| 22708010 | Pork, pig's hocks, cooked | Lo | Meats |
| 22709010 | Pork skin rinds | Lo | Meats |
| 22709110 | Pork skin, boiled | Lo | Meats |
| 22810010 | Ham, baby food, strained | Lo | Baby Foods |
| 22820000 | Meat stick, baby food | Lo | Baby Foods |
| 23000100 | Lamb, NS as to cut, cooked | Lo | Meats |
| 23101000 | Lamb chop, NS as to cut, cooked, | Lo | Meats |
| 23101010 | Lamb chop, NS as to cut, cooked, l | Lo | Meats |
| 23101020 | Lamb chop, NS as to cut, cooked, l | Lo | Meats |
| 23104000 | Lamb, loin chop, cooked, NS as to | Lo | Meats |
| 23104010 | Lamb, loin chop, cooked, lean and | Lo | Meats |
| 23104020 | Lamb, loin chop, cooked, lean only | Lo | Meats |
| 23107020 | Lamb, shoulder chop, cooked, lean | Lo | Meats |
| 23108020 | Lamb, shoulder, cooked, lean only | Lo | Meats |
| 23110000 | Lamb, ribs, cooked, lean only eaten | Lo | Meats |
| 23110050 | Lamb, ribs, cooked, lean and fat ea | Lo | Meats |
| 23111010 | Lamb hocks, cooked | Lo | Meats |
| 23120100 | Lamb, roast, cooked, NS as to fat e | Lo | Meats |
| 23120110 | Lamb, roast, cooked, lean and fat e | Lo | Meats |
| 23120120 | Lamb, roast, cooked, lean only eate | Lo | Meats |
| 23132000 | Lamb, ground or patty, cooked | Lo | Meats |
| 23150100 | Goat, boiled | Lo | Meats |
| 23150200 | Goat, fried | Lo | Meats |
| 23150250 | Goat, baked | Lo | Meats |
| 23150270 | Goat head, cooked | Lo | Meats |
| 23150300 | Goat ribs, cooked | Lo | Meats |
| 23200100 | Veal, NS as to cut, cooked, NS as t | Lo | Meats |
| 23200110 | Veal, NS as to cut, cooked, lean an | Lo | Meats |
| 23200120 | Veal, NS as to cut, cooked, lean on | Lo | Meats |
| 23201030 | Veal chop, NS as to cooking metho | Lo | Meats |
| 23203020 | Veal chop, fried, lean and fat eaten | Lo | Meats |
| 23203030 | Veal chop, fried, lean only eaten | Lo | Meats |
| 23203110 | Veal chop, broiled, lean and fat eat | Lo | Meats |
| 23203120 | Veal chop, broiled, lean only eaten | Lo | Meats |
| 23204010 | Veal cutlet or steak, NS as to cooki | Lo | Meats |
| 23204030 | Veal cutlet or steak, NS as to cooki | Lo | Meats |
| 23204200 | Veal cutlet or steak, broiled, NS as | Lo | Meats |
| 23204210 | Veal cutlet or steak, broiled, lean a | Lo | Meats |

| 23204220 | Veal cutlet or steak, broiled, lean o | Lo | Meats |
| --- | --- | --- | --- |
| 23205010 | Veal cutlet or steak, fried, NS as to | Lo | Meats |
| 23205020 | Veal cutlet or steak, fried, lean and | Lo | Meats |
| 23205030 | Veal cutlet or steak, fried, lean only | Lo | Meats |
| 23210030 | Veal, roasted, lean only eaten | Lo | Meats |
| 23220010 | Veal, ground or patty, cooked | Lo | Meats |
| 23220020 | Mock chicken legs, cooked | Lo | Meats |
| 23220030 | Veal patty, breaded, cooked | Lo | Meats |
| 23310000 | Rabbit, NS as to domestic or wild, c | Lo | Meats |
| 23311120 | Rabbit, NS as to domestic or wild, b | Lo | Meats |
| 23321000 | Venison/deer, NFS | Lo | Meats |
| 23321100 | Venison/deer, roasted | Lo | Meats |
| 23321200 | Venison/deer steak, cooked, NS as | Lo | Meats |
| 23321250 | Venison/deer steak, breaded or flou | Lo | Meats |
| 23321900 | Venison/deer jerky | Lo | Cured Meats/Poultry |
| 23322100 | Deer sausage | Lo | Cured Meats/Poultry |
| 23322300 | Deer chop, cooked | Lo | Meats |
| 23322350 | Venison/deer ribs, cooked | Lo | Meats |
| 23322400 | Venison/deer, stewed | Lo | Meats |
| 23323100 | Moose, cooked | Lo | Meats |
| 23323500 | Bear, cooked | Lo | Meats |
| 23326100 | Bison, cooked | Lo | Meats |
| 23333100 | Squirrel, cooked | Lo | Meats |
| 23334100 | Beaver, cooked | Lo | Meats |
| 23335100 | Raccoon, cooked | Lo | Meats |
| 23345100 | Wild pig, smoked | Lo | Meats |
| 23420010 | Veal, baby food, strained | Lo | Baby Foods |
| 24100000 | Chicken, NS as to part and cooking | Lo | Poultry |
| 24100010 | Chicken, NS as to part and cooking | Lo | Poultry |
| 24100020 | Chicken, NS as to part and cooking | Lo | Poultry |
| 24102000 | Chicken, NS as to part, baked, broi | Lo | Poultry |
| 24102010 | Chicken, NS as to part, baked, broi | Lo | Poultry |
| 24102020 | Chicken, NS as to part, baked, broi | Lo | Poultry |
| 24102050 | Chicken, NS as to part, rotisserie, N | Lo | Poultry |
| 24102060 | Chicken, NS as to part, rotisserie, s | Lo | Poultry |
| 24102070 | Chicken, NS as to part, rotisserie, s | Lo | Poultry |
| 24103000 | Chicken, NS as to part, stewed, NS | Lo | Poultry |
| 24103010 | Chicken, NS as to part, stewed, ski | Lo | Poultry |
| 24103020 | Chicken, NS as to part, stewed, ski | Lo | Poultry |
| 24103050 | Chicken, NS as to part, grilled witho | Lo | Poultry |
| 24103060 | Chicken, NS as to part, grilled witho | Lo | Poultry |
| 24103070 | Chicken, NS as to part, grilled with | Lo | Poultry |
| 24103075 | Chicken, NS as to part, grilled with | Lo | Poultry |
| 24103080 | Chicken, NS as to part, grilled with | Lo | Poultry |
| 24104000 | Chicken, NS as to part, fried, no co | Lo | Poultry |
| 24104010 | Chicken, NS as to part, fried, no co | Lo | Poultry |
| 24104020 | Chicken, NS as to part, fried, no co | Lo | Poultry |
| 24104051 | Chicken, NS as to part, sauteed, sk | Lo | Poultry |
| 24107000 | Chicken, NS as to part, coated, bak | Lo | Poultry |
| 24107001 | Chicken, NS as to part, coated, bak | Lo | Poultry |
| 24107010 | Chicken, NS as to part, coated, bak | Lo | Poultry |

| 24107020 | Chicken, NS as to part, coated, bak | Lo | Poultry |
| --- | --- | --- | --- |
| 24107040 | Chicken, NS as to part, coated, bak | Lo | Poultry |
| 24107050 | Chicken, NS as to part, coated, bak | Lo | Poultry |
| 24107060 | Chicken, NS as to part, coated, bak | Lo | Poultry |
| 24107070 | Chicken, NS as to part, fried, coate | Lo | Poultry |
| 24107071 | Chicken, NS as to part, fried, coate | Lo | Poultry |
| 24107080 | Chicken, NS as to part, baked, coat | Lo | Poultry |
| 24120100 | Chicken, breast, NS as to cooking | Lo | Poultry |
| 24120110 | Chicken breast, NS as to cooking m | Lo | Poultry |
| 24120120 | Chicken breast, NS as to cooking m | Lo | Poultry |
| 24122100 | Chicken, breast, roasted, broiled, o | Lo | Poultry |
| 24122110 | Chicken, breast, roasted, broiled, o | Lo | Poultry |
| 24122120 | Chicken, breast, roasted, broiled, o | Lo | Poultry |
| 24122130 | Chicken breast, baked, broiled, or r | Lo | Poultry |
| 24122131 | Chicken breast, baked, broiled, or r | Lo | Poultry |
| 24122140 | Chicken breast, baked or broiled, s | Lo | Poultry |
| 24122141 | Chicken breast, baked or broiled, s | Lo | Poultry |
| 24122150 | Chicken breast, baked or broiled, s | Lo | Poultry |
| 24122151 | Chicken breast, baked or broiled, s | Lo | Poultry |
| 24122160 | Chicken breast, baked, broiled, or r | Lo | Poultry |
| 24122161 | Chicken breast, baked, broiled, or r | Lo | Poultry |
| 24122170 | Chicken breast, rotisserie, skin eate | Lo | Poultry |
| 24122171 | Chicken breast, rotisserie, skin not | Lo | Poultry |
| 24123100 | Chicken, breast, stewed, NS as to s | Lo | Poultry |
| 24123110 | Chicken breast, stewed, skin eaten | Lo | Poultry |
| 24123120 | Chicken breast, stewed, skin not ea | Lo | Poultry |
| 24123300 | Chicken breast, grilled without sauc | Lo | Poultry |
| 24123301 | Chicken breast, grilled without sauc | Lo | Poultry |
| 24123310 | Chicken breast, grilled with sauce, | Lo | Poultry |
| 24123311 | Chicken breast, grilled with sauce, | Lo | Poultry |
| 24124100 | Chicken, breast, fried, no coating, N | Lo | Poultry |
| 24124110 | Chicken, breast, fried, no coating, s | Lo | Poultry |
| 24124113 | Chicken, breast, fried, no coating, s | Lo | Poultry |
| 24124115 | Chicken, breast, fried, no coating, s | Lo | Poultry |
| 24124120 | Chicken, breast, fried, no coating, s | Lo | Poultry |
| 24124121 | Chicken, breast, fried, no coating, s | Lo | Poultry |
| 24124122 | Chicken, breast, fried, no coating, s | Lo | Poultry |
| 24124123 | Chicken, breast, fried, no coating, s | Lo | Poultry |
| 24124124 | Chicken, breast, fried, no coating, s | Lo | Poultry |
| 24124125 | Chicken, breast, fried, no coating, s | Lo | Poultry |
| 24124200 | Chicken breast, sauteed, skin eate | Lo | Poultry |
| 24124201 | Chicken breast, sauteed, skin not e | Lo | Poultry |
| 24127100 | Chicken, breast, coated, baked or f | Lo | Poultry |
| 24127110 | Chicken, breast, coated, baked or f | Lo | Poultry |
| 24127112 | Chicken, breast, coated, baked or f | Lo | Poultry |
| 24127113 | Chicken, breast, coated, baked or f | Lo | Poultry |
| 24127115 | Chicken, breast, coated, baked or f | Lo | Poultry |
| 24127120 | Chicken, breast, coated, baked or f | Lo | Poultry |
| 24127125 | Chicken, breast, from fast food, coa | Lo | Poultry |
| 24127130 | Chicken, breast, from fast food, coa | Lo | Poultry |
| 24127135 | Chicken, breast, from fast food, coa | Lo | Poultry |

| 24127140 | Chicken, breast, coated, baked or f | Lo | Poultry |
| --- | --- | --- | --- |
| 24127141 | Chicken, breast, coated, baked or f | Lo | Poultry |
| 24127150 | Chicken, breast, coated, baked or f | Lo | Poultry |
| 24127151 | Chicken, breast, coated, baked or f | Lo | Poultry |
| 24127152 | Chicken, breast, coated, baked or f | Lo | Poultry |
| 24127153 | Chicken, breast, coated, baked or f | Lo | Poultry |
| 24127154 | Chicken, breast, coated, baked or f | Lo | Poultry |
| 24127155 | Chicken, breast, coated, baked or f | Lo | Poultry |
| 24127160 | Chicken, breast, coated, baked or f | Lo | Poultry |
| 24127163 | Chicken, breast, coated, baked or f | Lo | Poultry |
| 24127165 | Chicken, breast, coated, baked or f | Lo | Poultry |
| 24127200 | Chicken breast, fried, coated, skin / | Lo | Poultry |
| 24127201 | Chicken breast, fried, coated, skin / | Lo | Poultry |
| 24127202 | Chicken breast, fried, coated, prepa | Lo | Poultry |
| 24127210 | Chicken breast, fried, coated, skin / | Lo | Poultry |
| 24127211 | Chicken breast, fried, coated, skin / | Lo | Poultry |
| 24127220 | Chicken breast, fried, coated, skin / | Lo | Poultry |
| 24127221 | Chicken breast, fried, coated, skin / | Lo | Poultry |
| 24127500 | Chicken breast, baked, coated, ski | Lo | Poultry |
| 24127501 | Chicken breast, baked, coated, ski | Lo | Poultry |
| 24130200 | Chicken, leg (drumstick and thigh), | Lo | Poultry |
| 24130210 | Chicken leg, drumstick and thigh, N | Lo | Poultry |
| 24130220 | Chicken leg, drumstick and thigh, N | Lo | Poultry |
| 24132200 | Chicken, leg (drumstick and thigh), | Lo | Poultry |
| 24132210 | Chicken, leg (drumstick and thigh), | Lo | Poultry |
| 24132220 | Chicken, leg (drumstick and thigh), | Lo | Poultry |
| 24132230 | Chicken leg, drumstick and thigh, b | Lo | Poultry |
| 24132231 | Chicken leg, drumstick and thigh, b | Lo | Poultry |
| 24132240 | Chicken leg, drumstick and thigh, r | Lo | Poultry |
| 24132241 | Chicken leg, drumstick and thigh, r | Lo | Poultry |
| 24133200 | Chicken, leg (drumstick and thigh), | Lo | Poultry |
| 24133210 | Chicken leg, drumstick and thigh, s | Lo | Poultry |
| 24133220 | Chicken leg, drumstick and thigh, s | Lo | Poultry |
| 24134100 | Chicken leg, drumstick and thigh, g | Lo | Poultry |
| 24134101 | Chicken leg, drumstick and thigh, g | Lo | Poultry |
| 24134150 | Chicken leg, drumstick and thigh, g | Lo | Poultry |
| 24134151 | Chicken leg, drumstick and thigh, g | Lo | Poultry |
| 24134200 | Chicken, leg (drumstick and thigh), | Lo | Poultry |
| 24134210 | Chicken, leg (drumstick and thigh), | Lo | Poultry |
| 24134220 | Chicken, leg (drumstick and thigh), | Lo | Poultry |
| 24134300 | Chicken leg, drumstick and thigh, s | Lo | Poultry |
| 24134301 | Chicken leg, drumstick and thigh, s | Lo | Poultry |
| 24137200 | Chicken, leg (drumstick and thigh), | Lo | Poultry |
| 24137210 | Chicken, leg (drumstick and thigh), | Lo | Poultry |
| 24137211 | Chicken, leg (drumstick and thigh), | Lo | Poultry |
| 24137220 | Chicken, leg (drumstick and thigh), | Lo | Poultry |
| 24137240 | Chicken, leg (drumstick and thigh), | Lo | Poultry |
| 24137250 | Chicken, leg (drumstick and thigh), | Lo | Poultry |
| 24137251 | Chicken, leg (drumstick and thigh), | Lo | Poultry |
| 24137260 | Chicken, leg (drumstick and thigh), | Lo | Poultry |
| 24137300 | Chicken leg, drumstick and thigh, fr | Lo | Poultry |

| 24137301 | Chicken leg, drumstick and thigh, fr | Lo | Poultry |
| --- | --- | --- | --- |
| 24137310 | Chicken leg, drumstick and thigh, b | Lo | Poultry |
| 24137311 | Chicken leg, drumstick and thigh, b | Lo | Poultry |
| 24140200 | Chicken, drumstick, NS as to cooki | Lo | Poultry |
| 24140210 | Chicken drumstick, NS as to cookin | Lo | Poultry |
| 24140220 | Chicken drumstick, NS as to cookin | Lo | Poultry |
| 24142200 | Chicken, drumstick, roasted, broile | Lo | Poultry |
| 24142210 | Chicken, drumstick, roasted, broile | Lo | Poultry |
| 24142220 | Chicken, drumstick, roasted, broile | Lo | Poultry |
| 24142300 | Chicken drumstick, baked, broiled, | Lo | Poultry |
| 24142301 | Chicken drumstick, baked, broiled, | Lo | Poultry |
| 24142310 | Chicken drumstick, baked or broile | Lo | Poultry |
| 24142311 | Chicken drumstick, baked or broile | Lo | Poultry |
| 24142320 | Chicken drumstick, baked or broile | Lo | Poultry |
| 24142321 | Chicken drumstick, baked or broile | Lo | Poultry |
| 24142400 | Chicken drumstick, rotisserie, skin | Lo | Poultry |
| 24142401 | Chicken drumstick, rotisserie, skin | Lo | Poultry |
| 24142500 | Chicken drumstick, grilled without s | Lo | Poultry |
| 24142501 | Chicken drumstick, grilled without s | Lo | Poultry |
| 24142510 | Chicken drumstick, grilled with sau | Lo | Poultry |
| 24142511 | Chicken drumstick, grilled with sau | Lo | Poultry |
| 24143200 | Chicken, drumstick, stewed, NS as | Lo | Poultry |
| 24143210 | Chicken drumstick, stewed, skin ea | Lo | Poultry |
| 24143220 | Chicken drumstick, stewed, skin no | Lo | Poultry |
| 24144200 | Chicken, drumstick, fried, no coatin | Lo | Poultry |
| 24144210 | Chicken, drumstick, fried, no coatin | Lo | Poultry |
| 24144212 | Chicken, drumstick, fried, no coatin | Lo | Poultry |
| 24144213 | Chicken, drumstick, fried, no coatin | Lo | Poultry |
| 24144215 | Chicken, drumstick, fried, no coatin | Lo | Poultry |
| 24144220 | Chicken, drumstick, fried, no coatin | Lo | Poultry |
| 24144221 | Chicken, drumstick, fried, no coatin | Lo | Poultry |
| 24144222 | Chicken, drumstick, fried, no coatin | Lo | Poultry |
| 24144223 | Chicken, drumstick, fried, no coatin | Lo | Poultry |
| 24144225 | Chicken, drumstick, fried, no coatin | Lo | Poultry |
| 24144300 | Chicken drumstick, sauteed, skin e | Lo | Poultry |
| 24144301 | Chicken drumstick, sauteed, skin n | Lo | Poultry |
| 24147200 | Chicken, drumstick, coated, baked | Lo | Poultry |
| 24147210 | Chicken, drumstick, coated, baked | Lo | Poultry |
| 24147212 | Chicken, drumstick, coated, baked | Lo | Poultry |
| 24147213 | Chicken, drumstick, coated, baked | Lo | Poultry |
| 24147215 | Chicken, drumstick, coated, baked | Lo | Poultry |
| 24147220 | Chicken, drumstick, coated, baked | Lo | Poultry |
| 24147223 | Chicken, drumstick, coated, baked | Lo | Poultry |
| 24147225 | Chicken, drumstick, from fast food, | Lo | Poultry |
| 24147230 | Chicken, drumstick, from fast food, | Lo | Poultry |
| 24147235 | Chicken, drumstick, from fast food, | Lo | Poultry |
| 24147240 | Chicken, drumstick, coated, baked | Lo | Poultry |
| 24147250 | Chicken, drumstick, coated, baked | Lo | Poultry |
| 24147251 | Chicken, drumstick, coated, baked | Lo | Poultry |
| 24147252 | Chicken, drumstick, coated, baked | Lo | Poultry |
| 24147253 | Chicken, drumstick, coated, baked | Lo | Poultry |

| 24147255 | Chicken, drumstick, coated, baked | Lo | Poultry |
| --- | --- | --- | --- |
| 24147260 | Chicken, drumstick, coated, baked | Lo | Poultry |
| 24147263 | Chicken, drumstick, coated, baked | Lo | Poultry |
| 24147265 | Chicken, drumstick, coated, baked | Lo | Poultry |
| 24147300 | Chicken drumstick, fried, coated, s | Lo | Poultry |
| 24147301 | Chicken drumstick, fried, coated, s | Lo | Poultry |
| 24147302 | Chicken drumstick, fried, coated, pr | Lo | Poultry |
| 24147310 | Chicken drumstick, fried, coated, s | Lo | Poultry |
| 24147311 | Chicken drumstick, fried, coated, s | Lo | Poultry |
| 24147320 | Chicken drumstick, fried, coated, s | Lo | Poultry |
| 24147321 | Chicken drumstick, fried, coated, s | Lo | Poultry |
| 24147400 | Chicken drumstick, baked, coated, | Lo | Poultry |
| 24147401 | Chicken drumstick, baked, coated, | Lo | Poultry |
| 24150200 | Chicken, thigh, NS as to cooking m | Lo | Poultry |
| 24150210 | Chicken thigh, NS as to cooking me | Lo | Poultry |
| 24150220 | Chicken thigh, NS as to cooking me | Lo | Poultry |
| 24152200 | Chicken, thigh, roasted, broiled, or | Lo | Poultry |
| 24152210 | Chicken, thigh, roasted, broiled, or | Lo | Poultry |
| 24152220 | Chicken, thigh, roasted, broiled, or | Lo | Poultry |
| 24152230 | Chicken thigh, baked, broiled, or ro | Lo | Poultry |
| 24152231 | Chicken thigh, baked, broiled, or ro | Lo | Poultry |
| 24152240 | Chicken thigh, baked or broiled, ski | Lo | Poultry |
| 24152241 | Chicken thigh, baked or broiled, ski | Lo | Poultry |
| 24152250 | Chicken thigh, baked or broiled, ski | Lo | Poultry |
| 24152251 | Chicken thigh, baked or broiled, ski | Lo | Poultry |
| 24152300 | Chicken thigh, rotisserie, skin eaten | Lo | Poultry |
| 24152301 | Chicken thigh, rotisserie, skin not e | Lo | Poultry |
| 24153200 | Chicken, thigh, stewed, NS as to sk | Lo | Poultry |
| 24153210 | Chicken thigh, stewed, skin eaten | Lo | Poultry |
| 24153220 | Chicken thigh, stewed, skin not eat | Lo | Poultry |
| 24154010 | Chicken thigh, grilled without sauce | Lo | Poultry |
| 24154011 | Chicken thigh, grilled without sauce | Lo | Poultry |
| 24154020 | Chicken thigh, grilled with sauce, sk | Lo | Poultry |
| 24154021 | Chicken thigh, grilled with sauce, sk | Lo | Poultry |
| 24154200 | Chicken, thigh, fried, no coating, N | Lo | Poultry |
| 24154210 | Chicken, thigh, fried, no coating, sk | Lo | Poultry |
| 24154211 | Chicken, thigh, fried, no coating, sk | Lo | Poultry |
| 24154212 | Chicken, thigh, fried, no coating, sk | Lo | Poultry |
| 24154213 | Chicken, thigh, fried, no coating, sk | Lo | Poultry |
| 24154214 | Chicken, thigh, fried, no coating, sk | Lo | Poultry |
| 24154215 | Chicken, thigh, fried, no coating, sk | Lo | Poultry |
| 24154220 | Chicken, thigh, fried, no coating, sk | Lo | Poultry |
| 24154221 | Chicken, thigh, fried, no coating, sk | Lo | Poultry |
| 24154222 | Chicken, thigh, fried, no coating, sk | Lo | Poultry |
| 24154223 | Chicken, thigh, fried, no coating, sk | Lo | Poultry |
| 24154224 | Chicken, thigh, fried, no coating, sk | Lo | Poultry |
| 24154225 | Chicken, thigh, fried, no coating, sk | Lo | Poultry |
| 24154300 | Chicken thigh, sauteed, skin eaten | Lo | Poultry |
| 24154301 | Chicken thigh, sauteed, skin not ea | Lo | Poultry |
| 24157200 | Chicken, thigh, coated, baked or fri | Lo | Poultry |
| 24157210 | Chicken, thigh, coated, baked or fri | Lo | Poultry |

| 24157213 | Chicken, thigh, coated, baked or fri | Lo | Poultry |
| --- | --- | --- | --- |
| 24157215 | Chicken, thigh, coated, baked or fri | Lo | Poultry |
| 24157220 | Chicken, thigh, coated, baked or fri | Lo | Poultry |
| 24157223 | Chicken, thigh, coated, baked or fri | Lo | Poultry |
| 24157225 | Chicken, thigh, from fast food, coat | Lo | Poultry |
| 24157230 | Chicken, thigh, from fast food, coat | Lo | Poultry |
| 24157235 | Chicken, thigh, from fast food, coat | Lo | Poultry |
| 24157240 | Chicken, thigh, coated, baked or fri | Lo | Poultry |
| 24157250 | Chicken, thigh, coated, baked or fri | Lo | Poultry |
| 24157253 | Chicken, thigh, coated, baked or fri | Lo | Poultry |
| 24157255 | Chicken, thigh, coated, baked or fri | Lo | Poultry |
| 24157260 | Chicken, thigh, coated, baked or fri | Lo | Poultry |
| 24157263 | Chicken, thigh, coated, baked or fri | Lo | Poultry |
| 24157300 | Chicken thigh, fried, coated, skin / c | Lo | Poultry |
| 24157301 | Chicken thigh, fried, coated, skin / c | Lo | Poultry |
| 24157302 | Chicken thigh, fried, coated, prepar | Lo | Poultry |
| 24157310 | Chicken thigh, fried, coated, skin / c | Lo | Poultry |
| 24157311 | Chicken thigh, fried, coated, skin / c | Lo | Poultry |
| 24157320 | Chicken thigh, fried, coated, skin / c | Lo | Poultry |
| 24157321 | Chicken thigh, fried, coated, skin / c | Lo | Poultry |
| 24157330 | Chicken thigh, fried, coated, skin / c | Lo | Poultry |
| 24157331 | Chicken thigh, fried, coated, skin / c | Lo | Poultry |
| 24157400 | Chicken thigh, baked, coated, skin | Lo | Poultry |
| 24157401 | Chicken thigh, baked, coated, skin | Lo | Poultry |
| 24160100 | Chicken, wing, NS as to cooking m | Lo | Poultry |
| 24160110 | Chicken wing, NS as to cooking me | Lo | Poultry |
| 24160120 | Chicken, wing, NS as to cooking m | Lo | Poultry |
| 24162100 | Chicken, wing, roasted, broiled, or | Lo | Poultry |
| 24162110 | Chicken, wing, roasted, broiled, or | Lo | Poultry |
| 24162120 | Chicken, wing, roasted, broiled, or | Lo | Poultry |
| 24162130 | Chicken wing, baked, broiled, or ro | Lo | Poultry |
| 24162140 | Chicken wing, baked or broiled, fro | Lo | Poultry |
| 24162150 | Chicken wing, baked or broiled, fro | Lo | Poultry |
| 24162200 | Chicken wing, rotisserie | Lo | Poultry |
| 24163100 | Chicken, wing, stewed, NS as to sk | Lo | Poultry |
| 24163110 | Chicken wing, stewed | Lo | Poultry |
| 24163120 | Chicken, wing, stewed, skin not eat | Lo | Poultry |
| 24164000 | Chicken wing, grilled without sauce | Lo | Poultry |
| 24164010 | Chicken wing, grilled with sauce | Lo | Poultry |
| 24164100 | Chicken, wing, fried, no coating, NS | Lo | Poultry |
| 24164110 | Chicken, wing, fried, no coating, ski | Lo | Poultry |
| 24164111 | Chicken, wing, fried, no coating, ski | Lo | Poultry |
| 24164112 | Chicken, wing, fried, no coating, ski | Lo | Poultry |
| 24164113 | Chicken, wing, fried, no coating, ski | Lo | Poultry |
| 24164115 | Chicken, wing, fried, no coating, ski | Lo | Poultry |
| 24164120 | Chicken, wing, fried, no coating, ski | Lo | Poultry |
| 24164122 | Chicken, wing, fried, no coating, ski | Lo | Poultry |
| 24164123 | Chicken, wing, fried, no coating, ski | Lo | Poultry |
| 24164125 | Chicken, wing, fried, no coating, ski | Lo | Poultry |
| 24164200 | Chicken wing, sauteed | Lo | Poultry |
| 24167100 | Chicken, wing, coated, baked or fri | Lo | Poultry |

| 24167110 | Chicken, wing, coated, baked or fri | Lo | Poultry |
| --- | --- | --- | --- |
| 24167113 | Chicken, wing, coated, baked or fri | Lo | Poultry |
| 24167115 | Chicken, wing, coated, baked or fri | Lo | Poultry |
| 24167119 | Chicken, wing, coated, baked or fri | Lo | Poultry |
| 24167120 | Chicken, wing, coated, baked or fri | Lo | Poultry |
| 24167123 | Chicken, wing, coated, baked or fri | Lo | Poultry |
| 24167125 | Chicken, wing, from fast food, coat | Lo | Poultry |
| 24167130 | Chicken, wing, from fast food, coat | Lo | Poultry |
| 24167135 | Chicken, wing, from fast food, coat | Lo | Poultry |
| 24167200 | Chicken wing, fried, coated, from ra | Lo | Poultry |
| 24167210 | Chicken wing, fried, coated, from p | Lo | Poultry |
| 24167220 | Chicken wing, fried, coated, from fa | Lo | Poultry |
| 24167230 | Chicken wing, fried, coated, from re | Lo | Poultry |
| 24167300 | Chicken wing, baked, coated | Lo | Poultry |
| 24168000 | Chicken "wings" with hot sauce, fro | Lo | Poultry |
| 24168001 | Chicken "wings" with other sauces | Lo | Poultry |
| 24168002 | Chicken "wings", plain, from fast fo | Lo | Poultry |
| 24168010 | Chicken "wings" with hot sauce, fro | Lo | Poultry |
| 24168011 | Chicken "wings" with other sauces | Lo | Poultry |
| 24168012 | Chicken "wings", plain, from precoo | Lo | Poultry |
| 24168020 | Chicken "wings" with hot sauce, fro | Lo | Poultry |
| 24168021 | Chicken "wings" with other sauces | Lo | Poultry |
| 24168022 | Chicken "wings", plain, from other s | Lo | Poultry |
| 24168030 | Chicken "wings", boneless, with hot | Lo | Poultry |
| 24168031 | Chicken "wings", boneless, with hot | Lo | Poultry |
| 24170200 | Chicken, back | Lo | Poultry |
| 24180200 | Chicken, neck or ribs | Lo | Poultry |
| 24198440 | Chicken skin | Lo | Poultry |
| 24198500 | Chicken feet | Lo | Poultry |
| 24198570 | Chicken, canned, meat only | Lo | Poultry |
| 24198670 | Chicken, chicken roll, roasted | Lo | Poultry |
| 24198671 | Chicken patty, breaded | Lo | Poultry |
| 24198677 | Chicken fillet, breaded | Lo | Poultry |
| 24198683 | Chicken fillet, grilled | Lo | Poultry |
| 24198690 | Chicken patty, fillet, or tenders, bre | Lo | Poultry |
| 24198695 | Chicken patty, fillet, or tenders, bre | Lo | Poultry |
| 24198700 | Chicken patty, fillet, or tenders, bre | Lo | Poultry |
| 24198710 | Chicken patty with cheese, breaded | Lo | Poultry |
| 24198720 | Chicken, ground | Lo | Poultry |
| 24198729 | Chicken nuggets, NFS | Lo | Poultry |
| 24198730 | Chicken nuggets, from fast food / r | Lo | Poultry |
| 24198731 | Chicken nuggets, from fast food | Lo | Poultry |
| 24198732 | Chicken nuggets, from restaurant | Lo | Poultry |
| 24198735 | Chicken nuggets, from school lunc | Lo | Poultry |
| 24198736 | Chicken nuggets, from frozen | Lo | Poultry |
| 24198737 | Chicken nuggets, from other sourc | Lo | Poultry |
| 24198739 | Chicken tenders or strips, NFS | Lo | Poultry |
| 24198740 | Chicken nuggets | Lo | Poultry |
| 24198741 | Chicken tenders or strips, breaded, | Lo | Poultry |
| 24198742 | Chicken tenders or strips, breaded, | Lo | Poultry |
| 24198745 | Chicken tenders or strips, breaded, | Lo | Poultry |

| 24198746 | Chicken tenders or strips, breaded, | Lo | Poultry |
| --- | --- | --- | --- |
| 24198747 | Chicken tenders or strips, breaded, | Lo | Poultry |
| 24198840 | Fried chicken chunks, Puerto Rican | Lo | Poultry |
| 24201000 | Turkey, NFS | Lo | Poultry |
| 24201010 | Turkey, light meat, cooked, NS as t | Lo | Poultry |
| 24201020 | Turkey, light meat, skin not eaten | Lo | Poultry |
| 24201030 | Turkey, light meat, skin eaten | Lo | Poultry |
| 24201050 | Turkey, light meat, breaded, baked | Lo | Poultry |
| 24201060 | Turkey, light meat, breaded, baked | Lo | Poultry |
| 24201070 | Turkey, light meat, breaded, baked | Lo | Poultry |
| 24201110 | Turkey, light meat, roasted, NS as t | Lo | Poultry |
| 24201120 | Turkey, light meat, roasted, skin no | Lo | Poultry |
| 24201130 | Turkey, light meat, roasted, skin ea | Lo | Poultry |
| 24201210 | Turkey, dark meat, roasted, NS as | Lo | Poultry |
| 24201220 | Turkey, dark meat, roasted, skin no | Lo | Poultry |
| 24201230 | Turkey, dark meat, roasted, skin ea | Lo | Poultry |
| 24201310 | Turkey, light and dark meat, roaste | Lo | Poultry |
| 24201320 | Turkey, light and dark meat, roaste | Lo | Poultry |
| 24201330 | Turkey, light and dark meat, roaste | Lo | Poultry |
| 24201350 | Turkey, light or dark meat, battered | Lo | Poultry |
| 24201360 | Turkey, light or dark meat, fried, co | Lo | Poultry |
| 24201370 | Turkey, light or dark meat, fried, co | Lo | Poultry |
| 24201400 | Turkey, light or dark meat, stewed, | Lo | Poultry |
| 24201410 | Turkey, light or dark meat, stewed, | Lo | Poultry |
| 24201420 | Turkey light or dark meat, stewed, s | Lo | Poultry |
| 24201500 | Turkey, light or dark meat, smoked | Lo | Poultry |
| 24201510 | Turkey, light or dark meat, smoked | Lo | Poultry |
| 24201520 | Turkey, light or dark meat, smoked | Lo | Poultry |
| 24202000 | Turkey, drumstick, cooked, NS as t | Lo | Poultry |
| 24202010 | Turkey, drumstick, cooked, skin not | Lo | Poultry |
| 24202020 | Turkey, drumstick, cooked, skin eat | Lo | Poultry |
| 24202050 | Turkey, drumstick, roasted, NS as t | Lo | Poultry |
| 24202060 | Turkey, drumstick, roasted, skin no | Lo | Poultry |
| 24202070 | Turkey, drumstick, roasted, skin ea | Lo | Poultry |
| 24202120 | Turkey, drumstick, smoked, skin ea | Lo | Poultry |
| 24202450 | Turkey, thigh, cooked, NS as to ski | Lo | Poultry |
| 24202460 | Turkey, thigh, cooked, skin eaten | Lo | Poultry |
| 24202500 | Turkey, thigh, cooked, skin not eate | Lo | Poultry |
| 24202600 | Turkey, neck | Lo | Poultry |
| 24203000 | Turkey, wing, cooked, NS as to ski | Lo | Poultry |
| 24203010 | Turkey, wing, cooked, skin not eate | Lo | Poultry |
| 24203020 | Turkey, wing, cooked, skin eaten | Lo | Poultry |
| 24203120 | Turkey, wing, smoked, skin eaten | Lo | Poultry |
| 24204000 | Turkey, rolled roast, light or dark m | Lo | Poultry |
| 24205000 | Turkey, tail | Lo | Poultry |
| 24207000 | Turkey, ground | Lo | Poultry |
| 24208000 | Turkey, nuggets | Lo | Poultry |
| 24208500 | Turkey bacon, cooked | Lo | Cured Meats/Poultry |
| 24208510 | Turkey bacon, reduced sodium, co | Lo | Cured Meats/Poultry |
| 24209000 | Turkey with barbecue sauce, skin e | Lo | Mixed Dishes - Meat, Poultry, Seafood |
| 24209001 | Turkey with barbecue sauce, skin n | Lo | Mixed Dishes - Meat, Poultry, Seafood |

| 24300100 | Duck, cooked, NS as to skin eaten | Lo | Poultry |
| --- | --- | --- | --- |
| 24300110 | Duck, cooked, skin eaten | Lo | Poultry |
| 24300120 | Duck, cooked, skin not eaten | Lo | Poultry |
| 24301000 | Duck, roasted, NS as to skin eaten | Lo | Poultry |
| 24301010 | Duck, roasted, skin eaten | Lo | Poultry |
| 24301020 | Duck, roasted, skin not eaten | Lo | Poultry |
| 24301210 | Duck, coated, fried | Lo | Poultry |
| 24302010 | Duck, pressed, Chinese | Lo | Poultry |
| 24400000 | Cornish game hen, cooked, NS as | Lo | Poultry |
| 24400010 | Cornish game hen, cooked, skin ea | Lo | Poultry |
| 24400020 | Cornish game hen, cooked, skin no | Lo | Poultry |
| 24401010 | Cornish game hen, roasted, skin ea | Lo | Poultry |
| 24401020 | Cornish game hen, roasted, skin no | Lo | Poultry |
| 24402100 | Dove, cooked, NS as to cooking m | Lo | Poultry |
| 24403100 | Quail, cooked | Lo | Poultry |
| 24404100 | Pheasant, cooked | Lo | Poultry |
| 24701000 | Chicken, baby food, NS as to strain | Lo | Baby Foods |
| 24701010 | Chicken, baby food, strained | Lo | Baby Foods |
| 24701020 | Chicken, baby food, junior | Lo | Baby Foods |
| 24703000 | Turkey, baby food, NS as to straine | Lo | Baby Foods |
| 24703010 | Turkey, baby food, strained | Lo | Baby Foods |
| 24703020 | Turkey, baby food, junior | Lo | Baby Foods |
| 24705010 | Chicken stick, baby food | Lo | Baby Foods |
| 24706010 | Turkey stick, baby food | Lo | Baby Foods |
| 25110120 | Beef liver, braised | Lo | Meats |
| 25110140 | Beef liver, fried | Lo | Meats |
| 25110420 | Chicken liver, braised | Lo | Meats |
| 25110450 | Chicken liver, fried | Lo | Meats |
| 25112200 | Liver paste or pate, chicken | Lo | Meats |
| 25120000 | Heart, cooked | Lo | Meats |
| 25130000 | Kidney, cooked | Lo | Meats |
| 25150000 | Brains, cooked | Lo | Meats |
| 25160000 | Tongue, cooked | Lo | Meats |
| 25160110 | Tongue, smoked, cured, or pickled, | Lo | Cured Meats/Poultry |
| 25170110 | Tripe, cooked | Lo | Meats |
| 25170210 | Chitterlings, cooked | Lo | Meats |
| 25170310 | Hog maws, cooked | Lo | Meats |
| 25170420 | Gizzard, cooked | Lo | Meats |
| 25210110 | Frankfurter or hot dog, NFS | Lo | Cured Meats/Poultry |
| 25210150 | Frankfurter or hot dog, cheese-filled | Lo | Cured Meats/Poultry |
| 25210170 | Frankfurter or hot dog, chili-filled | Lo | Cured Meats/Poultry |
| 25210210 | Frankfurter or hot dog, beef | Lo | Cured Meats/Poultry |
| 25210220 | Frankfurter or hot dog, beef and po | Lo | Cured Meats/Poultry |
| 25210230 | Frankfurter or hot dog, beef and po | Lo | Cured Meats/Poultry |
| 25210240 | Frankfurter or hot dog, beef and po | Lo | Cured Meats/Poultry |
| 25210250 | Frankfurter or hot dog, meat and po | Lo | Cured Meats/Poultry |
| 25210280 | Frankfurter or hot dog, meat and po | Lo | Cured Meats/Poultry |
| 25210290 | Frankfurter or hot dog, meat and po | Lo | Cured Meats/Poultry |
| 25210310 | Frankfurter or hot dog, chicken | Lo | Cured Meats/Poultry |
| 25210410 | Frankfurter or hot dog, turkey | Lo | Cured Meats/Poultry |
| 25210510 | Frankfurter or hot dog, low salt | Lo | Cured Meats/Poultry |

| 25210610 | Frankfurter or hot dog, beef, lowfat | Lo | Cured Meats/Poultry |
| --- | --- | --- | --- |
| 25210620 | Frankfurter or hot dog, beef, reduce | Lo | Cured Meats/Poultry |
| 25210700 | Frankfurter or hot dog, meat & poul | Lo | Cured Meats/Poultry |
| 25220010 | Cold cut, NFS | Lo | Cured Meats/Poultry |
| 25220100 | Beef sausage, NFS | Lo | Cured Meats/Poultry |
| 25220105 | Beef sausage | Lo | Cured Meats/Poultry |
| 25220106 | Beef sausage, reduced fat | Lo | Cured Meats/Poultry |
| 25220108 | Beef sausage, reduced sodium | Lo | Cured Meats/Poultry |
| 25220110 | Beef sausage, brown and serve, lin | Lo | Cured Meats/Poultry |
| 25220120 | Beef sausage, smoked, stick | Lo | Cured Meats/Poultry |
| 25220130 | Beef sausage, smoked | Lo | Cured Meats/Poultry |
| 25220140 | Beef sausage, fresh, bulk, patty or l | Lo | Cured Meats/Poultry |
| 25220150 | Beef sausage with cheese | Lo | Cured Meats/Poultry |
| 25220210 | Blood sausage | Lo | Cured Meats/Poultry |
| 25220350 | Bratwurst | Lo | Cured Meats/Poultry |
| 25220360 | Bratwurst, with cheese | Lo | Cured Meats/Poultry |
| 25220370 | Bratwurst, beef, cooked | Lo | Cured Meats/Poultry |
| 25220390 | Bologna, beef, lowfat | Lo | Cured Meats/Poultry |
| 25220400 | Bologna, pork and beef | Lo | Cured Meats/Poultry |
| 25220410 | Bologna, NFS | Lo | Cured Meats/Poultry |
| 25220420 | Bologna, Lebanon | Lo | Cured Meats/Poultry |
| 25220425 | Bologna, made from any kind of me | Lo | Cured Meats/Poultry |
| 25220430 | Bologna, beef | Lo | Cured Meats/Poultry |
| 25220435 | Bologna, made from any kind of me | Lo | Cured Meats/Poultry |
| 25220440 | Bologna, turkey | Lo | Cured Meats/Poultry |
| 25220445 | Bologna, made from any kind of me | Lo | Cured Meats/Poultry |
| 25220460 | Bologna, pork | Lo | Cured Meats/Poultry |
| 25220470 | Bologna, beef, lower sodium | Lo | Cured Meats/Poultry |
| 25220480 | Bologna, chicken, beef, and pork | Lo | Cured Meats/Poultry |
| 25220500 | Bologna, beef and pork, lowfat | Lo | Cured Meats/Poultry |
| 25220510 | Capicola | Lo | Cured Meats/Poultry |
| 25220650 | Turkey or chicken and beef sausag | Lo | Cured Meats/Poultry |
| 25220710 | Chorizo | Lo | Cured Meats/Poultry |
| 25220910 | Head cheese | Lo | Cured Meats/Poultry |
| 25221110 | Knockwurst | Lo | Cured Meats/Poultry |
| 25221210 | Mortadella | Lo | Cured Meats/Poultry |
| 25221215 | Pastrami, NFS | Lo | Cured Meats/Poultry |
| 25221220 | Pastrami, made from any kind of m | Lo | Cured Meats/Poultry |
| 25221250 | Pepperoni, NFS | Lo | Cured Meats/Poultry |
| 25221255 | Pepperoni, reduced fat | Lo | Cured Meats/Poultry |
| 25221260 | Pepperoni, reduced sodium | Lo | Cured Meats/Poultry |
| 25221310 | Polish sausage | Lo | Cured Meats/Poultry |
| 25221350 | Italian sausage | Lo | Cured Meats/Poultry |
| 25221400 | Sausage, NFS | Lo | Cured Meats/Poultry |
| 25221405 | Pork sausage | Lo | Cured Meats/Poultry |
| 25221406 | Pork sausage, reduced fat | Lo | Cured Meats/Poultry |
| 25221408 | Pork sausage, reduced sodium | Lo | Cured Meats/Poultry |
| 25221410 | Pork sausage, fresh, bulk, patty or l | Lo | Cured Meats/Poultry |
| 25221420 | Pork sausage, brown and serve, co | Lo | Cured Meats/Poultry |
| 25221430 | Pork sausage, country style, fresh, | Lo | Cured Meats/Poultry |
| 25221450 | Pork sausage rice links | Lo | Cured Meats/Poultry |

| 25221460 | Pork and beef sausage | Lo | Cured Meats/Poultry |
| --- | --- | --- | --- |
| 25221470 | Pork and beef sausage, brown and | Lo | Cured Meats/Poultry |
| 25221500 | Salami, NFS | Lo | Cured Meats/Poultry |
| 25221505 | Salami, made from any type of mea | Lo | Cured Meats/Poultry |
| 25221510 | Salami, soft, cooked | Lo | Cured Meats/Poultry |
| 25221515 | Salami, made from any type of mea | Lo | Cured Meats/Poultry |
| 25221520 | Salami, dry or hard | Lo | Cured Meats/Poultry |
| 25221530 | Salami, beef | Lo | Cured Meats/Poultry |
| 25221610 | Scrapple, cooked | Lo | Cured Meats/Poultry |
| 25221650 | Smoked link sausage, pork | Lo | Cured Meats/Poultry |
| 25221660 | Smoked link sausage, pork and be | Lo | Cured Meats/Poultry |
| 25221680 | Smoked sausage, pork | Lo | Cured Meats/Poultry |
| 25221810 | Thuringer | Lo | Cured Meats/Poultry |
| 25221830 | Turkey or chicken sausage | Lo | Cured Meats/Poultry |
| 25221840 | Turkey breakfast sausage, bulk, pa | Lo | Cured Meats/Poultry |
| 25221850 | Turkey sausage, smoked | Lo | Cured Meats/Poultry |
| 25221855 | Turkey or chicken sausage, reduce | Lo | Cured Meats/Poultry |
| 25221860 | Turkey or chicken sausage, reduce | Lo | Cured Meats/Poultry |
| 25221870 | Turkey or chicken and pork sausag | Lo | Cured Meats/Poultry |
| 25221875 | Turkey or chicken, pork, and beef s | Lo | Cured Meats/Poultry |
| 25221880 | Turkey or chicken, pork, and beef s | Lo | Cured Meats/Poultry |
| 25221890 | Turkey, pork, and beef sausage, lo | Lo | Cured Meats/Poultry |
| 25221910 | Vienna sausage, canned | Lo | Cured Meats/Poultry |
| 25221920 | Vienna sausage, chicken, canned | Lo | Cured Meats/Poultry |
| 25221950 | Pickled sausage | Lo | Cured Meats/Poultry |
| 25230110 | Luncheon meat, NFS | Lo | Cured Meats/Poultry |
| 25230210 | Ham, prepackaged or deli, luncheo | Lo | Cured Meats/Poultry |
| 25230220 | Ham, prepackaged or deli, luncheo | Lo | Cured Meats/Poultry |
| 25230230 | Ham, sliced, extra lean, prepackag | Lo | Cured Meats/Poultry |
| 25230235 | Ham, sliced, extra lean, lower sodiu | Lo | Cured Meats/Poultry |
| 25230310 | Chicken or turkey loaf, prepackage | Lo | Cured Meats/Poultry |
| 25230320 | Chicken, prepackaged or deli, lunc | Lo | Cured Meats/Poultry |
| 25230340 | Chicken, prepackaged or deli, lunc | Lo | Cured Meats/Poultry |
| 25230410 | Ham loaf, luncheon meat | Lo | Cured Meats/Poultry |
| 25230420 | Ham luncheon meat, loaf type | Lo | Cured Meats/Poultry |
| 25230430 | Ham and cheese loaf | Lo | Cured Meats/Poultry |
| 25230450 | Honey loaf | Lo | Cured Meats/Poultry |
| 25230510 | Ham, luncheon meat, chopped, min | Lo | Cured Meats/Poultry |
| 25230520 | Ham, luncheon meat, chopped, min | Lo | Cured Meats/Poultry |
| 25230530 | Ham and pork, canned luncheon m | Lo | Cured Meats/Poultry |
| 25230540 | Ham, pork and chicken, canned lun | Lo | Cured Meats/Poultry |
| 25230550 | Ham, pork, and chicken, canned lu | Lo | Cured Meats/Poultry |
| 25230560 | Liverwurst | Lo | Cured Meats/Poultry |
| 25230610 | Luncheon meat, loaf type | Lo | Cured Meats/Poultry |
| 25230710 | Sandwich loaf, luncheon meat | Lo | Cured Meats/Poultry |
| 25230780 | Turkey, prepackaged or deli, lunch | Lo | Cured Meats/Poultry |
| 25230785 | Turkey, prepackaged or deli, lunch | Lo | Cured Meats/Poultry |
| 25230790 | Turkey ham, sliced, extra lean, pre | Lo | Cured Meats/Poultry |
| 25230800 | Turkey ham, prepackaged or deli, l | Lo | Cured Meats/Poultry |
| 25230810 | Veal loaf | Lo | Cured Meats/Poultry |
| 25230820 | Turkey pastrami | Lo | Cured Meats/Poultry |

| 25230840 | Turkey salami | Lo | Cured Meats/Poultry |
| --- | --- | --- | --- |
| 25230900 | Turkey or chicken breast, prepacka | Lo | Cured Meats/Poultry |
| 25230905 | Turkey or chicken breast, low salt, | Lo | Cured Meats/Poultry |
| 25231110 | Beef, prepackaged or deli, luncheo | Lo | Cured Meats/Poultry |
| 25231120 | Beef, prepackaged or deli, luncheo | Lo | Cured Meats/Poultry |
| 25231150 | Corned beef, pressed | Lo | Cured Meats/Poultry |
| 25240000 | Meat spread or potted meat, NFS | Lo | Cured Meats/Poultry |
| 25240110 | Chicken salad spread | Lo | Cured Meats/Poultry |
| 25240220 | Ham salad spread | Lo | Cured Meats/Poultry |
| 25240310 | Roast beef spread | Lo | Cured Meats/Poultry |
| 26100100 | Fish, NS as to type, raw | Lo | Seafood |
| 26100110 | Fish, NS as to type, cooked, NS as | Lo | Seafood |
| 26100120 | Fish, NS as to type, baked or broile | Lo | Seafood |
| 26100121 | Fish, NS as to type, baked or broile | Lo | Seafood |
| 26100122 | Fish, NS as to type, baked or broile | Lo | Seafood |
| 26100123 | Fish, NS as to type, baked or broile | Lo | Seafood |
| 26100130 | Fish, NS as to type, coated, baked | Lo | Seafood |
| 26100133 | Fish, NS as to type, coated, baked | Lo | Seafood |
| 26100140 | Fish, NS as to type, coated, fried, m | Lo | Seafood |
| 26100142 | Fish, NS as to type, coated, fried, m | Lo | Seafood |
| 26100143 | Fish, NS as to type, coated, fried, n | Lo | Seafood |
| 26100150 | Fish, NS as to type, battered, fried | Lo | Seafood |
| 26100160 | Fish, NS as to type, steamed | Lo | Seafood |
| 26100170 | Fish, NS as to type, dried | Lo | Seafood |
| 26100180 | Fish, NS as to type, canned | Lo | Seafood |
| 26100190 | Fish, NS as to type, smoked | Lo | Seafood |
| 26100200 | Fish, NS as to type, from fast food | Lo | Seafood |
| 26100210 | Fish stick, patty, or fillet, NS as to ty | Lo | Seafood |
| 26100220 | Fish stick, patty, or fillet, NS as to ty | Lo | Seafood |
| 26100230 | Fish stick, patty, or fillet, NS as to ty | Lo | Seafood |
| 26100240 | Fish stick, patty, or fillet, NS as to ty | Lo | Seafood |
| 26100250 | Fish stick, patty, or fillet, NS as to ty | Lo | Seafood |
| 26100260 | Fish stick, patty or nugget from fast | Lo | Seafood |
| 26100270 | Fish stick, patty or nugget from rest | Lo | Seafood |
| 26101110 | Anchovy, cooked, NS as to cooking | Lo | Seafood |
| 26101180 | Anchovy, canned | Lo | Seafood |
| 26105110 | Carp, cooked, NS as to cooking me | Lo | Seafood |
| 26105120 | Carp, baked or broiled, fat added | Lo | Seafood |
| 26105140 | Carp, coated, fried | Lo | Seafood |
| 26105160 | Carp, steamed or poached | Lo | Seafood |
| 26107110 | Catfish, cooked, NS as to cooking | Lo | Seafood |
| 26107120 | Catfish, baked or broiled, made wit | Lo | Seafood |
| 26107121 | Catfish, baked or broiled, made wit | Lo | Seafood |
| 26107123 | Catfish, baked or broiled, no added | Lo | Seafood |
| 26107124 | Catfish, baked or broiled, made wit | Lo | Seafood |
| 26107130 | Catfish, coated, baked or broiled, m | Lo | Seafood |
| 26107131 | Catfish, coated, baked or broiled, m | Lo | Seafood |
| 26107133 | Catfish, coated, baked or broiled, n | Lo | Seafood |
| 26107140 | Catfish, coated, fried, made with oil | Lo | Seafood |
| 26107143 | Catfish, coated, fried, no added fat | Lo | Seafood |
| 26107144 | Catfish, coated, fried, made with co | Lo | Seafood |

| 26107150 | Catfish, battered, fried | Lo | Seafood |
| --- | --- | --- | --- |
| 26107160 | Catfish, steamed or poached | Lo | Seafood |
| 26109110 | Cod, cooked, NS as to cooking met | Lo | Seafood |
| 26109120 | Cod, baked or broiled, made with o | Lo | Seafood |
| 26109121 | Cod, baked or broiled, made with b | Lo | Seafood |
| 26109122 | Cod, baked or broiled, made with m | Lo | Seafood |
| 26109123 | Cod, baked or broiled, no added fat | Lo | Seafood |
| 26109124 | Cod, baked or broiled, made with c | Lo | Seafood |
| 26109130 | Cod, coated, baked or broiled, mad | Lo | Seafood |
| 26109133 | Cod, coated, baked or broiled, no a | Lo | Seafood |
| 26109134 | Cod, coated, baked or broiled, mad | Lo | Seafood |
| 26109140 | Cod, coated, fried, made with oil | Lo | Seafood |
| 26109141 | Cod, coated, fried, made with butte | Lo | Seafood |
| 26109143 | Cod, coated, fried, no added fat | Lo | Seafood |
| 26109144 | Cod, coated, fried, made with cooki | Lo | Seafood |
| 26109150 | Cod, battered, fried | Lo | Seafood |
| 26109160 | Cod, steamed or poached | Lo | Seafood |
| 26109170 | Cod, dried, salted | Lo | Seafood |
| 26109180 | Cod, dried, salted, salt removed in | Lo | Seafood |
| 26111110 | Croaker, cooked, NS as to cooking | Lo | Seafood |
| 26111120 | Croaker, baked or broiled, fat adde | Lo | Seafood |
| 26111121 | Croaker, baked or broiled, no adde | Lo | Seafood |
| 26111130 | Croaker, coated, baked or broiled, f | Lo | Seafood |
| 26111140 | Croaker, coated, fried | Lo | Seafood |
| 26111160 | Croaker, steamed or poached | Lo | Seafood |
| 26113110 | Eel, cooked, NS as to cooking met | Lo | Seafood |
| 26113160 | Eel, steamed or poached | Lo | Seafood |
| 26113190 | Eel, smoked | Lo | Seafood |
| 26115110 | Flounder, cooked, NS as to cooking | Lo | Seafood |
| 26115120 | Flounder, baked or broiled, made w | Lo | Seafood |
| 26115121 | Flounder, baked or broiled, made w | Lo | Seafood |
| 26115122 | Flounder, baked or broiled, made w | Lo | Seafood |
| 26115123 | Flounder, baked or broiled, no adde | Lo | Seafood |
| 26115124 | Flounder, baked or broiled, made w | Lo | Seafood |
| 26115130 | Flounder, coated, baked or broiled, | Lo | Seafood |
| 26115132 | Flounder, coated, baked or broiled, | Lo | Seafood |
| 26115133 | Flounder, coated, baked or broiled, | Lo | Seafood |
| 26115140 | Flounder, coated, fried, made with | Lo | Seafood |
| 26115141 | Flounder, coated, fried, made with | Lo | Seafood |
| 26115150 | Flounder, battered, fried | Lo | Seafood |
| 26115160 | Flounder, steamed or poached | Lo | Seafood |
| 26115190 | Flounder, smoked | Lo | Seafood |
| 26117110 | Haddock, cooked, NS as to cooking | Lo | Seafood |
| 26117120 | Haddock, baked or broiled, fat adde | Lo | Seafood |
| 26117121 | Haddock, baked or broiled, no adde | Lo | Seafood |
| 26117130 | Haddock, coated, baked or broiled, | Lo | Seafood |
| 26117131 | Haddock, coated, baked or broiled, | Lo | Seafood |
| 26117140 | Haddock, coated, fried | Lo | Seafood |
| 26117150 | Haddock, battered, fried | Lo | Seafood |
| 26117160 | Haddock, steamed or poached | Lo | Seafood |
| 26118020 | Halibut, baked or broiled, made wit | Lo | Seafood |

| 26118023 | Halibut, baked or broiled, no added | Lo | Seafood |
| --- | --- | --- | --- |
| 26118024 | Halibut, baked or broiled, made wit | Lo | Seafood |
| 26118030 | Halibut, coated, baked or broiled, m | Lo | Seafood |
| 26118050 | Halibut, steamed or poached | Lo | Seafood |
| 26119100 | Herring, raw | Lo | Seafood |
| 26119110 | Herring, cooked, NS as to cooking | Lo | Seafood |
| 26119120 | Herring, baked or broiled, fat added | Lo | Seafood |
| 26119121 | Herring, baked or broiled, no added | Lo | Seafood |
| 26119130 | Herring, coated, baked or broiled, f | Lo | Seafood |
| 26119131 | Herring, coated, baked or broiled, n | Lo | Seafood |
| 26119140 | Herring, coated, fried | Lo | Seafood |
| 26119160 | Herring, pickled, in cream sauce | Lo | Seafood |
| 26119180 | Herring, pickled | Lo | Seafood |
| 26119190 | Herring, smoked, kippered | Lo | Seafood |
| 26121100 | Mackerel, raw | Lo | Seafood |
| 26121110 | Mackerel, cooked, NS as to cookin | Lo | Seafood |
| 26121120 | Mackerel, baked or broiled, fat add | Lo | Seafood |
| 26121121 | Mackerel, baked or broiled, no add | Lo | Seafood |
| 26121140 | Mackerel, coated, fried | Lo | Seafood |
| 26121160 | Mackerel, pickled | Lo | Seafood |
| 26121180 | Mackerel, canned | Lo | Seafood |
| 26123120 | Mullet, baked or broiled, fat added | Lo | Seafood |
| 26123121 | Mullet, baked or broiled, no added f | Lo | Seafood |
| 26123140 | Mullet, coated, fried | Lo | Seafood |
| 26123160 | Mullet, steamed or poached | Lo | Seafood |
| 26125110 | Ocean perch, cooked, NS as to coo | Lo | Seafood |
| 26125120 | Ocean perch, baked or broiled, fat | Lo | Seafood |
| 26125121 | Ocean perch, baked or broiled, no | Lo | Seafood |
| 26125130 | Ocean perch, coated, baked or broi | Lo | Seafood |
| 26125140 | Ocean perch, coated, fried | Lo | Seafood |
| 26125150 | Ocean perch, battered, fried | Lo | Seafood |
| 26125160 | Ocean perch, steamed or poached | Lo | Seafood |
| 26127110 | Perch, cooked, NS as to cooking m | Lo | Seafood |
| 26127120 | Perch, baked or broiled, made with | Lo | Seafood |
| 26127121 | Perch, baked or broiled, made with | Lo | Seafood |
| 26127123 | Perch, baked or broiled, no added f | Lo | Seafood |
| 26127130 | Perch, coated, baked or broiled, ma | Lo | Seafood |
| 26127133 | Perch, coated, baked or broiled, no | Lo | Seafood |
| 26127140 | Perch, coated, fried, made with oil | Lo | Seafood |
| 26127141 | Perch, coated, fried, made with butt | Lo | Seafood |
| 26127143 | Perch, coated, fried, no added fat | Lo | Seafood |
| 26127150 | Perch, battered, fried | Lo | Seafood |
| 26127160 | Perch, steamed or poached | Lo | Seafood |
| 26129120 | Pike, baked or broiled, fat added | Lo | Seafood |
| 26129140 | Pike, coated, fried | Lo | Seafood |
| 26131100 | Pompano, raw | Lo | Seafood |
| 26131110 | Pompano, cooked, NS as to cookin | Lo | Seafood |
| 26131120 | Pompano, baked or broiled, fat add | Lo | Seafood |
| 26131121 | Pompano, baked or broiled, no add | Lo | Seafood |
| 26131131 | Pompano, coated, baked or broiled | Lo | Seafood |
| 26131140 | Pompano, coated, fried | Lo | Seafood |

| 26131150 | Pompano, battered, fried | Lo | Seafood |
| --- | --- | --- | --- |
| 26131160 | Pompano, steamed or poached | Lo | Seafood |
| 26131190 | Pompano, smoked | Lo | Seafood |
| 26133110 | Porgy, cooked, NS as to cooking m | Lo | Seafood |
| 26133120 | Porgy, baked or broiled, fat added | Lo | Seafood |
| 26133121 | Porgy, baked or broiled, no added f | Lo | Seafood |
| 26133130 | Porgy, coated, baked or broiled, fat | Lo | Seafood |
| 26133140 | Porgy, coated, fried | Lo | Seafood |
| 26133150 | Porgy, battered, fried | Lo | Seafood |
| 26133160 | Porgy, steamed or poached | Lo | Seafood |
| 26135120 | Ray, baked or broiled, fat added | Lo | Seafood |
| 26137100 | Salmon, raw | Lo | Seafood |
| 26137110 | Salmon, cooked, NS as to cooking | Lo | Seafood |
| 26137120 | Salmon, baked or broiled, made wit | Lo | Seafood |
| 26137121 | Salmon, baked or broiled, made wit | Lo | Seafood |
| 26137122 | Salmon, baked or broiled, made wit | Lo | Seafood |
| 26137123 | Salmon, baked or broiled, no added | Lo | Seafood |
| 26137124 | Salmon, baked or broiled, made wit | Lo | Seafood |
| 26137130 | Salmon, coated, baked or broiled, | Lo | Seafood |
| 26137131 | Salmon, coated, baked or broiled, | Lo | Seafood |
| 26137133 | Salmon, coated, baked or broiled, n | Lo | Seafood |
| 26137134 | Salmon, coated, baked or broiled, | Lo | Seafood |
| 26137140 | Salmon, coated, fried, made with oi | Lo | Seafood |
| 26137141 | Salmon, coated, fried, made with b | Lo | Seafood |
| 26137142 | Salmon, coated, fried, made with m | Lo | Seafood |
| 26137143 | Salmon, coated, fried, no added fat | Lo | Seafood |
| 26137150 | Salmon, battered, fried | Lo | Seafood |
| 26137160 | Salmon, steamed or poached | Lo | Seafood |
| 26137170 | Salmon, dried | Lo | Seafood |
| 26137180 | Salmon, canned | Lo | Seafood |
| 26137190 | Salmon, smoked | Lo | Seafood |
| 26139110 | Sardines, cooked | Lo | Seafood |
| 26139170 | Sardines, dried | Lo | Seafood |
| 26139180 | Sardines, canned in oil | Lo | Seafood |
| 26139190 | Sardines, skinless, boneless, packe | Lo | Seafood |
| 26141110 | Sea bass, cooked, NS as to cookin | Lo | Seafood |
| 26141120 | Sea bass, baked or broiled, fat add | Lo | Seafood |
| 26141121 | Sea bass, baked or broiled, no add | Lo | Seafood |
| 26141130 | Sea bass, coated, baked or broiled | Lo | Seafood |
| 26141140 | Sea bass, coated, fried | Lo | Seafood |
| 26141160 | Sea bass, steamed or poached | Lo | Seafood |
| 26143110 | Shark, cooked, NS as to cooking m | Lo | Seafood |
| 26143120 | Shark, baked or broiled, fat added | Lo | Seafood |
| 26143160 | Shark, steamed or poached | Lo | Seafood |
| 26145140 | Smelt, floured or breaded, fried | Lo | Seafood |
| 26147110 | Sturgeon, cooked, NS as to cookin | Lo | Seafood |
| 26149110 | Swordfish, cooked, NS as to cookin | Lo | Seafood |
| 26149120 | Swordfish, baked or broiled, fat add | Lo | Seafood |
| 26149121 | Swordfish, baked or broiled, no add | Lo | Seafood |
| 26149130 | Swordfish, coated, baked or broiled | Lo | Seafood |
| 26149140 | Swordfish, coated, fried | Lo | Seafood |

| 26149160 | Swordfish, steamed or poached | Lo | Seafood |
| --- | --- | --- | --- |
| 26151110 | Trout, cooked, NS as to cooking m | Lo | Seafood |
| 26151120 | Trout, baked or broiled, made with | Lo | Seafood |
| 26151121 | Trout, baked or broiled, made with | Lo | Seafood |
| 26151122 | Trout, baked or broiled, made with | Lo | Seafood |
| 26151123 | Trout, baked or broiled, no added f | Lo | Seafood |
| 26151130 | Trout, coated, baked or broiled, ma | Lo | Seafood |
| 26151133 | Trout, coated, baked or broiled, no | Lo | Seafood |
| 26151140 | Trout, coated, fried, made with oil | Lo | Seafood |
| 26151142 | Trout, coated, fried, made with mar | Lo | Seafood |
| 26151143 | Trout, coated, fried, no added fat | Lo | Seafood |
| 26151150 | Trout, battered, fried | Lo | Seafood |
| 26151160 | Trout, steamed or poached | Lo | Seafood |
| 26151190 | Trout, smoked | Lo | Seafood |
| 26153110 | Tuna, fresh, cooked, NS as to cook | Lo | Seafood |
| 26153120 | Tuna, fresh, baked or broiled, fat ad | Lo | Seafood |
| 26153122 | Tuna, fresh, baked or broiled, no ad | Lo | Seafood |
| 26153130 | Tuna, fresh, coated, baked or broile | Lo | Seafood |
| 26153131 | Tuna, fresh, coated, baked or broile | Lo | Seafood |
| 26153140 | Tuna, fresh, coated, fried | Lo | Seafood |
| 26153160 | Tuna, fresh, steamed or poached | Lo | Seafood |
| 26155110 | Tuna, canned, NS as to oil or water | Lo | Seafood |
| 26155180 | Tuna, canned, oil pack | Lo | Seafood |
| 26155190 | Tuna, canned, water pack | Lo | Seafood |
| 26157110 | Whiting, cooked, NS as to cooking | Lo | Seafood |
| 26157120 | Whiting, baked or broiled, made wit | Lo | Seafood |
| 26157121 | Whiting, baked or broiled, made wit | Lo | Seafood |
| 26157122 | Whiting, baked or broiled, made wit | Lo | Seafood |
| 26157123 | Whiting, baked or broiled, no added | Lo | Seafood |
| 26157124 | Whiting, baked or broiled, made wit | Lo | Seafood |
| 26157130 | Whiting, coated, baked or broiled, | Lo | Seafood |
| 26157132 | Whiting, coated, baked or broiled, | Lo | Seafood |
| 26157133 | Whiting, coated, baked or broiled, n | Lo | Seafood |
| 26157140 | Whiting, coated, fried, made with oi | Lo | Seafood |
| 26157150 | Whiting, battered, fried | Lo | Seafood |
| 26157160 | Whiting, steamed or poached | Lo | Seafood |
| 26158000 | Tilapia, cooked, NS as to cooking m | Lo | Seafood |
| 26158010 | Tilapia, baked or broiled, made with | Lo | Seafood |
| 26158011 | Tilapia, baked or broiled, made with | Lo | Seafood |
| 26158012 | Tilapia, baked or broiled, made with | Lo | Seafood |
| 26158013 | Tilapia, baked or broiled, no added | Lo | Seafood |
| 26158014 | Tilapia, baked or broiled, made with | Lo | Seafood |
| 26158020 | Tilapia, coated, baked or broiled, m | Lo | Seafood |
| 26158021 | Tilapia, coated, baked or broiled, m | Lo | Seafood |
| 26158023 | Tilapia, coated, baked or broiled, no | Lo | Seafood |
| 26158024 | Tilapia, coated, baked or broiled, m | Lo | Seafood |
| 26158030 | Tilapia, coated, fried, made with oil | Lo | Seafood |
| 26158031 | Tilapia, coated, fried, made with bu | Lo | Seafood |
| 26158032 | Tilapia, coated, fried, made with ma | Lo | Seafood |
| 26158033 | Tilapia, coated, fried, no added fat | Lo | Seafood |
| 26158034 | Tilapia, coated, fried, made with co | Lo | Seafood |

| 26158040 | Tilapia, battered, fried | Lo | Seafood |
| --- | --- | --- | --- |
| 26158050 | Tilapia, steamed or poached | Lo | Seafood |
| 26203110 | Frog legs, NS as to cooking metho | Lo | Seafood |
| 26205110 | Octopus, cooked, NS as to cooking | Lo | Seafood |
| 26205160 | Octopus, steamed | Lo | Seafood |
| 26207110 | Roe, shad, cooked | Lo | Seafood |
| 26211100 | Roe, sturgeon | Lo | Seafood |
| 26213100 | Squid, raw | Lo | Seafood |
| 26213120 | Squid, baked or broiled, fat added | Lo | Seafood |
| 26213140 | Squid, coated, fried | Lo | Seafood |
| 26213160 | Squid, steamed or boiled | Lo | Seafood |
| 26213170 | Squid, dried | Lo | Seafood |
| 26213190 | Squid, canned | Lo | Seafood |
| 26215120 | Turtle, cooked, NS as to cooking m | Lo | Seafood |
| 26301110 | Abalone, cooked, NS as to cooking | Lo | Seafood |
| 26301140 | Abalone, floured or breaded, fried | Lo | Seafood |
| 26303100 | Clams, raw | Lo | Seafood |
| 26303110 | Clams, cooked, NS as to cooking m | Lo | Seafood |
| 26303120 | Clams, baked or broiled, fat added | Lo | Seafood |
| 26303121 | Clams, baked or broiled, no added | Lo | Seafood |
| 26303140 | Clams, coated, fried | Lo | Seafood |
| 26303150 | Clams, battered, fried | Lo | Seafood |
| 26303160 | Clams, steamed or boiled | Lo | Seafood |
| 26303180 | Clams, canned | Lo | Seafood |
| 26305110 | Crab, cooked, NS as to cooking me | Lo | Seafood |
| 26305120 | Crab, baked or broiled, fat added | Lo | Seafood |
| 26305121 | Crab, baked or broiled, no added fa | Lo | Seafood |
| 26305130 | Crab, coated, baked or broiled, fat | Lo | Seafood |
| 26305160 | Crab, hard shell, steamed | Lo | Seafood |
| 26305180 | Crab, canned | Lo | Seafood |
| 26307140 | Crab, soft shell, coated, fried | Lo | Seafood |
| 26309140 | Crayfish, coated, fried | Lo | Seafood |
| 26309160 | Crayfish, boiled or steamed | Lo | Seafood |
| 26311110 | Lobster, cooked, NS as to cooking | Lo | Seafood |
| 26311120 | Lobster, baked or broiled, fat added | Lo | Seafood |
| 26311121 | Lobster, baked or broiled, no added | Lo | Seafood |
| 26311140 | Lobster, coated, fried | Lo | Seafood |
| 26311160 | Lobster, steamed or boiled | Lo | Seafood |
| 26313100 | Mussels, raw | Lo | Seafood |
| 26313110 | Mussels, cooked, NS as to cooking | Lo | Seafood |
| 26313160 | Mussels, steamed or poached | Lo | Seafood |
| 26315110 | Oysters, cooked, NS as to cooking | Lo | Seafood |
| 26315120 | Oysters, baked or broiled, fat added | Lo | Seafood |
| 26315121 | Oysters, baked or broiled, no added | Lo | Seafood |
| 26315130 | Oysters, steamed | Lo | Seafood |
| 26315140 | Oysters, coated, fried | Lo | Seafood |
| 26315150 | Oysters, battered, fried | Lo | Seafood |
| 26315160 | Oysters, coated, baked or broiled, f | Lo | Seafood |
| 26315180 | Oysters, canned | Lo | Seafood |
| 26315190 | Oysters, smoked | Lo | Seafood |
| 26317110 | Scallops, cooked, NS as to cooking | Lo | Seafood |

| 26317120 | Scallops, baked or broiled, fat adde | Lo | Seafood |
| --- | --- | --- | --- |
| 26317121 | Scallops, baked or broiled, no adde | Lo | Seafood |
| 26317130 | Scallops, steamed or boiled | Lo | Seafood |
| 26317140 | Scallops, coated, fried | Lo | Seafood |
| 26317150 | Scallops, battered, fried | Lo | Seafood |
| 26317160 | Scallops, coated, baked or broiled, | Lo | Seafood |
| 26319110 | Shrimp, cooked, NS as to cooking | Lo | Seafood |
| 26319120 | Shrimp, baked or broiled, made wit | Lo | Seafood |
| 26319121 | Shrimp, baked or broiled, made wit | Lo | Seafood |
| 26319122 | Shrimp, baked or broiled, made wit | Lo | Seafood |
| 26319123 | Shrimp, baked or broiled, no added | Lo | Seafood |
| 26319124 | Shrimp, baked or broiled, made wit | Lo | Seafood |
| 26319130 | Shrimp, steamed or boiled | Lo | Seafood |
| 26319140 | Shrimp, coated, fried, made with oil | Lo | Seafood |
| 26319141 | Shrimp, coated, fried, made with bu | Lo | Seafood |
| 26319142 | Shrimp, coated, fried, made with m | Lo | Seafood |
| 26319143 | Shrimp, coated, fried, no added fat | Lo | Seafood |
| 26319145 | Shrimp, coated, fried, from fast foo | Lo | Seafood |
| 26319160 | Shrimp, coated, baked or broiled, m | Lo | Seafood |
| 26319161 | Shrimp, coated, baked or broiled, m | Lo | Seafood |
| 26319163 | Shrimp, coated, baked or broiled, n | Lo | Seafood |
| 26319164 | Shrimp, coated, baked or broiled, m | Lo | Seafood |
| 26319170 | Shrimp, dried | Lo | Seafood |
| 26319180 | Shrimp, canned | Lo | Seafood |
| 26321110 | Snails, cooked, NS as to cooking m | Lo | Seafood |
| 27111000 | Beef with tomato-based sauce | Lo | Mixed Dishes - Meat, Poultry, Seafood |
| 27111050 | Spaghetti sauce with beef or meat | Lo | Condiments and Sauces |
| 27111100 | Beef goulash | Lo | Mixed Dishes - Meat, Poultry, Seafood |
| 27111200 | Beef burgundy | Lo | Mixed Dishes - Meat, Poultry, Seafood |
| 27111300 | Beef stew, no potatoes, tomato-bas | Lo | Mixed Dishes - Meat, Poultry, Seafood |
| 27111310 | Beef stew, no potatoes, tomato-bas | Lo | Mixed Dishes - Meat, Poultry, Seafood |
| 27111400 | Chili con carne, NS as to beans | Lo | Mixed Dishes - Meat, Poultry, Seafood |
| 27111405 | Chili con carne with beans, from re | Lo | Mixed Dishes - Meat, Poultry, Seafood |
| 27111406 | Chili con carne with beans, home re | Lo | Mixed Dishes - Meat, Poultry, Seafood |
| 27111407 | Chili con carne with beans, canned | Lo | Mixed Dishes - Meat, Poultry, Seafood |
| 27111410 | Chili con carne with beans | Lo | Mixed Dishes - Meat, Poultry, Seafood |
| 27111420 | Chili con carne without beans | Lo | Mixed Dishes - Meat, Poultry, Seafood |
| 27111430 | Chili con carne, NS as to beans, wit | Lo | Mixed Dishes - Meat, Poultry, Seafood |
| 27111440 | Chili con carne with beans and che | Lo | Mixed Dishes - Meat, Poultry, Seafood |
| 27111500 | Beef sloppy joe, no bun | Lo | Mixed Dishes - Meat, Poultry, Seafood |
| 27112000 | Beef with gravy | Lo | Mixed Dishes - Meat, Poultry, Seafood |
| 27112010 | Salisbury steak with gravy | Lo | Mixed Dishes - Meat, Poultry, Seafood |
| 27113000 | Beef with cream or white sauce | Lo | Mixed Dishes - Meat, Poultry, Seafood |
| 27113100 | Beef stroganoff | Lo | Mixed Dishes - Meat, Poultry, Seafood |
| 27113200 | Creamed chipped or dried beef | Lo | Mixed Dishes - Meat, Poultry, Seafood |
| 27113300 | Swedish meatballs with cream or w | Lo | Mixed Dishes - Meat, Poultry, Seafood |
| 27114000 | Beef with mushroom sauce | Lo | Mixed Dishes - Meat, Poultry, Seafood |
| 27115000 | Beef with soy-based sauce | Lo | Mixed Dishes - Asian |
| 27115100 | Steak teriyaki | Lo | Mixed Dishes - Asian |
| 27116100 | Beef curry | Lo | Mixed Dishes - Meat, Poultry, Seafood |
| 27116110 | Beef curry with rice | Lo | Mixed Dishes - Meat, Poultry, Seafood |

| 27116200 | Beef with barbecue sauce | Lo | Mixed Dishes - Meat, Poultry, Seafood |
| --- | --- | --- | --- |
| 27116300 | Beef with sweet and sour sauce | Lo | Mixed Dishes - Asian |
| 27116350 | Stewed seasoned ground beef, Me | Lo | Mixed Dishes - Meat, Poultry, Seafood |
| 27118110 | Meatballs, Puerto Rican style | Lo | Mixed Dishes - Meat, Poultry, Seafood |
| 27118120 | Stewed seasoned ground beef, Pue | Lo | Mixed Dishes - Meat, Poultry, Seafood |
| 27118130 | Stewed dried beef, Puerto Rican st | Lo | Mixed Dishes - Meat, Poultry, Seafood |
| 27118180 | Beef stew, meat with gravy, no pota | Lo | Mixed Dishes - Meat, Poultry, Seafood |
| 27120020 | Ham or pork with gravy | Lo | Mixed Dishes - Meat, Poultry, Seafood |
| 27120030 | Ham or pork with barbecue sauce | Lo | Mixed Dishes - Meat, Poultry, Seafood |
| 27120060 | Sweet and sour pork | Lo | Mixed Dishes - Asian |
| 27120080 | Ham stroganoff | Lo | Mixed Dishes - Meat, Poultry, Seafood |
| 27120090 | Ham or pork with mushroom sauce | Lo | Mixed Dishes - Meat, Poultry, Seafood |
| 27120100 | Ham or pork with tomato-based sa | Lo | Mixed Dishes - Meat, Poultry, Seafood |
| 27120110 | Sausage with tomato-based sauce | Lo | Mixed Dishes - Meat, Poultry, Seafood |
| 27120120 | Sausage gravy | Lo | Mixed Dishes - Meat, Poultry, Seafood |
| 27120130 | Pork stew, no potatoes, tomato-bas | Lo | Mixed Dishes - Meat, Poultry, Seafood |
| 27120150 | Pork or ham with soy-based sauce | Lo | Mixed Dishes - Asian |
| 27120160 | Pork curry | Lo | Mixed Dishes - Meat, Poultry, Seafood |
| 27120210 | Frankfurter or hot dog, with chili, no | Lo | Mixed Dishes - Meat, Poultry, Seafood |
| 27120250 | Frankfurters or hot dogs with tomat | Lo | Mixed Dishes - Meat, Poultry, Seafood |
| 27121000 | Pork with chili and tomatoes | Lo | Mixed Dishes - Meat, Poultry, Seafood |
| 27121010 | Stewed pork, Puerto Rican style | Lo | Mixed Dishes - Meat, Poultry, Seafood |
| 27121410 | Chili con carne with beans, made w | Lo | Mixed Dishes - Meat, Poultry, Seafood |
| 27130010 | Lamb or mutton with gravy | Lo | Mixed Dishes - Meat, Poultry, Seafood |
| 27130040 | Spaghetti sauce with lamb or mutto | Lo | Condiments and Sauces |
| 27130100 | Lamb or mutton curry | Lo | Mixed Dishes - Meat, Poultry, Seafood |
| 27133010 | Stewed goat, Puerto Rican style | Lo | Mixed Dishes - Meat, Poultry, Seafood |
| 27135010 | Veal with gravy | Lo | Mixed Dishes - Meat, Poultry, Seafood |
| 27135040 | Veal with butter sauce | Lo | Mixed Dishes - Meat, Poultry, Seafood |
| 27135050 | Veal Marsala | Lo | Mixed Dishes - Meat, Poultry, Seafood |
| 27135110 | Veal parmigiana | Lo | Mixed Dishes - Meat, Poultry, Seafood |
| 27136050 | Venison or deer with tomato-based | Lo | Mixed Dishes - Meat, Poultry, Seafood |
| 27136080 | Venison or deer with gravy | Lo | Mixed Dishes - Meat, Poultry, Seafood |
| 27136100 | Chili con carne with venison/deer a | Lo | Mixed Dishes - Meat, Poultry, Seafood |
| 27141000 | Chicken or turkey cacciatore | Lo | Mixed Dishes - Meat, Poultry, Seafood |
| 27141030 | Spaghetti sauce with poultry | Lo | Condiments and Sauces |
| 27141035 | Spaghetti sauce with poultry and ad | Lo | Condiments and Sauces |
| 27141050 | Stewed chicken with tomato-based | Lo | Mixed Dishes - Meat, Poultry, Seafood |
| 27141500 | Chili con carne with chicken or turk | Lo | Mixed Dishes - Meat, Poultry, Seafood |
| 27142000 | Chicken with gravy | Lo | Mixed Dishes - Meat, Poultry, Seafood |
| 27142100 | Chicken or turkey fricassee | Lo | Mixed Dishes - Meat, Poultry, Seafood |
| 27142200 | Turkey with gravy | Lo | Mixed Dishes - Meat, Poultry, Seafood |
| 27143000 | Chicken or turkey with cream sauce | Lo | Mixed Dishes - Meat, Poultry, Seafood |
| 27144000 | Chicken or turkey with mushroom s | Lo | Mixed Dishes - Meat, Poultry, Seafood |
| 27145000 | Chicken or turkey with teriyaki | Lo | Mixed Dishes - Asian |
| 27146000 | Chicken or turkey with barbecue sa | Lo | Poultry |
| 27146010 | Chicken or turkey with barbecue sa | Lo | Poultry |
| 27146011 | Chicken, shredded or pulled, with b | Lo | Mixed Dishes - Meat, Poultry, Seafood |
| 27146050 | Chicken wing with hot pepper sauc | Lo | Poultry |
| 27146100 | Sweet and sour chicken or turkey | Lo | Mixed Dishes - Asian |
| 27146110 | Sweet and sour chicken or turkey, | Lo | Mixed Dishes - Asian |

| 27146150 | Chicken curry | Lo | Mixed Dishes - Meat, Poultry, Seafood |
| --- | --- | --- | --- |
| 27146155 | Chicken curry with rice | Lo | Mixed Dishes - Meat, Poultry, Seafood |
| 27146160 | Chicken with mole sauce | Lo | Mixed Dishes - Meat, Poultry, Seafood |
| 27146200 | Chicken or turkey with cheese sauc | Lo | Mixed Dishes - Meat, Poultry, Seafood |
| 27146250 | Chicken or turkey cordon bleu | Lo | Mixed Dishes - Meat, Poultry, Seafood |
| 27146300 | Chicken or turkey parmigiana | Lo | Mixed Dishes - Meat, Poultry, Seafood |
| 27146350 | Orange chicken | Lo | Mixed Dishes - Asian |
| 27146360 | Sesame chicken | Lo | Mixed Dishes - Asian |
| 27146400 | Chicken kiev | Lo | Mixed Dishes - Meat, Poultry, Seafood |
| 27148010 | Stuffed chicken, drumstick or breas | Lo | Mixed Dishes - Meat, Poultry, Seafood |
| 27150010 | Fish with cream or white sauce, not | Lo | Mixed Dishes - Meat, Poultry, Seafood |
| 27150020 | Crab, deviled | Lo | Mixed Dishes - Meat, Poultry, Seafood |
| 27150030 | Crab imperial | Lo | Mixed Dishes - Meat, Poultry, Seafood |
| 27150060 | Lobster newburg | Lo | Mixed Dishes - Meat, Poultry, Seafood |
| 27150070 | Lobster with butter sauce | Lo | Seafood |
| 27150100 | Shrimp curry | Lo | Mixed Dishes - Meat, Poultry, Seafood |
| 27150110 | Shrimp cocktail | Lo | Seafood |
| 27150120 | Tuna with cream or white sauce | Lo | Mixed Dishes - Meat, Poultry, Seafood |
| 27150130 | Seafood newburg | Lo | Mixed Dishes - Meat, Poultry, Seafood |
| 27150140 | Seafood sauce | Lo | Seafood |
| 27150151 | Spaghetti sauce with seafood | Lo | Condiments and Sauces |
| 27150155 | Spaghetti sauce with seafood and a | Lo | Condiments and Sauces |
| 27150160 | Shrimp with lobster sauce | Lo | Mixed Dishes - Meat, Poultry, Seafood |
| 27150170 | Sweet and sour shrimp | Lo | Mixed Dishes - Asian |
| 27150190 | Lobster sauce | Lo | Condiments and Sauces |
| 27150200 | Oyster sauce | Lo | Condiments and Sauces |
| 27150210 | Fish sauce | Lo | Condiments and Sauces |
| 27150230 | Shrimp scampi | Lo | Seafood |
| 27150250 | Fish moochim | Lo | Mixed Dishes - Meat, Poultry, Seafood |
| 27150310 | Fish with tomato-based sauce | Lo | Mixed Dishes - Meat, Poultry, Seafood |
| 27150320 | Fish curry | Lo | Mixed Dishes - Meat, Poultry, Seafood |
| 27150325 | Fish curry with rice | Lo | Mixed Dishes - Meat, Poultry, Seafood |
| 27150330 | Mussels with tomato-based sauce | Lo | Mixed Dishes - Meat, Poultry, Seafood |
| 27150350 | Sardines with tomato-based sauce | Lo | Mixed Dishes - Meat, Poultry, Seafood |
| 27150370 | Sardines with mustard sauce | Lo | Mixed Dishes - Meat, Poultry, Seafood |
| 27150410 | Shrimp teriyaki | Lo | Mixed Dishes - Asian |
| 27150510 | Scallops with cheese sauce | Lo | Mixed Dishes - Meat, Poultry, Seafood |
| 27151040 | Crabs in tomato-based sauce, Puer | Lo | Mixed Dishes - Meat, Poultry, Seafood |
| 27151050 | Shrimp in garlic sauce, Puerto Rica | Lo | Mixed Dishes - Meat, Poultry, Seafood |
| 27151070 | Stewed codfish, no potatoes, Puert | Lo | Mixed Dishes - Meat, Poultry, Seafood |
| 27160010 | Meat with barbecue sauce, NS as t | Lo | Mixed Dishes - Meat, Poultry, Seafood |
| 27160100 | Meatballs, NS as to type of meat, w | Lo | Mixed Dishes - Meat, Poultry, Seafood |
| 27162010 | Meat with tomato-based sauce | Lo | Mixed Dishes - Meat, Poultry, Seafood |
| 27162040 | Spaghetti sauce with meat | Lo | Condiments and Sauces |
| 27162050 | Spaghetti sauce with combination o | Lo | Condiments and Sauces |
| 27162060 | Spaghetti sauce with meat and add | Lo | Condiments and Sauces |
| 27162500 | Stewed, seasoned, ground beef an | Lo | Mixed Dishes - Meat, Poultry, Seafood |
| 27163010 | Meat with gravy, NS as to type of m | Lo | Mixed Dishes - Meat, Poultry, Seafood |
| 27211000 | Beef and potatoes, no sauce | Lo | Mixed Dishes - Meat, Poultry, Seafood |
| 27211100 | Beef stew with potatoes, tomato-ba | Lo | Mixed Dishes - Meat, Poultry, Seafood |
| 27211110 | Beef stew with potatoes, tomato-ba | Lo | Mixed Dishes - Meat, Poultry, Seafood |

| 27211150 | Beef goulash with potatoes | Lo | Mixed Dishes - Meat, Poultry, Seafood |
| --- | --- | --- | --- |
| 27211190 | Beef and potatoes with cream sauc | Lo | Mixed Dishes - Meat, Poultry, Seafood |
| 27211200 | Beef stew with potatoes, gravy | Lo | Mixed Dishes - Meat, Poultry, Seafood |
| 27211300 | Beef, roast, hash | Lo | Mixed Dishes - Meat, Poultry, Seafood |
| 27211400 | Corned beef hash | Lo | Mixed Dishes - Meat, Poultry, Seafood |
| 27211500 | Beef and potatoes with cheese sau | Lo | Mixed Dishes - Meat, Poultry, Seafood |
| 27211550 | Stewed, seasoned, ground beef wit | Lo | Mixed Dishes - Meat, Poultry, Seafood |
| 27212000 | Beef and noodles, no sauce | Lo | Mixed Dishes - Meat, Poultry, Seafood |
| 27212050 | Beef and macaroni with cheese sau | Lo | Mixed Dishes - Meat, Poultry, Seafood |
| 27212100 | Beef and noodles with tomato-base | Lo | Mixed Dishes - Meat, Poultry, Seafood |
| 27212120 | Chili con carne with beans and mac | Lo | Mixed Dishes - Meat, Poultry, Seafood |
| 27212150 | Beef goulash with noodles | Lo | Mixed Dishes - Meat, Poultry, Seafood |
| 27212200 | Beef and noodles with gravy | Lo | Mixed Dishes - Meat, Poultry, Seafood |
| 27212300 | Beef and noodles with cream or wh | Lo | Mixed Dishes - Meat, Poultry, Seafood |
| 27212350 | Beef stroganoff with noodles | Lo | Mixed Dishes - Meat, Poultry, Seafood |
| 27212400 | Beef and noodles with mushroom s | Lo | Mixed Dishes - Meat, Poultry, Seafood |
| 27212500 | Beef and noodles with soy-based s | Lo | Mixed Dishes - Asian |
| 27213000 | Beef and rice, no sauce | Lo | Mixed Dishes - Meat, Poultry, Seafood |
| 27213010 | Biryani with meat | Lo | Mixed Dishes - Grain-based |
| 27213100 | Beef and rice with tomato-based sa | Lo | Mixed Dishes - Meat, Poultry, Seafood |
| 27213120 | Porcupine balls with tomato-based | Lo | Mixed Dishes - Meat, Poultry, Seafood |
| 27213150 | Chili con carne with beans and rice | Lo | Mixed Dishes - Meat, Poultry, Seafood |
| 27213200 | Beef and rice with gravy | Lo | Mixed Dishes - Meat, Poultry, Seafood |
| 27213300 | Beef and rice with cream sauce | Lo | Mixed Dishes - Meat, Poultry, Seafood |
| 27213400 | Beef and rice with mushroom sauc | Lo | Mixed Dishes - Meat, Poultry, Seafood |
| 27213420 | Porcupine balls with mushroom sau | Lo | Mixed Dishes - Meat, Poultry, Seafood |
| 27213500 | Beef and rice with soy-based sauce | Lo | Mixed Dishes - Asian |
| 27213600 | Beef and rice with cheese sauce | Lo | Mixed Dishes - Meat, Poultry, Seafood |
| 27214100 | Meat loaf made with beef | Lo | Mixed Dishes - Meat, Poultry, Seafood |
| 27214110 | Meat loaf made with beef, with tom | Lo | Mixed Dishes - Meat, Poultry, Seafood |
| 27218210 | Beef stew with potatoes, Puerto Ric | Lo | Mixed Dishes - Meat, Poultry, Seafood |
| 27218310 | Stewed corned beef, Puerto Rican | Lo | Mixed Dishes - Meat, Poultry, Seafood |
| 27220010 | Meat loaf made with ham | Lo | Mixed Dishes - Meat, Poultry, Seafood |
| 27220020 | Ham and noodles with cream or wh | Lo | Mixed Dishes - Meat, Poultry, Seafood |
| 27220030 | Ham and rice with mushroom sauc | Lo | Mixed Dishes - Meat, Poultry, Seafood |
| 27220050 | Ham or pork with stuffing | Lo | Mixed Dishes - Meat, Poultry, Seafood |
| 27220080 | Ham croquette | Lo | Mixed Dishes - Meat, Poultry, Seafood |
| 27220110 | Pork and rice with tomato-based sa | Lo | Mixed Dishes - Meat, Poultry, Seafood |
| 27220120 | Sausage and rice with tomato-base | Lo | Mixed Dishes - Meat, Poultry, Seafood |
| 27220150 | Sausage and rice with mushroom s | Lo | Mixed Dishes - Meat, Poultry, Seafood |
| 27220170 | Sausage and rice with cheese sauc | Lo | Mixed Dishes - Meat, Poultry, Seafood |
| 27220190 | Sausage and noodles with cream o | Lo | Mixed Dishes - Meat, Poultry, Seafood |
| 27220210 | Ham and noodles, no sauce | Lo | Mixed Dishes - Meat, Poultry, Seafood |
| 27220310 | Ham or pork and rice, no sauce | Lo | Mixed Dishes - Meat, Poultry, Seafood |
| 27220510 | Ham or pork and potatoes with grav | Lo | Mixed Dishes - Meat, Poultry, Seafood |
| 27220520 | Ham or pork and potatoes with che | Lo | Mixed Dishes - Meat, Poultry, Seafood |
| 27221100 | Stewed pig's feet, Puerto Rican styl | Lo | Mixed Dishes - Meat, Poultry, Seafood |
| 27221150 | Pork stew, with potatoes, tomato-b | Lo | Mixed Dishes - Meat, Poultry, Seafood |
| 27230010 | Lamb or mutton loaf | Lo | Mixed Dishes - Meat, Poultry, Seafood |
| 27231000 | Lamb or mutton and potatoes with | Lo | Mixed Dishes - Meat, Poultry, Seafood |
| 27232000 | Lamb or mutton and potatoes with t | Lo | Mixed Dishes - Meat, Poultry, Seafood |

| 27235000 | Meat loaf made with venison/deer | Lo | Mixed Dishes - Meat, Poultry, Seafood |
| --- | --- | --- | --- |
| 27236000 | Venison or deer and noodles with c | Lo | Mixed Dishes - Meat, Poultry, Seafood |
| 27241010 | Chicken or turkey and potatoes wit | Lo | Mixed Dishes - Meat, Poultry, Seafood |
| 27242000 | Chicken or turkey and noodles, no | Lo | Mixed Dishes - Meat, Poultry, Seafood |
| 27242200 | Chicken or turkey and noodles with | Lo | Mixed Dishes - Meat, Poultry, Seafood |
| 27242250 | Chicken or turkey and noodles with | Lo | Mixed Dishes - Meat, Poultry, Seafood |
| 27242300 | Chicken or turkey and noodles with | Lo | Mixed Dishes - Meat, Poultry, Seafood |
| 27242310 | Chicken or turkey and noodles with | Lo | Mixed Dishes - Meat, Poultry, Seafood |
| 27242350 | Chicken or turkey tetrazzini | Lo | Mixed Dishes - Meat, Poultry, Seafood |
| 27242400 | Chicken or turkey and noodles with | Lo | Mixed Dishes - Meat, Poultry, Seafood |
| 27242500 | Chicken or turkey and noodles with | Lo | Mixed Dishes - Asian |
| 27243000 | Chicken or turkey and rice, no sauc | Lo | Mixed Dishes - Meat, Poultry, Seafood |
| 27243100 | Biryani with chicken | Lo | Mixed Dishes - Grain-based |
| 27243300 | Chicken or turkey and rice with cre | Lo | Mixed Dishes - Meat, Poultry, Seafood |
| 27243400 | Chicken or turkey and rice with mus | Lo | Mixed Dishes - Meat, Poultry, Seafood |
| 27243500 | Chicken or turkey and rice with tom | Lo | Mixed Dishes - Meat, Poultry, Seafood |
| 27243600 | Chicken or turkey and rice with soy | Lo | Mixed Dishes - Asian |
| 27246100 | Chicken or turkey with dumplings | Lo | Mixed Dishes - Meat, Poultry, Seafood |
| 27246200 | Chicken or turkey with stuffing | Lo | Mixed Dishes - Meat, Poultry, Seafood |
| 27246300 | Chicken or turkey cake, patty, or cr | Lo | Mixed Dishes - Meat, Poultry, Seafood |
| 27246400 | Chicken or turkey souffle | Lo | Mixed Dishes - Meat, Poultry, Seafood |
| 27246500 | Meat loaf made with chicken or turk | Lo | Mixed Dishes - Meat, Poultry, Seafood |
| 27246505 | Meat loaf made with chicken or turk | Lo | Mixed Dishes - Meat, Poultry, Seafood |
| 27250020 | Clams, stuffed | Lo | Mixed Dishes - Meat, Poultry, Seafood |
| 27250030 | Codfish ball or cake | Lo | Mixed Dishes - Meat, Poultry, Seafood |
| 27250040 | Crab cake | Lo | Mixed Dishes - Meat, Poultry, Seafood |
| 27250050 | Fish cake or patty, NS as to fish | Lo | Mixed Dishes - Meat, Poultry, Seafood |
| 27250060 | Gefilte fish | Lo | Mixed Dishes - Meat, Poultry, Seafood |
| 27250070 | Salmon cake or patty | Lo | Mixed Dishes - Meat, Poultry, Seafood |
| 27250080 | Salmon loaf | Lo | Mixed Dishes - Meat, Poultry, Seafood |
| 27250110 | Scallops and noodles with cheese s | Lo | Mixed Dishes - Meat, Poultry, Seafood |
| 27250120 | Shrimp and noodles, no sauce | Lo | Mixed Dishes - Meat, Poultry, Seafood |
| 27250122 | Shrimp and noodles with gravy | Lo | Mixed Dishes - Meat, Poultry, Seafood |
| 27250124 | Shrimp and noodles with mushroom | Lo | Mixed Dishes - Meat, Poultry, Seafood |
| 27250126 | Shrimp and noodles with cream or | Lo | Mixed Dishes - Meat, Poultry, Seafood |
| 27250128 | Shrimp and noodles with soy-based | Lo | Mixed Dishes - Asian |
| 27250130 | Shrimp and noodles with cheese sa | Lo | Mixed Dishes - Meat, Poultry, Seafood |
| 27250132 | Shrimp and noodles with tomato sa | Lo | Mixed Dishes - Meat, Poultry, Seafood |
| 27250150 | Tuna loaf | Lo | Mixed Dishes - Meat, Poultry, Seafood |
| 27250160 | Tuna cake or patty | Lo | Mixed Dishes - Meat, Poultry, Seafood |
| 27250210 | Clam cake or patty | Lo | Mixed Dishes - Meat, Poultry, Seafood |
| 27250220 | Oyster fritter | Lo | Mixed Dishes - Meat, Poultry, Seafood |
| 27250250 | Flounder with crab stuffing | Lo | Mixed Dishes - Meat, Poultry, Seafood |
| 27250260 | Lobster with bread stuffing, baked | Lo | Mixed Dishes - Meat, Poultry, Seafood |
| 27250300 | Mackerel cake or patty | Lo | Mixed Dishes - Meat, Poultry, Seafood |
| 27250400 | Shrimp cake or patty | Lo | Mixed Dishes - Meat, Poultry, Seafood |
| 27250410 | Shrimp with crab stuffing | Lo | Mixed Dishes - Meat, Poultry, Seafood |
| 27250450 | Shrimp toast, fried | Lo | Mixed Dishes - Meat, Poultry, Seafood |
| 27250510 | Fish cake (Kamaboko) tempura | Lo | Mixed Dishes - Meat, Poultry, Seafood |
| 27250520 | Seafood restructured | Lo | Seafood |
| 27250610 | Tuna noodle casserole with cream | Lo | Mixed Dishes - Meat, Poultry, Seafood |

| 27250630 | Tuna noodle casserole with mushro | Lo | Mixed Dishes - Meat, Poultry, Seafood |
| --- | --- | --- | --- |
| 27250710 | Tuna and rice with mushroom sauc | Lo | Mixed Dishes - Meat, Poultry, Seafood |
| 27250810 | Fish and rice with tomato-based sa | Lo | Mixed Dishes - Meat, Poultry, Seafood |
| 27250820 | Fish and rice with cream sauce | Lo | Mixed Dishes - Meat, Poultry, Seafood |
| 27250830 | Fish and rice with mushroom sauce | Lo | Mixed Dishes - Meat, Poultry, Seafood |
| 27250900 | Fish and noodles with mushroom s | Lo | Mixed Dishes - Meat, Poultry, Seafood |
| 27250950 | Shellfish and noodles with tomato-b | Lo | Mixed Dishes - Meat, Poultry, Seafood |
| 27260010 | Meat loaf, NS as to type of meat | Lo | Mixed Dishes - Meat, Poultry, Seafood |
| 27260050 | Meatballs, with breading, NS as to t | Lo | Mixed Dishes - Meat, Poultry, Seafood |
| 27260080 | Meat loaf made with beef and pork | Lo | Mixed Dishes - Meat, Poultry, Seafood |
| 27260090 | Meat loaf made with beef, veal and | Lo | Mixed Dishes - Meat, Poultry, Seafood |
| 27260100 | Meat loaf made with beef and pork, | Lo | Mixed Dishes - Meat, Poultry, Seafood |
| 27260110 | Hash, NS as to type of meat | Lo | Mixed Dishes - Meat, Poultry, Seafood |
| 27260500 | Vienna sausages stewed with potat | Lo | Mixed Dishes - Meat, Poultry, Seafood |
| 27260510 | Liver dumpling | Lo | Mixed Dishes - Meat, Poultry, Seafood |
| 27261500 | Stewed, seasoned, ground beef an | Lo | Mixed Dishes - Meat, Poultry, Seafood |
| 27311110 | Beef, potatoes, and vegetables incl | Lo | Mixed Dishes - Meat, Poultry, Seafood |
| 27311120 | Beef, potatoes, and vegetables, ex | Lo | Mixed Dishes - Meat, Poultry, Seafood |
| 27311210 | Corned beef, potatoes, and vegeta | Lo | Mixed Dishes - Meat, Poultry, Seafood |
| 27311220 | Corned beef, potatoes, and vegeta | Lo | Mixed Dishes - Meat, Poultry, Seafood |
| 27311310 | Beef stew with potatoes and vegeta | Lo | Mixed Dishes - Meat, Poultry, Seafood |
| 27311320 | Beef stew with potatoes and vegeta | Lo | Mixed Dishes - Meat, Poultry, Seafood |
| 27311410 | Beef stew with potatoes and vegeta | Lo | Mixed Dishes - Meat, Poultry, Seafood |
| 27311420 | Beef stew with potatoes and vegeta | Lo | Mixed Dishes - Meat, Poultry, Seafood |
| 27311510 | Shepherd's pie with beef | Lo | Mixed Dishes - Meat, Poultry, Seafood |
| 27311600 | Beef, potatoes, and vegetables incl | Lo | Mixed Dishes - Meat, Poultry, Seafood |
| 27311605 | Beef, potatoes, and vegetables exc | Lo | Mixed Dishes - Meat, Poultry, Seafood |
| 27311610 | Beef, potatoes, and vegetables incl | Lo | Mixed Dishes - Meat, Poultry, Seafood |
| 27311620 | Beef, potatoes, and vegetables exc | Lo | Mixed Dishes - Meat, Poultry, Seafood |
| 27311625 | Beef, potatoes, and vegetables incl | Lo | Mixed Dishes - Meat, Poultry, Seafood |
| 27311630 | Beef, potatoes, and vegetables exc | Lo | Mixed Dishes - Meat, Poultry, Seafood |
| 27311635 | Beef, potatoes, and vegetables incl | Lo | Mixed Dishes - Meat, Poultry, Seafood |
| 27311640 | Beef, potatoes, and vegetables exc | Lo | Mixed Dishes - Meat, Poultry, Seafood |
| 27311645 | Beef, potatoes, and vegetables incl | Lo | Mixed Dishes - Asian |
| 27311650 | Beef, potatoes, and vegetables exc | Lo | Mixed Dishes - Asian |
| 27313010 | Beef, noodles, and vegetables inclu | Lo | Mixed Dishes - Meat, Poultry, Seafood |
| 27313020 | Beef, noodles, and vegetables excl | Lo | Mixed Dishes - Meat, Poultry, Seafood |
| 27313110 | Beef chow mein or chop suey with | Lo | Mixed Dishes - Asian |
| 27313150 | Beef, noodles, and vegetables inclu | Lo | Mixed Dishes - Asian |
| 27313160 | Beef, noodles, and vegetables excl | Lo | Mixed Dishes - Asian |
| 27313210 | Beef, noodles, and vegetables inclu | Lo | Mixed Dishes - Meat, Poultry, Seafood |
| 27313220 | Beef, noodles, and vegetables excl | Lo | Mixed Dishes - Meat, Poultry, Seafood |
| 27313310 | Beef, noodles, and vegetables inclu | Lo | Mixed Dishes - Meat, Poultry, Seafood |
| 27313320 | Beef, noodles, and vegetables excl | Lo | Mixed Dishes - Meat, Poultry, Seafood |
| 27313410 | Beef, noodles, and vegetables inclu | Lo | Mixed Dishes - Meat, Poultry, Seafood |
| 27313420 | Beef, noodles, and vegetables excl | Lo | Mixed Dishes - Meat, Poultry, Seafood |
| 27315010 | Beef, rice, and vegetables including | Lo | Mixed Dishes - Meat, Poultry, Seafood |
| 27315020 | Beef, rice, and vegetables excludin | Lo | Mixed Dishes - Meat, Poultry, Seafood |
| 27315210 | Beef, rice, and vegetables including | Lo | Mixed Dishes - Meat, Poultry, Seafood |
| 27315220 | Beef, rice, and vegetables excludin | Lo | Mixed Dishes - Meat, Poultry, Seafood |
| 27315250 | Stuffed cabbage rolls with beef and | Lo | Mixed Dishes - Meat, Poultry, Seafood |

| 27315270 | Stuffed grape leaves with beef and | Lo | Mixed Dishes - Meat, Poultry, Seafood |
| --- | --- | --- | --- |
| 27315310 | Beef, rice, and vegetables including | Lo | Mixed Dishes - Meat, Poultry, Seafood |
| 27315320 | Beef, rice, and vegetables excludin | Lo | Mixed Dishes - Meat, Poultry, Seafood |
| 27315340 | Beef, rice, and vegetables excludin | Lo | Mixed Dishes - Meat, Poultry, Seafood |
| 27315410 | Beef, rice, and vegetables including | Lo | Mixed Dishes - Meat, Poultry, Seafood |
| 27315420 | Beef, rice, and vegetables excludin | Lo | Mixed Dishes - Meat, Poultry, Seafood |
| 27315510 | Beef, rice, and vegetables including | Lo | Mixed Dishes - Asian |
| 27315520 | Beef, rice, and vegetables excludin | Lo | Mixed Dishes - Asian |
| 27317010 | Beef pot pie | Lo | Mixed Dishes - Meat, Poultry, Seafood |
| 27319010 | Stuffed green pepper, Puerto Rican | Lo | Mixed Dishes - Meat, Poultry, Seafood |
| 27320020 | Ham pot pie | Lo | Mixed Dishes - Meat, Poultry, Seafood |
| 27320025 | Ham or pork, noodles and vegetabl | Lo | Mixed Dishes - Meat, Poultry, Seafood |
| 27320027 | Ham or pork, noodles, and vegetab | Lo | Mixed Dishes - Meat, Poultry, Seafood |
| 27320030 | Ham or pork, noodles and vegetabl | Lo | Mixed Dishes - Meat, Poultry, Seafood |
| 27320040 | Pork, potatoes, and vegetables incl | Lo | Mixed Dishes - Meat, Poultry, Seafood |
| 27320070 | Ham or pork, noodles, and vegetab | Lo | Mixed Dishes - Meat, Poultry, Seafood |
| 27320080 | Sausage, noodles, and vegetables | Lo | Mixed Dishes - Meat, Poultry, Seafood |
| 27320090 | Sausage, noodles, and vegetables | Lo | Mixed Dishes - Meat, Poultry, Seafood |
| 27320100 | Pork, potatoes, and vegetables incl | Lo | Mixed Dishes - Meat, Poultry, Seafood |
| 27320110 | Pork, potatoes, and vegetables exc | Lo | Mixed Dishes - Meat, Poultry, Seafood |
| 27320120 | Sausage, potatoes, and vegetables | Lo | Mixed Dishes - Meat, Poultry, Seafood |
| 27320130 | Sausage, potatoes, and vegetables | Lo | Mixed Dishes - Meat, Poultry, Seafood |
| 27320140 | Pork, potatoes, and vegetables incl | Lo | Mixed Dishes - Meat, Poultry, Seafood |
| 27320150 | Pork, potatoes, and vegetables exc | Lo | Mixed Dishes - Meat, Poultry, Seafood |
| 27320210 | Pork, potatoes, and vegetables exc | Lo | Mixed Dishes - Meat, Poultry, Seafood |
| 27320310 | Pork chow mein or chop suey with | Lo | Mixed Dishes - Asian |
| 27320320 | Pork, rice, and vegetables including | Lo | Mixed Dishes - Asian |
| 27320330 | Pork, rice, and vegetables excludin | Lo | Mixed Dishes - Asian |
| 27320340 | Pork, rice, and vegetables including | Lo | Mixed Dishes - Meat, Poultry, Seafood |
| 27320350 | Pork, rice, and vegetables excludin | Lo | Mixed Dishes - Meat, Poultry, Seafood |
| 27320410 | Ham, potatoes, and vegetables exc | Lo | Mixed Dishes - Meat, Poultry, Seafood |
| 27320450 | Ham, potatoes, and vegetables incl | Lo | Mixed Dishes - Meat, Poultry, Seafood |
| 27320500 | Sweet and sour pork with rice | Lo | Mixed Dishes - Asian |
| 27330010 | Shepherd's pie with lamb | Lo | Mixed Dishes - Meat, Poultry, Seafood |
| 27330030 | Lamb or mutton stew with potatoes | Lo | Mixed Dishes - Meat, Poultry, Seafood |
| 27330050 | Lamb or mutton, rice, and vegetabl | Lo | Mixed Dishes - Meat, Poultry, Seafood |
| 27330060 | Lamb or mutton, rice, and vegetabl | Lo | Mixed Dishes - Meat, Poultry, Seafood |
| 27330080 | Lamb or mutton, rice, and vegetabl | Lo | Mixed Dishes - Meat, Poultry, Seafood |
| 27330110 | Lamb or mutton stew with potatoes | Lo | Mixed Dishes - Meat, Poultry, Seafood |
| 27330170 | Stuffed grape leaves with lamb and | Lo | Mixed Dishes - Meat, Poultry, Seafood |
| 27330210 | Lamb or mutton stew with potatoes | Lo | Mixed Dishes - Meat, Poultry, Seafood |
| 27330220 | Lamb or mutton stew with potatoes | Lo | Mixed Dishes - Meat, Poultry, Seafood |
| 27332100 | Veal stew with potatoes and vegeta | Lo | Mixed Dishes - Meat, Poultry, Seafood |
| 27335100 | Rabbit stew with potatoes and vege | Lo | Mixed Dishes - Meat, Poultry, Seafood |
| 27336100 | Venison or deer stew with potatoes | Lo | Mixed Dishes - Meat, Poultry, Seafood |
| 27336150 | Venison or deer stew with potatoes | Lo | Mixed Dishes - Meat, Poultry, Seafood |
| 27336200 | Venison or deer, potatoes, and veg | Lo | Mixed Dishes - Meat, Poultry, Seafood |
| 27336250 | Venison or deer, potatoes, and veg | Lo | Mixed Dishes - Meat, Poultry, Seafood |
| 27336310 | Venison or deer, noodles, and vege | Lo | Mixed Dishes - Meat, Poultry, Seafood |
| 27341000 | Chicken or turkey, potatoes, corn, a | Lo | Mixed Dishes - Meat, Poultry, Seafood |
| 27341010 | Chicken or turkey, potatoes, and ve | Lo | Mixed Dishes - Meat, Poultry, Seafood |

| 27341020 | Chicken or turkey, potatoes, and ve | Lo | Mixed Dishes - Meat, Poultry, Seafood |
| --- | --- | --- | --- |
| 27341025 | Chicken or turkey, potatoes, and ve | Lo | Mixed Dishes - Meat, Poultry, Seafood |
| 27341030 | Chicken or turkey, potatoes, and ve | Lo | Mixed Dishes - Meat, Poultry, Seafood |
| 27341035 | Chicken or turkey, potatoes, and ve | Lo | Mixed Dishes - Meat, Poultry, Seafood |
| 27341040 | Chicken or turkey, potatoes, and ve | Lo | Mixed Dishes - Meat, Poultry, Seafood |
| 27341045 | Chicken or turkey, potatoes, and ve | Lo | Mixed Dishes - Meat, Poultry, Seafood |
| 27341050 | Chicken or turkey, potatoes, and ve | Lo | Mixed Dishes - Meat, Poultry, Seafood |
| 27341055 | Chicken or turkey, potatoes, and ve | Lo | Mixed Dishes - Meat, Poultry, Seafood |
| 27341060 | Chicken or turkey, potatoes, and ve | Lo | Mixed Dishes - Meat, Poultry, Seafood |
| 27341310 | Chicken or turkey stew with potatoe | Lo | Mixed Dishes - Meat, Poultry, Seafood |
| 27341320 | Chicken or turkey stew with potatoe | Lo | Mixed Dishes - Meat, Poultry, Seafood |
| 27341510 | Chicken or turkey stew with potatoe | Lo | Mixed Dishes - Meat, Poultry, Seafood |
| 27341520 | Chicken or turkey stew with potatoe | Lo | Mixed Dishes - Meat, Poultry, Seafood |
| 27343010 | Chicken or turkey, noodles, and ve | Lo | Mixed Dishes - Meat, Poultry, Seafood |
| 27343020 | Chicken or turkey, noodles, and ve | Lo | Mixed Dishes - Meat, Poultry, Seafood |
| 27343410 | Chicken or turkey, noodles, and ve | Lo | Mixed Dishes - Meat, Poultry, Seafood |
| 27343420 | Chicken or turkey, noodles, and ve | Lo | Mixed Dishes - Meat, Poultry, Seafood |
| 27343470 | Chicken or turkey, noodles, and ve | Lo | Mixed Dishes - Meat, Poultry, Seafood |
| 27343480 | Chicken or turkey, noodles, and ve | Lo | Mixed Dishes - Meat, Poultry, Seafood |
| 27343510 | Chicken or turkey, noodles, and ve | Lo | Mixed Dishes - Meat, Poultry, Seafood |
| 27343520 | Chicken or turkey, noodles, and ve | Lo | Mixed Dishes - Meat, Poultry, Seafood |
| 27343910 | Chicken or turkey chow mein or ch | Lo | Mixed Dishes - Asian |
| 27343950 | Chicken or turkey, noodles, and ve | Lo | Mixed Dishes - Meat, Poultry, Seafood |
| 27343960 | Chicken or turkey, noodles, and ve | Lo | Mixed Dishes - Meat, Poultry, Seafood |
| 27345010 | Chicken or turkey, rice, and vegeta | Lo | Mixed Dishes - Meat, Poultry, Seafood |
| 27345020 | Chicken or turkey, rice, and vegeta | Lo | Mixed Dishes - Meat, Poultry, Seafood |
| 27345210 | Chicken or turkey, rice, and vegeta | Lo | Mixed Dishes - Meat, Poultry, Seafood |
| 27345220 | Chicken or turkey, rice, and vegeta | Lo | Mixed Dishes - Meat, Poultry, Seafood |
| 27345230 | Chicken or turkey, rice, corn, and c | Lo | Mixed Dishes - Meat, Poultry, Seafood |
| 27345310 | Chicken or turkey, rice, and vegeta | Lo | Mixed Dishes - Asian |
| 27345320 | Chicken or turkey, rice, and vegeta | Lo | Mixed Dishes - Asian |
| 27345410 | Chicken or turkey, rice, and vegeta | Lo | Mixed Dishes - Meat, Poultry, Seafood |
| 27345420 | Chicken or turkey, rice, and vegeta | Lo | Mixed Dishes - Meat, Poultry, Seafood |
| 27345440 | Chicken or turkey, rice, and vegeta | Lo | Mixed Dishes - Meat, Poultry, Seafood |
| 27345450 | Chicken or turkey, rice, and vegeta | Lo | Mixed Dishes - Meat, Poultry, Seafood |
| 27345510 | Chicken or turkey, rice, and vegeta | Lo | Mixed Dishes - Meat, Poultry, Seafood |
| 27345520 | Chicken or turkey, rice, and vegeta | Lo | Mixed Dishes - Meat, Poultry, Seafood |
| 27347100 | Chicken or turkey pot pie | Lo | Mixed Dishes - Meat, Poultry, Seafood |
| 27347200 | Chicken or turkey, stuffing, and veg | Lo | Mixed Dishes - Meat, Poultry, Seafood |
| 27347210 | Chicken or turkey,stuffing, and veg | Lo | Mixed Dishes - Meat, Poultry, Seafood |
| 27347220 | Chicken or turkey, stuffing, and veg | Lo | Mixed Dishes - Meat, Poultry, Seafood |
| 27347230 | Chicken or turkey, stuffing, and veg | Lo | Mixed Dishes - Meat, Poultry, Seafood |
| 27347240 | Chicken or turkey, dumplings, and | Lo | Mixed Dishes - Meat, Poultry, Seafood |
| 27347250 | Chicken or turkey, dumplings, and | Lo | Mixed Dishes - Meat, Poultry, Seafood |
| 27348100 | Chicken fricassee, Puerto Rican sty | Lo | Mixed Dishes - Meat, Poultry, Seafood |
| 27350020 | Paella with seafood | Lo | Mixed Dishes - Grain-based |
| 27350030 | Seafood stew with potatoes and ve | Lo | Mixed Dishes - Meat, Poultry, Seafood |
| 27350050 | Shrimp chow mein or chop suey wit | Lo | Mixed Dishes - Asian |
| 27350060 | Shrimp creole, with rice | Lo | Mixed Dishes - Meat, Poultry, Seafood |
| 27350070 | Tuna pot pie | Lo | Mixed Dishes - Meat, Poultry, Seafood |
| 27350080 | Tuna noodle casserole with vegeta | Lo | Mixed Dishes - Meat, Poultry, Seafood |

| 27350090 | Fish, noodles, and vegetables inclu | Lo | Mixed Dishes - Meat, Poultry, Seafood |
| --- | --- | --- | --- |
| 27350100 | Fish, noodles, and vegetables exclu | Lo | Mixed Dishes - Meat, Poultry, Seafood |
| 27350110 | Bouillabaisse | Lo | Mixed Dishes - Meat, Poultry, Seafood |
| 27350310 | Seafood stew with potatoes and ve | Lo | Mixed Dishes - Meat, Poultry, Seafood |
| 27350410 | Tuna noodle casserole with vegeta | Lo | Mixed Dishes - Meat, Poultry, Seafood |
| 27351030 | Stewed codfish, Puerto Rican style | Lo | Mixed Dishes - Meat, Poultry, Seafood |
| 27351040 | Biscayne codfish, Puerto Rican styl | Lo | Mixed Dishes - Meat, Poultry, Seafood |
| 27360000 | Stew, NFS | Lo | Mixed Dishes - Meat, Poultry, Seafood |
| 27360010 | Goulash, NFS | Lo | Mixed Dishes - Meat, Poultry, Seafood |
| 27360050 | Meat pie, NFS | Lo | Mixed Dishes - Meat, Poultry, Seafood |
| 27360080 | Chow mein or chop suey, NS as to | Lo | Mixed Dishes - Asian |
| 27360090 | Paella, NFS | Lo | Mixed Dishes - Grain-based |
| 27360100 | Brunswick stew | Lo | Mixed Dishes - Meat, Poultry, Seafood |
| 27360120 | Chow mein or chop suey, various ty | Lo | Mixed Dishes - Asian |
| 27361010 | Stewed variety meats, mostly liver, | Lo | Mixed Dishes - Meat, Poultry, Seafood |
| 27362000 | Stewed tripe, with potatoes, Puerto | Lo | Mixed Dishes - Meat, Poultry, Seafood |
| 27363000 | Gumbo with rice | Lo | Mixed Dishes - Meat, Poultry, Seafood |
| 27363100 | Jambalaya with meat and rice | Lo | Mixed Dishes - Grain-based |
| 27410210 | Beef and vegetables including carro | Lo | Mixed Dishes - Meat, Poultry, Seafood |
| 27410220 | Beef and vegetables excluding carr | Lo | Mixed Dishes - Meat, Poultry, Seafood |
| 27410250 | Beef shish kabob with vegetables, | Lo | Mixed Dishes - Meat, Poultry, Seafood |
| 27411100 | Beef with vegetables including carr | Lo | Mixed Dishes - Meat, Poultry, Seafood |
| 27411120 | Swiss steak | Lo | Mixed Dishes - Meat, Poultry, Seafood |
| 27411150 | Beef rolls, stuffed with vegetables o | Lo | Mixed Dishes - Meat, Poultry, Seafood |
| 27411200 | Beef with vegetables excluding carr | Lo | Mixed Dishes - Meat, Poultry, Seafood |
| 27414100 | Beef with vegetables including carr | Lo | Mixed Dishes - Meat, Poultry, Seafood |
| 27414200 | Beef with vegetables excluding carr | Lo | Mixed Dishes - Meat, Poultry, Seafood |
| 27415100 | Beef and vegetables including carro | Lo | Mixed Dishes - Asian |
| 27415110 | Beef and broccoli | Lo | Mixed Dishes - Asian |
| 27415120 | Beef, tofu, and vegetables including | Lo | Mixed Dishes - Asian |
| 27415140 | Hunan beef | Lo | Mixed Dishes - Asian |
| 27415150 | Beef chow mein or chop suey, no n | Lo | Mixed Dishes - Asian |
| 27415170 | Kung Pao beef | Lo | Mixed Dishes - Asian |
| 27415200 | Beef and vegetables excluding carr | Lo | Mixed Dishes - Asian |
| 27415220 | Beef, tofu, and vegetables excludin | Lo | Mixed Dishes - Asian |
| 27416150 | Pepper steak | Lo | Mixed Dishes - Meat, Poultry, Seafood |
| 27416200 | Beef, ground, with egg and onion | Lo | Mixed Dishes - Meat, Poultry, Seafood |
| 27416250 | Beef salad | Lo | Mixed Dishes - Meat, Poultry, Seafood |
| 27416300 | Beef taco filling: beef, cheese, toma | Lo | Mixed Dishes - Mexican |
| 27416400 | Stir fried beef and vegetables in so | Lo | Mixed Dishes - Asian |
| 27416450 | Beef and vegetables including carro | Lo | Mixed Dishes - Meat, Poultry, Seafood |
| 27416500 | Beef and vegetables excluding carr | Lo | Mixed Dishes - Meat, Poultry, Seafood |
| 27418110 | Seasoned shredded soup meat | Lo | Mixed Dishes - Meat, Poultry, Seafood |
| 27418210 | Beef stew with vegetables excludin | Lo | Mixed Dishes - Meat, Poultry, Seafood |
| 27418310 | Corned beef with tomato sauce and | Lo | Mixed Dishes - Meat, Poultry, Seafood |
| 27418410 | Beef steak with onions, Puerto Rica | Lo | Mixed Dishes - Meat, Poultry, Seafood |
| 27420010 | Cabbage with ham hocks | Lo | Mixed Dishes - Meat, Poultry, Seafood |
| 27420020 | Ham or pork salad | Lo | Mixed Dishes - Meat, Poultry, Seafood |
| 27420040 | Frankfurters or hot dogs and sauer | Lo | Mixed Dishes - Meat, Poultry, Seafood |
| 27420060 | Pork and vegetables including carro | Lo | Mixed Dishes - Meat, Poultry, Seafood |
| 27420080 | Greens with ham or pork | Lo | Vegetables, excluding Potatoes |

| 27420100 | Pork, tofu, and vegetables including | Lo | Mixed Dishes - Asian |
| --- | --- | --- | --- |
| 27420110 | Pork and vegetables, Hawaiian styl | Lo | Mixed Dishes - Asian |
| 27420150 | Kung Pao pork | Lo | Mixed Dishes - Asian |
| 27420160 | Moo Shu pork, without Chinese pan | Lo | Mixed Dishes - Asian |
| 27420170 | Pork and onions with soy-based sa | Lo | Mixed Dishes - Asian |
| 27420200 | Pork hash | Lo | Mixed Dishes - Meat, Poultry, Seafood |
| 27420250 | Ham and vegetables including carr | Lo | Mixed Dishes - Meat, Poultry, Seafood |
| 27420270 | Ham and vegetables excluding carr | Lo | Mixed Dishes - Meat, Poultry, Seafood |
| 27420350 | Pork and vegetables excluding carr | Lo | Mixed Dishes - Meat, Poultry, Seafood |
| 27420370 | Pork, tofu, and vegetables, excludin | Lo | Mixed Dishes - Asian |
| 27420390 | Pork chow mein or chop suey, no n | Lo | Mixed Dishes - Asian |
| 27420400 | Pork and vegetables including carro | Lo | Mixed Dishes - Meat, Poultry, Seafood |
| 27420410 | Pork and vegetables excluding car | Lo | Mixed Dishes - Meat, Poultry, Seafood |
| 27420450 | Sausage and vegetables including | Lo | Mixed Dishes - Meat, Poultry, Seafood |
| 27420460 | Sausage and vegetables, excluding | Lo | Mixed Dishes - Meat, Poultry, Seafood |
| 27420470 | Sausage and peppers, no sauce | Lo | Mixed Dishes - Meat, Poultry, Seafood |
| 27420500 | Pork and vegetables including carro | Lo | Mixed Dishes - Asian |
| 27420510 | Pork and vegetables excluding carr | Lo | Mixed Dishes - Asian |
| 27420520 | Pork shish kabob with vegetables, | Lo | Mixed Dishes - Meat, Poultry, Seafood |
| 27421010 | Stuffed christophine, Puerto Rican | Lo | Mixed Dishes - Meat, Poultry, Seafood |
| 27422010 | Pork chop stewed with vegetables, | Lo | Mixed Dishes - Meat, Poultry, Seafood |
| 27430400 | Lamb or mutton stew with vegetabl | Lo | Mixed Dishes - Meat, Poultry, Seafood |
| 27430410 | Lamb or mutton stew with vegetabl | Lo | Mixed Dishes - Meat, Poultry, Seafood |
| 27430500 | Veal goulash with vegetables exclu | Lo | Mixed Dishes - Meat, Poultry, Seafood |
| 27430610 | Lamb shish kabob with vegetables, | Lo | Mixed Dishes - Meat, Poultry, Seafood |
| 27440110 | Chicken or turkey and vegetables i | Lo | Mixed Dishes - Meat, Poultry, Seafood |
| 27440120 | Chicken or turkey and vegetables e | Lo | Mixed Dishes - Meat, Poultry, Seafood |
| 27440130 | Chicken or turkey shish kabob with | Lo | Mixed Dishes - Meat, Poultry, Seafood |
| 27442110 | Chicken or turkey and vegetables i | Lo | Mixed Dishes - Meat, Poultry, Seafood |
| 27442120 | Chicken or turkey and vegetables e | Lo | Mixed Dishes - Meat, Poultry, Seafood |
| 27443110 | Chicken or turkey a la king with veg | Lo | Mixed Dishes - Meat, Poultry, Seafood |
| 27443120 | Chicken or turkey a la king with veg | Lo | Mixed Dishes - Meat, Poultry, Seafood |
| 27443150 | Chicken or turkey divan | Lo | Mixed Dishes - Meat, Poultry, Seafood |
| 27445110 | Chicken or turkey and vegetables i | Lo | Mixed Dishes - Asian |
| 27445120 | Chicken or turkey and vegetables e | Lo | Mixed Dishes - Asian |
| 27445125 | Chicken or turkey and vegetables i | Lo | Mixed Dishes - Meat, Poultry, Seafood |
| 27445130 | Chicken or turkey and vegetables e | Lo | Mixed Dishes - Meat, Poultry, Seafood |
| 27445150 | General Tso chicken | Lo | Mixed Dishes - Asian |
| 27445180 | Moo Goo Gai Pan | Lo | Mixed Dishes - Asian |
| 27445220 | Kung pao chicken | Lo | Mixed Dishes - Asian |
| 27445250 | Almond chicken | Lo | Mixed Dishes - Asian |
| 27446100 | Chicken or turkey chow mein or ch | Lo | Mixed Dishes - Asian |
| 27446200 | Chicken or turkey salad, made with | Lo | Mixed Dishes - Meat, Poultry, Seafood |
| 27446205 | Chicken or turkey salad with nuts a | Lo | Mixed Dishes - Meat, Poultry, Seafood |
| 27446220 | Chicken or turkey salad with egg | Lo | Mixed Dishes - Meat, Poultry, Seafood |
| 27446225 | Chicken or turkey salad, made with | Lo | Mixed Dishes - Meat, Poultry, Seafood |
| 27446230 | Chicken or turkey salad, made with | Lo | Mixed Dishes - Meat, Poultry, Seafood |
| 27446235 | Chicken or turkey salad, made with | Lo | Mixed Dishes - Meat, Poultry, Seafood |
| 27446240 | Chicken or turkey salad, made with | Lo | Mixed Dishes - Meat, Poultry, Seafood |
| 27446245 | Chicken or turkey salad, made with | Lo | Mixed Dishes - Meat, Poultry, Seafood |
| 27446260 | Chicken or turkey salad, made with | Lo | Mixed Dishes - Meat, Poultry, Seafood |

| 27446400 | Chicken or turkey and vegetables i | Lo | Mixed Dishes - Meat, Poultry, Seafood |
| --- | --- | --- | --- |
| 27446410 | Chicken or turkey and vegetables e | Lo | Mixed Dishes - Meat, Poultry, Seafood |
| 27448020 | Chicken or turkey fricassee, with sa | Lo | Mixed Dishes - Meat, Poultry, Seafood |
| 27450010 | Crab salad | Lo | Mixed Dishes - Meat, Poultry, Seafood |
| 27450020 | Lobster salad | Lo | Mixed Dishes - Meat, Poultry, Seafood |
| 27450040 | Shrimp chow mein or chop suey, no | Lo | Mixed Dishes - Asian |
| 27450060 | Tuna salad, made with mayonnaise | Lo | Mixed Dishes - Meat, Poultry, Seafood |
| 27450061 | Tuna salad, made with light mayon | Lo | Mixed Dishes - Meat, Poultry, Seafood |
| 27450062 | Tuna salad, made with mayonnaise | Lo | Mixed Dishes - Meat, Poultry, Seafood |
| 27450063 | Tuna salad, made with light mayon | Lo | Mixed Dishes - Meat, Poultry, Seafood |
| 27450064 | Tuna salad, made with creamy dres | Lo | Mixed Dishes - Meat, Poultry, Seafood |
| 27450065 | Tuna salad, made with light creamy | Lo | Mixed Dishes - Meat, Poultry, Seafood |
| 27450066 | Tuna salad, made with Italian dress | Lo | Mixed Dishes - Meat, Poultry, Seafood |
| 27450067 | Tuna salad, made with light Italian | Lo | Mixed Dishes - Meat, Poultry, Seafood |
| 27450068 | Tuna salad, made with any type of f | Lo | Mixed Dishes - Meat, Poultry, Seafood |
| 27450070 | Shrimp salad | Lo | Mixed Dishes - Meat, Poultry, Seafood |
| 27450080 | Seafood salad | Lo | Mixed Dishes - Meat, Poultry, Seafood |
| 27450130 | Crab salad made with imitation cra | Lo | Mixed Dishes - Meat, Poultry, Seafood |
| 27450150 | Fish, tofu, and vegetables, tempura | Lo | Mixed Dishes - Meat, Poultry, Seafood |
| 27450250 | Oysters Rockefeller | Lo | Mixed Dishes - Meat, Poultry, Seafood |
| 27450400 | Shrimp and vegetables including ca | Lo | Mixed Dishes - Meat, Poultry, Seafood |
| 27450405 | Shrimp and vegetables excluding c | Lo | Mixed Dishes - Meat, Poultry, Seafood |
| 27450410 | Shrimp and vegetables including ca | Lo | Mixed Dishes - Asian |
| 27450420 | Shrimp and vegetables excluding c | Lo | Mixed Dishes - Asian |
| 27450430 | Shrimp shish kabob with vegetable | Lo | Mixed Dishes - Meat, Poultry, Seafood |
| 27450450 | Shrimp creole, no rice | Lo | Mixed Dishes - Meat, Poultry, Seafood |
| 27450470 | Kung Pao shrimp | Lo | Mixed Dishes - Asian |
| 27450510 | Tuna casserole with vegetables an | Lo | Mixed Dishes - Meat, Poultry, Seafood |
| 27450600 | Shellfish mixture and vegetables in | Lo | Mixed Dishes - Asian |
| 27450610 | Shellfish mixture and vegetables ex | Lo | Mixed Dishes - Asian |
| 27450650 | Shellfish mixture and vegetables in | Lo | Mixed Dishes - Meat, Poultry, Seafood |
| 27450660 | Shellfish mixture and vegetables ex | Lo | Mixed Dishes - Meat, Poultry, Seafood |
| 27450700 | Fish and vegetables including carro | Lo | Mixed Dishes - Meat, Poultry, Seafood |
| 27450710 | Fish and vegetables excluding carr | Lo | Mixed Dishes - Meat, Poultry, Seafood |
| 27450740 | Fish and vegetables including carro | Lo | Mixed Dishes - Asian |
| 27450750 | Fish and vegetables excluding carr | Lo | Mixed Dishes - Asian |
| 27451030 | Lobster with sauce, Puerto Rican st | Lo | Mixed Dishes - Meat, Poultry, Seafood |
| 27460010 | Chow mein or chop suey, NS as to | Lo | Mixed Dishes - Asian |
| 27460510 | Antipasto with ham, fish, cheese, v | Lo | Mixed Dishes - Meat, Poultry, Seafood |
| 27460710 | Livers, chicken, chopped, with eggs | Lo | Mixed Dishes - Meat, Poultry, Seafood |
| 27460750 | Liver, beef or calves, and onions | Lo | Mixed Dishes - Meat, Poultry, Seafood |
| 27461010 | Stewed seasoned ground beef, Pue | Lo | Mixed Dishes - Meat, Poultry, Seafood |
| 27464000 | Gumbo, no rice | Lo | Mixed Dishes - Meat, Poultry, Seafood |
| 27500050 | Sandwich, NFS | Lo | Mixed Dishes - Sandwiches (single code) |
| 27500100 | Meat sandwich, NFS | Lo | Mixed Dishes - Sandwiches (single code) |
| 27500200 | Wrap sandwich, filled with meat, po | Lo | Mixed Dishes - Sandwiches (single code) |
| 27500300 | Wrap sandwich, NFS | Lo | Mixed Dishes - Sandwiches (single code) |
| 27510000 | Beef sandwich, NFS | Lo | Mixed Dishes - Sandwiches (single code) |
| 27510110 | Beef barbecue sandwich or Sloppy | Lo | Mixed Dishes - Sandwiches (single code) |
| 27510130 | Beef barbecue submarine sandwic | Lo | Mixed Dishes - Sandwiches (single code) |
| 27510140 | Cheeseburger slider, from fast food | Lo | Mixed Dishes - Sandwiches (single code) |

| 27510145 | Cheeseburger, 1 miniature patty, w | Lo | Mixed Dishes - Sandwiches (single code) |
| --- | --- | --- | --- |
| 27510150 | Cheeseburger, 1 miniature patty, o | Lo | Mixed Dishes - Sandwiches (single code) |
| 27510155 | Cheeseburger, NFS | Lo | Mixed Dishes - Sandwiches (single code) |
| 27510160 | Cheeseburger, from fast food, 1 sm | Lo | Mixed Dishes - Sandwiches (single code) |
| 27510165 | Cheeseburger, 1 small patty, with c | Lo | Mixed Dishes - Sandwiches (single code) |
| 27510170 | Cheeseburger (Burger King) | Lo | Mixed Dishes - Sandwiches (single code) |
| 27510171 | Whopper Jr with cheese (Burger Ki | Lo | Mixed Dishes - Sandwiches (single code) |
| 27510172 | Cheeseburger (McDonalds) | Lo | Mixed Dishes - Sandwiches (single code) |
| 27510173 | Cheeseburger, 1 small patty, with c | Lo | Mixed Dishes - Sandwiches (single code) |
| 27510174 | Cheeseburger, 1 small patty, with c | Lo | Mixed Dishes - Sandwiches (single code) |
| 27510175 | Cheeseburger, 1 small patty, with c | Lo | Mixed Dishes - Sandwiches (single code) |
| 27510190 | Cheeseburger, from school cafeteri | Lo | Mixed Dishes - Sandwiches (single code) |
| 27510191 | Cheeseburger slider | Lo | Mixed Dishes - Sandwiches (single code) |
| 27510195 | Cheeseburger, on white bun, 1 sma | Lo | Mixed Dishes - Sandwiches (single code) |
| 27510196 | Cheeseburger, on wheat bun, 1 sm | Lo | Mixed Dishes - Sandwiches (single code) |
| 27510205 | Cheeseburger, 1 small patty, with c | Lo | Mixed Dishes - Sandwiches (single code) |
| 27510206 | Cheeseburger, 1 small patty, with c | Lo | Mixed Dishes - Sandwiches (single code) |
| 27510207 | Cheeseburger, 1 small patty, with c | Lo | Mixed Dishes - Sandwiches (single code) |
| 27510210 | Cheeseburger, plain, on bun | Lo | Mixed Dishes - Sandwiches (single code) |
| 27510215 | Cheeseburger, from fast food, 1 me | Lo | Mixed Dishes - Sandwiches (single code) |
| 27510220 | Cheeseburger, with mayonnaise or | Lo | Mixed Dishes - Sandwiches (single code) |
| 27510225 | Cheeseburger, 1 medium patty, wit | Lo | Mixed Dishes - Sandwiches (single code) |
| 27510229 | Quarter Pounder (McDonalds) | Lo | Mixed Dishes - Sandwiches (single code) |
| 27510230 | Cheeseburger, with mayonnaise or | Lo | Mixed Dishes - Sandwiches (single code) |
| 27510231 | Whopper with cheese (Burger King | Lo | Mixed Dishes - Sandwiches (single code) |
| 27510232 | Quarter Pounder with cheese (McD | Lo | Mixed Dishes - Sandwiches (single code) |
| 27510233 | Cheeseburger, 1 medium patty, wit | Lo | Mixed Dishes - Sandwiches (single code) |
| 27510235 | Cheeseburger submarine sandwich | Lo | Mixed Dishes - Sandwiches (single code) |
| 27510240 | Cheeseburger, 1/4 lb meat, plain, o | Lo | Mixed Dishes - Sandwiches (single code) |
| 27510241 | Cheeseburger, on white bun, 1 me | Lo | Mixed Dishes - Sandwiches (single code) |
| 27510242 | Cheeseburger, on wheat bun, 1 me | Lo | Mixed Dishes - Sandwiches (single code) |
| 27510243 | Cheeseburger, 1 medium patty, pla | Lo | Mixed Dishes - Sandwiches (single code) |
| 27510245 | Cheeseburger, on white bun, 1 larg | Lo | Mixed Dishes - Sandwiches (single code) |
| 27510246 | Cheeseburger, on wheat bun, 1 lar | Lo | Mixed Dishes - Sandwiches (single code) |
| 27510250 | Cheeseburger, 1/4 lb meat, with ma | Lo | Mixed Dishes - Sandwiches (single code) |
| 27510251 | Cheeseburger, 1 medium patty, wit | Lo | Mixed Dishes - Sandwiches (single code) |
| 27510252 | Cheeseburger, 1 medium patty, wit | Lo | Mixed Dishes - Sandwiches (single code) |
| 27510253 | Cheeseburger, 1 medium patty, wit | Lo | Mixed Dishes - Sandwiches (single code) |
| 27510254 | Double cheeseburger, on white bun | Lo | Mixed Dishes - Sandwiches (single code) |
| 27510257 | Double cheeseburger, on white bun | Lo | Mixed Dishes - Sandwiches (single code) |
| 27510258 | Double cheeseburger, on wheat bu | Lo | Mixed Dishes - Sandwiches (single code) |
| 27510260 | Cheeseburger, 1/4 lb meat, with mu | Lo | Mixed Dishes - Sandwiches (single code) |
| 27510261 | Cheeseburger, from fast food, 1 lar | Lo | Mixed Dishes - Sandwiches (single code) |
| 27510262 | Double cheeseburger, on white bun | Lo | Mixed Dishes - Sandwiches (single code) |
| 27510265 | Double cheeseburger, (2 patties, 1 | Lo | Mixed Dishes - Sandwiches (single code) |
| 27510266 | Cheeseburger, 1 large patty, with c | Lo | Mixed Dishes - Sandwiches (single code) |
| 27510270 | Double cheeseburger (2 patties), pl | Lo | Mixed Dishes - Sandwiches (single code) |
| 27510276 | Bacon cheeseburger, 1 small patty, | Lo | Mixed Dishes - Sandwiches (single code) |
| 27510280 | Double cheeseburger (2 patties), w | Lo | Mixed Dishes - Sandwiches (single code) |
| 27510281 | Bacon cheeseburger, 1 small patty, | Lo | Mixed Dishes - Sandwiches (single code) |
| 27510290 | Double cheeseburger (2 patties), pl | Lo | Mixed Dishes - Sandwiches (single code) |

| 27510300 | Double cheeseburger (2 patties), w | Lo | Mixed Dishes - Sandwiches (single code) |
| --- | --- | --- | --- |
| 27510305 | Bacon cheeseburger, 1 medium pa | Lo | Mixed Dishes - Sandwiches (single code) |
| 27510310 | Cheeseburger with tomato and/or c | Lo | Mixed Dishes - Sandwiches (single code) |
| 27510311 | Cheeseburger, 1 oz meat, plain, on | Lo | Mixed Dishes - Sandwiches (single code) |
| 27510312 | Bacon cheeseburger, 1 medium pa | Lo | Mixed Dishes - Sandwiches (single code) |
| 27510320 | Cheeseburger, 1/4 lb meat, with to | Lo | Mixed Dishes - Sandwiches (single code) |
| 27510330 | Double cheeseburger (2 patties), w | Lo | Mixed Dishes - Sandwiches (single code) |
| 27510331 | Bacon cheeseburger, 1 medium pa | Lo | Mixed Dishes - Sandwiches (single code) |
| 27510340 | Double cheeseburger (2 patties), w | Lo | Mixed Dishes - Sandwiches (single code) |
| 27510341 | Bacon cheeseburger, 1 medium pa | Lo | Mixed Dishes - Sandwiches (single code) |
| 27510342 | Bacon cheeseburger, 1 medium pa | Lo | Mixed Dishes - Sandwiches (single code) |
| 27510343 | Bacon cheeseburger, 1 medium pa | Lo | Mixed Dishes - Sandwiches (single code) |
| 27510346 | Bacon cheeseburger, 1 large patty, | Lo | Mixed Dishes - Sandwiches (single code) |
| 27510350 | Cheeseburger, 1/4 lb meat, with ma | Lo | Mixed Dishes - Sandwiches (single code) |
| 27510355 | Cheeseburger, 1/3 lb meat, with ma | Lo | Mixed Dishes - Sandwiches (single code) |
| 27510359 | Cheeseburger, 1/3 lb meat, with ma | Lo | Mixed Dishes - Sandwiches (single code) |
| 27510360 | Bacon cheeseburger, with mayonn | Lo | Mixed Dishes - Sandwiches (single code) |
| 27510370 | Double cheeseburger (2 patties, 1/4 | Lo | Mixed Dishes - Sandwiches (single code) |
| 27510371 | Double cheeseburger, from fast foo | Lo | Mixed Dishes - Sandwiches (single code) |
| 27510375 | Double cheeseburger (2 patties, 1/4 | Lo | Mixed Dishes - Sandwiches (single code) |
| 27510376 | Double cheeseburger, 2 small patti | Lo | Mixed Dishes - Sandwiches (single code) |
| 27510380 | Triple cheeseburger (3 patties, 1/4 | Lo | Mixed Dishes - Sandwiches (single code) |
| 27510385 | Double bacon cheeseburger (2 patt | Lo | Mixed Dishes - Sandwiches (single code) |
| 27510386 | Double cheeseburger (Burger King | Lo | Mixed Dishes - Sandwiches (single code) |
| 27510387 | Double cheeseburger (McDonalds) | Lo | Mixed Dishes - Sandwiches (single code) |
| 27510388 | McDouble (McDonalds) | Lo | Mixed Dishes - Sandwiches (single code) |
| 27510389 | Big Mac (McDonalds) | Lo | Mixed Dishes - Sandwiches (single code) |
| 27510390 | Double bacon cheeseburger (2 patt | Lo | Mixed Dishes - Sandwiches (single code) |
| 27510391 | Double cheeseburger, 2 small patti | Lo | Mixed Dishes - Sandwiches (single code) |
| 27510400 | Bacon cheeseburger, 1/4 lb meat, | Lo | Mixed Dishes - Sandwiches (single code) |
| 27510401 | Double cheeseburger, from fast foo | Lo | Mixed Dishes - Sandwiches (single code) |
| 27510405 | Double cheeseburger, from fast foo | Lo | Mixed Dishes - Sandwiches (single code) |
| 27510406 | Double cheeseburger, 2 medium p | Lo | Mixed Dishes - Sandwiches (single code) |
| 27510410 | Chiliburger, on bun | Lo | Mixed Dishes - Sandwiches (single code) |
| 27510412 | Double cheeseburger, 2 medium p | Lo | Mixed Dishes - Sandwiches (single code) |
| 27510413 | Double cheeseburger, 2 medium p | Lo | Mixed Dishes - Sandwiches (single code) |
| 27510420 | Taco burger, on bun | Lo | Mixed Dishes - Sandwiches (single code) |
| 27510425 | Double bacon cheeseburger (2 patt | Lo | Mixed Dishes - Sandwiches (single code) |
| 27510430 | Double bacon cheeseburger (2 patt | Lo | Mixed Dishes - Sandwiches (single code) |
| 27510431 | Double bacon cheeseburger, 2 sma | Lo | Mixed Dishes - Sandwiches (single code) |
| 27510435 | Double bacon cheeseburger (2 patt | Lo | Mixed Dishes - Sandwiches (single code) |
| 27510440 | Bacon cheeseburger, 1/4 lb meat, | Lo | Mixed Dishes - Sandwiches (single code) |
| 27510445 | Bacon cheeseburger, 1/3 lb meat, | Lo | Mixed Dishes - Sandwiches (single code) |
| 27510446 | Double bacon cheeseburger, 2 me | Lo | Mixed Dishes - Sandwiches (single code) |
| 27510450 | Cheeseburger, 1/4 lb meat, with ha | Lo | Mixed Dishes - Sandwiches (single code) |
| 27510451 | Double bacon cheeseburger, 2 me | Lo | Mixed Dishes - Sandwiches (single code) |
| 27510465 | Double bacon cheeseburger, 2 me | Lo | Mixed Dishes - Sandwiches (single code) |
| 27510475 | Double bacon cheeseburger, 2 larg | Lo | Mixed Dishes - Sandwiches (single code) |
| 27510480 | Cheeseburger (hamburger with che | Lo | Mixed Dishes - Sandwiches (single code) |
| 27510486 | Triple cheeseburger, 3 medium pat | Lo | Mixed Dishes - Sandwiches (single code) |
| 27510500 | Hamburger, plain, on bun | Lo | Mixed Dishes - Sandwiches (single code) |

| 27510501 | Hamburger slider, from fast food | Lo | Mixed Dishes - Sandwiches (single code) |
| --- | --- | --- | --- |
| 27510506 | Hamburger, 1 miniature patty, with | Lo | Mixed Dishes - Sandwiches (single code) |
| 27510510 | Hamburger, with tomato and/or cat | Lo | Mixed Dishes - Sandwiches (single code) |
| 27510511 | Hamburger, 1 miniature patty, on m | Lo | Mixed Dishes - Sandwiches (single code) |
| 27510520 | Hamburger, with mayonnaise or sa | Lo | Mixed Dishes - Sandwiches (single code) |
| 27510530 | Hamburger, 1/4 lb meat, plain, on b | Lo | Mixed Dishes - Sandwiches (single code) |
| 27510531 | Hamburger, from fast food, 1 small | Lo | Mixed Dishes - Sandwiches (single code) |
| 27510536 | Hamburger, 1 small patty, with con | Lo | Mixed Dishes - Sandwiches (single code) |
| 27510540 | Double hamburger (2 patties), with | Lo | Mixed Dishes - Sandwiches (single code) |
| 27510550 | Double hamburger (2 patties), with | Lo | Mixed Dishes - Sandwiches (single code) |
| 27510551 | Hamburger (Burger King) | Lo | Mixed Dishes - Sandwiches (single code) |
| 27510552 | Whopper Jr (Burger King) | Lo | Mixed Dishes - Sandwiches (single code) |
| 27510553 | Hamburger (McDonalds) | Lo | Mixed Dishes - Sandwiches (single code) |
| 27510555 | Hamburger, 1 small patty, with con | Lo | Mixed Dishes - Sandwiches (single code) |
| 27510560 | Hamburger, 1/4 lb meat, with mayo | Lo | Mixed Dishes - Sandwiches (single code) |
| 27510565 | Hamburger, from school cafeteria | Lo | Mixed Dishes - Sandwiches (single code) |
| 27510570 | Hamburger, 2- 1/2 oz meat, with ma | Lo | Mixed Dishes - Sandwiches (single code) |
| 27510573 | Hamburger slider | Lo | Mixed Dishes - Sandwiches (single code) |
| 27510575 | Hamburger, on white bun, 1 small p | Lo | Mixed Dishes - Sandwiches (single code) |
| 27510576 | Hamburger, on wheat bun, 1 small | Lo | Mixed Dishes - Sandwiches (single code) |
| 27510577 | Hamburger, 1 small patty, plain, on | Lo | Mixed Dishes - Sandwiches (single code) |
| 27510585 | Hamburger, 1 small patty, with con | Lo | Mixed Dishes - Sandwiches (single code) |
| 27510587 | Hamburger, 1 small patty, with con | Lo | Mixed Dishes - Sandwiches (single code) |
| 27510590 | Hamburger, with mayonnaise or sa | Lo | Mixed Dishes - Sandwiches (single code) |
| 27510600 | Hamburger, 1 oz meat, plain, on mi | Lo | Mixed Dishes - Sandwiches (single code) |
| 27510601 | Hamburger, from fast food, 1 mediu | Lo | Mixed Dishes - Sandwiches (single code) |
| 27510605 | Hamburger, from fast food, 1 large | Lo | Mixed Dishes - Sandwiches (single code) |
| 27510606 | Hamburger, 1 medium patty, with c | Lo | Mixed Dishes - Sandwiches (single code) |
| 27510610 | Hamburger, 1 oz meat, with tomato | Lo | Mixed Dishes - Sandwiches (single code) |
| 27510615 | Whopper (Burger King) | Lo | Mixed Dishes - Sandwiches (single code) |
| 27510616 | Hamburger, 1 medium patty, with c | Lo | Mixed Dishes - Sandwiches (single code) |
| 27510620 | Hamburger, 1/4 lb meat, with tomat | Lo | Mixed Dishes - Sandwiches (single code) |
| 27510631 | Hamburger, on white bun, 1 mediu | Lo | Mixed Dishes - Sandwiches (single code) |
| 27510632 | Hamburger, on wheat bun, 1 mediu | Lo | Mixed Dishes - Sandwiches (single code) |
| 27510633 | Hamburger, 1 medium patty, plain, | Lo | Mixed Dishes - Sandwiches (single code) |
| 27510635 | Hamburger, on white bun, 1 large p | Lo | Mixed Dishes - Sandwiches (single code) |
| 27510636 | Hamburger, on wheat bun, 1 large | Lo | Mixed Dishes - Sandwiches (single code) |
| 27510641 | Hamburger, 1 medium patty, with c | Lo | Mixed Dishes - Sandwiches (single code) |
| 27510642 | Hamburger, 1 medium patty, with c | Lo | Mixed Dishes - Sandwiches (single code) |
| 27510643 | Hamburger, 1 medium patty, with c | Lo | Mixed Dishes - Sandwiches (single code) |
| 27510649 | Double hamburger, on white bun, 2 | Lo | Mixed Dishes - Sandwiches (single code) |
| 27510655 | Double hamburger, on white bun, 2 | Lo | Mixed Dishes - Sandwiches (single code) |
| 27510657 | Double hamburger, on wheat bun, | Lo | Mixed Dishes - Sandwiches (single code) |
| 27510658 | Double hamburger, on white bun, 2 | Lo | Mixed Dishes - Sandwiches (single code) |
| 27510661 | Double hamburger, from fast food, | Lo | Mixed Dishes - Sandwiches (single code) |
| 27510667 | Double hamburger, 2 small patties, | Lo | Mixed Dishes - Sandwiches (single code) |
| 27510670 | Double hamburger (2 patties), with | Lo | Mixed Dishes - Sandwiches (single code) |
| 27510671 | Double hamburger, from fast food, | Lo | Mixed Dishes - Sandwiches (single code) |
| 27510675 | Double hamburger, from fast food, | Lo | Mixed Dishes - Sandwiches (single code) |
| 27510676 | Double hamburger, 2 medium patti | Lo | Mixed Dishes - Sandwiches (single code) |
| 27510680 | Double hamburger (2 patties, 1/4 lb | Lo | Mixed Dishes - Sandwiches (single code) |

| 27510681 | Double hamburger, 2 medium patti | Lo | Mixed Dishes - Sandwiches (single code) |
| --- | --- | --- | --- |
| 27510682 | Double hamburger, 2 medium patti | Lo | Mixed Dishes - Sandwiches (single code) |
| 27510690 | Double hamburger (2 patties, 1/4 lb | Lo | Mixed Dishes - Sandwiches (single code) |
| 27510700 | Meatball and spaghetti sauce subm | Lo | Mixed Dishes - Sandwiches (single code) |
| 27510705 | Chiliburger, with or without cheese, | Lo | Mixed Dishes - Sandwiches (single code) |
| 27510910 | Corned beef sandwich | Lo | Mixed Dishes - Sandwiches (single code) |
| 27510950 | Reuben sandwich, corned beef san | Lo | Mixed Dishes - Sandwiches (single code) |
| 27511010 | Pastrami sandwich | Lo | Mixed Dishes - Sandwiches (single code) |
| 27513010 | Roast beef sandwich | Lo | Mixed Dishes - Sandwiches (single code) |
| 27513040 | Roast beef submarine sandwich, w | Lo | Mixed Dishes - Sandwiches (single code) |
| 27513041 | Roast beef submarine sandwich, w | Lo | Mixed Dishes - Sandwiches (single code) |
| 27513050 | Roast beef sandwich with cheese | Lo | Mixed Dishes - Sandwiches (single code) |
| 27513060 | Roast beef sandwich with bacon an | Lo | Mixed Dishes - Sandwiches (single code) |
| 27513070 | Roast beef submarine sandwich, o | Lo | Mixed Dishes - Sandwiches (single code) |
| 27515000 | Steak submarine sandwich with lett | Lo | Mixed Dishes - Sandwiches (single code) |
| 27515010 | Steak sandwich, plain, on roll | Lo | Mixed Dishes - Sandwiches (single code) |
| 27515020 | Steak and cheese submarine sand | Lo | Mixed Dishes - Sandwiches (single code) |
| 27515030 | Steak and cheese sandwich, plain, | Lo | Mixed Dishes - Sandwiches (single code) |
| 27515040 | Steak and cheese submarine sand | Lo | Mixed Dishes - Sandwiches (single code) |
| 27515050 | Fajita-style beef sandwich with che | Lo | Mixed Dishes - Sandwiches (single code) |
| 27515070 | Steak and cheese submarine sand | Lo | Mixed Dishes - Sandwiches (single code) |
| 27515080 | Steak sandwich, plain, on biscuit | Lo | Mixed Dishes - Sandwiches (single code) |
| 27516010 | Gyro sandwich (pita bread, beef, la | Lo | Mixed Dishes - Sandwiches (single code) |
| 27517000 | Hamburger wrap sandwich, from fa | Lo | Mixed Dishes - Sandwiches (single code) |
| 27517010 | Wrap sandwich filled with beef patt | Lo | Mixed Dishes - Sandwiches (single code) |
| 27520130 | Bacon, chicken, and tomato club sa | Lo | Mixed Dishes - Sandwiches (single code) |
| 27520135 | Bacon, chicken, and tomato club sa | Lo | Mixed Dishes - Sandwiches (single code) |
| 27520140 | Bacon and egg sandwich | Lo | Mixed Dishes - Sandwiches (single code) |
| 27520150 | Bacon, lettuce, and tomato sandwic | Lo | Mixed Dishes - Sandwiches (single code) |
| 27520155 | Bacon, lettuce, and tomato submar | Lo | Mixed Dishes - Sandwiches (single code) |
| 27520156 | Bacon, lettuce, tomato, and cheese | Lo | Mixed Dishes - Sandwiches (single code) |
| 27520160 | Bacon, chicken, and tomato club sa | Lo | Mixed Dishes - Sandwiches (single code) |
| 27520165 | Bacon, breaded fried chicken fillet, | Lo | Mixed Dishes - Sandwiches (single code) |
| 27520166 | Bacon, breaded fried chicken fillet, | Lo | Mixed Dishes - Sandwiches (single code) |
| 27520170 | Bacon on biscuit | Lo | Mixed Dishes - Sandwiches (single code) |
| 27520250 | Ham on biscuit | Lo | Mixed Dishes - Sandwiches (single code) |
| 27520300 | Ham sandwich, with spread | Lo | Mixed Dishes - Sandwiches (single code) |
| 27520310 | Ham sandwich with lettuce and spr | Lo | Mixed Dishes - Sandwiches (single code) |
| 27520320 | Ham and cheese sandwich, with let | Lo | Mixed Dishes - Sandwiches (single code) |
| 27520330 | Ham and egg sandwich | Lo | Mixed Dishes - Sandwiches (single code) |
| 27520350 | Ham and cheese sandwich, with sp | Lo | Mixed Dishes - Sandwiches (single code) |
| 27520360 | Ham and cheese sandwich, on bun | Lo | Mixed Dishes - Sandwiches (single code) |
| 27520370 | Hot ham and cheese sandwich, on | Lo | Mixed Dishes - Sandwiches (single code) |
| 27520380 | Ham and cheese on English muffin | Lo | Mixed Dishes - Sandwiches (single code) |
| 27520390 | Ham and cheese submarine sandw | Lo | Mixed Dishes - Sandwiches (single code) |
| 27520410 | Cuban sandwich, with spread | Lo | Mixed Dishes - Sandwiches (single code) |
| 27520500 | Pork sandwich, on white roll, with o | Lo | Mixed Dishes - Sandwiches (single code) |
| 27520510 | Pork barbecue sandwich or Sloppy | Lo | Mixed Dishes - Sandwiches (single code) |
| 27520520 | Pork sandwich | Lo | Mixed Dishes - Sandwiches (single code) |
| 27540110 | Sliced chicken sandwich, with spre | Lo | Mixed Dishes - Sandwiches (single code) |
| 27540111 | Sliced chicken sandwich, with chee | Lo | Mixed Dishes - Sandwiches (single code) |

| 27540120 | Chicken salad or chicken spread sa | Lo | Mixed Dishes - Sandwiches (single code) |
| --- | --- | --- | --- |
| 27540130 | Chicken barbecue sandwich | Lo | Mixed Dishes - Sandwiches (single code) |
| 27540132 | Chicken fillet sandwich, NFS | Lo | Mixed Dishes - Sandwiches (single code) |
| 27540139 | Chicken fillet sandwich, from schoo | Lo | Mixed Dishes - Sandwiches (single code) |
| 27540140 | Chicken fillet, breaded, fried, sandw | Lo | Mixed Dishes - Sandwiches (single code) |
| 27540145 | Chicken fillet biscuit, from fast food | Lo | Mixed Dishes - Sandwiches (single code) |
| 27540146 | Chicken fillet sandwich, fried, from | Lo | Mixed Dishes - Sandwiches (single code) |
| 27540147 | Chicken fillet sandwich, fried, from | Lo | Mixed Dishes - Sandwiches (single code) |
| 27540150 | Chicken fillet, breaded, fried, sandw | Lo | Mixed Dishes - Sandwiches (single code) |
| 27540151 | Chicken fillet, breaded, fried, sandw | Lo | Mixed Dishes - Sandwiches (single code) |
| 27540152 | Chicken fillet sandwich, grilled, from | Lo | Mixed Dishes - Sandwiches (single code) |
| 27540153 | Chicken fillet sandwich, grilled, from | Lo | Mixed Dishes - Sandwiches (single code) |
| 27540160 | Chicken fillet sandwich, NS as to fri | Lo | Mixed Dishes - Sandwiches (single code) |
| 27540170 | Chicken patty sandwich, miniature, | Lo | Mixed Dishes - Sandwiches (single code) |
| 27540175 | Chicken fillet sandwich, fried, on wh | Lo | Mixed Dishes - Sandwiches (single code) |
| 27540176 | Chicken fillet sandwich, fried, on wh | Lo | Mixed Dishes - Sandwiches (single code) |
| 27540180 | Chicken patty sandwich or biscuit | Lo | Mixed Dishes - Sandwiches (single code) |
| 27540185 | Chicken fillet sandwich, fried, on wh | Lo | Mixed Dishes - Sandwiches (single code) |
| 27540186 | Chicken fillet sandwich, fried, on wh | Lo | Mixed Dishes - Sandwiches (single code) |
| 27540190 | Chicken patty sandwich, with lettuc | Lo | Mixed Dishes - Sandwiches (single code) |
| 27540195 | Chicken fillet sandwich, grilled, on w | Lo | Mixed Dishes - Sandwiches (single code) |
| 27540196 | Chicken fillet sandwich, grilled, on w | Lo | Mixed Dishes - Sandwiches (single code) |
| 27540200 | Fajita-style chicken sandwich with c | Lo | Mixed Dishes - Sandwiches (single code) |
| 27540205 | Chicken fillet sandwich, grilled, on w | Lo | Mixed Dishes - Sandwiches (single code) |
| 27540206 | Chicken fillet sandwich, grilled, on w | Lo | Mixed Dishes - Sandwiches (single code) |
| 27540210 | Chicken fillet wrap sandwich, fried, | Lo | Mixed Dishes - Sandwiches (single code) |
| 27540230 | Chicken patty sandwich with chees | Lo | Mixed Dishes - Sandwiches (single code) |
| 27540235 | Chicken fillet, broiled, sandwich wit | Lo | Mixed Dishes - Sandwiches (single code) |
| 27540240 | Chicken fillet, broiled, sandwich, on | Lo | Mixed Dishes - Sandwiches (single code) |
| 27540250 | Chicken fillet, broiled, sandwich wit | Lo | Mixed Dishes - Sandwiches (single code) |
| 27540260 | Chicken fillet, broiled, sandwich, on | Lo | Mixed Dishes - Sandwiches (single code) |
| 27540270 | Chicken fillet, broiled, sandwich, wit | Lo | Mixed Dishes - Sandwiches (single code) |
| 27540280 | Chicken fillet, broiled, sandwich wit | Lo | Mixed Dishes - Sandwiches (single code) |
| 27540285 | Chicken, bacon, and tomato club s | Lo | Mixed Dishes - Sandwiches (single code) |
| 27540290 | Chicken submarine sandwich, with | Lo | Mixed Dishes - Sandwiches (single code) |
| 27540291 | Chicken submarine sandwich, with | Lo | Mixed Dishes - Sandwiches (single code) |
| 27540295 | Buffalo chicken submarine sandwic | Lo | Mixed Dishes - Sandwiches (single code) |
| 27540296 | Buffalo chicken submarine sandwic | Lo | Mixed Dishes - Sandwiches (single code) |
| 27540300 | Chicken fillet wrap sandwich, grilled | Lo | Mixed Dishes - Sandwiches (single code) |
| 27540310 | Turkey sandwich, with spread | Lo | Mixed Dishes - Sandwiches (single code) |
| 27540350 | Turkey submarine sandwich, with c | Lo | Mixed Dishes - Sandwiches (single code) |
| 27540360 | Turkey and bacon submarine sand | Lo | Mixed Dishes - Sandwiches (single code) |
| 27540361 | Turkey and bacon submarine sand | Lo | Mixed Dishes - Sandwiches (single code) |
| 27541000 | Turkey, ham, and roast beef club s | Lo | Mixed Dishes - Sandwiches (single code) |
| 27541001 | Turkey, ham, and roast beef club s | Lo | Mixed Dishes - Sandwiches (single code) |
| 27545000 | Turkey or chicken burger, plain, on | Lo | Mixed Dishes - Sandwiches (single code) |
| 27545010 | Turkey or chicken burger, with cond | Lo | Mixed Dishes - Sandwiches (single code) |
| 27545100 | Turkey or chicken burger, on white | Lo | Mixed Dishes - Sandwiches (single code) |
| 27545110 | Turkey or chicken burger, on wheat | Lo | Mixed Dishes - Sandwiches (single code) |
| 27545200 | Turkey or chicken burger, with cond | Lo | Mixed Dishes - Sandwiches (single code) |
| 27545210 | Turkey or chicken burger, with cond | Lo | Mixed Dishes - Sandwiches (single code) |

| 27545220 | Turkey or chicken burger, with cond | Lo | Mixed Dishes - Sandwiches (single code) |
| --- | --- | --- | --- |
| 27550000 | Fish sandwich, fried, from fast food | Lo | Mixed Dishes - Sandwiches (single code) |
| 27550100 | Fish sandwich, fried, from fast food | Lo | Mixed Dishes - Sandwiches (single code) |
| 27550110 | Crab cake sandwich | Lo | Mixed Dishes - Sandwiches (single code) |
| 27550120 | Salmon cake sandwich | Lo | Mixed Dishes - Sandwiches (single code) |
| 27550150 | Fried seafood sandwich | Lo | Mixed Dishes - Sandwiches (single code) |
| 27550200 | Fish sandwich, from school cafeteri | Lo | Mixed Dishes - Sandwiches (single code) |
| 27550300 | Fish sandwich, NFS | Lo | Mixed Dishes - Sandwiches (single code) |
| 27550400 | Fish sandwich, fried, on white bun | Lo | Mixed Dishes - Sandwiches (single code) |
| 27550405 | Fish sandwich, fried, on white bun, | Lo | Mixed Dishes - Sandwiches (single code) |
| 27550410 | Fish sandwich, fried, on wheat bun | Lo | Mixed Dishes - Sandwiches (single code) |
| 27550420 | Fish sandwich, grilled | Lo | Mixed Dishes - Sandwiches (single code) |
| 27550425 | Fish wrap sandwich | Lo | Mixed Dishes - Sandwiches (single code) |
| 27550510 | Sardine sandwich | Lo | Mixed Dishes - Sandwiches (single code) |
| 27550720 | Tuna salad sandwich, on bread | Lo | Mixed Dishes - Sandwiches (single code) |
| 27550730 | Tuna salad sandwich, on bread, wit | Lo | Mixed Dishes - Sandwiches (single code) |
| 27550740 | Tuna salad sandwich, on bun | Lo | Mixed Dishes - Sandwiches (single code) |
| 27550745 | Tuna salad sandwich, on bun, with | Lo | Mixed Dishes - Sandwiches (single code) |
| 27550750 | Tuna salad submarine sandwich, w | Lo | Mixed Dishes - Sandwiches (single code) |
| 27550751 | Tuna salad submarine sandwich, w | Lo | Mixed Dishes - Sandwiches (single code) |
| 27550755 | Tuna salad wrap sandwich | Lo | Mixed Dishes - Sandwiches (single code) |
| 27550800 | Seafood salad sandwich | Lo | Mixed Dishes - Sandwiches (single code) |
| 27560110 | Bologna sandwich, with spread | Lo | Mixed Dishes - Sandwiches (single code) |
| 27560120 | Bologna and cheese sandwich, with | Lo | Mixed Dishes - Sandwiches (single code) |
| 27560300 | Corn dog, frankfurter or hot dog wit | Lo | Mixed Dishes - Sandwiches (single code) |
| 27560320 | Frankfurter or hot dog, plain, on bu | Lo | Mixed Dishes - Sandwiches (single code) |
| 27560330 | Frankfurter or hot dog, with cheese | Lo | Mixed Dishes - Sandwiches (single code) |
| 27560340 | Frankfurter or hot dog, with catsup | Lo | Mixed Dishes - Sandwiches (single code) |
| 27560350 | Pig in a blanket, frankfurter or hot d | Lo | Mixed Dishes - Sandwiches (single code) |
| 27560360 | Frankfurter or hot dog, with chili, on | Lo | Mixed Dishes - Sandwiches (single code) |
| 27560370 | Frankfurter or hot dog with chili and | Lo | Mixed Dishes - Sandwiches (single code) |
| 27560400 | Chicken frankfurter or hot dog, plai | Lo | Mixed Dishes - Sandwiches (single code) |
| 27560500 | Pepperoni and salami submarine s | Lo | Mixed Dishes - Sandwiches (single code) |
| 27560510 | Salami sandwich, with spread | Lo | Mixed Dishes - Sandwiches (single code) |
| 27560650 | Sausage on biscuit | Lo | Mixed Dishes - Sandwiches (single code) |
| 27560660 | Sausage griddle cake sandwich | Lo | Mixed Dishes - Sandwiches (single code) |
| 27560670 | Sausage and cheese on English m | Lo | Mixed Dishes - Sandwiches (single code) |
| 27560705 | Sausage balls, made with biscuit m | Lo | Mixed Dishes - Meat, Poultry, Seafood |
| 27560710 | Sausage sandwich | Lo | Mixed Dishes - Sandwiches (single code) |
| 27560910 | Cold cut sumarine sandwich, with c | Lo | Mixed Dishes - Sandwiches (single code) |
| 27564000 | Frankfurter or hot dog sandwich, N | Lo | Mixed Dishes - Sandwiches (single code) |
| 27564001 | Frankfurter or hot dog sandwich, N | Lo | Mixed Dishes - Sandwiches (single code) |
| 27564002 | Frankfurter or hot dog sandwich, N | Lo | Mixed Dishes - Sandwiches (single code) |
| 27564010 | Frankfurter or hot dog sandwich, N | Lo | Mixed Dishes - Sandwiches (single code) |
| 27564020 | Frankfurter or hot dog sandwich, N | Lo | Mixed Dishes - Sandwiches (single code) |
| 27564030 | Frankfurter or hot dog sandwich, N | Lo | Mixed Dishes - Sandwiches (single code) |
| 27564060 | Frankfurter or hot dog sandwich, be | Lo | Mixed Dishes - Sandwiches (single code) |
| 27564061 | Frankfurter or hot dog sandwich, be | Lo | Mixed Dishes - Sandwiches (single code) |
| 27564062 | Frankfurter or hot dog sandwich, be | Lo | Mixed Dishes - Sandwiches (single code) |
| 27564063 | Frankfurter or hot dog sandwich, be | Lo | Mixed Dishes - Sandwiches (single code) |
| 27564064 | Frankfurter or hot dog sandwich, be | Lo | Mixed Dishes - Sandwiches (single code) |

| 27564070 | Frankfurter or hot dog sandwich, be | Lo | Mixed Dishes - Sandwiches (single code) |
| --- | --- | --- | --- |
| 27564080 | Frankfurter or hot dog sandwich, be | Lo | Mixed Dishes - Sandwiches (single code) |
| 27564090 | Frankfurter or hot dog sandwich, be | Lo | Mixed Dishes - Sandwiches (single code) |
| 27564100 | Frankfurter or hot dog sandwich, be | Lo | Mixed Dishes - Sandwiches (single code) |
| 27564110 | Frankfurter or hot dog sandwich, be | Lo | Mixed Dishes - Sandwiches (single code) |
| 27564120 | Frankfurter or hot dog sandwich, be | Lo | Mixed Dishes - Sandwiches (single code) |
| 27564121 | Frankfurter or hot dog sandwich, be | Lo | Mixed Dishes - Sandwiches (single code) |
| 27564122 | Frankfurter or hot dog sandwich, be | Lo | Mixed Dishes - Sandwiches (single code) |
| 27564130 | Frankfurter or hot dog sandwich, be | Lo | Mixed Dishes - Sandwiches (single code) |
| 27564140 | Frankfurter or hot dog sandwich, be | Lo | Mixed Dishes - Sandwiches (single code) |
| 27564150 | Frankfurter or hot dog sandwich, be | Lo | Mixed Dishes - Sandwiches (single code) |
| 27564160 | Frankfurter or hot dog sandwich, be | Lo | Mixed Dishes - Sandwiches (single code) |
| 27564180 | Frankfurter or hot dog sandwich, m | Lo | Mixed Dishes - Sandwiches (single code) |
| 27564181 | Frankfurter or hot dog sandwich, m | Lo | Mixed Dishes - Sandwiches (single code) |
| 27564182 | Frankfurter or hot dog sandwich, m | Lo | Mixed Dishes - Sandwiches (single code) |
| 27564190 | Frankfurter or hot dog sandwich, m | Lo | Mixed Dishes - Sandwiches (single code) |
| 27564200 | Frankfurter or hot dog sandwich, m | Lo | Mixed Dishes - Sandwiches (single code) |
| 27564210 | Frankfurter or hot dog sandwich, m | Lo | Mixed Dishes - Sandwiches (single code) |
| 27564220 | Frankfurter or hot dog sandwich, m | Lo | Mixed Dishes - Sandwiches (single code) |
| 27564230 | Frankfurter or hot dog sandwich, m | Lo | Mixed Dishes - Sandwiches (single code) |
| 27564240 | Frankfurter or hot dog sandwich, ch | Lo | Mixed Dishes - Sandwiches (single code) |
| 27564241 | Frankfurter or hot dog sandwich, ch | Lo | Mixed Dishes - Sandwiches (single code) |
| 27564242 | Frankfurter or hot dog sandwich, ch | Lo | Mixed Dishes - Sandwiches (single code) |
| 27564243 | Frankfurter or hot dog sandwich, ch | Lo | Mixed Dishes - Sandwiches (single code) |
| 27564250 | Frankfurter or hot dog sandwich, ch | Lo | Mixed Dishes - Sandwiches (single code) |
| 27564260 | Frankfurter or hot dog sandwich, ch | Lo | Mixed Dishes - Sandwiches (single code) |
| 27564270 | Frankfurter or hot dog sandwich, ch | Lo | Mixed Dishes - Sandwiches (single code) |
| 27564280 | Frankfurter or hot dog sandwich, ch | Lo | Mixed Dishes - Sandwiches (single code) |
| 27564290 | Frankfurter or hot dog sandwich, ch | Lo | Mixed Dishes - Sandwiches (single code) |
| 27564300 | Frankfurter or hot dog sandwich, re | Lo | Mixed Dishes - Sandwiches (single code) |
| 27564301 | Frankfurter or hot dog sandwich, re | Lo | Mixed Dishes - Sandwiches (single code) |
| 27564303 | Frankfurter or hot dog sandwich, re | Lo | Mixed Dishes - Sandwiches (single code) |
| 27564310 | Frankfurter or hot dog sandwich, re | Lo | Mixed Dishes - Sandwiches (single code) |
| 27564330 | Frankfurter or hot dog sandwich, re | Lo | Mixed Dishes - Sandwiches (single code) |
| 27564360 | Frankfurter or hot dog sandwich, fa | Lo | Mixed Dishes - Sandwiches (single code) |
| 27564361 | Frankfurter or hot dog sandwich, fa | Lo | Mixed Dishes - Sandwiches (single code) |
| 27564362 | Frankfurter or hot dog sandwich, fa | Lo | Mixed Dishes - Sandwiches (single code) |
| 27564364 | Frankfurter or hot dog sandwich, fa | Lo | Mixed Dishes - Sandwiches (single code) |
| 27564370 | Frankfurter or hot dog sandwich, fa | Lo | Mixed Dishes - Sandwiches (single code) |
| 27564380 | Frankfurter or hot dog sandwich, fa | Lo | Mixed Dishes - Sandwiches (single code) |
| 27564418 | Frankfurter or hot dog sandwich, re | Lo | Mixed Dishes - Sandwiches (single code) |
| 27564420 | Frankfurter or hot dog sandwich, m | Lo | Mixed Dishes - Sandwiches (single code) |
| 27564430 | Frankfurter or hot dog sandwich, m | Lo | Mixed Dishes - Sandwiches (single code) |
| 27564440 | Frankfurter or hot dog sandwich, wi | Lo | Mixed Dishes - Sandwiches (single code) |
| 27564441 | Frankfurter or hot dog sandwich, wi | Lo | Mixed Dishes - Sandwiches (single code) |
| 27564442 | Frankfurter or hot dog sandwich, wi | Lo | Mixed Dishes - Sandwiches (single code) |
| 27564443 | Frankfurter or hot dog sandwich, wi | Lo | Mixed Dishes - Sandwiches (single code) |
| 27564450 | Frankfurter or hot dog sandwich, wi | Lo | Mixed Dishes - Sandwiches (single code) |
| 27564460 | Frankfurter or hot dog sandwich, wi | Lo | Mixed Dishes - Sandwiches (single code) |
| 27564500 | Frankfurter or hot dog sandwich, wi | Lo | Mixed Dishes - Sandwiches (single code) |
| 27564510 | Frankfurter or hot dog sandwich, wi | Lo | Mixed Dishes - Sandwiches (single code) |

| 27564520 | Frankfurter or hot dog sandwich, wi | Lo | Mixed Dishes - Sandwiches (single code) |
| --- | --- | --- | --- |
| 27570310 | Hors d'oeuvres, with spread | Lo | Mixed Dishes - Sandwiches (single code) |
| 27601000 | Beef stew, baby food, toddler | Lo | Baby Foods |
| 27610100 | Beef and egg noodles, baby food, N | Lo | Baby Foods |
| 27610110 | Beef and egg noodles, baby food, s | Lo | Baby Foods |
| 27610120 | Beef and egg noodles, baby food, j | Lo | Baby Foods |
| 27610710 | Beef with vegetables, baby food, st | Lo | Baby Foods |
| 27610730 | Beef with vegetables, baby food, to | Lo | Baby Foods |
| 27640050 | Chicken and rice dinner, baby food, | Lo | Baby Foods |
| 27640100 | Chicken noodle dinner, baby food, | Lo | Baby Foods |
| 27640110 | Chicken noodle dinner, baby food, | Lo | Baby Foods |
| 27640120 | Chicken noodle dinner, baby food, j | Lo | Baby Foods |
| 27640810 | Chicken, noodles, and vegetables, | Lo | Baby Foods |
| 27641000 | Chicken stew, baby food, toddler | Lo | Baby Foods |
| 27642100 | Turkey, rice and vegetables, baby f | Lo | Baby Foods |
| 27642110 | Turkey, rice and vegetables, baby f | Lo | Baby Foods |
| 27642120 | Turkey, rice and vegetables, baby f | Lo | Baby Foods |
| 27642130 | Turkey, rice, and vegetables, baby | Lo | Baby Foods |
| 27644110 | Chicken soup, baby food | Lo | Baby Foods |
| 28101000 | Frozen dinner, NFS | Lo | Mixed Dishes - Meat, Poultry, Seafood |
| 28110000 | Beef dinner, NFS, frozen meal | Lo | Mixed Dishes - Meat, Poultry, Seafood |
| 28110110 | Beef with potatoes (frozen meal) | Lo | Mixed Dishes - Meat, Poultry, Seafood |
| 28110120 | Beef with potatoes (frozen meal, lar | Lo | Mixed Dishes - Meat, Poultry, Seafood |
| 28110150 | Beef with vegetable, diet frozen me | Lo | Mixed Dishes - Meat, Poultry, Seafood |
| 28110220 | Sirloin, chopped, with gravy, mashe | Lo | Mixed Dishes - Meat, Poultry, Seafood |
| 28110230 | Sirloin, chopped, or swiss steak wit | Lo | Mixed Dishes - Meat, Poultry, Seafood |
| 28110250 | Sirloin tips, with gravy, potatoes, ve | Lo | Mixed Dishes - Meat, Poultry, Seafood |
| 28110270 | Sirloin beef, with gravy, potatoes, v | Lo | Mixed Dishes - Meat, Poultry, Seafood |
| 28110300 | Salisbury steak dinner, NFS, frozen | Lo | Mixed Dishes - Meat, Poultry, Seafood |
| 28110310 | Salisbury steak with gravy, potatoe | Lo | Mixed Dishes - Meat, Poultry, Seafood |
| 28110330 | Salisbury steak with gravy, whipped | Lo | Mixed Dishes - Meat, Poultry, Seafood |
| 28110350 | Salisbury steak with gravy, potatoe | Lo | Mixed Dishes - Meat, Poultry, Seafood |
| 28110370 | Salisbury steak with gravy, macaro | Lo | Mixed Dishes - Meat, Poultry, Seafood |
| 28110380 | Salisbury steak with gravy, macaro | Lo | Mixed Dishes - Meat, Poultry, Seafood |
| 28110390 | Salisbury steak, potatoes, vegetabl | Lo | Mixed Dishes - Meat, Poultry, Seafood |
| 28110500 | Beef, sliced, with gravy, barley and | Lo | Mixed Dishes - Meat, Poultry, Seafood |
| 28110510 | Beef, sliced, with gravy, potatoes, v | Lo | Mixed Dishes - Meat, Poultry, Seafood |
| 28110620 | Beef short ribs, boneless, with barb | Lo | Mixed Dishes - Meat, Poultry, Seafood |
| 28110640 | Meatballs, Swedish, in sauce, with | Lo | Mixed Dishes - Meat, Poultry, Seafood |
| 28110660 | Meatballs, Swedish, in gravy, with n | Lo | Mixed Dishes - Meat, Poultry, Seafood |
| 28113110 | Salisbury steak, baked, with tomato | Lo | Mixed Dishes - Meat, Poultry, Seafood |
| 28113140 | Beef with spaetzle or rice, vegetabl | Lo | Mixed Dishes - Meat, Poultry, Seafood |
| 28120230 | Pork, sliced, with gravy, mashed po | Lo | Mixed Dishes - Meat, Poultry, Seafood |
| 28120310 | Pork with rice, vegetable, in soy-ba | Lo | Mixed Dishes - Asian |
| 28133110 | Veal, breaded, with spaghetti, in to | Lo | Mixed Dishes - Meat, Poultry, Seafood |
| 28140100 | Chicken dinner, NFS, frozen meal | Lo | Mixed Dishes - Meat, Poultry, Seafood |
| 28140150 | Chicken divan, frozen meal | Lo | Mixed Dishes - Meat, Poultry, Seafood |
| 28140320 | Chicken and noodles with vegetabl | Lo | Mixed Dishes - Meat, Poultry, Seafood |
| 28140710 | Chicken, fried, with potatoes, veget | Lo | Mixed Dishes - Meat, Poultry, Seafood |
| 28140720 | Chicken patty, or nuggets, boneles | Lo | Mixed Dishes - Meat, Poultry, Seafood |
| 28140730 | Chicken patty, breaded, with tomat | Lo | Mixed Dishes - Meat, Poultry, Seafood |

| 28140740 | Chicken patty or nuggets, boneless | Lo | Mixed Dishes - Meat, Poultry, Seafood |
| --- | --- | --- | --- |
| 28140810 | Chicken, fried, with potatoes, veget | Lo | Mixed Dishes - Meat, Poultry, Seafood |
| 28141010 | Chicken, fried, with potatoes, veget | Lo | Mixed Dishes - Meat, Poultry, Seafood |
| 28141050 | Chicken patty parmigiana, breaded | Lo | Mixed Dishes - Meat, Poultry, Seafood |
| 28141060 | Chicken patty with vegetable (diet f | Lo | Mixed Dishes - Meat, Poultry, Seafood |
| 28141200 | Chicken teriyaki with rice, vegetable | Lo | Mixed Dishes - Meat, Poultry, Seafood |
| 28141201 | Teriyaki chicken with rice and vege | Lo | Mixed Dishes - Asian |
| 28141250 | Chicken with rice and vegetable, di | Lo | Mixed Dishes - Meat, Poultry, Seafood |
| 28141300 | Chicken with rice and vegetable, re | Lo | Mixed Dishes - Meat, Poultry, Seafood |
| 28141600 | Chicken a la king with rice, frozen m | Lo | Mixed Dishes - Meat, Poultry, Seafood |
| 28141610 | Chicken and vegetables in cream o | Lo | Mixed Dishes - Meat, Poultry, Seafood |
| 28141650 | Chicken and vegetables au gratin w | Lo | Mixed Dishes - Meat, Poultry, Seafood |
| 28143010 | Chicken and vegetable entree with | Lo | Mixed Dishes - Meat, Poultry, Seafood |
| 28143020 | Chicken and vegetable entree with | Lo | Mixed Dishes - Meat, Poultry, Seafood |
| 28143030 | Chicken and vegetable entree, orie | Lo | Mixed Dishes - Meat, Poultry, Seafood |
| 28143040 | Chicken chow mein with rice, diet fr | Lo | Mixed Dishes - Asian |
| 28143080 | Chicken with noodles and cheese s | Lo | Mixed Dishes - Meat, Poultry, Seafood |
| 28143110 | Chicken cacciatore with noodles, di | Lo | Mixed Dishes - Meat, Poultry, Seafood |
| 28143130 | Chicken and vegetable entree with | Lo | Mixed Dishes - Meat, Poultry, Seafood |
| 28143150 | Chicken and vegetable entree with | Lo | Mixed Dishes - Meat, Poultry, Seafood |
| 28143170 | Chicken in cream sauce with noodl | Lo | Mixed Dishes - Meat, Poultry, Seafood |
| 28143180 | Chicken in butter sauce with potato | Lo | Mixed Dishes - Meat, Poultry, Seafood |
| 28143190 | Chicken in mushroom sauce, white | Lo | Mixed Dishes - Meat, Poultry, Seafood |
| 28143200 | Chicken in soy-based sauce, rice a | Lo | Mixed Dishes - Asian |
| 28143210 | Chicken in orange sauce with almo | Lo | Mixed Dishes - Asian |
| 28143220 | Chicken in barbecue sauce, with ric | Lo | Mixed Dishes - Meat, Poultry, Seafood |
| 28144100 | Chicken and vegetable entree with | Lo | Mixed Dishes - Meat, Poultry, Seafood |
| 28145000 | Turkey dinner, NFS, frozen meal | Lo | Mixed Dishes - Meat, Poultry, Seafood |
| 28145010 | Turkey with dressing, gravy, potato | Lo | Mixed Dishes - Meat, Poultry, Seafood |
| 28145100 | Turkey with gravy, dressing, vegeta | Lo | Mixed Dishes - Meat, Poultry, Seafood |
| 28145110 | Turkey with vegetable, stuffing, diet | Lo | Mixed Dishes - Meat, Poultry, Seafood |
| 28145210 | Turkey with gravy, dressing, potato | Lo | Mixed Dishes - Meat, Poultry, Seafood |
| 28145610 | Turkey with gravy, dressing, potato | Lo | Mixed Dishes - Meat, Poultry, Seafood |
| 28145710 | Turkey tetrazzini, frozen meal | Lo | Mixed Dishes - Meat, Poultry, Seafood |
| 28150000 | Fish dinner, NFS (frozen meal) | Lo | Mixed Dishes - Meat, Poultry, Seafood |
| 28150210 | Haddock with chopped spinach, die | Lo | Mixed Dishes - Meat, Poultry, Seafood |
| 28150220 | Flounder with chopped broccoli, die | Lo | Mixed Dishes - Meat, Poultry, Seafood |
| 28150510 | Fish in lemon-butter sauce with sta | Lo | Mixed Dishes - Meat, Poultry, Seafood |
| 28150650 | Fish, breaded, or fish sticks, with p | Lo | Mixed Dishes - Meat, Poultry, Seafood |
| 28152030 | Seafood newburg with rice, vegetab | Lo | Mixed Dishes - Meat, Poultry, Seafood |
| 28152050 | Shrimp with rice, vegetable (frozen | Lo | Mixed Dishes - Meat, Poultry, Seafood |
| 28153010 | Shrimp and clams in tomato-based | Lo | Mixed Dishes - Meat, Poultry, Seafood |
| 28154010 | Shrimp and vegetables in sauce wit | Lo | Mixed Dishes - Meat, Poultry, Seafood |
| 28160300 | Meat loaf dinner, NFS, frozen meal | Lo | Mixed Dishes - Meat, Poultry, Seafood |
| 28160310 | Meat loaf with potatoes, vegetable, | Lo | Mixed Dishes - Meat, Poultry, Seafood |
| 28160650 | Stuffed green pepper, frozen meal | Lo | Mixed Dishes - Bean/Vegetable-based |
| 28160710 | Stuffed cabbage, with meat and tom | Lo | Mixed Dishes - Meat, Poultry, Seafood |
| 28310110 | Beef, broth, bouillon, or consomme | Lo | Mixed Dishes - Soups |
| 28310150 | Oxtail soup | Lo | Mixed Dishes - Soups |
| 28310160 | Beef broth, with tomato, home recip | Lo | Mixed Dishes - Soups |
| 28310170 | Beef broth, without tomato, home r | Lo | Mixed Dishes - Soups |

| 28310210 | Chili beef soup | Lo | Mixed Dishes - Soups |
| --- | --- | --- | --- |
| 28310220 | Chili beef soup, chunky style | Lo | Mixed Dishes - Soups |
| 28310230 | Meatball soup, home recipe, Mexic | Lo | Mixed Dishes - Soups |
| 28310320 | Beef noodle soup, Puerto Rican sty | Lo | Mixed Dishes - Soups |
| 28310330 | Pho | Lo | Mixed Dishes - Soups |
| 28310420 | Beef and rice soup, Puerto Rican st | Lo | Mixed Dishes - Soups |
| 28311010 | Pepperpot soup | Lo | Mixed Dishes - Soups |
| 28311020 | Menudo soup, home recipe | Lo | Mixed Dishes - Soups |
| 28311030 | Menudo soup, canned, prepared wi | Lo | Mixed Dishes - Soups |
| 28315050 | Beef vegetable soup with potato, pa | Lo | Mixed Dishes - Soups |
| 28315100 | Beef vegetable soup with potato, st | Lo | Mixed Dishes - Soups |
| 28315120 | Beef vegetable soup with noodles, | Lo | Mixed Dishes - Soups |
| 28315130 | Beef vegetable soup with rice, stew | Lo | Mixed Dishes - Soups |
| 28315140 | Beef vegetable soup, home recipe, | Lo | Mixed Dishes - Soups |
| 28315150 | Meat and corn hominy soup, home | Lo | Mixed Dishes - Soups |
| 28315160 | Italian Wedding Soup | Lo | Mixed Dishes - Soups |
| 28316020 | Beef and mushroom soup, canned, | Lo | Mixed Dishes - Soups |
| 28317010 | Beef stroganoff soup, chunky style, | Lo | Mixed Dishes - Soups |
| 28320110 | Pork and rice soup, stew type, chun | Lo | Mixed Dishes - Soups |
| 28320120 | Pork vegetable soup with noodles, | Lo | Mixed Dishes - Soups |
| 28320130 | Ham, rice, and potato soup, Puerto | Lo | Mixed Dishes - Soups |
| 28320140 | Ham, noodle, and vegetable soup, | Lo | Mixed Dishes - Soups |
| 28320150 | Pork, vegetable soup with potatoes | Lo | Mixed Dishes - Soups |
| 28320160 | Pork vegetable soup with potato, pa | Lo | Mixed Dishes - Soups |
| 28320300 | Pork with vegetable excluding carro | Lo | Mixed Dishes - Soups |
| 28321130 | Bacon soup, cream of, prepared wi | Lo | Mixed Dishes - Soups |
| 28330110 | Scotch broth (lamb, vegetables, an | Lo | Mixed Dishes - Soups |
| 28331110 | Lamb, pasta, and vegetable soup, | Lo | Mixed Dishes - Soups |
| 28340110 | Chicken or turkey broth, bouillon, o | Lo | Mixed Dishes - Soups |
| 28340120 | Chicken or turkey broth, without tom | Lo | Mixed Dishes - Soups |
| 28340130 | Chicken or turkey broth, with tomat | Lo | Mixed Dishes - Soups |
| 28340140 | Chicken broth, bouillion, or consom | Lo | Other |
| 28340150 | Mexican style chicken broth soup s | Lo | Mixed Dishes - Soups |
| 28340170 | Chicken broth, canned, low sodium | Lo | Mixed Dishes - Soups |
| 28340179 | Beef broth, less or reduced sodium | Lo | Mixed Dishes - Soups |
| 28340180 | Chicken or turkey broth, less or red | Lo | Mixed Dishes - Soups |
| 28340210 | Chicken rice soup, Puerto Rican sty | Lo | Mixed Dishes - Soups |
| 28340220 | Chicken soup with noodles and pot | Lo | Mixed Dishes - Soups |
| 28340310 | Chicken or turkey gumbo soup, ho | Lo | Mixed Dishes - Soups |
| 28340510 | Chicken or turkey noodle soup, chu | Lo | Mixed Dishes - Soups |
| 28340520 | Chicken soup, canned, undiluted | Lo | Mixed Dishes - Soups |
| 28340530 | Chicken soup | Lo | Mixed Dishes - Soups |
| 28340550 | Sweet and sour soup | Lo | Mixed Dishes - Soups |
| 28340580 | Chicken or turkey soup with vegeta | Lo | Mixed Dishes - Soups |
| 28340590 | Chicken or turkey corn soup with no | Lo | Mixed Dishes - Soups |
| 28340600 | Chicken or turkey vegetable soup, | Lo | Mixed Dishes - Soups |
| 28340610 | Chicken or turkey vegetable soup, | Lo | Mixed Dishes - Soups |
| 28340630 | Chicken or turkey vegetable soup w | Lo | Mixed Dishes - Soups |
| 28340640 | Chicken or turkey vegetable soup w | Lo | Mixed Dishes - Soups |
| 28340660 | Chicken or turkey vegetable soup, | Lo | Mixed Dishes - Soups |
| 28340670 | Chicken or turkey vegetable soup w | Lo | Mixed Dishes - Soups |

| 28340680 | Chicken or turkey and corn hominy | Lo | Mixed Dishes - Soups |
| --- | --- | --- | --- |
| 28340690 | Chicken or turkey vegetable soup w | Lo | Mixed Dishes - Soups |
| 28340700 | Bird's nest soup | Lo | Mixed Dishes - Soups |
| 28340750 | Hot and sour soup | Lo | Mixed Dishes - Soups |
| 28340800 | Chicken or turkey soup with vegeta | Lo | Mixed Dishes - Soups |
| 28345010 | Chicken or turkey soup, cream of, c | Lo | Mixed Dishes - Soups |
| 28345020 | Chicken or turkey soup, cream of, c | Lo | Mixed Dishes - Soups |
| 28345030 | Chicken or turkey soup, cream of, c | Lo | Mixed Dishes - Soups |
| 28345040 | Chicken or turkey soup, cream of, c | Lo | Mixed Dishes - Soups |
| 28345110 | Chicken or turkey soup, cream of, | Lo | Mixed Dishes - Soups |
| 28345120 | Chicken or turkey soup, cream of, p | Lo | Mixed Dishes - Soups |
| 28345130 | Chicken or turkey soup, cream of, p | Lo | Mixed Dishes - Soups |
| 28345140 | Chicken or turkey soup, cream of, c | Lo | Mixed Dishes - Soups |
| 28345160 | Chicken or turkey mushroom soup, | Lo | Mixed Dishes - Soups |
| 28345170 | Duck soup | Lo | Mixed Dishes - Soups |
| 28350040 | Fish stock, home recipe | Lo | Mixed Dishes - Soups |
| 28350050 | Fish chowder | Lo | Mixed Dishes - Soups |
| 28350110 | Crab soup, NS as to tomato-base o | Lo | Mixed Dishes - Soups |
| 28350120 | Crab soup, tomato-base | Lo | Mixed Dishes - Soups |
| 28350210 | Clam chowder, NS as to Manhattan | Lo | Mixed Dishes - Soups |
| 28350220 | Clam chowder, Manhattan | Lo | Mixed Dishes - Soups |
| 28351110 | Fish and vegetable soup, no potato | Lo | Mixed Dishes - Soups |
| 28351120 | Fish soup with potatoes, Mexican s | Lo | Mixed Dishes - Soups |
| 28351160 | Codfish, rice, and vegetable soup, | Lo | Mixed Dishes - Soups |
| 28355110 | Clam chowder, New England, NS a | Lo | Mixed Dishes - Soups |
| 28355120 | Clam chowder, New England, prep | Lo | Mixed Dishes - Soups |
| 28355130 | Clam chowder, New England, prep | Lo | Mixed Dishes - Soups |
| 28355140 | Clam chowder, New England, redu | Lo | Mixed Dishes - Soups |
| 28355210 | Crab soup, cream of, prepared with | Lo | Mixed Dishes - Soups |
| 28355250 | Lobster bisque | Lo | Mixed Dishes - Soups |
| 28355260 | Lobster gumbo | Lo | Mixed Dishes - Meat, Poultry, Seafood |
| 28355310 | Oyster stew | Lo | Mixed Dishes - Soups |
| 28355350 | Salmon soup, cream style | Lo | Mixed Dishes - Soups |
| 28355410 | Shrimp soup, cream of, NS as to pr | Lo | Mixed Dishes - Soups |
| 28355420 | Shrimp soup, cream of, prepared w | Lo | Mixed Dishes - Soups |
| 28355430 | Shrimp soup, cream of, prepared w | Lo | Mixed Dishes - Soups |
| 28355440 | Shrimp gumbo | Lo | Mixed Dishes - Meat, Poultry, Seafood |
| 28355450 | Seafood soup with potatoes and ve | Lo | Mixed Dishes - Soups |
| 28355460 | Seafood soup with potatoes, and ve | Lo | Mixed Dishes - Soups |
| 28355470 | Seafood soup with vegetables inclu | Lo | Mixed Dishes - Soups |
| 28355480 | Seafood soup with vegetables excl | Lo | Mixed Dishes - Soups |
| 28360100 | Meat broth, Puerto Rican style | Lo | Mixed Dishes - Soups |
| 28401200 | Gelatin drink, powder, flavored, with | Lo | Diet Beverages |
| 28410000 | Bee pollen | Lo | Other |
| 28500000 | Gravy, poultry | Lo | Condiments and Sauces |
| 28500010 | Gravy, meat or poultry, with wine | Lo | Condiments and Sauces |
| 28500020 | Gravy, meat, with fruit | Lo | Condiments and Sauces |
| 28500030 | Gravy, poultry, low sodium | Lo | Condiments and Sauces |
| 28500040 | Gravy, beef | Lo | Condiments and Sauces |
| 28500050 | Gravy, giblet | Lo | Condiments and Sauces |
| 28500070 | Gravy, beef or meat, home recipe | Lo | Condiments and Sauces |

| 28500080 | Gravy, poultry, home recipe | Lo | Condiments and Sauces |
| --- | --- | --- | --- |
| 28500100 | Gravy, mushroom | Lo | Condiments and Sauces |
| 28501010 | Gravy, beef, fat free | Lo | Condiments and Sauces |
| 28501110 | Gravy, poultry, fat free | Lo | Condiments and Sauces |
| 28510010 | Gravy or sauce, poultry-based from | Lo | Condiments and Sauces |
| 28510030 | Gravy, meat-based, from Puerto-Ri | Lo | Condiments and Sauces |
| 28520000 | Gravy, made with soy sauce | Lo | Condiments and Sauces |
| 28520010 | Gravy, NFS | Lo | Condiments and Sauces |
| 28520100 | Oyster sauce | Lo | Condiments and Sauces |
| 28522000 | Mole sauce | Lo | Condiments and Sauces |
| 28522050 | Mole verde sauce | Lo | Condiments and Sauces |
| 31101010 | Egg, whole, raw | Lo | Eggs |
| 31102000 | Egg, whole, cooked, NS as to cook | Lo | Eggs |
| 31103000 | Egg, whole, boiled | Lo | Eggs |
| 31103010 | Egg, whole, boiled or poached | Lo | Eggs |
| 31104000 | Egg, whole, poached | Lo | Eggs |
| 31105000 | Egg, whole, fried | Lo | Eggs |
| 31105005 | Egg, whole, fried, NS as to fat | Lo | Eggs |
| 31105010 | Egg, whole, fried no added fat | Lo | Eggs |
| 31105020 | Egg, whole, fried with margarine | Lo | Eggs |
| 31105030 | Egg, whole, fried with oil | Lo | Eggs |
| 31105040 | Egg, whole, fried with butter | Lo | Eggs |
| 31105060 | Egg, whole, fried with animal fat or | Lo | Eggs |
| 31105080 | Egg, whole, fried with cooking spra | Lo | Eggs |
| 31105085 | Egg, whole, fried, NS as to fat type | Lo | Eggs |
| 31105090 | Egg, whole, fried, from fast food / re | Lo | Eggs |
| 31106000 | Egg, whole, baked, NS as to fat | Lo | Eggs |
| 31106010 | Egg, whole, baked, no added fat | Lo | Eggs |
| 31106020 | Egg, whole, baked, fat added | Lo | Eggs |
| 31107000 | Egg, whole, pickled | Lo | Eggs |
| 31108010 | Egg, white only, raw | Lo | Eggs |
| 31108100 | Egg, white, cooked, NS as to fat | Lo | Eggs |
| 31108110 | Egg, white, cooked, no added fat | Lo | Eggs |
| 31108120 | Egg, white, cooked, fat added | Lo | Eggs |
| 31109000 | Egg, white only, cooked, NS as to f | Lo | Eggs |
| 31109010 | Egg, white only, cooked, fat not add | Lo | Eggs |
| 31109020 | Egg, white only, cooked, fat added | Lo | Eggs |
| 31110010 | Egg, yolk only, raw | Lo | Eggs |
| 31111000 | Egg, yolk only, cooked, NS as to fat | Lo | Eggs |
| 31111010 | Egg, yolk only, cooked, no added fa | Lo | Eggs |
| 31111020 | Egg, yolk only, cooked, fat added | Lo | Eggs |
| 31201000 | Duck egg, cooked | Lo | Eggs |
| 31202000 | Goose egg, cooked | Lo | Eggs |
| 31203000 | Quail egg, canned | Lo | Eggs |
| 32101500 | Egg, Benedict | Lo | Eggs |
| 32101530 | Egg curry | Lo | Mixed Dishes - Bean/Vegetable-based |
| 32102000 | Egg, deviled | Lo | Eggs |
| 32103000 | Egg salad, made with mayonnaise | Lo | Eggs |
| 32103015 | Egg salad, made with light mayonn | Lo | Eggs |
| 32103020 | Egg salad, made with mayonnaise- | Lo | Eggs |
| 32103025 | Egg salad, made with light mayonn | Lo | Eggs |

| 32103035 | Egg salad, made with light creamy | Lo | Eggs |
| --- | --- | --- | --- |
| 32103050 | Egg Salad, made with any type of f | Lo | Eggs |
| 32104900 | Egg omelet or scrambled egg, NS a | Lo | Eggs |
| 32104950 | Egg omelet or scrambled egg, fat n | Lo | Eggs |
| 32105000 | Egg omelet or scrambled egg, fat a | Lo | Eggs |
| 32105010 | Egg omelet or scrambled egg, with | Lo | Eggs |
| 32105013 | Egg omelet or scrambled egg, with | Lo | Eggs |
| 32105020 | Egg omelet or scrambled egg, with | Lo | Eggs |
| 32105030 | Egg omelet or scrambled egg, with | Lo | Eggs |
| 32105040 | Egg omelet or scrambled egg, with | Lo | Eggs |
| 32105045 | Egg omelet or scrambled egg, with | Lo | Eggs |
| 32105048 | Egg omelet or scrambled egg, with | Lo | Eggs |
| 32105050 | Egg omelet or scrambled egg, with | Lo | Eggs |
| 32105055 | Egg omelet or scrambled egg, with | Lo | Eggs |
| 32105060 | Egg omelet or scrambled egg, with | Lo | Eggs |
| 32105070 | Egg omelet or scrambled egg, with | Lo | Eggs |
| 32105080 | Egg omelet or scrambled egg, with | Lo | Eggs |
| 32105081 | Egg omelet or scrambled egg, with | Lo | Eggs |
| 32105082 | Egg omelet or scrambled egg, with | Lo | Eggs |
| 32105085 | Egg omelet or scrambled egg, with | Lo | Eggs |
| 32105100 | Egg omelet or scrambled egg, with | Lo | Eggs |
| 32105110 | Egg omelet or scrambled egg, with | Lo | Eggs |
| 32105118 | Egg omelet or scrambled egg, with | Lo | Eggs |
| 32105119 | Egg omelet or scrambled egg, with | Lo | Eggs |
| 32105120 | Egg omelet or scrambled egg, with | Lo | Eggs |
| 32105121 | Egg omelet or scrambled egg, with | Lo | Eggs |
| 32105122 | Egg omelet or scrambled egg, with | Lo | Eggs |
| 32105125 | Egg omelet or scrambled egg, with | Lo | Eggs |
| 32105126 | Egg omelet or scrambled egg, with | Lo | Eggs |
| 32105130 | Egg omelet or scrambled egg, Spa | Lo | Eggs |
| 32105150 | Egg omelet or scrambled egg, with | Lo | Eggs |
| 32105160 | Egg omelet or scrambled egg, with | Lo | Eggs |
| 32105161 | Egg omelet or scrambled egg, with | Lo | Eggs |
| 32105170 | Egg omelet or scrambled egg, with | Lo | Eggs |
| 32105180 | Huevos rancheros | Lo | Eggs |
| 32105190 | Egg casserole with bread, cheese, | Lo | Eggs |
| 32105200 | Egg foo yung, NFS | Lo | Mixed Dishes - Asian |
| 32105210 | Chicken egg foo yung | Lo | Mixed Dishes - Asian |
| 32105220 | Pork egg foo yung | Lo | Mixed Dishes - Asian |
| 32105230 | Shrimp egg foo yung | Lo | Mixed Dishes - Asian |
| 32105240 | Beef egg foo yung | Lo | Mixed Dishes - Asian |
| 32105310 | Ripe plantain omelet, Puerto Rican | Lo | Eggs |
| 32105330 | Scrambled eggs with jerked beef, P | Lo | Eggs |
| 32110150 | Shrimp-egg patty | Lo | Eggs |
| 32129990 | Egg omelet or scrambled egg, NS a | Lo | Eggs |
| 32130000 | Egg omelet or scrambled egg, mad | Lo | Eggs |
| 32130010 | Egg omelet or scrambled egg, mad | Lo | Eggs |
| 32130020 | Egg omelet or scrambled egg, mad | Lo | Eggs |
| 32130040 | Egg omelet or scrambled egg, mad | Lo | Eggs |
| 32130060 | Egg omelet or scrambled egg, mad | Lo | Eggs |
| 32130065 | Egg omelet or scrambled egg, NS a | Lo | Eggs |

| 32130070 | Egg omelet or scrambled egg, no a | Lo | Eggs |
| --- | --- | --- | --- |
| 32130080 | Egg omelet or scrambled egg, from | Lo | Eggs |
| 32130100 | Egg omelet or scrambled egg, with | Lo | Eggs |
| 32130110 | Egg omelet or scrambled egg, with | Lo | Eggs |
| 32130120 | Egg omelet or scrambled egg, with | Lo | Eggs |
| 32130140 | Egg omelet or scrambled egg, with | Lo | Eggs |
| 32130160 | Egg omelet or scrambled egg, with | Lo | Eggs |
| 32130170 | Egg omelet or scrambled egg, with | Lo | Eggs |
| 32130190 | Egg omelet or scrambled egg, with | Lo | Eggs |
| 32130200 | Egg omelet or scrambled egg, with | Lo | Eggs |
| 32130210 | Egg omelet or scrambled egg, with | Lo | Eggs |
| 32130220 | Egg omelet or scrambled egg, with | Lo | Eggs |
| 32130240 | Egg omelet or scrambled egg, with | Lo | Eggs |
| 32130260 | Egg omelet or scrambled egg, with | Lo | Eggs |
| 32130265 | Egg omelet or scrambled egg, with | Lo | Eggs |
| 32130270 | Egg omelet or scrambled egg, with | Lo | Eggs |
| 32130290 | Egg omelet or scrambled egg, with | Lo | Eggs |
| 32130300 | Egg omelet or scrambled egg, with | Lo | Eggs |
| 32130310 | Egg omelet or scrambled egg, with | Lo | Eggs |
| 32130320 | Egg omelet or scrambled egg, with | Lo | Eggs |
| 32130360 | Egg omelet or scrambled egg, with | Lo | Eggs |
| 32130365 | Egg omelet or scrambled egg, with | Lo | Eggs |
| 32130370 | Egg omelet or scrambled egg, with | Lo | Eggs |
| 32130400 | Egg omelet or scrambled egg, with | Lo | Eggs |
| 32130410 | Egg omelet or scrambled egg, with | Lo | Eggs |
| 32130420 | Egg omelet or scrambled egg, with | Lo | Eggs |
| 32130430 | Egg omelet or scrambled egg, with | Lo | Eggs |
| 32130440 | Egg omelet or scrambled egg, with | Lo | Eggs |
| 32130450 | Egg omelet or scrambled egg, with | Lo | Eggs |
| 32130460 | Egg omelet or scrambled egg, with | Lo | Eggs |
| 32130480 | Egg omelet or scrambled egg, with | Lo | Eggs |
| 32130490 | Egg omelet or scrambled egg, with | Lo | Eggs |
| 32130500 | Egg omelet or scrambled egg, with | Lo | Eggs |
| 32130510 | Egg omelet or scrambled egg, with | Lo | Eggs |
| 32130600 | Egg omelet or scrambled egg, with | Lo | Eggs |
| 32130610 | Egg omelet or scrambled egg, with | Lo | Eggs |
| 32130620 | Egg omelet or scrambled egg, with | Lo | Eggs |
| 32130630 | Egg omelet or scrambled egg, with | Lo | Eggs |
| 32130640 | Egg omelet or scrambled egg, with | Lo | Eggs |
| 32130650 | Egg omelet or scrambled egg, with | Lo | Eggs |
| 32130660 | Egg omelet or scrambled egg, with | Lo | Eggs |
| 32130680 | Egg omelet or scrambled egg, with | Lo | Eggs |
| 32130690 | Egg omelet or scrambled egg, with | Lo | Eggs |
| 32130700 | Egg omelet or scrambled egg, with | Lo | Eggs |
| 32130710 | Egg omelet or scrambled egg, with | Lo | Eggs |
| 32130800 | Egg omelet or scrambled egg, with | Lo | Eggs |
| 32130810 | Egg omelet or scrambled egg, with | Lo | Eggs |
| 32130820 | Egg omelet or scrambled egg, with | Lo | Eggs |
| 32130830 | Egg omelet or scrambled egg, with | Lo | Eggs |
| 32130840 | Egg omelet or scrambled egg, with | Lo | Eggs |
| 32130850 | Egg omelet or scrambled egg, with | Lo | Eggs |

| 32130890 | Egg omelet or scrambled egg, with | Lo | Eggs |
| --- | --- | --- | --- |
| 32130900 | Egg omelet or scrambled egg, with | Lo | Eggs |
| 32130910 | Egg omelet or scrambled egg, with | Lo | Eggs |
| 32131000 | Egg omelet or scrambled egg, with | Lo | Eggs |
| 32131010 | Egg omelet or scrambled egg, with | Lo | Eggs |
| 32131020 | Egg omelet or scrambled egg, with | Lo | Eggs |
| 32131030 | Egg omelet or scrambled egg, with | Lo | Eggs |
| 32131040 | Egg omelet or scrambled egg, with | Lo | Eggs |
| 32131050 | Egg omelet or scrambled egg, with | Lo | Eggs |
| 32131060 | Egg omelet or scrambled egg, with | Lo | Eggs |
| 32131070 | Egg omelet or scrambled egg, with | Lo | Eggs |
| 32131080 | Egg omelet or scrambled egg, with | Lo | Eggs |
| 32131090 | Egg omelet or scrambled egg, with | Lo | Eggs |
| 32131100 | Egg omelet or scrambled egg, with | Lo | Eggs |
| 32131110 | Egg omelet or scrambled egg, with | Lo | Eggs |
| 32131200 | Egg omelet or scrambled egg, with | Lo | Eggs |
| 32131210 | Egg omelet or scrambled egg, with | Lo | Eggs |
| 32131220 | Egg omelet or scrambled egg, with | Lo | Eggs |
| 32201000 | Fried egg sandwich | Lo | Mixed Dishes - Sandwiches (single code) |
| 32202000 | Egg, cheese, ham, and bacon on b | Lo | Mixed Dishes - Sandwiches (single code) |
| 32202010 | Egg, cheese, and ham on English | Lo | Mixed Dishes - Sandwiches (single code) |
| 32202020 | Egg, cheese, and ham on biscuit | Lo | Mixed Dishes - Sandwiches (single code) |
| 32202025 | Egg, cheese and ham on bagel | Lo | Mixed Dishes - Sandwiches (single code) |
| 32202030 | Egg, cheese, and sausage on Engl | Lo | Mixed Dishes - Sandwiches (single code) |
| 32202034 | Egg, cheese, and sausage on bun | Lo | Mixed Dishes - Sandwiches (single code) |
| 32202035 | Egg, extra cheese, and extra sausa | Lo | Mixed Dishes - Sandwiches (single code) |
| 32202040 | Egg, cheese, and beef on English | Lo | Mixed Dishes - Sandwiches (single code) |
| 32202045 | Egg, cheese, and steak on bagel | Lo | Mixed Dishes - Sandwiches (single code) |
| 32202050 | Egg, cheese, and sausage on biscu | Lo | Mixed Dishes - Sandwiches (single code) |
| 32202055 | Egg, cheese, and sausage griddle | Lo | Mixed Dishes - Sandwiches (single code) |
| 32202060 | Egg and sausage on biscuit | Lo | Mixed Dishes - Sandwiches (single code) |
| 32202070 | Egg, cheese, and bacon on biscuit | Lo | Mixed Dishes - Sandwiches (single code) |
| 32202075 | Egg, cheese, and bacon griddle ca | Lo | Mixed Dishes - Sandwiches (single code) |
| 32202080 | Egg, cheese, and bacon on English | Lo | Mixed Dishes - Sandwiches (single code) |
| 32202085 | Egg, cheese and bacon on bagel | Lo | Mixed Dishes - Sandwiches (single code) |
| 32202090 | Egg and bacon on biscuit | Lo | Mixed Dishes - Sandwiches (single code) |
| 32202110 | Egg and ham on biscuit | Lo | Mixed Dishes - Sandwiches (single code) |
| 32202120 | Egg, cheese and sausage on bagel | Lo | Mixed Dishes - Sandwiches (single code) |
| 32202130 | Egg and steak on biscuit | Lo | Mixed Dishes - Sandwiches (single code) |
| 32202200 | Egg and cheese on biscuit | Lo | Mixed Dishes - Sandwiches (single code) |
| 32204010 | Scrambled egg sandwich | Lo | Mixed Dishes - Sandwiches (single code) |
| 32300100 | Egg drop soup | Lo | Mixed Dishes - Soups |
| 32301100 | Garlic egg soup, Puerto Rican style | Lo | Mixed Dishes - Soups |
| 32400010 | Egg white omelet or scrambled egg | Lo | Eggs |
| 32400011 | Egg white omelet or scrambled egg | Lo | Eggs |
| 32400012 | Egg white omelet or scrambled egg | Lo | Eggs |
| 32400050 | Egg white omelet or scrambled egg | Lo | Eggs |
| 32400055 | Egg white omelet, scrambled, or fri | Lo | Eggs |
| 32400060 | Egg white omelet, scrambled, or fri | Lo | Eggs |
| 32400065 | Egg white omelet, scrambled, or fri | Lo | Eggs |
| 32400070 | Egg white omelet, scrambled, or fri | Lo | Eggs |

| 32400075 | Egg white omelet, scrambled, or fri | Lo | Eggs |
| --- | --- | --- | --- |
| 32400078 | Egg white omelet, scrambled, or fri | Lo | Eggs |
| 32400080 | Egg white omelet, scrambled, or fri | Lo | Eggs |
| 32400100 | Egg white, omelet, scrambled, or fri | Lo | Eggs |
| 32400110 | Egg white, omelet, scrambled, or fri | Lo | Eggs |
| 32400120 | Egg white, omelet, scrambled, or fri | Lo | Eggs |
| 32400200 | Egg white, omelet, scrambled, or fri | Lo | Eggs |
| 32400210 | Egg white, omelet, scrambled, or fri | Lo | Eggs |
| 32400220 | Egg white, omelet, scrambled, or fri | Lo | Eggs |
| 32400300 | Egg white, omelet, scrambled, or fri | Lo | Eggs |
| 32400310 | Egg white, omelet, scrambled, or fri | Lo | Eggs |
| 32400320 | Egg white, omelet, scrambled, or fri | Lo | Eggs |
| 32400400 | Egg white, omelet, scrambled, or fri | Lo | Eggs |
| 32400410 | Egg white, omelet, scrambled, or fri | Lo | Eggs |
| 32400420 | Egg white, omelet, scrambled, or fri | Lo | Eggs |
| 32400500 | Egg white, omelet, scrambled, or fri | Lo | Eggs |
| 32400510 | Egg white, omelet, scrambled, or fri | Lo | Eggs |
| 32400520 | Egg white, omelet, scrambled, or fri | Lo | Eggs |
| 32400600 | Egg white, omelet, scrambled, or fri | Lo | Eggs |
| 32400610 | Egg white, omelet, scrambled, or fri | Lo | Eggs |
| 32400620 | Egg white, omelet, scrambled, or fri | Lo | Eggs |
| 32400700 | Egg white, omelet, scrambled, or fri | Lo | Eggs |
| 32400710 | Egg white, omelet, scrambled, or fri | Lo | Eggs |
| 32400720 | Egg white, omelet, scrambled, or fri | Lo | Eggs |
| 32401000 | Meringues | Lo | Other Desserts |
| 33000100 | Egg substitute, NS as to powdered, | Lo | Eggs |
| 33000990 | Egg substitute, omelet, scrambled, | Lo | Eggs |
| 33001000 | Egg substitute, omelet, scrambled, | Lo | Eggs |
| 33001010 | Egg substitute, omelet, scrambled, | Lo | Eggs |
| 33001020 | Egg substitute, omelet, scrambled, | Lo | Eggs |
| 33001040 | Egg substitute, omelet, scrambled, | Lo | Eggs |
| 33001050 | Egg substitute, omelet, scrambled, | Lo | Eggs |
| 33001200 | Egg substitute, vegetable flavored, | Lo | Eggs |
| 33001210 | Egg substitute, vegetable flavored, | Lo | Eggs |
| 33102010 | Scrambled egg, made from powder | Lo | Eggs |
| 33201010 | Scrambled egg, made from cholest | Lo | Eggs |
| 33201110 | Scrambled egg, made from cholest | Lo | Eggs |
| 33201500 | Scrambled egg, made from cholest | Lo | Eggs |
| 33202010 | Scrambled egg, made from frozen | Lo | Eggs |
| 33301010 | Scrambled egg, made from packag | Lo | Eggs |
| 33401000 | Egg substitute, omelet, scrambled, | Lo | Eggs |
| 33401020 | Egg substitute, omelet, scrambled, | Lo | Eggs |
| 33401100 | Egg substitute, omelet, scrambled, | Lo | Eggs |
| 33401110 | Egg substitute, omelet, scrambled, | Lo | Eggs |
| 33401200 | Egg substitute, omelet, scrambled, | Lo | Eggs |
| 33401220 | Egg substitute, omelet, scrambled, | Lo | Eggs |
| 33401300 | Egg substitute, omelet, scrambled, | Lo | Eggs |
| 33401310 | Egg substitute, omelet, scrambled, | Lo | Eggs |
| 33401400 | Egg substitute, omelet, scrambled, | Lo | Eggs |
| 33401420 | Egg substitute, omelet, scrambled, | Lo | Eggs |
| 33401500 | Egg substitute, omelet, scrambled, | Lo | Eggs |

| 33401510 | Egg substitute, omelet, scrambled, | Lo | Eggs |
| --- | --- | --- | --- |
| 33401600 | Egg substitute, omelet, scrambled, | Lo | Eggs |
| 33401610 | Egg substitute, omelet, scrambled, | Lo | Eggs |
| 33401620 | Egg substitute, omelet, scrambled, | Lo | Eggs |
| 35001000 | Scrambled eggs, sausage, hash br | Lo | Eggs |
| 35002000 | Scrambled eggs, bacon, home fried | Lo | Eggs |
| 41100990 | Beans, NFS | Lo | Plant-based Protein Foods |
| 41101000 | Beans, dry, cooked, NS as to type | Lo | Plant-based Protein Foods |
| 41101010 | Beans, from dried, NS as to type, fa | Lo | Plant-based Protein Foods |
| 41101020 | Beans, from dried, NS as to type, n | Lo | Plant-based Protein Foods |
| 41101050 | Beans, canned, drained, NS as to t | Lo | Plant-based Protein Foods |
| 41101060 | Beans, from canned, NS as to type | Lo | Plant-based Protein Foods |
| 41101070 | Beans, from canned, NS as to type | Lo | Plant-based Protein Foods |
| 41101080 | Beans, from fast food / restaurant, | Lo | Plant-based Protein Foods |
| 41101090 | White beans, NFS | Lo | Plant-based Protein Foods |
| 41101100 | White beans, dry, cooked, NS as to | Lo | Plant-based Protein Foods |
| 41101110 | White beans, from dried, fat added | Lo | Plant-based Protein Foods |
| 41101111 | White beans, dry, cooked, made wi | Lo | Plant-based Protein Foods |
| 41101112 | White beans, dry, cooked, made wi | Lo | Plant-based Protein Foods |
| 41101113 | White beans, dry, cooked, made wi | Lo | Plant-based Protein Foods |
| 41101120 | White beans, from dried, no added | Lo | Plant-based Protein Foods |
| 41101140 | White beans, from canned, fat add | Lo | Plant-based Protein Foods |
| 41101150 | White beans, canned, drained, mad | Lo | Plant-based Protein Foods |
| 41101180 | White beans, from canned, no add | Lo | Plant-based Protein Foods |
| 41101990 | Black beans, NFS | Lo | Plant-based Protein Foods |
| 41102000 | Black, brown, or Bayo beans, dry, c | Lo | Plant-based Protein Foods |
| 41102010 | Black beans, from dried, fat added | Lo | Plant-based Protein Foods |
| 41102011 | Black, brown, or Bayo beans, dry, c | Lo | Plant-based Protein Foods |
| 41102012 | Black, brown, or Bayo beans, dry, c | Lo | Plant-based Protein Foods |
| 41102013 | Black, brown, or Bayo beans, dry, c | Lo | Plant-based Protein Foods |
| 41102020 | Black beans, from dried, no added | Lo | Plant-based Protein Foods |
| 41102030 | Black, brown, or Bayo beans, cann | Lo | Plant-based Protein Foods |
| 41102040 | Black beans, from canned, fat adde | Lo | Plant-based Protein Foods |
| 41102050 | Black, brown, or Bayo beans, cann | Lo | Plant-based Protein Foods |
| 41102060 | Black, brown, or Bayo beans, cann | Lo | Plant-based Protein Foods |
| 41102080 | Black beans, from canned, no adde | Lo | Plant-based Protein Foods |
| 41102100 | Black, brown, or Bayo beans, cann | Lo | Plant-based Protein Foods |
| 41102110 | Black beans, from canned, reduced | Lo | Plant-based Protein Foods |
| 41102120 | Black, brown, or Bayo beans, cann | Lo | Plant-based Protein Foods |
| 41102150 | Black beans, from fast food / restau | Lo | Plant-based Protein Foods |
| 41102170 | Black beans with meat | Lo | Plant-based Protein Foods |
| 41102200 | Fava beans, dry, cooked, NS as to | Lo | Plant-based Protein Foods |
| 41102210 | Fava beans, cooked | Lo | Plant-based Protein Foods |
| 41102220 | Fava beans, dry, cooked, fat not ad | Lo | Plant-based Protein Foods |
| 41102260 | Fava beans, canned, drained, fat a | Lo | Plant-based Protein Foods |
| 41102990 | Lima beans, NFS | Lo | Plant-based Protein Foods |
| 41103000 | Lima beans, dry, cooked, NS as to | Lo | Plant-based Protein Foods |
| 41103010 | Lima beans, from dried | Lo | Plant-based Protein Foods |
| 41103011 | Lima beans, dry, cooked, made wit | Lo | Plant-based Protein Foods |
| 41103012 | Lima beans, dry, cooked, made wit | Lo | Plant-based Protein Foods |
| 41103013 | Lima beans, dry, cooked, made wit | Lo | Plant-based Protein Foods |

| 41103020 | Lima beans, dry, cooked, fat not ad | Lo | Plant-based Protein Foods |
| --- | --- | --- | --- |
| 41103050 | Pink beans, dry, cooked, NS as to f | Lo | Plant-based Protein Foods |
| 41103060 | Pink beans, dry, cooked, fat not ad | Lo | Plant-based Protein Foods |
| 41103070 | Pink beans, cooked | Lo | Plant-based Protein Foods |
| 41103090 | Pink beans, canned, drained, fat ad | Lo | Plant-based Protein Foods |
| 41103100 | Pink beans, canned, drained, fat no | Lo | Plant-based Protein Foods |
| 41103990 | Pinto beans, NFS | Lo | Plant-based Protein Foods |
| 41104000 | Pinto, calico, or red Mexican beans | Lo | Plant-based Protein Foods |
| 41104010 | Pinto beans, from dried, fat added | Lo | Plant-based Protein Foods |
| 41104011 | Pinto, calico, or red Mexican beans | Lo | Plant-based Protein Foods |
| 41104012 | Pinto, calico, or red Mexican beans | Lo | Plant-based Protein Foods |
| 41104013 | Pinto, calico, or red Mexican beans | Lo | Plant-based Protein Foods |
| 41104020 | Pinto beans, from dried, no added f | Lo | Plant-based Protein Foods |
| 41104030 | Pinto, calico, or red Mexican beans | Lo | Plant-based Protein Foods |
| 41104040 | Pinto beans, from canned, fat adde | Lo | Plant-based Protein Foods |
| 41104050 | Pinto, calico, or red Mexican beans | Lo | Plant-based Protein Foods |
| 41104060 | Pinto, calico, or red Mexican beans | Lo | Plant-based Protein Foods |
| 41104080 | Pinto beans, from canned, no adde | Lo | Plant-based Protein Foods |
| 41104110 | Pinto beans, from canned, reduced | Lo | Plant-based Protein Foods |
| 41104120 | Pinto, calico, or red Mexican beans | Lo | Plant-based Protein Foods |
| 41104200 | Pinto beans, from fast food / restau | Lo | Plant-based Protein Foods |
| 41104250 | Pinto beans with meat | Lo | Plant-based Protein Foods |
| 41105990 | Kidney beans, NFS | Lo | Plant-based Protein Foods |
| 41106000 | Red kidney beans, dry, cooked, NS | Lo | Plant-based Protein Foods |
| 41106010 | Kidney beans, from dried, fat added | Lo | Plant-based Protein Foods |
| 41106011 | Red kidney beans, dry, cooked, ma | Lo | Plant-based Protein Foods |
| 41106012 | Red kidney beans, dry, cooked, ma | Lo | Plant-based Protein Foods |
| 41106013 | Red kidney beans, dry, cooked, ma | Lo | Plant-based Protein Foods |
| 41106020 | Kidney beans, from dried, no added | Lo | Plant-based Protein Foods |
| 41106040 | Kidney beans, from canned, fat add | Lo | Plant-based Protein Foods |
| 41106050 | Red kidney beans, canned, drained | Lo | Plant-based Protein Foods |
| 41106080 | Kidney beans, from canned, no add | Lo | Plant-based Protein Foods |
| 41106100 | Red kidney beans, canned, drained | Lo | Plant-based Protein Foods |
| 41106110 | Kidney beans, from canned, reduce | Lo | Plant-based Protein Foods |
| 41106120 | Red kidney beans, canned, drained | Lo | Plant-based Protein Foods |
| 41106150 | Kidney beans, from fast food / resta | Lo | Plant-based Protein Foods |
| 41106170 | Kidney beans with meat | Lo | Plant-based Protein Foods |
| 41106500 | Yellow, canary, or Peruvian beans, | Lo | Plant-based Protein Foods |
| 41106510 | Peruvian beans, from dried | Lo | Plant-based Protein Foods |
| 41106511 | Yellow, canary, or Peruvian beans, | Lo | Plant-based Protein Foods |
| 41106520 | Yellow, canary, or Peruvian beans, | Lo | Plant-based Protein Foods |
| 41107000 | Soybeans, dry, cooked, fat not add | Lo | Plant-based Protein Foods |
| 41107010 | Soybeans, cooked | Lo | Plant-based Protein Foods |
| 41107020 | Soybeans, dry, cooked, NS as to fa | Lo | Plant-based Protein Foods |
| 41108000 | Mung beans, dry, cooked, fat not a | Lo | Plant-based Protein Foods |
| 41108010 | Mung beans, cooked | Lo | Plant-based Protein Foods |
| 41108020 | Mung beans, dry, cooked, NS as to | Lo | Plant-based Protein Foods |
| 41108030 | Mung beans, canned, drained, NS | Lo | Plant-based Protein Foods |
| 41109000 | Mungo beans, cooked, fat not adde | Lo | Plant-based Protein Foods |
| 41201010 | Baked beans | Lo | Plant-based Protein Foods |
| 41201020 | Baked beans, vegetarian | Lo | Plant-based Protein Foods |

| 41201050 | Baked beans from fast food / resta | Lo | Plant-based Protein Foods |
| --- | --- | --- | --- |
| 41202020 | Chili beans, barbecue beans, ranch | Lo | Plant-based Protein Foods |
| 41202500 | Beans and tomatoes, NS as to fat a | Lo | Plant-based Protein Foods |
| 41202505 | Beans and tomatoes, no added fat | Lo | Plant-based Protein Foods |
| 41202510 | Beans and tomatoes, fat added | Lo | Plant-based Protein Foods |
| 41203030 | Black bean salad | Lo | Mixed Dishes - Bean/Vegetable-based |
| 41204020 | Boston baked beans | Lo | Plant-based Protein Foods |
| 41205010 | Refried beans | Lo | Plant-based Protein Foods |
| 41205011 | Refried beans, made with oil | Lo | Plant-based Protein Foods |
| 41205012 | Refried beans, made with animal fa | Lo | Plant-based Protein Foods |
| 41205013 | Refried beans, made with margarin | Lo | Plant-based Protein Foods |
| 41205015 | Refried beans, fat not added in coo | Lo | Plant-based Protein Foods |
| 41205016 | Refried beans, NS as to fat added i | Lo | Plant-based Protein Foods |
| 41205017 | Refried beans, from fast food / rest | Lo | Plant-based Protein Foods |
| 41205020 | Refried beans with cheese | Lo | Plant-based Protein Foods |
| 41205030 | Refried beans with meat | Lo | Plant-based Protein Foods |
| 41205040 | Refried beans, from canned, reduc | Lo | Plant-based Protein Foods |
| 41205050 | Bean dip, made with refried beans | Lo | Condiments and Sauces |
| 41205055 | Layer dip | Lo | Condiments and Sauces |
| 41205070 | Hummus, plain | Lo | Condiments and Sauces |
| 41205075 | Hummus, flavored | Lo | Condiments and Sauces |
| 41205100 | Black bean sauce | Lo | Condiments and Sauces |
| 41206030 | Beans and franks | Lo | Plant-based Protein Foods |
| 41207030 | Beans, dry, cooked with ground be | Lo | Plant-based Protein Foods |
| 41208030 | Pork and beans | Lo | Plant-based Protein Foods |
| 41208100 | Beans with meat, NS as to type | Lo | Plant-based Protein Foods |
| 41209000 | Falafel | Lo | Mixed Dishes - Bean/Vegetable-based |
| 41210000 | Bean cake | Lo | Mixed Dishes - Bean/Vegetable-based |
| 41210090 | Stewed beans with pork, tomatoes, | Lo | Mixed Dishes - Bean/Vegetable-based |
| 41210100 | Stewed red beans, Puerto Rican st | Lo | Mixed Dishes - Bean/Vegetable-based |
| 41210150 | Stewed pink beans with white potat | Lo | Mixed Dishes - Bean/Vegetable-based |
| 41210190 | Stewed red beans with pig's feet an | Lo | Mixed Dishes - Bean/Vegetable-based |
| 41210200 | Black beans, Cuban style | Lo | Plant-based Protein Foods |
| 41221000 | Baked beans, reduced sodium | Lo | Plant-based Protein Foods |
| 41221010 | Baked beans, low sodium | Lo | Plant-based Protein Foods |
| 41221020 | Chili with beans, without meat | Lo | Mixed Dishes - Bean/Vegetable-based |
| 41300990 | Blackeyed peas, NFS | Lo | Plant-based Protein Foods |
| 41301000 | Cowpeas, dry, cooked, NS as to fat | Lo | Plant-based Protein Foods |
| 41301010 | Blackeyed peas, from dried | Lo | Plant-based Protein Foods |
| 41301020 | Cowpeas, dry, cooked, fat not adde | Lo | Plant-based Protein Foods |
| 41301990 | Chickpeas, NFS | Lo | Plant-based Protein Foods |
| 41302000 | Chickpeas, dry, cooked, NS as to f | Lo | Plant-based Protein Foods |
| 41302010 | Chickpeas, from dried, fat added | Lo | Plant-based Protein Foods |
| 41302011 | Chickpeas, dry, cooked, made with | Lo | Plant-based Protein Foods |
| 41302012 | Chickpeas, dry, cooked, made with | Lo | Plant-based Protein Foods |
| 41302020 | Chickpeas, from dried, no added fa | Lo | Plant-based Protein Foods |
| 41302030 | Chickpeas, canned, drained, NS as | Lo | Plant-based Protein Foods |
| 41302040 | Chickpeas, from canned, fat added | Lo | Plant-based Protein Foods |
| 41302050 | Chickpeas, canned, drained, made | Lo | Plant-based Protein Foods |
| 41302080 | Chickpeas, from canned, no added | Lo | Plant-based Protein Foods |
| 41302100 | Chickpeas, canned, drained, low so | Lo | Plant-based Protein Foods |

| 41302110 | Chickpeas, from canned, reduced s | Lo | Plant-based Protein Foods |
| --- | --- | --- | --- |
| 41303000 | Split peas, from dried, no added fat | Lo | Plant-based Protein Foods |
| 41303010 | Split peas, from dried, fat added | Lo | Plant-based Protein Foods |
| 41303011 | Green or yellow split peas, dry, coo | Lo | Plant-based Protein Foods |
| 41303012 | Green or yellow split peas, dry, coo | Lo | Plant-based Protein Foods |
| 41303013 | Green or yellow split peas, dry, coo | Lo | Plant-based Protein Foods |
| 41303020 | Green or yellow split peas, dry, coo | Lo | Plant-based Protein Foods |
| 41304000 | Wasabi peas | Lo | Plant-based Protein Foods |
| 41304130 | Cowpeas, dry, cooked with pork | Lo | Plant-based Protein Foods |
| 41304970 | Lentils, NFS | Lo | Plant-based Protein Foods |
| 41304980 | Lentils, dry, cooked, NS as to fat ad | Lo | Plant-based Protein Foods |
| 41304990 | Lentils, from dried, fat added | Lo | Plant-based Protein Foods |
| 41304991 | Lentils, dry, cooked, made with oil | Lo | Plant-based Protein Foods |
| 41304992 | Lentils, dry, cooked, made with ani | Lo | Plant-based Protein Foods |
| 41305000 | Lentils, from dried, no added fat | Lo | Plant-based Protein Foods |
| 41305020 | Lentils, from canned | Lo | Plant-based Protein Foods |
| 41306000 | Loaf, lentil | Lo | Plant-based Protein Foods |
| 41310100 | Stewed pigeon peas, Puerto Rican | Lo | Mixed Dishes - Bean/Vegetable-based |
| 41310150 | Stewed chickpeas, Puerto Rican st | Lo | Mixed Dishes - Bean/Vegetable-based |
| 41310160 | Stewed chickpeas, with potatoes, P | Lo | Mixed Dishes - Bean/Vegetable-based |
| 41310200 | Chickpeas stewed with pig's feet, P | Lo | Mixed Dishes - Bean/Vegetable-based |
| 41310220 | Fried chickpeas with bacon, Puerto | Lo | Plant-based Protein Foods |
| 41310900 | Bean chips | Lo | Savory Snacks |
| 41311000 | Papad, grilled or broiled | Lo | Plant-based Protein Foods |
| 41311020 | Sambar, vegetable stew | Lo | Mixed Dishes - Bean/Vegetable-based |
| 41311030 | Lentil curry | Lo | Mixed Dishes - Bean/Vegetable-based |
| 41311040 | Lentil curry with rice | Lo | Mixed Dishes - Bean/Vegetable-based |
| 41410010 | Soy nuts | Lo | Plant-based Protein Foods |
| 41410015 | Soy chips | Lo | Savory Snacks |
| 41420010 | Soybean curd | Lo | Plant-based Protein Foods |
| 41420020 | Edamame, cooked | Lo | Plant-based Protein Foods |
| 41420050 | Soybean curd cheese | Lo | Plant-based Protein Foods |
| 41420250 | Hoisin sauce | Lo | Condiments and Sauces |
| 41420300 | Soy sauce | Lo | Condiments and Sauces |
| 41420350 | Soy sauce, reduced sodium | Lo | Condiments and Sauces |
| 41420400 | Teriyaki sauce | Lo | Condiments and Sauces |
| 41420410 | Teriyaki sauce, reduced sodium | Lo | Condiments and Sauces |
| 41420450 | Worcestershire sauce | Lo | Condiments and Sauces |
| 41421010 | Soybean curd, deep fried | Lo | Plant-based Protein Foods |
| 41421020 | Soybean curd, breaded, fried | Lo | Plant-based Protein Foods |
| 41422010 | Soybean meal | Lo | Plant-based Protein Foods |
| 41425010 | Vermicelli, made from soybeans | Lo | Plant-based Protein Foods |
| 41430000 | Protein powder, NFS | Lo | Other |
| 41430010 | Protein supplement, powdered | Lo | Other |
| 41430200 | Meal replacement or supplement, s | Lo | Sweetened Beverages |
| 41430310 | Protein diet powder with soy and ca | Lo | Other |
| 41435110 | High protein bar, candy-like, soy an | Lo | Snack/Meal Bars |
| 41435120 | Zone Perfect Classic Crunch nutriti | Lo | Snack/Meal Bars |
| 41435300 | Balance Original Bar | Lo | Snack/Meal Bars |
| 41435500 | Clif Bar | Lo | Snack/Meal Bars |
| 41435700 | South Beach Living High Protein C | Lo | Snack/Meal Bars |

| 41435710 | South Beach Living Meal Replacem | Lo | Snack/Meal Bars |
| --- | --- | --- | --- |
| 41436000 | Nutritional supplement for people w | Lo | Sweetened Beverages |
| 41440000 | Textured vegetable protein, dry | Lo | Plant-based Protein Foods |
| 41440010 | Ensure liquid nutrition | Lo | Sweetened Beverages |
| 41440020 | Ensure with fiber, liquid | Lo | Sweetened Beverages |
| 41440050 | Ensure Plus liquid nutrition | Lo | Sweetened Beverages |
| 41440100 | Meal replacement or supplement, li | Lo | Sweetened Beverages |
| 41480000 | Tofu, frozen dessert, flavors other t | Lo | Other Desserts |
| 41480010 | Tofu, frozen dessert, chocolate | Lo | Other Desserts |
| 41480020 | Frozen dessert, non-dairy | Lo | Plant-based Protein Foods |
| 41601010 | Bean soup, NFS | Lo | Mixed Dishes - Soups |
| 41601020 | Bean with bacon or ham soup, can | Lo | Mixed Dishes - Soups |
| 41601030 | Black bean soup, home recipe, can | Lo | Mixed Dishes - Soups |
| 41601040 | Lima bean soup, home recipe, can | Lo | Mixed Dishes - Soups |
| 41601060 | Bean soup, with macaroni and mea | Lo | Mixed Dishes - Soups |
| 41601070 | Soybean soup, miso broth | Lo | Mixed Dishes - Soups |
| 41601080 | Pinto bean soup, home recipe, can | Lo | Mixed Dishes - Soups |
| 41601090 | Bean soup, with macaroni, home re | Lo | Mixed Dishes - Soups |
| 41601100 | Portuguese bean soup, home recip | Lo | Mixed Dishes - Soups |
| 41601110 | Bean and ham soup, chunky style, | Lo | Mixed Dishes - Soups |
| 41601120 | Bean soup with vegetables, rice, an | Lo | Mixed Dishes - Soups |
| 41601130 | Bean soup, mixed beans, home rec | Lo | Mixed Dishes - Soups |
| 41601140 | Bean soup, home recipe | Lo | Mixed Dishes - Soups |
| 41601160 | Bean and ham soup, canned, reduc | Lo | Mixed Dishes - Soups |
| 41601170 | Bean and rice soup | Lo | Mixed Dishes - Soups |
| 41601180 | Bean and ham soup, home recipe | Lo | Mixed Dishes - Soups |
| 41601200 | Liquid from stewed kidney beans, P | Lo | Mixed Dishes - Soups |
| 41602010 | Pea and ham soup, chunky style, c | Lo | Mixed Dishes - Soups |
| 41602020 | Garbanzo bean or chickpea soup, h | Lo | Mixed Dishes - Soups |
| 41602030 | Split pea and ham soup | Lo | Mixed Dishes - Soups |
| 41602050 | Split pea soup | Lo | Mixed Dishes - Soups |
| 41602070 | Split pea soup, canned, reduced so | Lo | Mixed Dishes - Soups |
| 41602090 | Split pea and ham soup, canned, re | Lo | Mixed Dishes - Soups |
| 41603010 | Lentil soup, home recipe, canned, o | Lo | Mixed Dishes - Soups |
| 41610100 | White bean soup, Puerto Rican styl | Lo | Mixed Dishes - Soups |
| 41810200 | Bacon strip, meatless | Lo | Plant-based Protein Foods |
| 41810250 | Bacon bits | Lo | Cured Meats/Poultry |
| 41810400 | Breakfast link, pattie, or slice, meat | Lo | Plant-based Protein Foods |
| 41810600 | Chicken, meatless, NFS | Lo | Plant-based Protein Foods |
| 41810610 | Chicken, meatless, breaded, fried | Lo | Plant-based Protein Foods |
| 41811200 | Fish stick, meatless | Lo | Plant-based Protein Foods |
| 41811400 | Frankfurter or hot dog, meatless | Lo | Plant-based Protein Foods |
| 41811600 | Luncheon slice, meatless-beef, chi | Lo | Plant-based Protein Foods |
| 41811800 | Meatball, meatless | Lo | Plant-based Protein Foods |
| 41811890 | Vegetarian burger or patty, meatles | Lo | Plant-based Protein Foods |
| 41811900 | Soyburger, meatless, no bun | Lo | Plant-based Protein Foods |
| 41811910 | Vegetable burger or patty, meatless | Lo | Plant-based Protein Foods |
| 41811950 | Swiss steak, with gravy, meatless | Lo | Mixed Dishes - Bean/Vegetable-based |
| 41812000 | Sandwich spread, meat substitute t | Lo | Plant-based Protein Foods |
| 41812400 | Vegetarian pot pie | Lo | Mixed Dishes - Bean/Vegetable-based |
| 41812450 | Vegetarian chili, made with meat su | Lo | Mixed Dishes - Bean/Vegetable-based |

| 41812500 | Tofu and vegetables including carro | Lo | Mixed Dishes - Asian |
| --- | --- | --- | --- |
| 41812510 | Tofu and vegetables excluding carr | Lo | Mixed Dishes - Asian |
| 41812600 | Vegetarian, fillet | Lo | Plant-based Protein Foods |
| 41812800 | Vegetarian stew | Lo | Mixed Dishes - Bean/Vegetable-based |
| 41812850 | Vegetarian stroganoff | Lo | Mixed Dishes - Bean/Vegetable-based |
| 41812900 | Vegetarian meat loaf | Lo | Mixed Dishes - Bean/Vegetable-based |
| 41901020 | Soyburger, meatless, with cheese o | Lo | Plant-based Protein Foods |
| 42100050 | Nuts, NFS | Lo | Plant-based Protein Foods |
| 42100100 | Almonds, NFS | Lo | Plant-based Protein Foods |
| 42101000 | Almonds, unroasted | Lo | Plant-based Protein Foods |
| 42101100 | Almonds, roasted | Lo | Plant-based Protein Foods |
| 42101110 | Almonds, salted | Lo | Plant-based Protein Foods |
| 42101120 | Almonds, lightly salted | Lo | Plant-based Protein Foods |
| 42101130 | Almonds, unsalted | Lo | Plant-based Protein Foods |
| 42101200 | Almonds, dry roasted, salted | Lo | Plant-based Protein Foods |
| 42101210 | Almonds, dry roasted, without salt | Lo | Plant-based Protein Foods |
| 42101300 | Almonds, flavored | Lo | Plant-based Protein Foods |
| 42101350 | Almonds, honey roasted | Lo | Plant-based Protein Foods |
| 42102000 | Brazil nuts | Lo | Plant-based Protein Foods |
| 42104000 | Cashews, NFS | Lo | Plant-based Protein Foods |
| 42104050 | Cashews, unroasted | Lo | Plant-based Protein Foods |
| 42104100 | Cashews, salted | Lo | Plant-based Protein Foods |
| 42104105 | Cashews, lightly salted | Lo | Plant-based Protein Foods |
| 42104110 | Cashews, unsalted | Lo | Plant-based Protein Foods |
| 42104200 | Cashew nuts, dry roasted, salted | Lo | Plant-based Protein Foods |
| 42104205 | Cashew nuts, dry roasted, without s | Lo | Plant-based Protein Foods |
| 42104500 | Cashews, honey roasted | Lo | Plant-based Protein Foods |
| 42105000 | Chestnuts | Lo | Plant-based Protein Foods |
| 42106000 | Coconut, fresh | Lo | Plant-based Protein Foods |
| 42106020 | Coconut, packaged | Lo | Plant-based Protein Foods |
| 42107000 | Hazelnuts | Lo | Plant-based Protein Foods |
| 42109000 | Macadamia nuts, unroasted | Lo | Plant-based Protein Foods |
| 42109100 | Macadamia nuts | Lo | Plant-based Protein Foods |
| 42109105 | Macadamia nuts, roasted, without s | Lo | Plant-based Protein Foods |
| 42110000 | Mixed nuts, NFS | Lo | Plant-based Protein Foods |
| 42110015 | Mixed nuts, salted | Lo | Plant-based Protein Foods |
| 42110020 | Mixed nuts, without salt | Lo | Plant-based Protein Foods |
| 42110050 | Mixed nuts, unroasted | Lo | Plant-based Protein Foods |
| 42110100 | Mixed nuts, with peanuts, salted | Lo | Plant-based Protein Foods |
| 42110110 | Mixed nuts, with peanuts, lightly sal | Lo | Plant-based Protein Foods |
| 42110120 | Mixed nuts, with peanuts, unsalted | Lo | Plant-based Protein Foods |
| 42110150 | Mixed nuts, without peanuts, salted | Lo | Plant-based Protein Foods |
| 42110160 | Mixed nuts, without peanuts, unsalt | Lo | Plant-based Protein Foods |
| 42110200 | Mixed nuts, dry roasted | Lo | Plant-based Protein Foods |
| 42110300 | Mixed nuts, honey roasted | Lo | Plant-based Protein Foods |
| 42110500 | Mixed nuts, in shell | Lo | Plant-based Protein Foods |
| 42111000 | Peanuts, NFS | Lo | Plant-based Protein Foods |
| 42111010 | Peanuts, in shell, NFS (shell not ea | Lo | Plant-based Protein Foods |
| 42111030 | Peanuts, boiled | Lo | Plant-based Protein Foods |
| 42111040 | Peanuts, unroasted | Lo | Plant-based Protein Foods |
| 42111100 | Peanuts, roasted, salted | Lo | Plant-based Protein Foods |

| 42111110 | Peanuts, roasted, unsalted | Lo | Plant-based Protein Foods |
| --- | --- | --- | --- |
| 42111200 | Peanuts, dry roasted, salted | Lo | Plant-based Protein Foods |
| 42111205 | Peanuts, dry roasted, lightly salted | Lo | Plant-based Protein Foods |
| 42111210 | Peanuts, dry roasted, unsalted | Lo | Plant-based Protein Foods |
| 42111500 | Peanuts, honey roasted | Lo | Plant-based Protein Foods |
| 42112000 | Pecans, NFS | Lo | Plant-based Protein Foods |
| 42112100 | Pecans, unroasted | Lo | Plant-based Protein Foods |
| 42112200 | Pecans, salted | Lo | Plant-based Protein Foods |
| 42112210 | Pecans, unsalted | Lo | Plant-based Protein Foods |
| 42112300 | Pecans, honey roasted | Lo | Plant-based Protein Foods |
| 42113000 | Pine nuts | Lo | Plant-based Protein Foods |
| 42114130 | Pistachio nuts, NFS | Lo | Plant-based Protein Foods |
| 42114140 | Pistachio nuts, salted | Lo | Plant-based Protein Foods |
| 42114142 | Pistachio nuts, lightly salted | Lo | Plant-based Protein Foods |
| 42114145 | Pistachio nuts, unsalted | Lo | Plant-based Protein Foods |
| 42116000 | Walnuts, excluding honey roasted | Lo | Plant-based Protein Foods |
| 42116050 | Walnuts | Lo | Plant-based Protein Foods |
| 42116055 | Walnuts, roasted, without salt | Lo | Plant-based Protein Foods |
| 42116100 | Walnuts, honey roasted | Lo | Plant-based Protein Foods |
| 42200500 | Almond butter | Lo | Plant-based Protein Foods |
| 42200510 | Almond butter, lower sodium | Lo | Plant-based Protein Foods |
| 42200600 | Almond paste | Lo | Plant-based Protein Foods |
| 42201000 | Cashew butter | Lo | Plant-based Protein Foods |
| 42202000 | Peanut butter | Lo | Plant-based Protein Foods |
| 42202010 | Peanut butter, lower sodium | Lo | Plant-based Protein Foods |
| 42202100 | Peanut butter, lower sodium and lo | Lo | Plant-based Protein Foods |
| 42202130 | Peanut butter, lower sugar | Lo | Plant-based Protein Foods |
| 42202150 | Peanut butter, reduced fat | Lo | Plant-based Protein Foods |
| 42202200 | Peanut butter, vitamin and mineral | Lo | Plant-based Protein Foods |
| 42203000 | Peanut butter and jelly | Lo | Plant-based Protein Foods |
| 42203100 | Peanut butter and chocolate spread | Lo | Plant-based Protein Foods |
| 42203200 | Soy nut butter | Lo | Plant-based Protein Foods |
| 42204050 | Peanut sauce | Lo | Condiments and Sauces |
| 42204100 | Gravy, vegetarian | Lo | Condiments and Sauces |
| 42301010 | Peanut butter sandwich, NFS | Lo | Mixed Dishes - Sandwiches (single code) |
| 42301015 | Peanut butter sandwich, with regula | Lo | Mixed Dishes - Sandwiches (single code) |
| 42301020 | Peanut butter sandwich, with regula | Lo | Mixed Dishes - Sandwiches (single code) |
| 42301025 | Peanut butter sandwich, with regula | Lo | Mixed Dishes - Sandwiches (single code) |
| 42301115 | Peanut butter sandwich, with reduc | Lo | Mixed Dishes - Sandwiches (single code) |
| 42301120 | Peanut butter sandwich, with reduc | Lo | Mixed Dishes - Sandwiches (single code) |
| 42301125 | Peanut butter sandwich, with reduc | Lo | Mixed Dishes - Sandwiches (single code) |
| 42302010 | Peanut butter and jelly sandwich, N | Lo | Mixed Dishes - Sandwiches (single code) |
| 42302015 | Peanut butter and jelly sandwich, w | Lo | Mixed Dishes - Sandwiches (single code) |
| 42302020 | Peanut butter and jelly sandwich, w | Lo | Mixed Dishes - Sandwiches (single code) |
| 42302025 | Peanut butter and jelly sandwich, w | Lo | Mixed Dishes - Sandwiches (single code) |
| 42302055 | Peanut butter and jelly sandwich, w | Lo | Mixed Dishes - Sandwiches (single code) |
| 42302060 | Peanut butter and jelly sandwich, w | Lo | Mixed Dishes - Sandwiches (single code) |
| 42302065 | Peanut butter and jelly sandwich, w | Lo | Mixed Dishes - Sandwiches (single code) |
| 42302105 | Peanut butter and jelly sandwich, w | Lo | Mixed Dishes - Sandwiches (single code) |
| 42302110 | Peanut butter and jelly sandwich, w | Lo | Mixed Dishes - Sandwiches (single code) |
| 42302115 | Peanut butter and jelly sandwich, w | Lo | Mixed Dishes - Sandwiches (single code) |

| 42302155 | Peanut butter and jelly sandwich, w | Lo | Mixed Dishes - Sandwiches (single code) |
| --- | --- | --- | --- |
| 42302160 | Peanut butter and jelly sandwich, w | Lo | Mixed Dishes - Sandwiches (single code) |
| 42302165 | Peanut butter and jelly sandwich, w | Lo | Mixed Dishes - Sandwiches (single code) |
| 42303100 | Peanut butter and jelly sandwich, fr | Lo | Mixed Dishes - Sandwiches (single code) |
| 42401010 | Coconut milk, used in cooking | Lo | Dairy Drinks and Substitutes |
| 42401100 | Yogurt, coconut milk | Lo | Yogurt |
| 42402010 | Coconut cream, canned, sweetene | Lo | Dairy Drinks and Substitutes |
| 42403010 | Coconut water, unsweetened | Lo | Sweetened Beverages |
| 42404010 | Coconut water, sweetened | Lo | Sweetened Beverages |
| 42502000 | Nut mixture with seeds | Lo | Plant-based Protein Foods |
| 42502100 | Trail mix with pretzels, cereal, or gr | Lo | Plant-based Protein Foods |
| 43101000 | Pumpkin and/or squash seeds, hull | Lo | Plant-based Protein Foods |
| 43101050 | Pumpkin seeds, NFS | Lo | Plant-based Protein Foods |
| 43101100 | Pumpkin seeds, salted | Lo | Plant-based Protein Foods |
| 43101150 | Pumpkin seeds, unsalted | Lo | Plant-based Protein Foods |
| 43102000 | Sunflower seeds, plain, unsalted | Lo | Plant-based Protein Foods |
| 43102100 | Sunflower seeds, plain, salted | Lo | Plant-based Protein Foods |
| 43102110 | Sunflower seeds, hulled, roasted, w | Lo | Plant-based Protein Foods |
| 43102200 | Sunflower seeds, hulled, dry roaste | Lo | Plant-based Protein Foods |
| 43102300 | Sunflower seeds, flavored | Lo | Plant-based Protein Foods |
| 43102400 | Sunflower seeds, NFS | Lo | Plant-based Protein Foods |
| 43103000 | Sesame seeds | Lo | Plant-based Protein Foods |
| 43103050 | Sesame seeds, whole seed | Lo | Plant-based Protein Foods |
| 43103100 | Sesame sauce | Lo | Condiments and Sauces |
| 43104000 | Flax seeds | Lo | Plant-based Protein Foods |
| 43105200 | Psyllium seed, husks | Lo | Plant-based Protein Foods |
| 43107000 | Mixed seeds | Lo | Plant-based Protein Foods |
| 43108010 | Chia seeds | Lo | Plant-based Protein Foods |
| 44201000 | Carob chips | Lo | Candy |
| 44202000 | Carob syrup | Lo | Sugars |
| 50010000 | Flour, white | Lo | Other |
| 51000100 | Bread, NS as to major flour | Lo | Breads, Rolls, Tortillas |
| 51000110 | Bread, NS as to major flour, toaste | Lo | Breads, Rolls, Tortillas |
| 51000180 | Bread, made from home recipe or p | Lo | Breads, Rolls, Tortillas |
| 51000190 | Bread, made from home recipe or p | Lo | Breads, Rolls, Tortillas |
| 51000200 | Roll, NS as to major flour | Lo | Breads, Rolls, Tortillas |
| 51000230 | Roll, NS as to major flour, toasted | Lo | Breads, Rolls, Tortillas |
| 51000250 | Roll, made from home recipe or pu | Lo | Breads, Rolls, Tortillas |
| 51000300 | Roll, hard, NS as to major flour | Lo | Breads, Rolls, Tortillas |
| 51000400 | Roll, bran, NS as to type of bran | Lo | Breads, Rolls, Tortillas |
| 51101000 | Bread, white | Lo | Breads, Rolls, Tortillas |
| 51101010 | Bread, white, toasted | Lo | Breads, Rolls, Tortillas |
| 51101050 | Bread, white, made from home reci | Lo | Breads, Rolls, Tortillas |
| 51101060 | Bread, white, made from home reci | Lo | Breads, Rolls, Tortillas |
| 51102010 | Bread, white with whole wheat swirl | Lo | Breads, Rolls, Tortillas |
| 51102020 | Bread, white with whole wheat swirl | Lo | Breads, Rolls, Tortillas |
| 51105010 | Bread, Cuban | Lo | Breads, Rolls, Tortillas |
| 51105040 | Bread, Cuban, toasted | Lo | Breads, Rolls, Tortillas |
| 51106010 | Bread, native, water, Puerto Rican | Lo | Breads, Rolls, Tortillas |
| 51107010 | Bread, French or Vienna | Lo | Breads, Rolls, Tortillas |
| 51107040 | Bread, French or Vienna, toasted | Lo | Breads, Rolls, Tortillas |

| 51108010 | Focaccia, Italian flatbread, plain | Lo | Breads, Rolls, Tortillas |
| --- | --- | --- | --- |
| 51108100 | Naan, Indian flatbread | Lo | Breads, Rolls, Tortillas |
| 51109010 | Bread, Italian, Grecian, Armenian | Lo | Breads, Rolls, Tortillas |
| 51109040 | Bread, Italian, Grecian, Armenian, t | Lo | Breads, Rolls, Tortillas |
| 51109100 | Bread, pita | Lo | Breads, Rolls, Tortillas |
| 51109110 | Bread, pita, toasted | Lo | Breads, Rolls, Tortillas |
| 51109150 | Bread, pita with fruit | Lo | Breads, Rolls, Tortillas |
| 51110010 | Bread, batter | Lo | Breads, Rolls, Tortillas |
| 51111010 | Bread, cheese | Lo | Breads, Rolls, Tortillas |
| 51111040 | Bread, cheese, toasted | Lo | Breads, Rolls, Tortillas |
| 51113010 | Bread, cinnamon | Lo | Breads, Rolls, Tortillas |
| 51113100 | Bread, cinnamon, toasted | Lo | Breads, Rolls, Tortillas |
| 51115010 | Bread, cornmeal and molasses | Lo | Breads, Rolls, Tortillas |
| 51115020 | Bread, cornmeal and molasses, toa | Lo | Breads, Rolls, Tortillas |
| 51119010 | Bread, egg, Challah | Lo | Breads, Rolls, Tortillas |
| 51119040 | Bread, egg, Challah, toasted | Lo | Breads, Rolls, Tortillas |
| 51119100 | Bread, lowfat, 98% fat free | Lo | Breads, Rolls, Tortillas |
| 51121010 | Bread, garlic | Lo | Breads, Rolls, Tortillas |
| 51121015 | Garlic bread, NFS | Lo | Breads, Rolls, Tortillas |
| 51121025 | Garlic bread, from fast food / restau | Lo | Breads, Rolls, Tortillas |
| 51121035 | Garlic bread, from frozen | Lo | Breads, Rolls, Tortillas |
| 51121040 | Bread, garlic, toasted | Lo | Breads, Rolls, Tortillas |
| 51121045 | Garlic bread, with parmesan chees | Lo | Breads, Rolls, Tortillas |
| 51121055 | Garlic bread, with parmesan chees | Lo | Breads, Rolls, Tortillas |
| 51121065 | Garlic bread, with melted cheese, fr | Lo | Breads, Rolls, Tortillas |
| 51121075 | Garlic bread, with melted cheese, fr | Lo | Breads, Rolls, Tortillas |
| 51121110 | Bread, onion | Lo | Breads, Rolls, Tortillas |
| 51122000 | Bread, reduced calorie and/or high | Lo | Breads, Rolls, Tortillas |
| 51122010 | Bread, reduced calorie and/or high | Lo | Breads, Rolls, Tortillas |
| 51122050 | Bread, reduced calorie and/or high | Lo | Breads, Rolls, Tortillas |
| 51122100 | Bread, reduced calorie and/or high | Lo | Breads, Rolls, Tortillas |
| 51122110 | Bread, reduced calorie and/or high | Lo | Breads, Rolls, Tortillas |
| 51122300 | Bread, white, special formula, adde | Lo | Breads, Rolls, Tortillas |
| 51123010 | Bread, high protein | Lo | Breads, Rolls, Tortillas |
| 51123020 | Bread, high protein, toasted | Lo | Breads, Rolls, Tortillas |
| 51126010 | Bread, milk and honey | Lo | Breads, Rolls, Tortillas |
| 51126020 | Bread, milk and honey, toasted | Lo | Breads, Rolls, Tortillas |
| 51127010 | Bread, potato | Lo | Breads, Rolls, Tortillas |
| 51127020 | Bread, potato, toasted | Lo | Breads, Rolls, Tortillas |
| 51129010 | Bread, raisin | Lo | Breads, Rolls, Tortillas |
| 51129020 | Bread, raisin, toasted | Lo | Breads, Rolls, Tortillas |
| 51133010 | Bread, sour dough | Lo | Breads, Rolls, Tortillas |
| 51133020 | Bread, sour dough, toasted | Lo | Breads, Rolls, Tortillas |
| 51134000 | Bread, sweet potato | Lo | Breads, Rolls, Tortillas |
| 51135000 | Bread, vegetable | Lo | Breads, Rolls, Tortillas |
| 51135010 | Bread, vegetable, toasted | Lo | Breads, Rolls, Tortillas |
| 51136000 | Bruschetta | Lo | Mixed Dishes - Sandwiches (single code) |
| 51140100 | Bread, dough, fried | Lo | Breads, Rolls, Tortillas |
| 51150000 | Roll, white, soft | Lo | Breads, Rolls, Tortillas |
| 51150100 | Roll, white, soft, toasted | Lo | Breads, Rolls, Tortillas |
| 51151060 | Roll, white, soft, made from home r | Lo | Breads, Rolls, Tortillas |

| 51152000 | Roll, white, soft, reduced calorie an | Lo | Breads, Rolls, Tortillas |
| --- | --- | --- | --- |
| 51153000 | Roll, white, hard | Lo | Breads, Rolls, Tortillas |
| 51153010 | Roll, white, hard, toasted | Lo | Breads, Rolls, Tortillas |
| 51154010 | Roll, white, hot dog bun | Lo | Breads, Rolls, Tortillas |
| 51154100 | Roll, white, hamburger bun | Lo | Breads, Rolls, Tortillas |
| 51154510 | Roll, diet | Lo | Breads, Rolls, Tortillas |
| 51154550 | Roll, egg bread | Lo | Breads, Rolls, Tortillas |
| 51154600 | Roll, cheese | Lo | Breads, Rolls, Tortillas |
| 51155000 | Roll, French or Vienna | Lo | Breads, Rolls, Tortillas |
| 51155010 | Roll, French or Vienna, toasted | Lo | Breads, Rolls, Tortillas |
| 51156500 | Roll, garlic | Lo | Breads, Rolls, Tortillas |
| 51157000 | Roll, white, hoagie, submarine | Lo | Breads, Rolls, Tortillas |
| 51158100 | Roll, Mexican, bolillo | Lo | Breads, Rolls, Tortillas |
| 51159000 | Roll, sour dough | Lo | Breads, Rolls, Tortillas |
| 51160000 | Roll, sweet, no frosting | Lo | Sweet Bakery Products |
| 51160010 | Roll, sweet, toasted | Lo | Sweet Bakery Products |
| 51160100 | Roll, sweet, cinnamon bun, no frost | Lo | Sweet Bakery Products |
| 51160110 | Roll, sweet, cinnamon bun, frosted | Lo | Sweet Bakery Products |
| 51161000 | Pan Dulce, with fruit, no frosting | Lo | Sweet Bakery Products |
| 51161020 | Roll, sweet, with fruit, frosted | Lo | Sweet Bakery Products |
| 51161030 | Roll, sweet, with fruit, frosted, diet | Lo | Sweet Bakery Products |
| 51161050 | Roll, sweet, frosted | Lo | Sweet Bakery Products |
| 51161070 | Roll, sweet, with fruit, frosted, fat fr | Lo | Sweet Bakery Products |
| 51161100 | Roll, sweet, with fruit and nuts, no f | Lo | Sweet Bakery Products |
| 51161150 | Roll, sweet, with fruit and nuts, fros | Lo | Sweet Bakery Products |
| 51161200 | Roll, sweet, with nuts, no frosting | Lo | Sweet Bakery Products |
| 51161250 | Pan Dulce, no topping | Lo | Sweet Bakery Products |
| 51161260 | Roll, sweet, crumb topping, Mexica | Lo | Sweet Bakery Products |
| 51161270 | Pan Dulce, with sugar topping | Lo | Sweet Bakery Products |
| 51161280 | Pan Dulce, with raisins and icing | Lo | Sweet Bakery Products |
| 51165000 | Coffee cake, yeast type | Lo | Sweet Bakery Products |
| 51165060 | Coffee cake, yeast type, made from | Lo | Sweet Bakery Products |
| 51165100 | Coffee cake, yeast type, fat free, ch | Lo | Sweet Bakery Products |
| 51166000 | Croissant | Lo | Sweet Bakery Products |
| 51166100 | Croissant, cheese | Lo | Sweet Bakery Products |
| 51166200 | Croissant, chocolate | Lo | Sweet Bakery Products |
| 51166500 | Croissant, fruit | Lo | Sweet Bakery Products |
| 51167000 | Brioche | Lo | Sweet Bakery Products |
| 51168000 | Bread, Spanish coffee | Lo | Sweet Bakery Products |
| 51180010 | Bagel | Lo | Breads, Rolls, Tortillas |
| 51180020 | Bagel, toasted | Lo | Breads, Rolls, Tortillas |
| 51180030 | Bagel, with raisins | Lo | Breads, Rolls, Tortillas |
| 51180040 | Bagel, with raisins, toasted | Lo | Breads, Rolls, Tortillas |
| 51180080 | Bagel, with fruit other than raisins | Lo | Breads, Rolls, Tortillas |
| 51180090 | Bagel, with fruit other than raisins, t | Lo | Breads, Rolls, Tortillas |
| 51182010 | Bread stuffing | Lo | Mixed Dishes - Grain-based |
| 51182020 | Bread stuffing made with egg | Lo | Mixed Dishes - Grain-based |
| 51183990 | Breadsticks, NFS | Lo | Breads, Rolls, Tortillas |
| 51184000 | Breadsticks, hard, NFS | Lo | Crackers |
| 51184010 | Bread stick, soft | Lo | Breads, Rolls, Tortillas |
| 51184020 | Bread stick, NS as to hard or soft | Lo | Breads, Rolls, Tortillas |

| 51184030 | Bread stick, soft, prepared with garl | Lo | Breads, Rolls, Tortillas |
| --- | --- | --- | --- |
| 51184100 | Breadsticks, hard, reduced sodium | Lo | Crackers |
| 51184200 | Breadsticks, soft, NFS | Lo | Breads, Rolls, Tortillas |
| 51184210 | Breadsticks, soft, from fast food / re | Lo | Breads, Rolls, Tortillas |
| 51184220 | Breadsticks, soft, from frozen | Lo | Breads, Rolls, Tortillas |
| 51184230 | Breadsticks, soft, with parmesan ch | Lo | Breads, Rolls, Tortillas |
| 51184240 | Breadsticks, soft, with parmesan ch | Lo | Breads, Rolls, Tortillas |
| 51184250 | Breadsticks, soft, topped with melte | Lo | Breads, Rolls, Tortillas |
| 51184260 | Breadsticks, soft, stuffed with melte | Lo | Breads, Rolls, Tortillas |
| 51185000 | Croutons | Lo | Crackers |
| 51186010 | Muffin, English | Lo | Breads, Rolls, Tortillas |
| 51186020 | Muffin, English, toasted | Lo | Breads, Rolls, Tortillas |
| 51186100 | Muffin, English, with raisins | Lo | Breads, Rolls, Tortillas |
| 51186120 | Muffin, English, with raisins, toasted | Lo | Breads, Rolls, Tortillas |
| 51186160 | Muffin, English, with fruit other than | Lo | Breads, Rolls, Tortillas |
| 51187000 | Melba toast | Lo | Crackers |
| 51187020 | Anisette toast | Lo | Sweet Bakery Products |
| 51188100 | Pannetone | Lo | Sweet Bakery Products |
| 51188500 | Zwieback toast | Lo | Crackers |
| 51201010 | Bread, whole wheat, 100% | Lo | Breads, Rolls, Tortillas |
| 51201020 | Bread, whole wheat, 100%, toasted | Lo | Breads, Rolls, Tortillas |
| 51201060 | Bread, whole wheat, 100%, made f | Lo | Breads, Rolls, Tortillas |
| 51201070 | Bread, whole wheat, 100%, made f | Lo | Breads, Rolls, Tortillas |
| 51201110 | Bread, whole wheat, 100%, with rai | Lo | Breads, Rolls, Tortillas |
| 51201120 | Bread, whole wheat, 100%, with rai | Lo | Breads, Rolls, Tortillas |
| 51201150 | Bread, pita, whole wheat, 100% | Lo | Breads, Rolls, Tortillas |
| 51202000 | Muffin, English, whole wheat, 100% | Lo | Breads, Rolls, Tortillas |
| 51204010 | Bread, wheat germ | Lo | Breads, Rolls, Tortillas |
| 51207010 | Bread, sprouted wheat | Lo | Breads, Rolls, Tortillas |
| 51207020 | Bread, sprouted wheat, toasted | Lo | Breads, Rolls, Tortillas |
| 51208000 | Bagel, whole wheat, 100% | Lo | Breads, Rolls, Tortillas |
| 51208010 | Bagel, whole wheat, 100%, toasted | Lo | Breads, Rolls, Tortillas |
| 51208100 | Bagel, whole wheat, 100%, with rai | Lo | Breads, Rolls, Tortillas |
| 51208110 | Bagel, whole wheat, 100%, with rai | Lo | Breads, Rolls, Tortillas |
| 51220000 | Roll, whole wheat, 100% | Lo | Breads, Rolls, Tortillas |
| 51300050 | Bread, whole grain white | Lo | Breads, Rolls, Tortillas |
| 51300060 | Bread, whole grain white, toasted | Lo | Breads, Rolls, Tortillas |
| 51300100 | Bagel, whole grain white | Lo | Breads, Rolls, Tortillas |
| 51300110 | Bread, whole wheat | Lo | Breads, Rolls, Tortillas |
| 51300120 | Bread, whole wheat, toasted | Lo | Breads, Rolls, Tortillas |
| 51300140 | Bread, whole wheat, made from ho | Lo | Breads, Rolls, Tortillas |
| 51300150 | Bread, whole wheat, made from ho | Lo | Breads, Rolls, Tortillas |
| 51300175 | Bread, chappatti or roti, wheat | Lo | Breads, Rolls, Tortillas |
| 51300180 | Bread, puri, wheat | Lo | Breads, Rolls, Tortillas |
| 51300185 | Bread, paratha, wheat | Lo | Breads, Rolls, Tortillas |
| 51300210 | Bread, whole wheat, with raisins | Lo | Breads, Rolls, Tortillas |
| 51300220 | Bread, whole wheat, with raisins, to | Lo | Breads, Rolls, Tortillas |
| 51300300 | Bread, sprouted wheat | Lo | Breads, Rolls, Tortillas |
| 51300310 | Bread, sprouted wheat, toasted | Lo | Breads, Rolls, Tortillas |
| 51301010 | Bread, wheat or cracked wheat | Lo | Breads, Rolls, Tortillas |
| 51301020 | Bread, wheat or cracked wheat, toa | Lo | Breads, Rolls, Tortillas |

| 51301040 | Bread, wheat or cracked wheat, ma | Lo | Breads, Rolls, Tortillas |
| --- | --- | --- | --- |
| 51301050 | Bread, wheat or cracked wheat, ma | Lo | Breads, Rolls, Tortillas |
| 51301120 | Bread, wheat or cracked wheat, wit | Lo | Breads, Rolls, Tortillas |
| 51301130 | Bread, wheat or cracked wheat, wit | Lo | Breads, Rolls, Tortillas |
| 51301510 | Bread, wheat or cracked wheat, red | Lo | Breads, Rolls, Tortillas |
| 51301520 | Bread, wheat or cracked wheat, red | Lo | Breads, Rolls, Tortillas |
| 51301540 | Bread, French or Vienna, whole wh | Lo | Breads, Rolls, Tortillas |
| 51301550 | Bread, French or Vienna, whole wh | Lo | Breads, Rolls, Tortillas |
| 51301600 | Bread, pita, whole wheat | Lo | Breads, Rolls, Tortillas |
| 51301610 | Bread, pita, whole wheat, toasted | Lo | Breads, Rolls, Tortillas |
| 51301620 | Bread, pita, wheat or cracked whea | Lo | Breads, Rolls, Tortillas |
| 51301630 | Bread, pita, wheat or cracked whea | Lo | Breads, Rolls, Tortillas |
| 51301700 | Bagel, wheat | Lo | Breads, Rolls, Tortillas |
| 51301710 | Bagel, wheat, toasted | Lo | Breads, Rolls, Tortillas |
| 51301750 | Bagel, whole wheat | Lo | Breads, Rolls, Tortillas |
| 51301760 | Bagel, whole wheat, NS as to 100% | Lo | Breads, Rolls, Tortillas |
| 51301800 | Bagel, wheat, with raisins | Lo | Breads, Rolls, Tortillas |
| 51301805 | Bagel, whole wheat, with raisins | Lo | Breads, Rolls, Tortillas |
| 51301820 | Bagel, wheat, with fruit and nuts | Lo | Breads, Rolls, Tortillas |
| 51301830 | Bagel, wheat, with fruit and nuts, to | Lo | Breads, Rolls, Tortillas |
| 51301900 | Bagel, wheat bran | Lo | Breads, Rolls, Tortillas |
| 51302010 | Bread, wheat bran | Lo | Breads, Rolls, Tortillas |
| 51302020 | Bread, wheat bran, toasted | Lo | Breads, Rolls, Tortillas |
| 51302050 | Bread, wheat bran, with raisins | Lo | Breads, Rolls, Tortillas |
| 51302060 | Bread, wheat bran, with raisins, toa | Lo | Breads, Rolls, Tortillas |
| 51302500 | Muffin, English, wheat bran | Lo | Breads, Rolls, Tortillas |
| 51302520 | Muffin, English, wheat bran, with ra | Lo | Breads, Rolls, Tortillas |
| 51303010 | Muffin, English, wheat or cracked w | Lo | Breads, Rolls, Tortillas |
| 51303020 | Muffin, English, wheat or cracked w | Lo | Breads, Rolls, Tortillas |
| 51303030 | Muffin, English, whole wheat | Lo | Breads, Rolls, Tortillas |
| 51303040 | Muffin, English, whole wheat, NS a | Lo | Breads, Rolls, Tortillas |
| 51303050 | Muffin, English, wheat or cracked w | Lo | Breads, Rolls, Tortillas |
| 51303070 | Muffin, English, whole wheat, with r | Lo | Breads, Rolls, Tortillas |
| 51303100 | Muffin, English, whole grain white | Lo | Breads, Rolls, Tortillas |
| 51306000 | Breadsticks, hard, whole wheat | Lo | Crackers |
| 51320010 | Roll, wheat or cracked wheat | Lo | Breads, Rolls, Tortillas |
| 51320020 | Roll, wheat or cracked wheat, toast | Lo | Breads, Rolls, Tortillas |
| 51320040 | Roll, wheat or cracked wheat, mad | Lo | Breads, Rolls, Tortillas |
| 51320060 | Roll, wheat or cracked wheat, hot d | Lo | Breads, Rolls, Tortillas |
| 51320070 | Roll, wheat or cracked wheat, ham | Lo | Breads, Rolls, Tortillas |
| 51320500 | Roll, whole wheat | Lo | Breads, Rolls, Tortillas |
| 51320510 | Roll, whole wheat, NS as to 100%, | Lo | Breads, Rolls, Tortillas |
| 51320530 | Roll, whole wheat, NS as to 100%, | Lo | Breads, Rolls, Tortillas |
| 51320550 | Roll, whole wheat, hot dog bun | Lo | Breads, Rolls, Tortillas |
| 51320560 | Roll, whole wheat, hamburger bun | Lo | Breads, Rolls, Tortillas |
| 51320700 | Roll, whole grain white | Lo | Breads, Rolls, Tortillas |
| 51320710 | Roll, whole grain white, hot dog bun | Lo | Breads, Rolls, Tortillas |
| 51320720 | Roll, whole grain white, hamburger | Lo | Breads, Rolls, Tortillas |
| 51401010 | Bread, rye | Lo | Breads, Rolls, Tortillas |
| 51401020 | Bread, rye, toasted | Lo | Breads, Rolls, Tortillas |
| 51401030 | Bread, marble rye and pumpernick | Lo | Breads, Rolls, Tortillas |

| 51401040 | Bread, marble rye and pumpernick | Lo | Breads, Rolls, Tortillas |
| --- | --- | --- | --- |
| 51401060 | Bread, rye, reduced calorie and/or | Lo | Breads, Rolls, Tortillas |
| 51401070 | Bread, rye, reduced calorie and/or | Lo | Breads, Rolls, Tortillas |
| 51404010 | Bread, pumpernickel | Lo | Breads, Rolls, Tortillas |
| 51404020 | Bread, pumpernickel, toasted | Lo | Breads, Rolls, Tortillas |
| 51404500 | Bagel, pumpernickel | Lo | Breads, Rolls, Tortillas |
| 51404550 | Muffin, English, pumpernickel | Lo | Breads, Rolls, Tortillas |
| 51407010 | Bread, black | Lo | Breads, Rolls, Tortillas |
| 51407020 | Bread, black, toasted | Lo | Breads, Rolls, Tortillas |
| 51420000 | Roll, rye | Lo | Breads, Rolls, Tortillas |
| 51421000 | Roll, pumpernickel | Lo | Breads, Rolls, Tortillas |
| 51501010 | Bread, oatmeal | Lo | Breads, Rolls, Tortillas |
| 51501020 | Bread, oatmeal, toasted | Lo | Breads, Rolls, Tortillas |
| 51501040 | Bread, oat bran | Lo | Breads, Rolls, Tortillas |
| 51501050 | Bread, oat bran, toasted | Lo | Breads, Rolls, Tortillas |
| 51501060 | Bread, oat bran, reduced calorie an | Lo | Breads, Rolls, Tortillas |
| 51501070 | Bread, oat bran, reduced calorie an | Lo | Breads, Rolls, Tortillas |
| 51501080 | Bagel, oat bran | Lo | Breads, Rolls, Tortillas |
| 51502010 | Roll, oatmeal | Lo | Breads, Rolls, Tortillas |
| 51502100 | Roll, oat bran | Lo | Breads, Rolls, Tortillas |
| 51503000 | Muffin, English, oat bran | Lo | Breads, Rolls, Tortillas |
| 51601010 | Bread, multigrain, toasted | Lo | Breads, Rolls, Tortillas |
| 51601020 | Bread, multigrain | Lo | Breads, Rolls, Tortillas |
| 51601210 | Bread, multigrain, with raisins | Lo | Breads, Rolls, Tortillas |
| 51601220 | Bread, multigrain, with raisins, toas | Lo | Breads, Rolls, Tortillas |
| 51602010 | Bread, multigrain, reduced calorie a | Lo | Breads, Rolls, Tortillas |
| 51602020 | Bread, multigrain, reduced calorie a | Lo | Breads, Rolls, Tortillas |
| 51620000 | Roll, multigrain | Lo | Breads, Rolls, Tortillas |
| 51620020 | Roll, multigrain, hot dog bun | Lo | Breads, Rolls, Tortillas |
| 51620030 | Roll, multigrain, hamburger bun | Lo | Breads, Rolls, Tortillas |
| 51630000 | Bagel, multigrain | Lo | Breads, Rolls, Tortillas |
| 51630100 | Bagel, multigrain, with raisins | Lo | Breads, Rolls, Tortillas |
| 51630110 | Bagel, multigrain, with raisins, toast | Lo | Breads, Rolls, Tortillas |
| 51630200 | Muffin, English, multigrain | Lo | Breads, Rolls, Tortillas |
| 51801010 | Bread, barley | Lo | Breads, Rolls, Tortillas |
| 51801020 | Bread, barley, toasted | Lo | Breads, Rolls, Tortillas |
| 51804010 | Bread, soy | Lo | Breads, Rolls, Tortillas |
| 51804020 | Bread, soy, toasted | Lo | Breads, Rolls, Tortillas |
| 51805010 | Bread, sunflower meal | Lo | Breads, Rolls, Tortillas |
| 51805020 | Bread, sunflower meal, toasted | Lo | Breads, Rolls, Tortillas |
| 51806010 | Bread, rice | Lo | Breads, Rolls, Tortillas |
| 51806020 | Bread, rice, toasted | Lo | Breads, Rolls, Tortillas |
| 51807000 | Injera, Ethiopian bread | Lo | Breads, Rolls, Tortillas |
| 51808000 | Bread, gluten free | Lo | Breads, Rolls, Tortillas |
| 51808010 | Bread, gluten free, toasted | Lo | Breads, Rolls, Tortillas |
| 51808050 | Breadsticks, hard, gluten free | Lo | Crackers |
| 51808100 | Roll, gluten free | Lo | Breads, Rolls, Tortillas |
| 52101000 | Biscuit, NFS | Lo | Quick Breads and Bread Products |
| 52101030 | Biscuit dough, fried | Lo | Quick Breads and Bread Products |
| 52101040 | Crumpet | Lo | Quick Breads and Bread Products |
| 52101100 | Biscuit, baking powder or buttermilk | Lo | Quick Breads and Bread Products |

| 52101150 | Biscuit, baking powder or buttermilk | Lo | Quick Breads and Bread Products |
| --- | --- | --- | --- |
| 52102040 | Biscuit, from refrigerated dough | Lo | Quick Breads and Bread Products |
| 52103000 | Biscuit, from fast food / restaurant | Lo | Quick Breads and Bread Products |
| 52104010 | Biscuit, home recipe | Lo | Quick Breads and Bread Products |
| 52104040 | Biscuit, wheat | Lo | Quick Breads and Bread Products |
| 52104100 | Biscuit, cheese | Lo | Quick Breads and Bread Products |
| 52104200 | Biscuit with fruit | Lo | Quick Breads and Bread Products |
| 52105100 | Scone | Lo | Quick Breads and Bread Products |
| 52105200 | Scone, with fruit | Lo | Quick Breads and Bread Products |
| 52201000 | Cornbread, prepared from mix | Lo | Quick Breads and Bread Products |
| 52202060 | Cornbread, made from home recip | Lo | Quick Breads and Bread Products |
| 52204000 | Cornbread stuffing | Lo | Mixed Dishes - Grain-based |
| 52206010 | Cornbread muffin, stick, round | Lo | Quick Breads and Bread Products |
| 52206060 | Cornbread muffin, stick, round, ma | Lo | Quick Breads and Bread Products |
| 52207010 | Corn flour patty or tart, fried | Lo | Quick Breads and Bread Products |
| 52208010 | Corn pone, baked | Lo | Quick Breads and Bread Products |
| 52208020 | Corn pone, fried | Lo | Quick Breads and Bread Products |
| 52208750 | Gordita/sope shell, plain, no filling, | Lo | Breads, Rolls, Tortillas |
| 52208760 | Gordita/sope shell, plain, no filling | Lo | Breads, Rolls, Tortillas |
| 52209010 | Hush puppy | Lo | Quick Breads and Bread Products |
| 52215000 | Tortilla, NFS | Lo | Breads, Rolls, Tortillas |
| 52215100 | Tortilla, corn | Lo | Breads, Rolls, Tortillas |
| 52215200 | Tortilla, flour | Lo | Breads, Rolls, Tortillas |
| 52215260 | Tortilla, whole wheat | Lo | Breads, Rolls, Tortillas |
| 52215300 | Taco shell, corn | Lo | Breads, Rolls, Tortillas |
| 52215350 | Taco shell, flour | Lo | Breads, Rolls, Tortillas |
| 52220110 | Arepa Dominicana | Lo | Quick Breads and Bread Products |
| 52301000 | Muffin, NFS | Lo | Quick Breads and Bread Products |
| 52302010 | Muffin, fruit | Lo | Quick Breads and Bread Products |
| 52302020 | Muffin, fruit, low fat | Lo | Quick Breads and Bread Products |
| 52302100 | Muffin, fruit, fat free, cholesterol fre | Lo | Quick Breads and Bread Products |
| 52302500 | Muffin, chocolate chip | Lo | Quick Breads and Bread Products |
| 52302600 | Muffin, chocolate | Lo | Quick Breads and Bread Products |
| 52302610 | Muffin, chocolate, lowfat | Lo | Quick Breads and Bread Products |
| 52303010 | Muffin, whole wheat | Lo | Quick Breads and Bread Products |
| 52303500 | Muffin, wheat | Lo | Quick Breads and Bread Products |
| 52304000 | Muffin, whole grain | Lo | Quick Breads and Bread Products |
| 52304010 | Muffin, wheat bran | Lo | Quick Breads and Bread Products |
| 52304040 | Muffin, bran with fruit, lowfat | Lo | Quick Breads and Bread Products |
| 52304060 | Muffin, bran with fruit, no fat, no ch | Lo | Quick Breads and Bread Products |
| 52304100 | Muffin, oatmeal | Lo | Quick Breads and Bread Products |
| 52304150 | Muffin, oat bran | Lo | Quick Breads and Bread Products |
| 52304200 | Muffin, oat bran with fruit and/or nut | Lo | Quick Breads and Bread Products |
| 52306010 | Muffin, plain | Lo | Quick Breads and Bread Products |
| 52306300 | Muffin, cheese | Lo | Quick Breads and Bread Products |
| 52306500 | Muffin, pumpkin | Lo | Quick Breads and Bread Products |
| 52306550 | Muffin, zucchini | Lo | Quick Breads and Bread Products |
| 52306700 | Muffin, carrot | Lo | Quick Breads and Bread Products |
| 52307120 | Muffin, multigrain, with fruit | Lo | Quick Breads and Bread Products |
| 52308010 | Matzo, fritters | Lo | Quick Breads and Bread Products |
| 52308020 | Matzo ball | Lo | Quick Breads and Bread Products |

| 52311010 | Popover | Lo | Quick Breads and Bread Products |
| --- | --- | --- | --- |
| 52320110 | Toaster muffin, fruit, toasted | Lo | Quick Breads and Bread Products |
| 52401000 | Bread, Boston Brown | Lo | Quick Breads and Bread Products |
| 52403000 | Bread, nut | Lo | Quick Breads and Bread Products |
| 52404060 | Bread, pumpkin | Lo | Quick Breads and Bread Products |
| 52405010 | Bread, fruit | Lo | Quick Breads and Bread Products |
| 52405100 | Bread, fruit and nut | Lo | Quick Breads and Bread Products |
| 52406010 | Bread, whole wheat, with nuts | Lo | Quick Breads and Bread Products |
| 52407000 | Bread, zucchini | Lo | Quick Breads and Bread Products |
| 52408000 | Bread, Irish soda | Lo | Quick Breads and Bread Products |
| 53100050 | Cake batter, raw, chocolate | Lo | Sweet Bakery Products |
| 53100070 | Cake batter, raw, not chocolate | Lo | Sweet Bakery Products |
| 53100100 | Cake or cupcake, NS as to type | Lo | Sweet Bakery Products |
| 53101100 | Cake, angel food, without icing or fi | Lo | Sweet Bakery Products |
| 53101200 | Cake, angel food, with icing or fillin | Lo | Sweet Bakery Products |
| 53101250 | Cake, angel food, with fruit and icin | Lo | Sweet Bakery Products |
| 53101300 | Cake, angel food, chocolate, withou | Lo | Sweet Bakery Products |
| 53102000 | Cake, applesauce, NS as to icing | Lo | Sweet Bakery Products |
| 53102100 | Cake or cupcake, applesauce, with | Lo | Sweet Bakery Products |
| 53102200 | Cake or cupcake, applesauce, with | Lo | Sweet Bakery Products |
| 53102300 | Cake, applesauce, diet, without icin | Lo | Sweet Bakery Products |
| 53102500 | Cake, banana, NS as to icing | Lo | Sweet Bakery Products |
| 53102600 | Cake or cupcake, banana, without i | Lo | Sweet Bakery Products |
| 53102700 | Cake or cupcake, banana, with icin | Lo | Sweet Bakery Products |
| 53102800 | Cake or cupcake, Black Forest | Lo | Sweet Bakery Products |
| 53103000 | Cake, Boston cream pie | Lo | Sweet Bakery Products |
| 53103550 | Cake, butter, without icing | Lo | Sweet Bakery Products |
| 53103600 | Cake, butter, with icing | Lo | Sweet Bakery Products |
| 53104000 | Cake, carrot, NS as to icing | Lo | Sweet Bakery Products |
| 53104100 | Cake or cupcake, carrot, without ici | Lo | Sweet Bakery Products |
| 53104260 | Cake or cupcake, carrot, with icing | Lo | Sweet Bakery Products |
| 53104300 | Cake, carrot, diet | Lo | Sweet Bakery Products |
| 53104400 | Cake or cupcake, coconut, with icin | Lo | Sweet Bakery Products |
| 53104500 | Cheesecake | Lo | Sweet Bakery Products |
| 53104520 | Cheesecake, diet | Lo | Sweet Bakery Products |
| 53104550 | Cheesecake with fruit | Lo | Sweet Bakery Products |
| 53104570 | Cheesecake, diet, with fruit | Lo | Sweet Bakery Products |
| 53104580 | Cheesecake -type dessert, made w | Lo | Sweet Bakery Products |
| 53104600 | Cheesecake, chocolate | Lo | Sweet Bakery Products |
| 53105050 | Cake, chocolate, devil's food, or fud | Lo | Sweet Bakery Products |
| 53105100 | Cake, chocolate, devil's food, or fud | Lo | Sweet Bakery Products |
| 53105160 | Cake, chocolate, devil's food, or fud | Lo | Sweet Bakery Products |
| 53105200 | Cake, chocolate, devil's food, or fud | Lo | Sweet Bakery Products |
| 53105260 | Cake, chocolate, devil's food, or fud | Lo | Sweet Bakery Products |
| 53105270 | Cake or cupcake, chocolate, devil's | Lo | Sweet Bakery Products |
| 53105275 | Cake or cupcake, chocolate, devil's | Lo | Sweet Bakery Products |
| 53105300 | Cake or cupcake, German chocolat | Lo | Sweet Bakery Products |
| 53105500 | Cake, chocolate, with icing, diet | Lo | Sweet Bakery Products |
| 53105600 | Cake, chocolate, devil's food, or fud | Lo | Sweet Bakery Products |
| 53105700 | Cake, chocolate, devil's food, or fud | Lo | Sweet Bakery Products |
| 53105750 | Cake, chocolate, devil's food, or fud | Lo | Sweet Bakery Products |

| 53106000 | Cake, chocolate, devil's food, or fud | Lo | Sweet Bakery Products |
| --- | --- | --- | --- |
| 53106050 | Cake, chocolate, devil's food, or fud | Lo | Sweet Bakery Products |
| 53106500 | Cake, cream, without icing or toppi | Lo | Sweet Bakery Products |
| 53107000 | Cake, cupcake, NS as to type or ici | Lo | Sweet Bakery Products |
| 53107100 | Cake, cupcake, NS as to type, with | Lo | Sweet Bakery Products |
| 53107200 | Cake, cupcake, NS as to type, with | Lo | Sweet Bakery Products |
| 53108000 | Cake, cupcake, chocolate, NS as to | Lo | Sweet Bakery Products |
| 53108100 | Cake, cupcake, chocolate, without i | Lo | Sweet Bakery Products |
| 53108200 | Snack cake, chocolate, with icing o | Lo | Sweet Bakery Products |
| 53108220 | Snack cake, chocolate, with icing o | Lo | Sweet Bakery Products |
| 53109000 | Cake, cupcake, not chocolate, NS | Lo | Sweet Bakery Products |
| 53109100 | Cake, cupcake, not chocolate, with | Lo | Sweet Bakery Products |
| 53109200 | Snack cake, not chocolate, with icin | Lo | Sweet Bakery Products |
| 53109210 | Cake, cupcake, not chocolate, with | Lo | Sweet Bakery Products |
| 53109220 | Snack cake, not chocolate, with icin | Lo | Sweet Bakery Products |
| 53109250 | Cake, cupcake, not chocolate, with | Lo | Sweet Bakery Products |
| 53109270 | Cake, cupcake, chocolate, with or w | Lo | Sweet Bakery Products |
| 53109300 | Cake, Dobos Torte | Lo | Sweet Bakery Products |
| 53110000 | Cake, fruit cake, light or dark, holid | Lo | Sweet Bakery Products |
| 53111000 | Cake or cupcake, gingerbread | Lo | Sweet Bakery Products |
| 53111500 | Cake, graham cracker, without icin | Lo | Sweet Bakery Products |
| 53112000 | Cake, ice cream and cake roll, cho | Lo | Sweet Bakery Products |
| 53112100 | Ice cream cake | Lo | Sweet Bakery Products |
| 53113000 | Cake, jelly roll | Lo | Sweet Bakery Products |
| 53114000 | Cake or cupcake, lemon, without ic | Lo | Sweet Bakery Products |
| 53114100 | Cake or cupcake, lemon, with icing | Lo | Sweet Bakery Products |
| 53114200 | Cake, lemon, lowfat, without icing | Lo | Sweet Bakery Products |
| 53114250 | Cake, lemon, lowfat, with icing | Lo | Sweet Bakery Products |
| 53115100 | Cake or cupcake, marble, without i | Lo | Sweet Bakery Products |
| 53115200 | Cake or cupcake, marble, with icing | Lo | Sweet Bakery Products |
| 53115310 | Cake or cupcake, nut, without icing | Lo | Sweet Bakery Products |
| 53115320 | Cake or cupcake, nut, with icing or | Lo | Sweet Bakery Products |
| 53115400 | Cake, oatmeal, without icing | Lo | Sweet Bakery Products |
| 53115410 | Cake or cupcake, oatmeal | Lo | Sweet Bakery Products |
| 53115450 | Cake or cupcake, peanut butter | Lo | Sweet Bakery Products |
| 53115600 | Cake, poppyseed, without icing | Lo | Sweet Bakery Products |
| 53116000 | Cake, pound, without icing or filling | Lo | Sweet Bakery Products |
| 53116020 | Cake, pound, with icing or filling | Lo | Sweet Bakery Products |
| 53116270 | Cake, pound, chocolate | Lo | Sweet Bakery Products |
| 53116350 | Cake, pound, Puerto Rican style | Lo | Sweet Bakery Products |
| 53116380 | Cake, pound, fat free, cholesterol fr | Lo | Sweet Bakery Products |
| 53116390 | Cake, pound, reduced fat, choleste | Lo | Sweet Bakery Products |
| 53116490 | Cake, pumpkin, NS as to icing | Lo | Sweet Bakery Products |
| 53116500 | Cake or cupcake, pumpkin, without | Lo | Sweet Bakery Products |
| 53116510 | Cake or cupcake, pumpkin, with ici | Lo | Sweet Bakery Products |
| 53116550 | Cake or cupcake, raisin-nut | Lo | Sweet Bakery Products |
| 53116560 | Cake, raisin-nut, with icing | Lo | Sweet Bakery Products |
| 53116570 | Cake, Ravani | Lo | Sweet Bakery Products |
| 53116600 | Cake, rice flour, without icing or filli | Lo | Sweet Bakery Products |
| 53116650 | Cake, Quezadilla, El Salvadorian st | Lo | Sweet Bakery Products |
| 53117100 | Cake or cupcake, spice, without ici | Lo | Sweet Bakery Products |

| 53117200 | Cake or cupcake, spice, with icing | Lo | Sweet Bakery Products |
| --- | --- | --- | --- |
| 53118100 | Cake, sponge, without icing or fillin | Lo | Sweet Bakery Products |
| 53118200 | Cake, sponge, with icing or filling | Lo | Sweet Bakery Products |
| 53118300 | Cake, sponge, chocolate | Lo | Sweet Bakery Products |
| 53118310 | Cake, sponge, chocolate, with icing | Lo | Sweet Bakery Products |
| 53118350 | Cake, sweetpotato, with icing | Lo | Sweet Bakery Products |
| 53118410 | Rum cake, without icing | Lo | Sweet Bakery Products |
| 53118500 | Cake, torte | Lo | Sweet Bakery Products |
| 53118550 | Cake, tres leche | Lo | Sweet Bakery Products |
| 53119000 | Cake, pineapple, upside down | Lo | Sweet Bakery Products |
| 53120000 | Cake, white, standard-type mix (eg | Lo | Sweet Bakery Products |
| 53120060 | Cake, white, made from home recip | Lo | Sweet Bakery Products |
| 53120100 | Cake, white, standard-type mix (eg | Lo | Sweet Bakery Products |
| 53120160 | Cake, white, without icing, made fro | Lo | Sweet Bakery Products |
| 53120200 | Cake, white, standard-type mix (eg | Lo | Sweet Bakery Products |
| 53120260 | Cake, white, with icing, made from | Lo | Sweet Bakery Products |
| 53120270 | Cake or cupcake, white, with icing o | Lo | Sweet Bakery Products |
| 53120275 | Cake or cupcake, white, without ici | Lo | Sweet Bakery Products |
| 53120330 | Cake, white, pudding-type mix (oil, | Lo | Sweet Bakery Products |
| 53120350 | Cake, white, pudding-type mix (oil, | Lo | Sweet Bakery Products |
| 53120400 | Cake, white, eggless, lowfat | Lo | Sweet Bakery Products |
| 53120500 | Cake, whole wheat, with fruit and n | Lo | Sweet Bakery Products |
| 53121060 | Cake, yellow, made from home rec | Lo | Sweet Bakery Products |
| 53121100 | Cake, yellow, standard-type mix (eg | Lo | Sweet Bakery Products |
| 53121160 | Cake, yellow, without icing, made fr | Lo | Sweet Bakery Products |
| 53121200 | Cake, yellow, standard-type mix (eg | Lo | Sweet Bakery Products |
| 53121260 | Cake, yellow, with icing, made from | Lo | Sweet Bakery Products |
| 53121270 | Cake or cupcake, yellow, with icing | Lo | Sweet Bakery Products |
| 53121275 | Cake or cupcake, yellow, without ic | Lo | Sweet Bakery Products |
| 53121280 | Cake, yellow, pudding-type mix (oil, | Lo | Sweet Bakery Products |
| 53121300 | Cake, yellow, pudding-type mix (oil, | Lo | Sweet Bakery Products |
| 53121330 | Cake, yellow, pudding-type mix (oil, | Lo | Sweet Bakery Products |
| 53122070 | Cake, shortcake, biscuit type, with | Lo | Sweet Bakery Products |
| 53122080 | Cake, shortcake, biscuit type, with f | Lo | Sweet Bakery Products |
| 53123070 | Cake, shortcake, sponge type, with | Lo | Sweet Bakery Products |
| 53123080 | Cake, shortcake, sponge type, with | Lo | Sweet Bakery Products |
| 53123500 | Cake, shortcake, with whipped topp | Lo | Sweet Bakery Products |
| 53124110 | Cake or cupcake, zucchini | Lo | Sweet Bakery Products |
| 53124120 | Cake, zucchini, with icing | Lo | Sweet Bakery Products |
| 53200100 | Cookie, batter or dough, raw | Lo | Sweet Bakery Products |
| 53201000 | Cookie, NFS | Lo | Sweet Bakery Products |
| 53202000 | Cookie, almond | Lo | Sweet Bakery Products |
| 53203000 | Cookie, applesauce | Lo | Sweet Bakery Products |
| 53203050 | Cookie, fruit, baby | Lo | Baby Foods |
| 53203100 | Cookie, baby | Lo | Baby Foods |
| 53203500 | Cookie, biscotti | Lo | Sweet Bakery Products |
| 53204000 | Cookie, brownie, NS as to icing | Lo | Sweet Bakery Products |
| 53204010 | Cookie, brownie, without icing | Lo | Sweet Bakery Products |
| 53204100 | Cookie, brownie, with icing or filling | Lo | Sweet Bakery Products |
| 53204500 | Cookie, brownie, with cream chees | Lo | Sweet Bakery Products |
| 53204600 | Cookie, brownie, with peanut butter | Lo | Sweet Bakery Products |

| 53204800 | Cookie, brownie, diet, NS as to icin | Lo | Sweet Bakery Products |
| --- | --- | --- | --- |
| 53204830 | Cookie, brownie, lowfat, with icing | Lo | Sweet Bakery Products |
| 53204840 | Cookie, brownie, reduced fat, NS a | Lo | Sweet Bakery Products |
| 53204850 | Cookie, brownie, fat free, cholester | Lo | Sweet Bakery Products |
| 53204860 | Cookie, brownie, fat free, NS as to | Lo | Sweet Bakery Products |
| 53205250 | Cookie, butterscotch, brownie | Lo | Sweet Bakery Products |
| 53205260 | Cookie, bar, with chocolate | Lo | Sweet Bakery Products |
| 53205500 | Cookie, butterscotch chip | Lo | Sweet Bakery Products |
| 53205600 | Cookie, caramel coated, with nuts | Lo | Sweet Bakery Products |
| 53206000 | Cookie, chocolate chip | Lo | Sweet Bakery Products |
| 53206010 | Cookie, chocolate chip, with raisins | Lo | Sweet Bakery Products |
| 53206020 | Cookie, chocolate chip, made from | Lo | Sweet Bakery Products |
| 53206030 | Cookie, chocolate chip, reduced fat | Lo | Sweet Bakery Products |
| 53206050 | Cookie, rich, chocolate chip, with c | Lo | Sweet Bakery Products |
| 53206100 | Cookie, chocolate chip sandwich | Lo | Sweet Bakery Products |
| 53206500 | Cookie, chocolate, made with rice c | Lo | Sweet Bakery Products |
| 53206550 | Cookie, chocolate, made with oatm | Lo | Sweet Bakery Products |
| 53207000 | Cookie, chocolate or fudge | Lo | Sweet Bakery Products |
| 53207020 | Cookie, chocolate or fudge, reduce | Lo | Sweet Bakery Products |
| 53207050 | Cookie, chocolate, with chocolate fi | Lo | Sweet Bakery Products |
| 53208000 | Cookie, marshmallow, chocolate-co | Lo | Sweet Bakery Products |
| 53208200 | Cookie, marshmallow pie, chocolat | Lo | Sweet Bakery Products |
| 53209000 | Cookie, chocolate, chocolate sandw | Lo | Sweet Bakery Products |
| 53209005 | Cookie, chocolate, with icing or coa | Lo | Sweet Bakery Products |
| 53209010 | Cookie, sugar wafer, chocolate-cov | Lo | Sweet Bakery Products |
| 53209015 | Cookie, chocolate sandwich | Lo | Sweet Bakery Products |
| 53209020 | Cookie, chocolate sandwich, reduc | Lo | Sweet Bakery Products |
| 53209050 | Cookie, chocolate-covered, chocola | Lo | Sweet Bakery Products |
| 53209100 | Cookie, chocolate, sandwich, with e | Lo | Sweet Bakery Products |
| 53209500 | Cookie, chocolate and vanilla sand | Lo | Sweet Bakery Products |
| 53210000 | Cookie, chocolate wafer | Lo | Sweet Bakery Products |
| 53210900 | Cookie, graham cracker with choco | Lo | Sweet Bakery Products |
| 53210910 | Cookie, graham cracker with marsh | Lo | Sweet Bakery Products |
| 53211000 | Cookie bar, with chocolate, nuts, an | Lo | Sweet Bakery Products |
| 53215500 | Cookie, coconut | Lo | Sweet Bakery Products |
| 53216000 | Cookie, coconut and nut | Lo | Sweet Bakery Products |
| 53220000 | Cookie, fruit-filled bar | Lo | Sweet Bakery Products |
| 53220010 | Cookie, fruit-filled bar, fat free | Lo | Sweet Bakery Products |
| 53220020 | Cookie, date bar | Lo | Sweet Bakery Products |
| 53220030 | Cookie, fig bar | Lo | Sweet Bakery Products |
| 53220040 | Cookie, fig bar, fat free | Lo | Sweet Bakery Products |
| 53222010 | Cookie, fortune | Lo | Sweet Bakery Products |
| 53222020 | Cookie, cone shell, ice cream type, | Lo | Sweet Bakery Products |
| 53222100 | Cookie, cone shell, ice cream type, | Lo | Sweet Bakery Products |
| 53223000 | Cookie, gingersnaps | Lo | Sweet Bakery Products |
| 53223100 | Cookie, granola | Lo | Sweet Bakery Products |
| 53224000 | Cookie, ladyfinger | Lo | Sweet Bakery Products |
| 53224250 | Cookie, lemon bar | Lo | Sweet Bakery Products |
| 53225000 | Cookie, macaroon | Lo | Sweet Bakery Products |
| 53226000 | Cookie, marshmallow, with coconut | Lo | Sweet Bakery Products |
| 53226500 | Cookie, marshmallow, with rice cer | Lo | Sweet Bakery Products |

| 53226550 | Cookie, marshmallow, with rice cer | Lo | Sweet Bakery Products |
| --- | --- | --- | --- |
| 53226600 | Cookie, marshmallow and peanut b | Lo | Sweet Bakery Products |
| 53227000 | Cookie, marshmallow pies, non-cho | Lo | Sweet Bakery Products |
| 53228000 | Cookie, meringue | Lo | Sweet Bakery Products |
| 53230000 | Cookie, molasses | Lo | Sweet Bakery Products |
| 53231000 | Cookie, Lebkuchen | Lo | Sweet Bakery Products |
| 53231400 | Cookie, multigrain, high fiber | Lo | Sweet Bakery Products |
| 53233000 | Cookie, oatmeal | Lo | Sweet Bakery Products |
| 53233010 | Cookie, oatmeal, with raisins | Lo | Sweet Bakery Products |
| 53233020 | Cookie, oatmeal, with fruit filling | Lo | Sweet Bakery Products |
| 53233030 | Cookie, oatmeal, fat free, with raisi | Lo | Sweet Bakery Products |
| 53233040 | Cookie, oatmeal, reduced fat, NS a | Lo | Sweet Bakery Products |
| 53233050 | Cookie, oatmeal sandwich, with cre | Lo | Sweet Bakery Products |
| 53233060 | Cookie, oatmeal, with chocolate chi | Lo | Sweet Bakery Products |
| 53233080 | Cookie, oatmeal sandwich, with pe | Lo | Sweet Bakery Products |
| 53233100 | Cookie, oatmeal, with chocolate an | Lo | Sweet Bakery Products |
| 53233500 | Cookie, oat bran | Lo | Sweet Bakery Products |
| 53234000 | Cookie, peanut butter | Lo | Sweet Bakery Products |
| 53234010 | Cookie, peanut butter, with oatmea | Lo | Sweet Bakery Products |
| 53234100 | Cookie, peanut butter, with chocola | Lo | Sweet Bakery Products |
| 53234250 | Cookie, peanut butter with rice cere | Lo | Sweet Bakery Products |
| 53235000 | Cookie, peanut butter sandwich | Lo | Sweet Bakery Products |
| 53235500 | Cookie, with peanut butter filling, ch | Lo | Sweet Bakery Products |
| 53235600 | Cookie, Pfeffernusse | Lo | Sweet Bakery Products |
| 53236000 | Cookie, Pizzelle | Lo | Sweet Bakery Products |
| 53236100 | Cookie, pumpkin | Lo | Sweet Bakery Products |
| 53237000 | Cookie, raisin | Lo | Sweet Bakery Products |
| 53237010 | Cookie, raisin sandwich, cream-fille | Lo | Sweet Bakery Products |
| 53237500 | Cookie, rum ball, no bake | Lo | Sweet Bakery Products |
| 53238000 | Cookie, sandwich-type, not chocola | Lo | Sweet Bakery Products |
| 53239000 | Cookie, shortbread | Lo | Sweet Bakery Products |
| 53239010 | Cookie, shortbread, reduced fat | Lo | Sweet Bakery Products |
| 53239050 | Cookie, shortbread, with icing or fill | Lo | Sweet Bakery Products |
| 53239100 | Pocky | Lo | Sweet Bakery Products |
| 53240000 | Cookie, animal | Lo | Sweet Bakery Products |
| 53240010 | Cookie, animal, with frosting or icin | Lo | Sweet Bakery Products |
| 53241500 | Cookie, butter or sugar | Lo | Sweet Bakery Products |
| 53241510 | Marie biscuit | Lo | Sweet Bakery Products |
| 53241600 | Cookie, butter or sugar, with fruit an | Lo | Sweet Bakery Products |
| 53242000 | Cookie, sugar wafer | Lo | Sweet Bakery Products |
| 53242250 | Cookie, teething, baby food | Lo | Baby Foods |
| 53242500 | Cookie, toffee bar | Lo | Sweet Bakery Products |
| 53243000 | Cookie, vanilla sandwich | Lo | Sweet Bakery Products |
| 53243010 | Cookie, vanilla sandwich, extra fillin | Lo | Sweet Bakery Products |
| 53243050 | Cookie, vanilla sandwich, reduced f | Lo | Sweet Bakery Products |
| 53243100 | Cookie, rich, all chocolate, with cho | Lo | Sweet Bakery Products |
| 53244010 | Cookie, butter or sugar, with chocol | Lo | Sweet Bakery Products |
| 53244020 | Cookie, butter or sugar, with icing o | Lo | Sweet Bakery Products |
| 53245000 | Cookie, vanilla waffle creme | Lo | Sweet Bakery Products |
| 53246000 | Cookie, tea, Japanese | Lo | Sweet Bakery Products |
| 53247000 | Cookie, vanilla wafer | Lo | Sweet Bakery Products |

| 53247050 | Cookie, vanilla wafer, reduced fat | Lo | Sweet Bakery Products |
| --- | --- | --- | --- |
| 53247500 | Cookie, vanilla with caramel, cocon | Lo | Sweet Bakery Products |
| 53248000 | Cookie, whole wheat, dried fruit, nu | Lo | Sweet Bakery Products |
| 53251100 | Cookie, rugelach | Lo | Sweet Bakery Products |
| 53260030 | Cookie, chocolate chip, sugar free | Lo | Sweet Bakery Products |
| 53260150 | Cookie, lemon wafer, lowfat | Lo | Sweet Bakery Products |
| 53260200 | Cookie, oatmeal, sugar free | Lo | Sweet Bakery Products |
| 53260300 | Cookie, sandwich, sugar free | Lo | Sweet Bakery Products |
| 53260400 | Cookie, sugar or plain, sugar free | Lo | Sweet Bakery Products |
| 53260500 | Cookie, sugar wafer, sugar free | Lo | Sweet Bakery Products |
| 53260600 | Cookie, peanut butter, sugar free | Lo | Sweet Bakery Products |
| 53261000 | Cookie, gluten free | Lo | Sweet Bakery Products |
| 53270100 | Cookies, Puerto Rican style | Lo | Sweet Bakery Products |
| 53300100 | Pie, NFS | Lo | Sweet Bakery Products |
| 53300170 | Pie, individual size or tart, NFS | Lo | Sweet Bakery Products |
| 53301000 | Pie, apple, two crust | Lo | Sweet Bakery Products |
| 53301070 | Pie, apple, individual size or tart | Lo | Sweet Bakery Products |
| 53301080 | Pie, apple, fried pie | Lo | Sweet Bakery Products |
| 53301500 | Pie, apple, one crust | Lo | Sweet Bakery Products |
| 53301750 | Pie, apple, diet | Lo | Sweet Bakery Products |
| 53302000 | Pie, apricot, two crust | Lo | Sweet Bakery Products |
| 53302070 | Pie, apricot, individual size or tart | Lo | Sweet Bakery Products |
| 53303000 | Pie, blackberry, two crust | Lo | Sweet Bakery Products |
| 53303070 | Pie, blackberry, individual size or ta | Lo | Sweet Bakery Products |
| 53303500 | Pie, berry, not blackberry, blueberry | Lo | Sweet Bakery Products |
| 53303510 | Pie, berry, not blackberry, blueberry | Lo | Sweet Bakery Products |
| 53303570 | Pie, berry, not blackberry, blueberry | Lo | Sweet Bakery Products |
| 53304000 | Pie, blueberry, two crust | Lo | Sweet Bakery Products |
| 53304050 | Pie, blueberry, one crust | Lo | Sweet Bakery Products |
| 53304070 | Pie, blueberry, individual size or tart | Lo | Sweet Bakery Products |
| 53305000 | Pie, cherry, two crust | Lo | Sweet Bakery Products |
| 53305010 | Pie, cherry, one crust | Lo | Sweet Bakery Products |
| 53305070 | Pie, cherry, individual size or tart | Lo | Sweet Bakery Products |
| 53305080 | Pie, cherry, fried pie | Lo | Sweet Bakery Products |
| 53305700 | Pie, lemon, not cream or meringue | Lo | Sweet Bakery Products |
| 53305720 | Pie, lemon, not cream or meringue, | Lo | Sweet Bakery Products |
| 53306000 | Pie, mince, two crust | Lo | Sweet Bakery Products |
| 53306070 | Pie, mince, individual size or tart | Lo | Sweet Bakery Products |
| 53307000 | Pie, peach, two crust | Lo | Sweet Bakery Products |
| 53307050 | Pie, peach, one crust | Lo | Sweet Bakery Products |
| 53307070 | Pie, peach, individual size or tart | Lo | Sweet Bakery Products |
| 53307080 | Pie, peach, fried pie | Lo | Sweet Bakery Products |
| 53307500 | Pie, pear, two crust | Lo | Sweet Bakery Products |
| 53308000 | Pie, pineapple, two crust | Lo | Sweet Bakery Products |
| 53308070 | Pie, pineapple, individual size or tar | Lo | Sweet Bakery Products |
| 53308500 | Pie, prune, one crust | Lo | Sweet Bakery Products |
| 53309000 | Pie, raisin, two crust | Lo | Sweet Bakery Products |
| 53309070 | Pie, raisin, individual size or tart | Lo | Sweet Bakery Products |
| 53310000 | Pie, raspberry, one crust | Lo | Sweet Bakery Products |
| 53310050 | Pie, raspberry, two crust | Lo | Sweet Bakery Products |
| 53311000 | Pie, rhubarb, two crust | Lo | Sweet Bakery Products |

| 53311050 | Pie, rhubarb, one crust | Lo | Sweet Bakery Products |
| --- | --- | --- | --- |
| 53311070 | Pie, rhubarb, individual size or tart | Lo | Sweet Bakery Products |
| 53312000 | Pie, strawberry, one crust | Lo | Sweet Bakery Products |
| 53313000 | Pie, strawberry-rhubarb, two crust | Lo | Sweet Bakery Products |
| 53314000 | Pie, strawberry, individual size or ta | Lo | Sweet Bakery Products |
| 53340500 | Pie, cherry, made with cream chee | Lo | Sweet Bakery Products |
| 53341000 | Pie, banana cream | Lo | Sweet Bakery Products |
| 53341070 | Pie, banana cream, individual size | Lo | Sweet Bakery Products |
| 53341500 | Pie, buttermilk | Lo | Sweet Bakery Products |
| 53341750 | Pie, chess | Lo | Sweet Bakery Products |
| 53342000 | Pie, chocolate cream | Lo | Sweet Bakery Products |
| 53342070 | Pie, chocolate cream, individual siz | Lo | Sweet Bakery Products |
| 53343000 | Pie, coconut cream | Lo | Sweet Bakery Products |
| 53343070 | Pie, coconut cream, individual size | Lo | Sweet Bakery Products |
| 53344000 | Pie, custard | Lo | Sweet Bakery Products |
| 53344070 | Pie, custard, individual size or tart | Lo | Sweet Bakery Products |
| 53344200 | Mixed fruit tart filled with custard or | Lo | Sweet Bakery Products |
| 53344300 | Dessert pizza | Lo | Sweet Bakery Products |
| 53345000 | Pie, lemon cream | Lo | Sweet Bakery Products |
| 53345070 | Pie, lemon cream, individual size or | Lo | Sweet Bakery Products |
| 53346000 | Pie, peanut butter cream | Lo | Sweet Bakery Products |
| 53346500 | Pie, pineapple cream | Lo | Sweet Bakery Products |
| 53347000 | Pie, pumpkin | Lo | Sweet Bakery Products |
| 53347070 | Pie, pumpkin, individual size or tart | Lo | Sweet Bakery Products |
| 53347100 | Pie, raspberry cream | Lo | Sweet Bakery Products |
| 53347600 | Pie, squash | Lo | Sweet Bakery Products |
| 53348000 | Pie, strawberry cream | Lo | Sweet Bakery Products |
| 53348070 | Pie, strawberry cream, individual si | Lo | Sweet Bakery Products |
| 53360000 | Pie, sweet potato | Lo | Sweet Bakery Products |
| 53365000 | Pie, vanilla cream | Lo | Sweet Bakery Products |
| 53366000 | Pie, yogurt, frozen | Lo | Sweet Bakery Products |
| 53370000 | Pie, chiffon, not chocolate | Lo | Sweet Bakery Products |
| 53381000 | Pie, lemon meringue | Lo | Sweet Bakery Products |
| 53381070 | Pie, lemon meringue, individual siz | Lo | Sweet Bakery Products |
| 53382000 | Pie, chocolate-marshmallow | Lo | Sweet Bakery Products |
| 53385000 | Pie, pecan | Lo | Sweet Bakery Products |
| 53385070 | Pie, pecan, individual size or tart | Lo | Sweet Bakery Products |
| 53385500 | Pie, oatmeal | Lo | Sweet Bakery Products |
| 53386000 | Pie, pudding, flavors other than cho | Lo | Sweet Bakery Products |
| 53386050 | Pie, pudding, flavors other than cho | Lo | Sweet Bakery Products |
| 53386250 | Pie, pudding, chocolate, with choco | Lo | Sweet Bakery Products |
| 53386500 | Pie, pudding, flavors other than cho | Lo | Sweet Bakery Products |
| 53387000 | Pie, Toll house chocolate chip | Lo | Sweet Bakery Products |
| 53390000 | Pie, shoo-fly | Lo | Sweet Bakery Products |
| 53391000 | Pie shell | Lo | Sweet Bakery Products |
| 53391100 | Pie shell, graham cracker | Lo | Sweet Bakery Products |
| 53391150 | Pie shell, chocolate wafer | Lo | Sweet Bakery Products |
| 53400200 | Blintz, cheese-filled | Lo | Quick Breads and Bread Products |
| 53400300 | Blintz, fruit-filled | Lo | Quick Breads and Bread Products |
| 53410100 | Cobbler, apple | Lo | Sweet Bakery Products |
| 53410300 | Cobbler, berry | Lo | Sweet Bakery Products |

| 53410500 | Cobbler, cherry | Lo | Sweet Bakery Products |
| --- | --- | --- | --- |
| 53410800 | Cobbler, peach | Lo | Sweet Bakery Products |
| 53410850 | Cobbler, pear | Lo | Sweet Bakery Products |
| 53410860 | Cobbler, pineapple | Lo | Sweet Bakery Products |
| 53410900 | Cobbler, rhubarb | Lo | Sweet Bakery Products |
| 53415100 | Crisp, apple, apple dessert | Lo | Sweet Bakery Products |
| 53415120 | Fritter, apple | Lo | Sweet Bakery Products |
| 53415200 | Fritter, banana | Lo | Sweet Bakery Products |
| 53415220 | Fritter, berry | Lo | Sweet Bakery Products |
| 53415300 | Crisp, blueberry | Lo | Sweet Bakery Products |
| 53415400 | Crisp, cherry | Lo | Sweet Bakery Products |
| 53415500 | Crisp, peach | Lo | Sweet Bakery Products |
| 53415600 | Crisp, rhubarb | Lo | Sweet Bakery Products |
| 53420000 | Cream puff, eclair, custard or cream | Lo | Sweet Bakery Products |
| 53420100 | Cream puff, eclair, custard or cream | Lo | Sweet Bakery Products |
| 53420200 | Cream puff, eclair, custard or cream | Lo | Sweet Bakery Products |
| 53420210 | Cream puff, eclair, custard or cream | Lo | Sweet Bakery Products |
| 53420250 | Cream puff, no filling or icing | Lo | Sweet Bakery Products |
| 53420300 | Air filled fritter or fried puff, without | Lo | Sweet Bakery Products |
| 53420310 | Wheat flour fritter, without syrup | Lo | Sweet Bakery Products |
| 53420400 | Sopaipilla, without syrup or honey | Lo | Sweet Bakery Products |
| 53420410 | Sopaipilla with syrup or honey | Lo | Sweet Bakery Products |
| 53430000 | Crepe, NS as to filling | Lo | Quick Breads and Bread Products |
| 53430100 | Crepe, chocolate filled | Lo | Quick Breads and Bread Products |
| 53430200 | Crepe, fruit filled | Lo | Quick Breads and Bread Products |
| 53430250 | Crepe suzette | Lo | Quick Breads and Bread Products |
| 53430700 | Tamale, sweet | Lo | Sweet Bakery Products |
| 53430750 | Tamale, sweet, with fruit | Lo | Sweet Bakery Products |
| 53440000 | Strudel, apple | Lo | Sweet Bakery Products |
| 53440300 | Strudel, berry | Lo | Sweet Bakery Products |
| 53440500 | Strudel, cherry | Lo | Sweet Bakery Products |
| 53440600 | Strudel, cheese | Lo | Sweet Bakery Products |
| 53440800 | Strudel, cheese and fruit | Lo | Sweet Bakery Products |
| 53441110 | Baklava | Lo | Sweet Bakery Products |
| 53441210 | Basbousa | Lo | Sweet Bakery Products |
| 53450000 | Turnover or dumpling, apple | Lo | Sweet Bakery Products |
| 53450300 | Turnover or dumpling, berry | Lo | Sweet Bakery Products |
| 53450500 | Turnover or dumpling, cherry | Lo | Sweet Bakery Products |
| 53450800 | Turnover or dumpling, lemon | Lo | Sweet Bakery Products |
| 53451000 | Turnover or dumpling, peach | Lo | Sweet Bakery Products |
| 53451500 | Turnover, guava | Lo | Sweet Bakery Products |
| 53451750 | Turnover, pumpkin | Lo | Sweet Bakery Products |
| 53452100 | Pastry, fruit-filled | Lo | Sweet Bakery Products |
| 53452120 | Pastry, made with bean or lotus see | Lo | Sweet Bakery Products |
| 53452130 | Pastry, made with bean paste and s | Lo | Sweet Bakery Products |
| 53452150 | Pastry, Chinese, made with rice flo | Lo | Sweet Bakery Products |
| 53452170 | Pastry, cookie type, fried | Lo | Sweet Bakery Products |
| 53452200 | Pastry, Italian, with cheese | Lo | Sweet Bakery Products |
| 53452400 | Pastry, puff | Lo | Sweet Bakery Products |
| 53452420 | Pastry, puff, custard or cream filled | Lo | Sweet Bakery Products |
| 53452450 | Cheese pastry puffs | Lo | Sweet Bakery Products |

| 53452500 | Pastry, mainly flour and water, fried | Lo | Sweet Bakery Products |
| --- | --- | --- | --- |
| 53453150 | Empanada, Mexican turnover, fruit- | Lo | Sweet Bakery Products |
| 53453170 | Empanada, Mexican turnover, pum | Lo | Sweet Bakery Products |
| 53500100 | Breakfast pastry, NFS | Lo | Sweet Bakery Products |
| 53510000 | Danish pastry, plain or spice | Lo | Sweet Bakery Products |
| 53510100 | Danish pastry, with fruit | Lo | Sweet Bakery Products |
| 53510200 | Danish pastry, with nuts | Lo | Sweet Bakery Products |
| 53511000 | Danish pastry, with cheese | Lo | Sweet Bakery Products |
| 53511500 | Danish pastry, with cheese, fat free | Lo | Sweet Bakery Products |
| 53520000 | Doughnut, NFS | Lo | Sweet Bakery Products |
| 53520100 | Doughnut, cake type, plain | Lo | Sweet Bakery Products |
| 53520110 | Doughnut, cake type | Lo | Sweet Bakery Products |
| 53520120 | Doughnut, chocolate | Lo | Sweet Bakery Products |
| 53520130 | Doughnut, cake type, powdered su | Lo | Sweet Bakery Products |
| 53520135 | Doughnut, cake type, with icing | Lo | Sweet Bakery Products |
| 53520140 | Doughnut, cake type, chocolate icin | Lo | Sweet Bakery Products |
| 53520150 | Doughnut, cake type, chocolate cov | Lo | Sweet Bakery Products |
| 53520160 | Doughnut, chocolate, with chocolat | Lo | Sweet Bakery Products |
| 53520170 | Doughnut holes | Lo | Sweet Bakery Products |
| 53520200 | Churros | Lo | Sweet Bakery Products |
| 53520500 | Doughnut, Asian | Lo | Sweet Bakery Products |
| 53520510 | Beignet | Lo | Sweet Bakery Products |
| 53520600 | Cruller, NFS | Lo | Sweet Bakery Products |
| 53520700 | French cruller | Lo | Sweet Bakery Products |
| 53521100 | Doughnut, chocolate, raised or yea | Lo | Sweet Bakery Products |
| 53521110 | Doughnut, yeast type | Lo | Sweet Bakery Products |
| 53521120 | Doughnut, chocolate, raised or yea | Lo | Sweet Bakery Products |
| 53521130 | Doughnut, yeast type, with chocolat | Lo | Sweet Bakery Products |
| 53521140 | Doughnut, jelly | Lo | Sweet Bakery Products |
| 53521210 | Doughnut, custard-filled | Lo | Sweet Bakery Products |
| 53521220 | Doughnut, chocolate cream-filled | Lo | Sweet Bakery Products |
| 53521230 | Doughnut, custard-filled, with icing | Lo | Sweet Bakery Products |
| 53521250 | Doughnut, wheat | Lo | Sweet Bakery Products |
| 53521300 | Doughnut, wheat, chocolate covere | Lo | Sweet Bakery Products |
| 53530000 | Breakfast tart | Lo | Sweet Bakery Products |
| 53530010 | Breakfast tart, lowfat | Lo | Sweet Bakery Products |
| 53540000 | Breakfast bar, NFS | Lo | Snack/Meal Bars |
| 53540200 | Breakfast bar, cereal crust with fruit | Lo | Snack/Meal Bars |
| 53540250 | Breakfast bar, cereal crust with fruit | Lo | Snack/Meal Bars |
| 53540300 | Fiber One Chewy Bar | Lo | Snack/Meal Bars |
| 53540400 | Kellogg's Nutri-Grain Cereal Bar | Lo | Snack/Meal Bars |
| 53540402 | Kellogg's Nutri-Grain Yogurt Bar | Lo | Snack/Meal Bars |
| 53540404 | Kellogg's Nutri-Grain Fruit and Nut | Lo | Snack/Meal Bars |
| 53540500 | Breakfast bar, date, with yogurt coa | Lo | Snack/Meal Bars |
| 53540600 | Milk 'n Cereal bar | Lo | Snack/Meal Bars |
| 53540700 | Kellogg's Special K bar | Lo | Snack/Meal Bars |
| 53540800 | Kashi GOLEAN Chewy Bars | Lo | Snack/Meal Bars |
| 53540802 | Kashi TLC Chewy Granola Bar | Lo | Snack/Meal Bars |
| 53540804 | Kashi GOLEAN Crunchy Bars | Lo | Snack/Meal Bars |
| 53540806 | Kashi TLC Crunchy Granola Bar | Lo | Snack/Meal Bars |
| 53540900 | Nature Valley Chewy Trail Mix Gran | Lo | Snack/Meal Bars |

| 53540902 | Nature Valley Chewy Granola Bar w | Lo | Snack/Meal Bars |
| --- | --- | --- | --- |
| 53540904 | Nature Valley Sweet and Salty Nut | Lo | Snack/Meal Bars |
| 53540906 | Nature Valley Crunchy Granola Bar | Lo | Snack/Meal Bars |
| 53541000 | Quaker Chewy Granola Bar | Lo | Snack/Meal Bars |
| 53541002 | Quaker Chewy 90 Calorie Granola | Lo | Snack/Meal Bars |
| 53541004 | Quaker Chewy 25% Less Sugar Gr | Lo | Snack/Meal Bars |
| 53541006 | Quaker Chewy Dipps Granola Bar | Lo | Snack/Meal Bars |
| 53541200 | Meal replacement bar | Lo | Snack/Meal Bars |
| 53541300 | Slim Fast Original Meal Bar | Lo | Snack/Meal Bars |
| 53542000 | Snack bar, oatmeal | Lo | Snack/Meal Bars |
| 53542100 | Granola bar, NFS | Lo | Snack/Meal Bars |
| 53542200 | Granola bar, lowfat, NFS | Lo | Snack/Meal Bars |
| 53542210 | Granola bar, nonfat | Lo | Snack/Meal Bars |
| 53543000 | Granola bar, reduced sugar, NFS | Lo | Snack/Meal Bars |
| 53543100 | Granola bar, peanuts, oats, sugar, | Lo | Snack/Meal Bars |
| 53544200 | Granola bar, chocolate-coated, NF | Lo | Snack/Meal Bars |
| 53544210 | Granola bar, with coconut, chocolat | Lo | Snack/Meal Bars |
| 53544220 | Granola bar with nuts, chocolate-co | Lo | Snack/Meal Bars |
| 53544230 | Granola bar, oats, nuts, coated with | Lo | Snack/Meal Bars |
| 53544250 | Granola bar, coated with non-choco | Lo | Snack/Meal Bars |
| 53544300 | Granola bar, high fiber, coated with | Lo | Snack/Meal Bars |
| 53544400 | Granola bar, with rice cereal | Lo | Snack/Meal Bars |
| 53544410 | Quaker Granola Bites | Lo | Snack/Meal Bars |
| 53544450 | PowerBar (fortified high energy bar | Lo | Snack/Meal Bars |
| 53610000 | Coffee cake, NFS | Lo | Sweet Bakery Products |
| 53610100 | Coffee cake, crumb or quick-bread | Lo | Sweet Bakery Products |
| 53610120 | Coffee cake, crumb or quick-bread | Lo | Sweet Bakery Products |
| 53610170 | Coffee cake, crumb or quick-bread | Lo | Sweet Bakery Products |
| 53610200 | Coffee cake, crumb or quick-bread | Lo | Sweet Bakery Products |
| 53610250 | Coffee cake, crumb or quick-bread | Lo | Sweet Bakery Products |
| 53710400 | Cereal or granola bar (General Mill | Lo | Snack/Meal Bars |
| 53710500 | Cereal or granola bar (Kellogg's Nu | Lo | Snack/Meal Bars |
| 53710502 | Cereal or granola bar (Kellogg's Nu | Lo | Snack/Meal Bars |
| 53710504 | Cereal or granola bar (Kellogg's Nu | Lo | Snack/Meal Bars |
| 53710600 | Milk 'n Cereal bar | Lo | Snack/Meal Bars |
| 53710700 | Cereal or granola bar (Kellogg's Sp | Lo | Snack/Meal Bars |
| 53710800 | Cereal or granola bar (Kashi Chewy | Lo | Snack/Meal Bars |
| 53710802 | Cereal or granola bar (Kashi Crunc | Lo | Snack/Meal Bars |
| 53710804 | Kashi GOLEAN Crunchy Bars | Lo | Snack/Meal Bars |
| 53710806 | Kashi TLC Crunchy Granola Bar | Lo | Snack/Meal Bars |
| 53710810 | Cereal or granola bar (KIND Fruit a | Lo | Snack/Meal Bars |
| 53710900 | Cereal or granola bar (General Mill | Lo | Snack/Meal Bars |
| 53710902 | Cereal or granola bar, with yogurt c | Lo | Snack/Meal Bars |
| 53710904 | Cereal or granola bar (General Mill | Lo | Snack/Meal Bars |
| 53710906 | Cereal or granola bar (General Mill | Lo | Snack/Meal Bars |
| 53711000 | Cereal or granola bar (Quaker Che | Lo | Snack/Meal Bars |
| 53711002 | Cereal or granola bar (Quaker Che | Lo | Snack/Meal Bars |
| 53711004 | Cereal or granola bar (Quaker Che | Lo | Snack/Meal Bars |
| 53711006 | Cereal or granola bar (Quaker Che | Lo | Snack/Meal Bars |
| 53711100 | Cereal or granola bar (Quaker Gran | Lo | Snack/Meal Bars |
| 53712000 | Snack bar, oatmeal | Lo | Snack/Meal Bars |

| 53712100 | Cereal or Granola bar, NFS | Lo | Snack/Meal Bars |
| --- | --- | --- | --- |
| 53712200 | Cereal or granola bar, lowfat, NFS | Lo | Snack/Meal Bars |
| 53712210 | Cereal or granola bar, nonfat | Lo | Snack/Meal Bars |
| 53713010 | Cereal or granola bar, fruit and nut | Lo | Snack/Meal Bars |
| 53713100 | Cereal or granola bar, peanuts , oa | Lo | Snack/Meal Bars |
| 53714200 | Cereal or granola bar, chocolate co | Lo | Snack/Meal Bars |
| 53714210 | Cereal or granola bar, with coconut | Lo | Snack/Meal Bars |
| 53714220 | Cereal or granola bar with nuts, cho | Lo | Snack/Meal Bars |
| 53714230 | Cereal or granola bar, oats, nuts, c | Lo | Snack/Meal Bars |
| 53714250 | Cereal or granola bar, coated with n | Lo | Snack/Meal Bars |
| 53714300 | Cereal or granola bar, high fiber, co | Lo | Snack/Meal Bars |
| 53714400 | Cereal or granola bar, with rice cere | Lo | Snack/Meal Bars |
| 53714500 | Breakfast bar, NFS | Lo | Snack/Meal Bars |
| 53714520 | Breakfast bar, cereal crust with fruit | Lo | Snack/Meal Bars |
| 53720100 | Nutrition bar (Balance Original Bar) | Lo | Snack/Meal Bars |
| 53720200 | Nutrition bar (Clif Bar) | Lo | Snack/Meal Bars |
| 53720210 | Nutrition bar (Clif Kids Organic Zba | Lo | Snack/Meal Bars |
| 53720300 | Nutrition bar (PowerBar) | Lo | Snack/Meal Bars |
| 53720400 | Nutrition bar (Slim Fast Original Me | Lo | Snack/Meal Bars |
| 53720500 | Nutrition bar (Snickers Marathon Pr | Lo | Snack/Meal Bars |
| 53720600 | Nutrition bar (South Beach Living M | Lo | Snack/Meal Bars |
| 53720610 | Nutrition bar (South Beach Living H | Lo | Snack/Meal Bars |
| 53720700 | Nutrition bar (Tiger's Milk) | Lo | Snack/Meal Bars |
| 53720800 | Nutrition bar (Zone Perfect Classic | Lo | Snack/Meal Bars |
| 53729000 | Nutrition bar or meal replacement b | Lo | Snack/Meal Bars |
| 53801000 | Cereal bar with fruit filling, baby foo | Lo | Baby Foods |
| 53803050 | Cookie, fruit, baby food | Lo | Baby Foods |
| 53803100 | Cookie, baby food | Lo | Baby Foods |
| 53803250 | Cookie, teething, baby | Lo | Baby Foods |
| 53803300 | Cookie, rice, baby | Lo | Baby Foods |
| 54001000 | Crackers, NFS | Lo | Crackers |
| 54101010 | Cracker, animal | Lo | Sweet Bakery Products |
| 54102010 | Graham crackers | Lo | Sweet Bakery Products |
| 54102015 | Graham crackers (Teddy Grahams | Lo | Sweet Bakery Products |
| 54102020 | Graham crackers, chocolate covere | Lo | Sweet Bakery Products |
| 54102050 | Crackers, oatmeal | Lo | Crackers |
| 54102060 | Crackers, Cuban | Lo | Sweet Bakery Products |
| 54102100 | Graham crackers, reduced fat | Lo | Sweet Bakery Products |
| 54102110 | Crackers, graham, fat free | Lo | Sweet Bakery Products |
| 54102200 | Graham crackers, sandwich, with fi | Lo | Sweet Bakery Products |
| 54103000 | Crackers, breakfast biscuit | Lo | Crackers |
| 54200100 | Crackers, butter, reduced sodium | Lo | Crackers |
| 54201010 | Crackers, matzo, reduced sodium | Lo | Crackers |
| 54202010 | Crackers, saltine, low sodium | Lo | Crackers |
| 54202020 | Crackers, saltine, reduced sodium | Lo | Crackers |
| 54202050 | Crackers, saltine, fat free, low sodiu | Lo | Crackers |
| 54203010 | Crackers, toast thins (rye, wheat, w | Lo | Crackers |
| 54204010 | Cracker, 100% whole wheat, low so | Lo | Crackers |
| 54204020 | Crackers, wheat, reduced sodium | Lo | Crackers |
| 54204030 | Crackers, woven wheat, reduced s | Lo | Crackers |
| 54205010 | Cracker, snack, low sodium | Lo | Crackers |

| 54205030 | Cracker, cheese, low sodium | Lo | Crackers |
| --- | --- | --- | --- |
| 54205100 | Cracker, snack, reduced fat, reduc | Lo | Crackers |
| 54206010 | Puffed rice cake without salt | Lo | Crackers |
| 54207010 | Crispbread, wheat, low sodium | Lo | Crackers |
| 54210010 | Cracker, multigrain, low sodium | Lo | Crackers |
| 54222000 | Crispbread, rye, low sodium | Lo | Crackers |
| 54301000 | Cracker, snack | Lo | Crackers |
| 54301010 | Crackers, butter, plain | Lo | Crackers |
| 54301020 | Crackers, butter, flavored | Lo | Crackers |
| 54301030 | Crackers, butter (Ritz) | Lo | Crackers |
| 54301100 | Crackers, butter, reduced fat | Lo | Crackers |
| 54301200 | Cracker, snack, fat free | Lo | Crackers |
| 54304000 | Crackers, cheese | Lo | Crackers |
| 54304005 | Crackers, cheese (Cheez-It) | Lo | Crackers |
| 54304020 | Crackers, cheese (Goldfish) | Lo | Crackers |
| 54304100 | Crackers, cheese, reduced fat | Lo | Crackers |
| 54304110 | Crackers, cheese, reduced sodium | Lo | Crackers |
| 54304150 | Crackers, cheese, whole grain | Lo | Crackers |
| 54304500 | Cracker, high fiber, no added fat | Lo | Crackers |
| 54305000 | Crispbread, wheat, no added fat | Lo | Crackers |
| 54305010 | Crackers, crispbread | Lo | Crackers |
| 54305020 | Crackers, flatbread | Lo | Crackers |
| 54305500 | Crispbread, wheat or rye, extra cris | Lo | Crackers |
| 54307000 | Crackers, matzo | Lo | Crackers |
| 54308000 | Crackers, milk | Lo | Crackers |
| 54309000 | Crackers, oat | Lo | Crackers |
| 54313000 | Crackers, oyster | Lo | Crackers |
| 54318000 | Chips, rice | Lo | Savory Snacks |
| 54318500 | Rice cake | Lo | Crackers |
| 54319000 | Crackers, rice | Lo | Crackers |
| 54319005 | Crackers, rice and nuts | Lo | Crackers |
| 54319010 | Puffed rice cake | Lo | Crackers |
| 54319020 | Popcorn cake | Lo | Crackers |
| 54319500 | Rice paper | Lo | Crackers |
| 54322000 | Crispbread, rye, no added fat | Lo | Crackers |
| 54325000 | Crackers, saltine | Lo | Crackers |
| 54325010 | Crackers, saltine, reduced fat | Lo | Crackers |
| 54325050 | Crackers, saltine, whole wheat | Lo | Crackers |
| 54325060 | Crackers, saltine, multigrain | Lo | Crackers |
| 54326000 | Crackers, multigrain | Lo | Crackers |
| 54327950 | Crackers, cylindrical, peanut-butter | Lo | Crackers |
| 54328000 | Crackers, sandwich | Lo | Crackers |
| 54328100 | Crackers, sandwich, peanut butter f | Lo | Crackers |
| 54328105 | Crackers, sandwich, peanut butter f | Lo | Crackers |
| 54328110 | Crackers, sandwich, reduced fat, p | Lo | Crackers |
| 54328120 | Crackers, whole grain, sandwich, p | Lo | Crackers |
| 54328200 | Crackers, sandwich, cheese filled | Lo | Crackers |
| 54328210 | Crackers, sandwich, cheese filled ( | Lo | Crackers |
| 54334000 | Crackers, toast thins (rye, pumpern | Lo | Crackers |
| 54336000 | Crackers, water | Lo | Crackers |
| 54336100 | Crackers, wonton | Lo | Crackers |

| 54337000 | Cracker, 100% whole wheat | Lo | Crackers |
| --- | --- | --- | --- |
| 54337010 | Crackers, woven wheat | Lo | Crackers |
| 54337020 | Crackers, woven wheat, plain (Trisc | Lo | Crackers |
| 54337030 | Crackers, woven wheat, flavored (T | Lo | Crackers |
| 54337050 | Cracker, 100% whole wheat, reduc | Lo | Crackers |
| 54337060 | Crackers, woven wheat, reduced fa | Lo | Crackers |
| 54337100 | Crackers, whole wheat and bran | Lo | Crackers |
| 54338000 | Crackers, wheat | Lo | Crackers |
| 54338010 | Crackers, wheat, plain (Wheat Thin | Lo | Crackers |
| 54338020 | Crackers, wheat, flavored (Wheat T | Lo | Crackers |
| 54338100 | Crackers, wheat, reduced fat | Lo | Crackers |
| 54339000 | Crackers, corn | Lo | Crackers |
| 54340100 | Crackers, gluten free, plain | Lo | Crackers |
| 54340110 | Crackers, gluten free, flavored | Lo | Crackers |
| 54350000 | Crackers, baby food | Lo | Baby Foods |
| 54350010 | Gerber Finger Foods, Puffs, baby f | Lo | Baby Foods |
| 54350020 | Finger Foods, Puffs, baby food | Lo | Baby Foods |
| 54360000 | Crunchy snacks, corn based, baby | Lo | Baby Foods |
| 54401010 | Salty snacks, corn or cornmeal bas | Lo | Savory Snacks |
| 54401011 | Corn nuts | Lo | Savory Snacks |
| 54401020 | Salty snacks, corn or cornmeal bas | Lo | Savory Snacks |
| 54401021 | Corn chips, plain | Lo | Savory Snacks |
| 54401026 | Corn chips, flavored | Lo | Savory Snacks |
| 54401031 | Corn chips, plain (Fritos) | Lo | Savory Snacks |
| 54401035 | Corn chips, flavored (Fritos) | Lo | Savory Snacks |
| 54401050 | Salty snacks, corn or cornmeal bas | Lo | Savory Snacks |
| 54401055 | Cheese flavored corn snacks | Lo | Savory Snacks |
| 54401065 | Cheese flavored corn snacks, redu | Lo | Savory Snacks |
| 54401075 | Tortilla chips, plain | Lo | Savory Snacks |
| 54401080 | Salty snacks, corn or cornmeal bas | Lo | Savory Snacks |
| 54401081 | Cheese flavored corn snacks (Che | Lo | Savory Snacks |
| 54401085 | Tortilla chips, flavored | Lo | Savory Snacks |
| 54401090 | Corn chips, reduced sodium | Lo | Savory Snacks |
| 54401100 | Salty snacks, corn or cornmeal bas | Lo | Savory Snacks |
| 54401110 | Tortilla chips, nacho cheese flavor ( | Lo | Savory Snacks |
| 54401111 | Tortilla chips, cool ranch flavor (Dor | Lo | Savory Snacks |
| 54401112 | Tortilla chips, other flavors (Doritos | Lo | Savory Snacks |
| 54401120 | Salty snacks, corn or cornmeal bas | Lo | Savory Snacks |
| 54401121 | Tortilla chips, reduced fat, plain | Lo | Savory Snacks |
| 54401122 | Tortilla chips, reduced fat, flavored | Lo | Savory Snacks |
| 54401150 | Salty snacks, corn or cornmeal bas | Lo | Savory Snacks |
| 54401170 | Tortilla chips, low fat, unsalted | Lo | Savory Snacks |
| 54401210 | Salty snacks, corn based puffs and | Lo | Savory Snacks |
| 54402080 | Tortilla chips, reduced sodium | Lo | Savory Snacks |
| 54402200 | Snack mix | Lo | Savory Snacks |
| 54402500 | Salty snacks, wheat- and corn-base | Lo | Savory Snacks |
| 54402600 | Salty snacks, multigrain, whole grai | Lo | Savory Snacks |
| 54402610 | Potato chips, restructured, multigra | Lo | Savory Snacks |
| 54402700 | Pita chips | Lo | Crackers |
| 54403000 | Popcorn, popped in oil, unbuttered | Lo | Savory Snacks |
| 54403001 | Popcorn, NFS | Lo | Savory Snacks |

| 54403005 | Popcorn, movie theater, with added | Lo | Savory Snacks |
| --- | --- | --- | --- |
| 54403006 | Popcorn, movie theater, unbuttered | Lo | Savory Snacks |
| 54403010 | Popcorn, air-popped, unbuttered | Lo | Savory Snacks |
| 54403020 | Popcorn, popped in oil, buttered | Lo | Savory Snacks |
| 54403040 | Popcorn, air-popped, with added bu | Lo | Savory Snacks |
| 54403045 | Popcorn, popped in oil, unbuttered | Lo | Savory Snacks |
| 54403046 | Popcorn, popped in oil, with added | Lo | Savory Snacks |
| 54403050 | Popcorn, flavored | Lo | Savory Snacks |
| 54403051 | Popcorn, microwave, NFS | Lo | Savory Snacks |
| 54403052 | Popcorn, microwave, plain | Lo | Savory Snacks |
| 54403053 | Popcorn, microwave, plain, light | Lo | Savory Snacks |
| 54403054 | Popcorn, microwave, low sodium | Lo | Savory Snacks |
| 54403055 | Popcorn, microwave, unsalted | Lo | Savory Snacks |
| 54403056 | Popcorn, microwave, butter flavore | Lo | Savory Snacks |
| 54403057 | Popcorn, microwave, butter flavore | Lo | Savory Snacks |
| 54403058 | Popcorn, microwave, cheese flavor | Lo | Savory Snacks |
| 54403059 | Popcorn, microwave, kettle corn | Lo | Savory Snacks |
| 54403060 | Popcorn, popped in oil, lowfat, redu | Lo | Savory Snacks |
| 54403061 | Popcorn, microwave, kettle corn, lig | Lo | Savory Snacks |
| 54403062 | Popcorn, microwave, other flavored | Lo | Savory Snacks |
| 54403070 | Popcorn, popped in oil, lowfat | Lo | Savory Snacks |
| 54403080 | Popcorn, ready-to-eat packaged, N | Lo | Savory Snacks |
| 54403081 | Popcorn, ready-to-eat packaged, pl | Lo | Savory Snacks |
| 54403082 | Popcorn, ready-to-eat packaged, pl | Lo | Savory Snacks |
| 54403083 | Popcorn, ready-to-eat packaged, lo | Lo | Savory Snacks |
| 54403085 | Popcorn, ready-to-eat packaged, b | Lo | Savory Snacks |
| 54403086 | Popcorn, ready-to-eat packaged, b | Lo | Savory Snacks |
| 54403087 | Popcorn, ready-to-eat packaged, c | Lo | Savory Snacks |
| 54403088 | Popcorn, ready-to-eat packaged, c | Lo | Savory Snacks |
| 54403089 | Popcorn, ready-to-eat-packaged, k | Lo | Savory Snacks |
| 54403090 | Popcorn, popped in oil, unsalted | Lo | Savory Snacks |
| 54403091 | Popcorn, ready-to-eat packaged, k | Lo | Savory Snacks |
| 54403092 | Popcorn, ready-to-eat packaged, ot | Lo | Savory Snacks |
| 54403110 | Popcorn, caramel coated | Lo | Savory Snacks |
| 54403120 | Popcorn, caramel coated, with nuts | Lo | Savory Snacks |
| 54403150 | Popcorn, sugar syrup or caramel-c | Lo | Savory Snacks |
| 54403160 | Popcorn, chocolate coated | Lo | Savory Snacks |
| 54404000 | Popcorn chips, plain | Lo | Savory Snacks |
| 54404010 | Popcorn chips, other flavors | Lo | Savory Snacks |
| 54404020 | Popcorn chips, sweet flavors | Lo | Savory Snacks |
| 54406010 | Onion flavored rings | Lo | Savory Snacks |
| 54406200 | Shrimp chips | Lo | Savory Snacks |
| 54408000 | Pretzels, NFS | Lo | Savory Snacks |
| 54408010 | Pretzels, hard | Lo | Savory Snacks |
| 54408015 | Pretzels, hard, NFS | Lo | Savory Snacks |
| 54408016 | Pretzels, hard, plain, salted | Lo | Savory Snacks |
| 54408017 | Pretzels, hard, plain, lightly salted | Lo | Savory Snacks |
| 54408020 | Pretzels, soft | Lo | Savory Snacks |
| 54408030 | Pretzels, hard, plain, unsalted | Lo | Savory Snacks |
| 54408035 | Pretzels, hard, flavored | Lo | Savory Snacks |
| 54408040 | Pretzels, soft, unsalted | Lo | Savory Snacks |

| 54408070 | Pretzels, hard, multigrain | Lo | Savory Snacks |
| --- | --- | --- | --- |
| 54408080 | Pretzel, gluten free | Lo | Savory Snacks |
| 54408081 | Pretzels, hard, plain, gluten free | Lo | Savory Snacks |
| 54408082 | Pretzels, hard, flavored, gluten free | Lo | Savory Snacks |
| 54408105 | Pretzel chips, hard, plain | Lo | Savory Snacks |
| 54408110 | Pretzel chips, hard, flavored | Lo | Savory Snacks |
| 54408190 | Pretzels, hard, coated, NFS | Lo | Savory Snacks |
| 54408200 | Pretzels, hard, chocolate coated | Lo | Savory Snacks |
| 54408210 | Pretzels, hard, white chocolate coat | Lo | Savory Snacks |
| 54408250 | Pretzels, hard, yogurt coated | Lo | Savory Snacks |
| 54408290 | Pretzels, hard, filled, NFS | Lo | Savory Snacks |
| 54408300 | Pretzels, hard, cheese filled | Lo | Savory Snacks |
| 54408310 | Pretzels, hard, peanut butter filled | Lo | Savory Snacks |
| 54408400 | Pretzels, soft, NFS | Lo | Savory Snacks |
| 54408405 | Pretzels, soft, ready-to-eat, NFS | Lo | Savory Snacks |
| 54408410 | Pretzels, soft, ready-to-eat, salted, | Lo | Savory Snacks |
| 54408411 | Pretzels, soft, ready-to-eat, unsalte | Lo | Savory Snacks |
| 54408415 | Pretzels, soft, ready-to-eat, salted, | Lo | Savory Snacks |
| 54408416 | Pretzels, soft, ready-to-eat, unsalte | Lo | Savory Snacks |
| 54408420 | Pretzels, soft, ready-to-eat, cinnam | Lo | Savory Snacks |
| 54408422 | Pretzels, soft, ready-to-eat, coated | Lo | Savory Snacks |
| 54408430 | Pretzels, soft, ready-to-eat, topped | Lo | Savory Snacks |
| 54408432 | Pretzels, soft, ready-to-eat, topped | Lo | Savory Snacks |
| 54408456 | Pretzels, soft, from frozen, unsalted | Lo | Savory Snacks |
| 54408470 | Pretzels, soft, filled with cheese | Lo | Savory Snacks |
| 54408475 | Pretzels, soft, from school lunch | Lo | Savory Snacks |
| 54408480 | Pretzels, soft, multigrain | Lo | Savory Snacks |
| 54408485 | Pretzels, soft, gluten free | Lo | Savory Snacks |
| 54408487 | Pretzels, soft, gluten free, coated o | Lo | Savory Snacks |
| 54420010 | Multigrain mixture, pretzels, cereal | Lo | Savory Snacks |
| 54420100 | Oriental party mix, with peanuts, se | Lo | Savory Snacks |
| 54420200 | Multigrain mixture, bread sticks, se | Lo | Savory Snacks |
| 54420210 | Multigrain chips (Sun Chips) | Lo | Savory Snacks |
| 54420220 | Snack mix, plain (Chex Mix) | Lo | Savory Snacks |
| 54430010 | Yogurt chips | Lo | Crackers |
| 54440010 | Bagel chips | Lo | Crackers |
| 54440020 | Cracker chips | Lo | Savory Snacks |
| 55100005 | Pancakes, NFS | Lo | Quick Breads and Bread Products |
| 55100010 | Pancakes, plain, from frozen | Lo | Quick Breads and Bread Products |
| 55100015 | Pancakes, plain, reduced fat, from | Lo | Quick Breads and Bread Products |
| 55100020 | Pancakes, with fruit, from frozen | Lo | Quick Breads and Bread Products |
| 55100025 | Pancakes, with chocolate, from froz | Lo | Quick Breads and Bread Products |
| 55100030 | Pancakes, whole grain, from frozen | Lo | Quick Breads and Bread Products |
| 55100035 | Pancakes, whole grain, reduced fat | Lo | Quick Breads and Bread Products |
| 55100040 | Pancakes, gluten free, from frozen | Lo | Quick Breads and Bread Products |
| 55100050 | Pancakes, plain, from fast food / re | Lo | Quick Breads and Bread Products |
| 55100055 | Pancakes, with fruit, from fast food | Lo | Quick Breads and Bread Products |
| 55100060 | Pancakes, with chocolate, from fas | Lo | Quick Breads and Bread Products |
| 55100065 | Pancakes, whole grain, from fast fo | Lo | Quick Breads and Bread Products |
| 55100080 | Pancakes, from school, NFS | Lo | Quick Breads and Bread Products |
| 55101000 | Pancakes, plain | Lo | Quick Breads and Bread Products |

| 55101010 | Pancakes, reduced calorie, high fib | Lo | Quick Breads and Bread Products |
| --- | --- | --- | --- |
| 55101015 | Pancakes, plain, reduced fat | Lo | Quick Breads and Bread Products |
| 55101020 | Pancakes, plain, fat free | Lo | Quick Breads and Bread Products |
| 55103000 | Pancakes, with fruit | Lo | Quick Breads and Bread Products |
| 55103020 | Pancakes, pumpkin | Lo | Quick Breads and Bread Products |
| 55103100 | Pancakes, with chocolate | Lo | Quick Breads and Bread Products |
| 55105000 | Pancakes, buckwheat | Lo | Quick Breads and Bread Products |
| 55105100 | Pancakes, cornmeal | Lo | Quick Breads and Bread Products |
| 55105200 | Pancakes, whole grain | Lo | Quick Breads and Bread Products |
| 55105205 | Pancakes, whole grain, reduced fat | Lo | Quick Breads and Bread Products |
| 55105210 | Pancakes, whole wheat, fat free | Lo | Quick Breads and Bread Products |
| 55105300 | Pancakes, sour dough | Lo | Quick Breads and Bread Products |
| 55105400 | Pancakes, rye | Lo | Quick Breads and Bread Products |
| 55106000 | Pancakes, gluten free | Lo | Quick Breads and Bread Products |
| 55200010 | Waffle, NFS | Lo | Quick Breads and Bread Products |
| 55200020 | Waffle, plain, from frozen | Lo | Quick Breads and Bread Products |
| 55200030 | Waffle, plain, reduced fat, from froz | Lo | Quick Breads and Bread Products |
| 55200040 | Waffle, fruit, from frozen | Lo | Quick Breads and Bread Products |
| 55200050 | Waffle, chocolate, from frozen | Lo | Quick Breads and Bread Products |
| 55200060 | Waffle, whole grain, from frozen | Lo | Quick Breads and Bread Products |
| 55200070 | Waffle, whole grain, reduced fat, fro | Lo | Quick Breads and Bread Products |
| 55200080 | Waffle, whole grain, fruit, from froze | Lo | Quick Breads and Bread Products |
| 55200090 | Waffle, gluten free, from frozen | Lo | Quick Breads and Bread Products |
| 55200100 | Waffle, plain, from fast food / resta | Lo | Quick Breads and Bread Products |
| 55200110 | Waffle, chocolate, from fast food / r | Lo | Quick Breads and Bread Products |
| 55200120 | Waffle, fruit, from fast food / restau | Lo | Quick Breads and Bread Products |
| 55200130 | Waffle, whole grain, from fast food | Lo | Quick Breads and Bread Products |
| 55200200 | Waffle, from school, NFS | Lo | Quick Breads and Bread Products |
| 55201000 | Waffle, plain | Lo | Quick Breads and Bread Products |
| 55202000 | Waffle, wheat, bran, or multigrain | Lo | Quick Breads and Bread Products |
| 55203000 | Waffle, fruit | Lo | Quick Breads and Bread Products |
| 55203500 | Waffle, nut and honey | Lo | Quick Breads and Bread Products |
| 55203600 | Waffle, chocolate | Lo | Quick Breads and Bread Products |
| 55203700 | Waffle, cinnamon | Lo | Quick Breads and Bread Products |
| 55204000 | Waffle, cornmeal | Lo | Quick Breads and Bread Products |
| 55205000 | Waffle, whole grain | Lo | Quick Breads and Bread Products |
| 55206000 | Waffle, oat bran | Lo | Quick Breads and Bread Products |
| 55207000 | Waffle, multi-bran | Lo | Quick Breads and Bread Products |
| 55208000 | Waffle, gluten free | Lo | Quick Breads and Bread Products |
| 55211000 | Waffle, plain, fat free | Lo | Quick Breads and Bread Products |
| 55211050 | Waffle, plain, reduced fat | Lo | Quick Breads and Bread Products |
| 55212000 | Waffle, whole grain, reduced fat | Lo | Quick Breads and Bread Products |
| 55300010 | French toast, NFS | Lo | Quick Breads and Bread Products |
| 55300020 | French toast, plain, from frozen | Lo | Quick Breads and Bread Products |
| 55300050 | French toast, plain, from fast food / | Lo | Quick Breads and Bread Products |
| 55300055 | French toast, whole grain, from fast | Lo | Quick Breads and Bread Products |
| 55300060 | French toast, from school, NFS | Lo | Quick Breads and Bread Products |
| 55301000 | French toast, plain | Lo | Quick Breads and Bread Products |
| 55301015 | French toast, whole grain | Lo | Quick Breads and Bread Products |
| 55301020 | French toast, whole grain, reduced | Lo | Quick Breads and Bread Products |
| 55301025 | French toast, gluten free | Lo | Quick Breads and Bread Products |

| 55301030 | French toast sticks, NFS | Lo | Quick Breads and Bread Products |
| --- | --- | --- | --- |
| 55301031 | French toast sticks, plain, from froz | Lo | Quick Breads and Bread Products |
| 55301040 | French toast sticks, plain, from fast | Lo | Quick Breads and Bread Products |
| 55301048 | French toast sticks, from school, N | Lo | Quick Breads and Bread Products |
| 55301050 | French toast sticks, plain | Lo | Quick Breads and Bread Products |
| 55301055 | French toast sticks, whole grain | Lo | Quick Breads and Bread Products |
| 55310100 | Fried bread, Puerto Rican style | Lo | Quick Breads and Bread Products |
| 55401000 | Crepe, plain | Lo | Quick Breads and Bread Products |
| 55501000 | Chinese pancake | Lo | Quick Breads and Bread Products |
| 55502000 | Flour and water gravy | Lo | Condiments and Sauces |
| 55610200 | Dumpling, fried, Puerto Rican style | Lo | Mixed Dishes - Grain-based |
| 55610300 | Dumpling, plain | Lo | Quick Breads and Bread Products |
| 55701000 | Cake made with glutinous rice | Lo | Mixed Dishes - Grain-based |
| 55702000 | Cake or pancake made with rice flo | Lo | Mixed Dishes - Grain-based |
| 55702100 | Dosa (Indian), plain | Lo | Quick Breads and Bread Products |
| 55703000 | Cake made with glutinous rice and | Lo | Mixed Dishes - Grain-based |
| 55801000 | Funnel cake with sugar | Lo | Sweet Bakery Products |
| 55801010 | Funnel cake with sugar and fruit | Lo | Sweet Bakery Products |
| 56101000 | Macaroni, cooked, NS as to fat add | Lo | Cooked Grains |
| 56101010 | Macaroni, cooked, fat not added in | Lo | Cooked Grains |
| 56101030 | Macaroni, cooked, fat added in coo | Lo | Cooked Grains |
| 56102000 | Macaroni, whole wheat, cooked, NS | Lo | Cooked Grains |
| 56102010 | Macaroni, whole wheat, cooked, fat | Lo | Cooked Grains |
| 56102020 | Macaroni, whole wheat, cooked, fat | Lo | Cooked Grains |
| 56103010 | Macaroni, cooked, spinach, fat not | Lo | Cooked Grains |
| 56104000 | Pasta, vegetable, cooked | Lo | Cooked Grains |
| 56104010 | Macaroni, cooked, vegetable, fat no | Lo | Cooked Grains |
| 56104020 | Macaroni, cooked, vegetable, fat ad | Lo | Cooked Grains |
| 56112000 | Noodles, cooked | Lo | Cooked Grains |
| 56112010 | Noodles, cooked, fat not added in c | Lo | Cooked Grains |
| 56112030 | Noodles, cooked, fat added in cook | Lo | Cooked Grains |
| 56113000 | Noodles, whole grain, cooked | Lo | Cooked Grains |
| 56113010 | Noodles, cooked, whole wheat, fat | Lo | Cooked Grains |
| 56113990 | Noodles, vegetable, cooked | Lo | Cooked Grains |
| 56114000 | Noodles, cooked, spinach, fat not a | Lo | Cooked Grains |
| 56114020 | Noodles, cooked, spinach, fat adde | Lo | Cooked Grains |
| 56116000 | Noodles, chow mein | Lo | Crackers |
| 56116990 | Long rice noodles, made from mun | Lo | Cooked Grains |
| 56117000 | Long rice noodles (made from mun | Lo | Cooked Grains |
| 56117010 | Long rice noodles (made from mun | Lo | Cooked Grains |
| 56117090 | Rice noodles, cooked | Lo | Cooked Grains |
| 56117100 | Chow fun rice noodles, cooked, fat | Lo | Cooked Grains |
| 56117110 | Chow fun rice noodles, cooked, fat | Lo | Cooked Grains |
| 56130000 | Pasta, cooked | Lo | Cooked Grains |
| 56130010 | Spaghetti, cooked, fat not added in | Lo | Cooked Grains |
| 56131000 | Spaghetti, cooked, fat added in coo | Lo | Cooked Grains |
| 56132990 | Pasta, whole grain, cooked | Lo | Cooked Grains |
| 56133000 | Spaghetti, cooked, whole wheat, fat | Lo | Cooked Grains |
| 56133010 | Spaghetti, cooked, whole wheat, fat | Lo | Cooked Grains |
| 56140100 | Pasta, gluten free | Lo | Cooked Grains |
| 56200300 | Cereal, cooked, NFS | Lo | Cooked Cereals |

| 56200350 | Cereal, cooked, instant, NS as to g | Lo | Cooked Cereals |
| --- | --- | --- | --- |
| 56200390 | Barley, NS as to fat | Lo | Cooked Grains |
| 56200400 | Barley, no added fat | Lo | Cooked Grains |
| 56200490 | Buckwheat groats, NS as to fat | Lo | Cooked Grains |
| 56200500 | Buckwheat groats, no added fat | Lo | Cooked Grains |
| 56200510 | Buckwheat groats, fat added | Lo | Cooked Grains |
| 56200990 | Grits, NS as to regular, quick, or ins | Lo | Cooked Cereals |
| 56201000 | Grits, NS as to regular, quick, or ins | Lo | Cooked Cereals |
| 56201010 | Grits, cooked, corn or hominy, regu | Lo | Cooked Cereals |
| 56201020 | Grits, cooked, corn or hominy, regu | Lo | Cooked Cereals |
| 56201030 | Grits, cooked, corn or hominy, regu | Lo | Cooked Cereals |
| 56201040 | Grits, NS as to regular, quick, or ins | Lo | Cooked Cereals |
| 56201050 | Grits, regular or quick, made with w | Lo | Cooked Cereals |
| 56201051 | Grits, regular or quick, made with w | Lo | Cooked Cereals |
| 56201052 | Grits, regular or quick, made with w | Lo | Cooked Cereals |
| 56201055 | Grits, regular or quick, made with m | Lo | Cooked Cereals |
| 56201056 | Grits, regular or quick, made with m | Lo | Cooked Cereals |
| 56201057 | Grits, regular or quick, made with m | Lo | Cooked Cereals |
| 56201060 | Grits, cooked, corn or hominy, with | Lo | Cooked Cereals |
| 56201061 | Grits, cooked, corn or hominy, with | Lo | Cooked Cereals |
| 56201062 | Grits, cooked, corn or hominy, with | Lo | Cooked Cereals |
| 56201070 | Grits, cooked, corn or hominy, with | Lo | Cooked Cereals |
| 56201071 | Grits, cooked, corn or hominy, with | Lo | Cooked Cereals |
| 56201072 | Grits, cooked, corn or hominy, with | Lo | Cooked Cereals |
| 56201081 | Grits, cooked, corn or hominy, with | Lo | Cooked Cereals |
| 56201082 | Grits, cooked, corn or hominy, with | Lo | Cooked Cereals |
| 56201090 | Grits, with cheese, NS as to fat | Lo | Cooked Cereals |
| 56201091 | Grits, with cheese, no added fat | Lo | Cooked Cereals |
| 56201092 | Grits, with cheese, fat added | Lo | Cooked Cereals |
| 56201110 | Grits, cooked, corn or hominy, quic | Lo | Cooked Cereals |
| 56201120 | Grits, cooked, corn or hominy, quic | Lo | Cooked Cereals |
| 56201130 | Grits, cooked, corn or hominy, quic | Lo | Cooked Cereals |
| 56201210 | Grits, instant, made with water, no | Lo | Cooked Cereals |
| 56201220 | Grits, instant, made with water, fat | Lo | Cooked Cereals |
| 56201230 | Grits, instant, made with water, NS | Lo | Cooked Cereals |
| 56201240 | Grits, cooked, flavored, corn or hom | Lo | Cooked Cereals |
| 56201298 | Grits, cooked, corn or hominy, NS a | Lo | Cooked Cereals |
| 56201300 | Grits, cooked, corn or hominy, NS a | Lo | Cooked Cereals |
| 56201320 | Grits, cooked, corn or hominy, regu | Lo | Cooked Cereals |
| 56201322 | Grits, cooked, corn or hominy, regu | Lo | Cooked Cereals |
| 56201324 | Grits, cooked, corn or hominy, regu | Lo | Cooked Cereals |
| 56201330 | Grits, cooked, corn or hominy, quic | Lo | Cooked Cereals |
| 56201332 | Grits, cooked, corn or hominy, quic | Lo | Cooked Cereals |
| 56201340 | Grits, instant, made with milk, fat ad | Lo | Cooked Cereals |
| 56201342 | Grits, instant, made with milk, no ad | Lo | Cooked Cereals |
| 56201360 | Grits, instant, made with non-dairy | Lo | Cooked Cereals |
| 56201510 | Cornmeal mush, made with water | Lo | Cooked Cereals |
| 56201515 | Cornmeal mush, NS as to fat | Lo | Cooked Cereals |
| 56201516 | Cornmeal mush, no added fat | Lo | Cooked Cereals |
| 56201517 | Cornmeal mush, fat added | Lo | Cooked Cereals |
| 56201520 | Cornmeal mush, fried | Lo | Cooked Cereals |

| 56201530 | Cornmeal mush, made with milk | Lo | Cooked Cereals |
| --- | --- | --- | --- |
| 56201540 | Cornmeal, Puerto Rican Style | Lo | Cooked Cereals |
| 56201550 | Cornmeal dumpling | Lo | Quick Breads and Bread Products |
| 56201600 | Masa harina, cooked | Lo | Cooked Cereals |
| 56201700 | Cornstarch with milk, eaten as a ce | Lo | Cooked Cereals |
| 56201750 | Cornstarch, dry | Lo | Other |
| 56201800 | Cornstarch, hydrolyzed powder | Lo | Other |
| 56201990 | Millet, NS as to fat | Lo | Cooked Grains |
| 56202000 | Millet, no added fat | Lo | Cooked Grains |
| 56202100 | Millet, fat added | Lo | Cooked Grains |
| 56202900 | Oatmeal, from fast food, plain | Lo | Cooked Cereals |
| 56202905 | Oatmeal, from fast food, maple flav | Lo | Cooked Cereals |
| 56202910 | Oatmeal, from fast food, fruit flavor | Lo | Cooked Cereals |
| 56202920 | Oatmeal, from fast food, other flavo | Lo | Cooked Cereals |
| 56202960 | Oatmeal, NS as to regular, quick, o | Lo | Cooked Cereals |
| 56202970 | Oatmeal, cooked, quick (1 or 3 min | Lo | Cooked Cereals |
| 56202980 | Oatmeal, cooked, regular, NS as to | Lo | Cooked Cereals |
| 56203000 | Oatmeal, NS as to regular, quick, o | Lo | Cooked Cereals |
| 56203010 | Oatmeal, cooked, regular, fat not a | Lo | Cooked Cereals |
| 56203020 | Oatmeal, cooked, quick (1 or 3 min | Lo | Cooked Cereals |
| 56203030 | Oatmeal, cooked, instant, fat not ad | Lo | Cooked Cereals |
| 56203040 | Oatmeal, NS as to regular, quick, o | Lo | Cooked Cereals |
| 56203050 | Oatmeal, cooked, regular, fat adde | Lo | Cooked Cereals |
| 56203055 | Oatmeal, regular or quick, made wi | Lo | Cooked Cereals |
| 56203056 | Oatmeal, regular or quick, made wi | Lo | Cooked Cereals |
| 56203057 | Oatmeal, regular or quick, made wi | Lo | Cooked Cereals |
| 56203060 | Oatmeal, cooked, quick (1 or 3 min | Lo | Cooked Cereals |
| 56203065 | Oatmeal, regular or quick, made wi | Lo | Cooked Cereals |
| 56203066 | Oatmeal, regular or quick, made wi | Lo | Cooked Cereals |
| 56203067 | Oatmeal, regular or quick, made wi | Lo | Cooked Cereals |
| 56203070 | Oatmeal, cooked, instant, fat added | Lo | Cooked Cereals |
| 56203075 | Oatmeal, regular or quick, made wi | Lo | Cooked Cereals |
| 56203076 | Oatmeal, regular or quick, made wi | Lo | Cooked Cereals |
| 56203077 | Oatmeal, regular or quick, made wi | Lo | Cooked Cereals |
| 56203080 | Oatmeal, cooked, instant, NS as to | Lo | Cooked Cereals |
| 56203086 | Oatmeal, instant, plain, made with | Lo | Cooked Cereals |
| 56203087 | Oatmeal, instant, plain, made with | Lo | Cooked Cereals |
| 56203096 | Oatmeal, instant, plain, made with | Lo | Cooked Cereals |
| 56203097 | Oatmeal, instant, plain, made with | Lo | Cooked Cereals |
| 56203106 | Oatmeal, instant, plain, made with | Lo | Cooked Cereals |
| 56203125 | Oatmeal, instant, maple flavored, N | Lo | Cooked Cereals |
| 56203130 | Oatmeal, instant, maple flavored, n | Lo | Cooked Cereals |
| 56203135 | Oatmeal, instant, maple flavored, fa | Lo | Cooked Cereals |
| 56203150 | Oatmeal, instant, fruit flavored, NS | Lo | Cooked Cereals |
| 56203155 | Oatmeal, instant, fruit flavored, no a | Lo | Cooked Cereals |
| 56203160 | Oatmeal, instant, fruit flavored, fat a | Lo | Cooked Cereals |
| 56203175 | Oatmeal, instant, other flavors, no a | Lo | Cooked Cereals |
| 56203180 | Oatmeal, instant, other flavors, fat a | Lo | Cooked Cereals |
| 56203200 | Oatmeal with fruit, cooked | Lo | Cooked Cereals |
| 56203210 | Oatmeal, NS as to regular, quick, o | Lo | Cooked Cereals |
| 56203211 | Oatmeal, cooked, regular, made wi | Lo | Cooked Cereals |

| 56203212 | Oatmeal, cooked, quick (1 or 3 min | Lo | Cooked Cereals |
| --- | --- | --- | --- |
| 56203213 | Oatmeal, cooked, instant, made wit | Lo | Cooked Cereals |
| 56203220 | Oatmeal, NS as to regular, quick, o | Lo | Cooked Cereals |
| 56203221 | Oatmeal, cooked, regular, made wi | Lo | Cooked Cereals |
| 56203222 | Oatmeal, cooked, quick (1 or 3 min | Lo | Cooked Cereals |
| 56203223 | Oatmeal, cooked, instant, made wit | Lo | Cooked Cereals |
| 56203230 | Oatmeal, NS as to regular, quick, o | Lo | Cooked Cereals |
| 56203231 | Oatmeal, cooked, regular, made wi | Lo | Cooked Cereals |
| 56203232 | Oatmeal, cooked, quick (1 or 3 min | Lo | Cooked Cereals |
| 56203233 | Oatmeal, cooked, instant, made wit | Lo | Cooked Cereals |
| 56203510 | Oatmeal, reduced sugar, plain, no | Lo | Cooked Cereals |
| 56203540 | Oatmeal, made with milk and sugar | Lo | Cooked Cereals |
| 56203550 | Oatmeal, reduced sugar, flavored, | Lo | Cooked Cereals |
| 56203555 | Oatmeal, reduced sugar, flavored, | Lo | Cooked Cereals |
| 56203560 | Oatmeal, reduced sugar, flavored, f | Lo | Cooked Cereals |
| 56203600 | Oatmeal, multigrain, NS as to fat | Lo | Cooked Cereals |
| 56203610 | Oatmeal, multigrain, no added fat | Lo | Cooked Cereals |
| 56203620 | Oatmeal, multigrain, fat added | Lo | Cooked Cereals |
| 56204000 | Quinoa, NS as to fat | Lo | Cooked Grains |
| 56204005 | Quinoa, no added fat | Lo | Cooked Grains |
| 56204010 | Quinoa, fat added | Lo | Cooked Grains |
| 56204980 | Rice, white, cooked, converted, NS | Lo | Cooked Grains |
| 56204990 | Rice, white, cooked, regular, NS as | Lo | Cooked Grains |
| 56205000 | Rice, cooked, NFS | Lo | Cooked Grains |
| 56205001 | Rice, white, cooked, NS as to fat | Lo | Cooked Grains |
| 56205002 | Rice, white, cooked, made with oil | Lo | Cooked Grains |
| 56205004 | Rice, white, cooked, made with butt | Lo | Cooked Grains |
| 56205006 | Rice, white, cooked, made with ma | Lo | Cooked Grains |
| 56205007 | Rice, white, cooked, fat added, NS | Lo | Cooked Grains |
| 56205008 | Rice, white, cooked, no added fat | Lo | Cooked Grains |
| 56205010 | Rice, white, cooked, regular, fat not | Lo | Cooked Grains |
| 56205011 | Rice, brown, cooked, NS as to fat | Lo | Cooked Grains |
| 56205012 | Rice, brown, cooked, fat added, ma | Lo | Cooked Grains |
| 56205014 | Rice, brown, cooked, made with bu | Lo | Cooked Grains |
| 56205016 | Rice, brown, cooked, made with ma | Lo | Cooked Grains |
| 56205017 | Rice, brown, cooked, fat added, NS | Lo | Cooked Grains |
| 56205018 | Rice, brown, cooked, no added fat | Lo | Cooked Grains |
| 56205020 | Rice, white, cooked, instant, NS as | Lo | Cooked Grains |
| 56205030 | Rice, white, cooked, instant, fat not | Lo | Cooked Grains |
| 56205040 | Rice, white, cooked, converted, fat | Lo | Cooked Grains |
| 56205050 | Rice, cream of, cooked, no added f | Lo | Cooked Cereals |
| 56205060 | Rice, cooked, with milk | Lo | Cooked Grains |
| 56205070 | Rice, sweet, cooked with honey | Lo | Cooked Grains |
| 56205080 | Rice, creamed, made with milk and | Lo | Cooked Cereals |
| 56205090 | Rice, cream of, cooked, fat added | Lo | Cooked Cereals |
| 56205092 | Rice, cream of, cooked, NS as to fa | Lo | Cooked Cereals |
| 56205094 | Rice, cream of, cooked, made with | Lo | Cooked Cereals |
| 56205101 | Congee | Lo | Cooked Grains |
| 56205110 | Rice, brown, cooked, regular, fat no | Lo | Cooked Grains |
| 56205120 | Rice, brown, cooked, regular, NS a | Lo | Cooked Grains |
| 56205130 | Yellow rice, cooked, NS as to fat | Lo | Cooked Grains |

| 56205150 | Yellow rice, cooked, no added fat | Lo | Cooked Grains |
| --- | --- | --- | --- |
| 56205170 | Yellow rice, cooked, fat added | Lo | Cooked Grains |
| 56205190 | Rice, white, cooked, glutinous | Lo | Cooked Grains |
| 56205200 | Rice, frozen dessert, nondairy, flav | Lo | Other Desserts |
| 56205205 | Rice, wild, 100%, cooked, NS as to | Lo | Cooked Grains |
| 56205210 | Rice, wild, 100%, cooked, no added | Lo | Cooked Grains |
| 56205215 | Rice, wild, 100%, cooked, fat added | Lo | Cooked Grains |
| 56205230 | Rice dessert bar, frozen, flavors oth | Lo | Other Desserts |
| 56205300 | Rice, white and wild, cooked, no ad | Lo | Cooked Grains |
| 56205310 | Rice, brown and wild, cooked, no a | Lo | Cooked Grains |
| 56205320 | Rice, white and wild, cooked, fat ad | Lo | Cooked Grains |
| 56205330 | Rice, white and wild, cooked, NS as | Lo | Cooked Grains |
| 56205340 | Rice, brown and wild, cooked, fat a | Lo | Cooked Grains |
| 56205350 | Rice, brown and wild, cooked, NS a | Lo | Cooked Grains |
| 56205400 | Rice, cooked, NS as to type, fat ad | Lo | Cooked Grains |
| 56205410 | Rice, white, cooked with fat, Puerto | Lo | Cooked Grains |
| 56205420 | Rice, white, cooked, regular, fat ad | Lo | Cooked Grains |
| 56205430 | Rice, white, cooked, instant, fat add | Lo | Cooked Grains |
| 56205440 | Rice, white, cooked, converted, fat | Lo | Cooked Grains |
| 56205510 | Rice, brown, cooked, regular, fat ad | Lo | Cooked Grains |
| 56205530 | Rice, brown, cooked, instant, NS as | Lo | Cooked Grains |
| 56205540 | Rice, brown, cooked, instant, fat no | Lo | Cooked Grains |
| 56205550 | Rice, brown, cooked, instant, fat ad | Lo | Cooked Grains |
| 56206970 | Wheat, cream of, cooked, quick, N | Lo | Cooked Cereals |
| 56206990 | Cream of wheat, NS as to regular, | Lo | Cooked Cereals |
| 56207000 | Cream of wheat, NS as to regular, | Lo | Cooked Cereals |
| 56207005 | Cream of wheat, NS as to regular, | Lo | Cooked Cereals |
| 56207010 | Wheat, cream of, cooked, regular, f | Lo | Cooked Cereals |
| 56207016 | Cream of wheat, regular or quick, m | Lo | Cooked Cereals |
| 56207017 | Cream of wheat, regular or quick, m | Lo | Cooked Cereals |
| 56207020 | Wheat, cream of, cooked, quick, fa | Lo | Cooked Cereals |
| 56207022 | Cream of wheat, regular or quick, m | Lo | Cooked Cereals |
| 56207023 | Cream of wheat, regular or quick, m | Lo | Cooked Cereals |
| 56207027 | Cream of wheat, regular or quick, m | Lo | Cooked Cereals |
| 56207030 | Cream of wheat, instant, made with | Lo | Cooked Cereals |
| 56207040 | Wheat, cream of, cooked, made wi | Lo | Cooked Cereals |
| 56207060 | Cream of wheat, instant, made with | Lo | Cooked Cereals |
| 56207080 | Wheat, cream of, cooked, NS as to | Lo | Cooked Cereals |
| 56207086 | Wheat, cream of, cooked, regular, | Lo | Cooked Cereals |
| 56207087 | Wheat, cream of, cooked, regular, | Lo | Cooked Cereals |
| 56207092 | Wheat, cream of, cooked, quick, m | Lo | Cooked Cereals |
| 56207094 | Cream of wheat, instant, made with | Lo | Cooked Cereals |
| 56207095 | Cream of wheat, instant, made with | Lo | Cooked Cereals |
| 56207102 | Cream of wheat, instant, made with | Lo | Cooked Cereals |
| 56207110 | Bulgur, no added fat | Lo | Cooked Grains |
| 56207120 | Bulgur, fat added | Lo | Cooked Grains |
| 56207130 | Bulgur, NS as to fat | Lo | Cooked Grains |
| 56207150 | Couscous, plain, cooked, fat not ad | Lo | Cooked Grains |
| 56207160 | Couscous, plain, cooked | Lo | Cooked Grains |
| 56207180 | Couscous, plain, cooked, fat added | Lo | Cooked Grains |
| 56207190 | Whole wheat cereal, cooked, NS a | Lo | Cooked Cereals |

| 56207200 | Whole wheat cereal, cooked, no ad | Lo | Cooked Cereals |
| --- | --- | --- | --- |
| 56207210 | Whole wheat cereal, cooked, fat ad | Lo | Cooked Cereals |
| 56207212 | Whole wheat cereal, cooked, made | Lo | Cooked Cereals |
| 56207220 | Wheat, cream of, cooked, regular, f | Lo | Cooked Cereals |
| 56207230 | Wheat, cream of, cooked, quick, fa | Lo | Cooked Cereals |
| 56207300 | Whole wheat cereal, wheat and bar | Lo | Cooked Cereals |
| 56207330 | Whole wheat cereal, wheat and bar | Lo | Cooked Cereals |
| 56207340 | Whole wheat cereal, wheat and bar | Lo | Cooked Cereals |
| 56207342 | Whole wheat cereal, wheat and bar | Lo | Cooked Cereals |
| 56207350 | Wheat cereal, chocolate flavored, c | Lo | Cooked Cereals |
| 56207360 | Wheat cereal, chocolate flavored, c | Lo | Cooked Cereals |
| 56207370 | Wheat cereal, chocolate flavored, c | Lo | Cooked Cereals |
| 56208500 | Oat bran cereal, cooked, no added | Lo | Cooked Cereals |
| 56208510 | Oat bran cereal, cooked, fat added | Lo | Cooked Cereals |
| 56208520 | Oat bran cereal, cooked, NS as to f | Lo | Cooked Cereals |
| 56208530 | Oat bran cereal, cooked, made with | Lo | Cooked Cereals |
| 56209000 | Cream of rye | Lo | Cooked Cereals |
| 56210000 | Cereal, nestum | Lo | Baby Foods |
| 57000000 | Cereal, NFS | Lo | Ready-to-Eat Cereals |
| 57000050 | Kashi cereal, NS as to ready to eat | Lo | Ready-to-Eat Cereals |
| 57000100 | Cereal, oat, NFS | Lo | Ready-to-Eat Cereals |
| 57100100 | Cereal, ready-to-eat, NFS | Lo | Ready-to-Eat Cereals |
| 57100400 | Character cereals, TV or movie, Ge | Lo | Ready-to-Eat Cereals |
| 57100500 | Character cereals, TV or movie, Ke | Lo | Ready-to-Eat Cereals |
| 57101000 | Cereal (Kellogg's All-Bran) | Lo | Ready-to-Eat Cereals |
| 57101020 | All-Bran with Extra Fiber | Lo | Ready-to-Eat Cereals |
| 57102000 | Cereal (Alpen) | Lo | Ready-to-Eat Cereals |
| 57103000 | Cereal (Post Alpha-Bits) | Lo | Ready-to-Eat Cereals |
| 57103020 | Alpha-bits with marshmallows | Lo | Ready-to-Eat Cereals |
| 57103050 | Amaranth Flakes | Lo | Ready-to-Eat Cereals |
| 57103100 | Cereal (General Mills Cheerios App | Lo | Ready-to-Eat Cereals |
| 57103500 | Apple Cinnamon Squares Mini-Wh | Lo | Ready-to-Eat Cereals |
| 57104000 | Cereal (Kellogg's Apple Jacks) | Lo | Ready-to-Eat Cereals |
| 57106050 | Cereal (Post Great Grains Banana | Lo | Ready-to-Eat Cereals |
| 57106060 | Cereal (General Mills Cheerios Ban | Lo | Ready-to-Eat Cereals |
| 57106100 | Cereal (General Mills Basic 4) | Lo | Ready-to-Eat Cereals |
| 57106250 | Cereal (General Mills Kix Berry Ber | Lo | Ready-to-Eat Cereals |
| 57106260 | Cereal (General Mills Cheerios Ber | Lo | Ready-to-Eat Cereals |
| 57106530 | Cereal (Post Selects Blueberry Mor | Lo | Ready-to-Eat Cereals |
| 57107000 | Cereal (General Mills Boo Berry) | Lo | Ready-to-Eat Cereals |
| 57110000 | Cereal (Kellogg's All-Bran Bran Bud | Lo | Ready-to-Eat Cereals |
| 57111000 | Bran Chex | Lo | Ready-to-Eat Cereals |
| 57117000 | Cereal (Quaker Cap'n Crunch) | Lo | Ready-to-Eat Cereals |
| 57117500 | Cereal (Quaker Christmas Crunch) | Lo | Ready-to-Eat Cereals |
| 57119000 | Cereal (Quaker Cap'n Crunch's Cru | Lo | Ready-to-Eat Cereals |
| 57120000 | Cereal (Quaker Cap'n Crunch's Pe | Lo | Ready-to-Eat Cereals |
| 57123000 | Cereal (General Mills Cheerios) | Lo | Ready-to-Eat Cereals |
| 57124000 | Chex cereal, NFS | Lo | Ready-to-Eat Cereals |
| 57124030 | Cereal (General Mills Chex Chocol | Lo | Ready-to-Eat Cereals |
| 57124050 | Cereal (General Mills Chex Cinnam | Lo | Ready-to-Eat Cereals |
| 57124100 | Cereal (General Mills Cheerios Cho | Lo | Ready-to-Eat Cereals |

| 57124200 | Cereal, chocolate flavored, frosted, | Lo | Ready-to-Eat Cereals |
| --- | --- | --- | --- |
| 57124300 | Cereal (General Mills Lucky Charm | Lo | Ready-to-Eat Cereals |
| 57124500 | Cinnamon Grahams, General Mills | Lo | Ready-to-Eat Cereals |
| 57124900 | Cereal (Kellogg's Cinnabon) | Lo | Ready-to-Eat Cereals |
| 57125000 | Cereal (General Mills Cinnamon To | Lo | Ready-to-Eat Cereals |
| 57125010 | Cereal (General Mills 25% Less Su | Lo | Ready-to-Eat Cereals |
| 57125900 | Cereal (General Mills Honey Nut Cl | Lo | Ready-to-Eat Cereals |
| 57126000 | Cereal (Kellogg's Cocoa Krispies) | Lo | Ready-to-Eat Cereals |
| 57126500 | Cocoa Blasts, Quaker | Lo | Ready-to-Eat Cereals |
| 57127000 | Cereal (Post Cocoa Pebbles) | Lo | Ready-to-Eat Cereals |
| 57128000 | Cereal (General Mills Cocoa Puffs) | Lo | Ready-to-Eat Cereals |
| 57128005 | Cereal (General Mills 25% Less Su | Lo | Ready-to-Eat Cereals |
| 57128880 | Complete Oat Bran Flakes, Kellogg | Lo | Ready-to-Eat Cereals |
| 57130000 | Cereal (General Mills Cookie Crisp) | Lo | Ready-to-Eat Cereals |
| 57131000 | Cereal (Quaker Corn Bran Crunch) | Lo | Ready-to-Eat Cereals |
| 57132000 | Cereal (General Mills Chex Corn) | Lo | Ready-to-Eat Cereals |
| 57134000 | Cereal, corn flakes | Lo | Ready-to-Eat Cereals |
| 57134090 | Corn flakes, low sodium | Lo | Ready-to-Eat Cereals |
| 57135000 | Cereal (Kellogg's Corn Flakes) | Lo | Ready-to-Eat Cereals |
| 57137000 | Cereal, corn puffs | Lo | Ready-to-Eat Cereals |
| 57138000 | Total Corn Flakes | Lo | Ready-to-Eat Cereals |
| 57139000 | Cereal (General Mills Count Chocu | Lo | Ready-to-Eat Cereals |
| 57143000 | Cereal (Kellogg's Cracklin' Oat Bra | Lo | Ready-to-Eat Cereals |
| 57143500 | Cereal (Post Great Grains, Cranber | Lo | Ready-to-Eat Cereals |
| 57144000 | Crisp Crunch | Lo | Ready-to-Eat Cereals |
| 57148000 | Cereal (Kellogg's Crispix) | Lo | Ready-to-Eat Cereals |
| 57148500 | Cereal, crispy brown rice | Lo | Ready-to-Eat Cereals |
| 57148600 | Harmony cereal, General Mills | Lo | Ready-to-Eat Cereals |
| 57151000 | Cereal, crispy rice | Lo | Ready-to-Eat Cereals |
| 57152000 | Crispy Wheats'n Raisins | Lo | Ready-to-Eat Cereals |
| 57160000 | Curves Fruit and Nut Crunch Cerea | Lo | Ready-to-Eat Cereals |
| 57201800 | Disney cereals, Kellogg's | Lo | Ready-to-Eat Cereals |
| 57201900 | Cereal (General Mills Dora The Exp | Lo | Ready-to-Eat Cereals |
| 57206000 | Cereal (Famila) | Lo | Ready-to-Eat Cereals |
| 57206700 | Cereal (General Mills Fiber One) | Lo | Ready-to-Eat Cereals |
| 57206705 | Cereal (General Mills Fiber One Ca | Lo | Ready-to-Eat Cereals |
| 57206710 | Cereal (General Mills Fiber One Ho | Lo | Ready-to-Eat Cereals |
| 57206715 | Cereal (General Mills Fiber One Ra | Lo | Ready-to-Eat Cereals |
| 57206800 | Cereal (Healt Valley Fiber 7 Flakes | Lo | Ready-to-Eat Cereals |
| 57207000 | Cereal, bran flakes | Lo | Ready-to-Eat Cereals |
| 57208000 | Cereal (Kellogg's All-Bran Complet | Lo | Ready-to-Eat Cereals |
| 57209000 | Cereal (Post Bran Flakes) | Lo | Ready-to-Eat Cereals |
| 57211000 | Cereal (General Mills Frankenberry | Lo | Ready-to-Eat Cereals |
| 57212100 | French Toast Crunch, General Mills | Lo | Ready-to-Eat Cereals |
| 57213000 | Cereal (Kellogg's Froot Loops) | Lo | Ready-to-Eat Cereals |
| 57213005 | Froot Loops Cereal Straws | Lo | Ready-to-Eat Cereals |
| 57213010 | Cereal (Kellogg's Froot Loops Mars | Lo | Ready-to-Eat Cereals |
| 57213850 | Cereal (General Mills Cheerios Fro | Lo | Ready-to-Eat Cereals |
| 57214000 | Cereal (Kellogg's Frosted Mini-Whe | Lo | Ready-to-Eat Cereals |
| 57214100 | Frosted Wheat Bites | Lo | Ready-to-Eat Cereals |
| 57215000 | Frosty O's | Lo | Ready-to-Eat Cereals |

| 57216000 | Cereal, frosted rice | Lo | Ready-to-Eat Cereals |
| --- | --- | --- | --- |
| 57218000 | Cereal (Kellogg's Frosted Krispies) | Lo | Ready-to-Eat Cereals |
| 57219000 | Cereal, fruit and fiber | Lo | Ready-to-Eat Cereals |
| 57221000 | Cereal, fiber and fruit | Lo | Ready-to-Eat Cereals |
| 57221650 | Fruit Harvest cereal, Kellogg's | Lo | Ready-to-Eat Cereals |
| 57221700 | Cereal, fruit rings | Lo | Ready-to-Eat Cereals |
| 57221800 | Cereal, fruit whirls | Lo | Ready-to-Eat Cereals |
| 57221810 | Cereal (General Mills Cheerios Frui | Lo | Ready-to-Eat Cereals |
| 57223000 | Cereal (Post Fruity Pebbles) | Lo | Ready-to-Eat Cereals |
| 57224000 | Cereal (General Mills Golden Grah | Lo | Ready-to-Eat Cereals |
| 57227000 | Cereal, granola | Lo | Ready-to-Eat Cereals |
| 57228000 | Granola, homemade | Lo | Ready-to-Eat Cereals |
| 57229000 | Cereal (Kellogg's Low Fat Granola) | Lo | Ready-to-Eat Cereals |
| 57229500 | Cereal (Kellogg's Low Fat Granola | Lo | Ready-to-Eat Cereals |
| 57230000 | Cereal (Post Grape-Nuts) | Lo | Ready-to-Eat Cereals |
| 57231000 | Cereal (Post Grape-Nuts Flakes) | Lo | Ready-to-Eat Cereals |
| 57231200 | Cereal (Post Great Grains Raisins, | Lo | Ready-to-Eat Cereals |
| 57231250 | Cereal (Post Great Grains Double | Lo | Ready-to-Eat Cereals |
| 57232100 | Healthy Choice Almond Crunch wit | Lo | Ready-to-Eat Cereals |
| 57237100 | Cereal (Post Honey Bunches of Oa | Lo | Ready-to-Eat Cereals |
| 57237200 | Cereal (Post Honey Bunches of Oa | Lo | Ready-to-Eat Cereals |
| 57237300 | Cereal (Post Honey Bunches of Oa | Lo | Ready-to-Eat Cereals |
| 57237310 | Cereal (Post Honey Bunches of Oa | Lo | Ready-to-Eat Cereals |
| 57237900 | Cereal (Post Honey Bunches of Oa | Lo | Ready-to-Eat Cereals |
| 57238000 | Cereal (Post Honeycomb) | Lo | Ready-to-Eat Cereals |
| 57239000 | Honeycomb, strawberry | Lo | Ready-to-Eat Cereals |
| 57239100 | Cereal (Kellogg's Honey Crunch Co | Lo | Ready-to-Eat Cereals |
| 57240100 | Cereal (General Mills Chex Honey | Lo | Ready-to-Eat Cereals |
| 57241000 | Cereal (General Mills Cheerios Hon | Lo | Ready-to-Eat Cereals |
| 57241200 | Cereal (Post Shredded Wheat Hon | Lo | Ready-to-Eat Cereals |
| 57243000 | Cereal (Kellogg's Honey Smacks) | Lo | Ready-to-Eat Cereals |
| 57245000 | Just Right Fruit and Nut (formerly J | Lo | Ready-to-Eat Cereals |
| 57301100 | Kaboom | Lo | Ready-to-Eat Cereals |
| 57301500 | Cereal (Kashi 7 Whole Grain Puffs) | Lo | Ready-to-Eat Cereals |
| 57301505 | Cereal (Kashi Autumn Wheat) | Lo | Ready-to-Eat Cereals |
| 57301510 | Cereal (Kashi GOLEAN) | Lo | Ready-to-Eat Cereals |
| 57301511 | Cereal (Kashi GOLEAN Crunch) | Lo | Ready-to-Eat Cereals |
| 57301512 | Cereal (Kashi GOLEAN Crunch Ho | Lo | Ready-to-Eat Cereals |
| 57301520 | Cereal (Kashi Good Friends) | Lo | Ready-to-Eat Cereals |
| 57301530 | Cereal (Kashi Heart to Heart Honey | Lo | Ready-to-Eat Cereals |
| 57301535 | Cereal (Kashi Heart to Heart Oat Fl | Lo | Ready-to-Eat Cereals |
| 57301540 | Cereal (Kashi Honey Sunshine Squ | Lo | Ready-to-Eat Cereals |
| 57302100 | Cereal (Quaker King Vitaman) | Lo | Ready-to-Eat Cereals |
| 57303100 | Cereal (General Mills Kix) | Lo | Ready-to-Eat Cereals |
| 57303105 | Cereal (General Mills Honey Kix) | Lo | Ready-to-Eat Cereals |
| 57303200 | Cereal (Kellogg's Krave) | Lo | Ready-to-Eat Cereals |
| 57304100 | Cereal (Quaker Life) | Lo | Ready-to-Eat Cereals |
| 57305100 | Cereal (General Mills Lucky Charm | Lo | Ready-to-Eat Cereals |
| 57305150 | Cereal, frosted oat cereal with mars | Lo | Ready-to-Eat Cereals |
| 57305160 | Cereal (Malt-O-Meal Blueberry Muf | Lo | Ready-to-Eat Cereals |
| 57305165 | Cereal (Malt-O-Meal Cinnamon To | Lo | Ready-to-Eat Cereals |

| 57305170 | Cereal (Malt-O-Meal Coco-Roos) | Lo | Ready-to-Eat Cereals |
| --- | --- | --- | --- |
| 57305174 | Cereal (Malt-O-Meal Colossal Crun | Lo | Ready-to-Eat Cereals |
| 57305175 | Cereal (Malt-O-Meal Cocoa Dyno-B | Lo | Ready-to-Eat Cereals |
| 57305180 | Cereal (Malt-O-Meal Corn Bursts) | Lo | Ready-to-Eat Cereals |
| 57305200 | Cereal (Malt-O-Meal Crispy Rice) | Lo | Ready-to-Eat Cereals |
| 57305210 | Cereal (Malt-O-Meal Frosted Flake | Lo | Ready-to-Eat Cereals |
| 57305215 | Cereal (Malt-O-Meal Frosted Mini S | Lo | Ready-to-Eat Cereals |
| 57305300 | Cereal (Malt-O-Meal Fruity Dyno-Bi | Lo | Ready-to-Eat Cereals |
| 57305400 | Cereal (Malt-O-Meal Honey Graha | Lo | Ready-to-Eat Cereals |
| 57305500 | Cereal (Malt-O-Meal Honey Nut To | Lo | Ready-to-Eat Cereals |
| 57305600 | Cereal (Malt-O-Meal Marshmallow | Lo | Ready-to-Eat Cereals |
| 57306100 | Malt-O-Meal Puffed Rice | Lo | Ready-to-Eat Cereals |
| 57306120 | Malt-O-Meal Puffed Wheat | Lo | Ready-to-Eat Cereals |
| 57306130 | Cereal (Malt-O-Meal Raisin Bran) | Lo | Ready-to-Eat Cereals |
| 57306500 | Cereal (Malt-O-Meal Golden Puffs) | Lo | Ready-to-Eat Cereals |
| 57306700 | Cereal (Malt-O-Meal Toasted Oat C | Lo | Ready-to-Eat Cereals |
| 57306800 | Cereal (Malt-O-Meal Tootie Fruities | Lo | Ready-to-Eat Cereals |
| 57307010 | Cereal (Post Maple Pecan Crunch) | Lo | Ready-to-Eat Cereals |
| 57307150 | Marshmallow Safari, Quaker | Lo | Ready-to-Eat Cereals |
| 57307500 | Cereal, millet, puffed | Lo | Ready-to-Eat Cereals |
| 57307600 | Mini-Swirlz Cinnamon Bun Cereal, | Lo | Ready-to-Eat Cereals |
| 57308150 | Mueslix cereal, NFS | Lo | Ready-to-Eat Cereals |
| 57308190 | Cereal, muesli | Lo | Ready-to-Eat Cereals |
| 57308300 | Multi Bran Chex | Lo | Ready-to-Eat Cereals |
| 57308400 | Cereal (General Mills Cheerios Mul | Lo | Ready-to-Eat Cereals |
| 57309100 | Cereal (Nature Valley Granola) | Lo | Ready-to-Eat Cereals |
| 57316200 | Cereal, nutty nuggets | Lo | Ready-to-Eat Cereals |
| 57316300 | Cereal (Health Valley Oat Bran Flak | Lo | Ready-to-Eat Cereals |
| 57316380 | Cereal (General Mills Cheerios Oat | Lo | Ready-to-Eat Cereals |
| 57316385 | Cereal (General Mills Cheerios Pro | Lo | Ready-to-Eat Cereals |
| 57316410 | Oatmeal Crisp, Apple Cinnamon (fo | Lo | Ready-to-Eat Cereals |
| 57316450 | Cereal (General Mills Oatmeal Cris | Lo | Ready-to-Eat Cereals |
| 57316500 | Cereal (General Mills Oatmeal Cris | Lo | Ready-to-Eat Cereals |
| 57316710 | Cereal (Quaker Honey Graham Oh | Lo | Ready-to-Eat Cereals |
| 57316750 | Oh's, Fruitangy, Quaker | Lo | Ready-to-Eat Cereals |
| 57318000 | 100% Bran | Lo | Ready-to-Eat Cereals |
| 57319000 | 100% Natural Cereal, plain, Quake | Lo | Ready-to-Eat Cereals |
| 57319500 | Sun Country 100% Natural Granola | Lo | Ready-to-Eat Cereals |
| 57320500 | Cereal (Quaker Granola with Oats, | Lo | Ready-to-Eat Cereals |
| 57321500 | 100 % Natural Wholegrain Cereal w | Lo | Ready-to-Eat Cereals |
| 57321700 | Optimum, Nature's Path | Lo | Ready-to-Eat Cereals |
| 57321800 | Optimum Slim, Nature's Path | Lo | Ready-to-Eat Cereals |
| 57321900 | Cereal (Nature's Path Organic Flax | Lo | Ready-to-Eat Cereals |
| 57321905 | Organic Flax Plus, Pumpkin Granol | Lo | Ready-to-Eat Cereals |
| 57322500 | Oreo O's cereal, Post | Lo | Ready-to-Eat Cereals |
| 57323000 | Cereal (Quaker Sweet Crunch) | Lo | Ready-to-Eat Cereals |
| 57323050 | Sweet Puffs, Quaker | Lo | Ready-to-Eat Cereals |
| 57324000 | Peanut Butter Toast Crunch, Gener | Lo | Ready-to-Eat Cereals |
| 57325000 | Cereal (Kellogg's Product 19) | Lo | Ready-to-Eat Cereals |
| 57326000 | Cereal (Barbara's Puffins) | Lo | Ready-to-Eat Cereals |
| 57327450 | Cereal (Quaker Toasted Oat Bran) | Lo | Ready-to-Eat Cereals |

| 57327500 | Cereal (Quaker Oatmeal Squares) | Lo | Ready-to-Eat Cereals |
| --- | --- | --- | --- |
| 57328000 | Cereal (Quaker Quisp) | Lo | Ready-to-Eat Cereals |
| 57329000 | Cereal, raisin bran | Lo | Ready-to-Eat Cereals |
| 57330000 | Cereal (Kellogg's Raisin Bran) | Lo | Ready-to-Eat Cereals |
| 57330010 | Cereal (Kellogg's Raisin Bran Crun | Lo | Ready-to-Eat Cereals |
| 57331000 | Cereal (Post Raisin Bran) | Lo | Ready-to-Eat Cereals |
| 57332050 | Cereal (General Mills Total Raisin B | Lo | Ready-to-Eat Cereals |
| 57332100 | Cereal (General Mills Raisin Nut Br | Lo | Ready-to-Eat Cereals |
| 57335550 | Cereal (General Mills Reese's Puffs | Lo | Ready-to-Eat Cereals |
| 57336000 | Cereal (General Mills Chex Rice) | Lo | Ready-to-Eat Cereals |
| 57337000 | Cereal, rice flakes | Lo | Ready-to-Eat Cereals |
| 57339000 | Cereal (Kellogg's Rice Krispies) | Lo | Ready-to-Eat Cereals |
| 57339100 | Rice Krispies with Real Strawberrie | Lo | Ready-to-Eat Cereals |
| 57339500 | Cereal (Kellogg's Rice Krispies Tre | Lo | Ready-to-Eat Cereals |
| 57340000 | Cereal, puffed rice | Lo | Ready-to-Eat Cereals |
| 57340700 | Scooby Doo cereal, Kellogg's | Lo | Ready-to-Eat Cereals |
| 57341000 | Cereal (Post Shredded Wheat'n Br | Lo | Ready-to-Eat Cereals |
| 57341200 | Cereal (Kellogg's Smart Start Stron | Lo | Ready-to-Eat Cereals |
| 57341300 | Cereal (Kellogg's Smorz) | Lo | Ready-to-Eat Cereals |
| 57342010 | Smorz, Kellogg's | Lo | Ready-to-Eat Cereals |
| 57344000 | Cereal (Kellogg's Special K) | Lo | Ready-to-Eat Cereals |
| 57344001 | Cereal (Kellogg's Special K Bluebe | Lo | Ready-to-Eat Cereals |
| 57344005 | Cereal (Kellogg's Special K Chocol | Lo | Ready-to-Eat Cereals |
| 57344007 | Cereal (Kellogg's Special K Low Fa | Lo | Ready-to-Eat Cereals |
| 57344010 | Cereal (Kellogg's Special K Red Be | Lo | Ready-to-Eat Cereals |
| 57344015 | Cereal (Kellogg's Special K Fruit & | Lo | Ready-to-Eat Cereals |
| 57344020 | Cereal (Kellogg's Special K Vanilla | Lo | Ready-to-Eat Cereals |
| 57344025 | Cereal (Kellogg's Special K Cinnam | Lo | Ready-to-Eat Cereals |
| 57346500 | Oatmeal Honey Nut Heaven, Quak | Lo | Ready-to-Eat Cereals |
| 57347000 | Cereal (Kellogg's Corn Pops) | Lo | Ready-to-Eat Cereals |
| 57347500 | Strawberry Squares Mini-Wheats, K | Lo | Ready-to-Eat Cereals |
| 57348000 | Cereal, frosted corn flakes | Lo | Ready-to-Eat Cereals |
| 57349000 | Cereal (Kellogg's Frosted Flakes) | Lo | Ready-to-Eat Cereals |
| 57349020 | Cereal (Kellogg's Frosted Flakes, R | Lo | Ready-to-Eat Cereals |
| 57355000 | Cereal (Post Golden Crisp) | Lo | Ready-to-Eat Cereals |
| 57401100 | Cereal, toasted oat | Lo | Ready-to-Eat Cereals |
| 57403100 | Toasties, Post | Lo | Ready-to-Eat Cereals |
| 57404100 | Malt-O-Meal Toasty O's | Lo | Ready-to-Eat Cereals |
| 57404200 | Malt-O-Meal Apple and Cinnamon | Lo | Ready-to-Eat Cereals |
| 57406100 | Cereal (General Mills Total) | Lo | Ready-to-Eat Cereals |
| 57406105 | Total Cranberry Crunch | Lo | Ready-to-Eat Cereals |
| 57407100 | Cereal (General Mills Trix) | Lo | Ready-to-Eat Cereals |
| 57407110 | Cereal (General Mills 25% Less Su | Lo | Ready-to-Eat Cereals |
| 57408100 | Cereal (Uncle Sam) | Lo | Ready-to-Eat Cereals |
| 57409100 | Cereal (Post Waffle Crisp) | Lo | Ready-to-Eat Cereals |
| 57410000 | Cereal (Weetabix Whole Grain) | Lo | Ready-to-Eat Cereals |
| 57411000 | Cereal (General Mills Chex Wheat) | Lo | Ready-to-Eat Cereals |
| 57412000 | Wheat germ, plain | Lo | Other |
| 57416000 | Cereal, puffed wheat, plain | Lo | Ready-to-Eat Cereals |
| 57416010 | Cereal, puffed wheat, sweetened | Lo | Ready-to-Eat Cereals |
| 57417000 | Cereal (Post Shredded Wheat) | Lo | Ready-to-Eat Cereals |

| 57418000 | Cereal (General Mills Wheaties) | Lo | Ready-to-Eat Cereals |
| --- | --- | --- | --- |
| 57419000 | Cereal (General Mills Cheerios Yog | Lo | Ready-to-Eat Cereals |
| 57601100 | Wheat bran, unprocessed | Lo | Other |
| 57603100 | Rice polishings | Lo | Other |
| 57604100 | Whole wheat, cracked | Lo | Other |
| 57801000 | Barley cereal, baby food, dry, instan | Lo | Baby Foods |
| 57803000 | Mixed cereal, baby food, dry, instan | Lo | Baby Foods |
| 57804000 | Oatmeal cereal, baby food, dry, inst | Lo | Baby Foods |
| 57805000 | Rice cereal, baby food, dry, instant | Lo | Baby Foods |
| 57805080 | Rice cereal with apples, baby food, | Lo | Baby Foods |
| 57805090 | Rice cereal with mixed fruits, baby f | Lo | Baby Foods |
| 57805100 | Rice cereal with bananas, baby foo | Lo | Baby Foods |
| 57805500 | Brown rice cereal, baby food, dry, i | Lo | Baby Foods |
| 57806000 | Mixed cereal with bananas, baby fo | Lo | Baby Foods |
| 57806050 | Multigrain, whole grain cereal, baby | Lo | Baby Foods |
| 57806100 | Oatmeal cereal with bananas, baby | Lo | Baby Foods |
| 57806200 | Oatmeal cereal with fruit, baby food | Lo | Baby Foods |
| 57807010 | Whole wheat cereal with apples, ba | Lo | Baby Foods |
| 57820000 | Cereal, baby food, jarred, NFS | Lo | Baby Foods |
| 57820100 | Rice cereal, baby food, jarred, NFS | Lo | Baby Foods |
| 57822000 | Mixed cereal with applesauce and b | Lo | Baby Foods |
| 57823000 | Oatmeal with applesauce and bana | Lo | Baby Foods |
| 57824000 | Rice cereal with applesauce and ba | Lo | Baby Foods |
| 57824500 | Rice cereal with mixed fruit, baby fo | Lo | Baby Foods |
| 57830100 | Gerber Graduates Finger Snacks C | Lo | Baby Foods |
| 58100000 | Burrito, taco, or quesadilla with egg | Lo | Mixed Dishes - Sandwiches (single code) |
| 58100005 | Burrito, taco, or quesadilla with egg | Lo | Mixed Dishes - Sandwiches (single code) |
| 58100010 | Burrito, taco, or quesadilla with egg | Lo | Mixed Dishes - Sandwiches (single code) |
| 58100013 | Burrito, taco, or quesadilla with egg | Lo | Mixed Dishes - Sandwiches (single code) |
| 58100015 | Burrito, taco, or quesadilla with egg | Lo | Mixed Dishes - Sandwiches (single code) |
| 58100017 | Burrito, taco, or quesadilla with egg | Lo | Mixed Dishes - Sandwiches (single code) |
| 58100020 | Burrito, taco, or quesadilla with egg | Lo | Mixed Dishes - Sandwiches (single code) |
| 58100100 | Burrito with meat | Lo | Mixed Dishes - Mexican |
| 58100110 | Burrito with beef and beans | Lo | Mixed Dishes - Mexican |
| 58100120 | Burrito with meat and beans | Lo | Mixed Dishes - Mexican |
| 58100125 | Burrito with meat and beans, from f | Lo | Mixed Dishes - Mexican |
| 58100130 | Burrito with beef and cheese, no be | Lo | Mixed Dishes - Mexican |
| 58100135 | Burrito with meat and sour cream | Lo | Mixed Dishes - Mexican |
| 58100140 | Burrito with meat, beans, and sour | Lo | Mixed Dishes - Mexican |
| 58100145 | Burrito with meat, beans, and sour | Lo | Mixed Dishes - Mexican |
| 58100150 | Burrito with beef and potato, no bea | Lo | Mixed Dishes - Mexican |
| 58100155 | Burrito with beef, rice, and cheese | Lo | Mixed Dishes - Mexican |
| 58100160 | Burrito with meat, beans, and rice | Lo | Mixed Dishes - Mexican |
| 58100165 | Burrito with meat, beans, rice, and | Lo | Mixed Dishes - Mexican |
| 58100180 | Burrito with pork and beans | Lo | Mixed Dishes - Mexican |
| 58100200 | Burrito with chicken | Lo | Mixed Dishes - Mexican |
| 58100210 | Burrito with chicken and beans | Lo | Mixed Dishes - Mexican |
| 58100220 | Burrito with chicken and beans | Lo | Mixed Dishes - Mexican |
| 58100230 | Burrito with chicken and cheese | Lo | Mixed Dishes - Mexican |
| 58100235 | Burrito with chicken and sour cream | Lo | Mixed Dishes - Mexican |
| 58100240 | Burrito with chicken, NFS | Lo | Mixed Dishes - Mexican |

| 58100245 | Burrito with chicken, beans, and so | Lo | Mixed Dishes - Mexican |
| --- | --- | --- | --- |
| 58100250 | Burrito with chicken, rice, and chee | Lo | Mixed Dishes - Mexican |
| 58100255 | Burrito with chicken, beans, and ric | Lo | Mixed Dishes - Mexican |
| 58100260 | Burrito with chicken, beans, rice, an | Lo | Mixed Dishes - Mexican |
| 58100300 | Burrito with beans and rice, meatle | Lo | Mixed Dishes - Mexican |
| 58100310 | Burrito with beans, meatless | Lo | Mixed Dishes - Mexican |
| 58100320 | Burrito with beans, meatless | Lo | Mixed Dishes - Mexican |
| 58100325 | Burrito with beans, meatless, from f | Lo | Mixed Dishes - Mexican |
| 58100330 | Burrito with beans, rice, and sour cr | Lo | Mixed Dishes - Mexican |
| 58100340 | Burrito with eggs, sausage, cheese | Lo | Mixed Dishes - Sandwiches (single code) |
| 58100350 | Burrito with eggs and cheese, no b | Lo | Mixed Dishes - Sandwiches (single code) |
| 58100360 | Chilaquiles, tortilla casserole with s | Lo | Mixed Dishes - Mexican |
| 58100370 | Chilaquiles, tortilla casserole with s | Lo | Mixed Dishes - Mexican |
| 58100400 | Enchilada with beef, no beans | Lo | Mixed Dishes - Mexican |
| 58100410 | Burrito with beef, cheese, and sour | Lo | Mixed Dishes - Mexican |
| 58100500 | Enchilada, no sauce | Lo | Mixed Dishes - Mexican |
| 58100510 | Enchilada with beef and beans | Lo | Mixed Dishes - Mexican |
| 58100520 | Enchilada with meat and beans, red | Lo | Mixed Dishes - Mexican |
| 58100525 | Enchilada with meat and beans, gre | Lo | Mixed Dishes - Mexican |
| 58100530 | Enchilada with meat, red-chile or e | Lo | Mixed Dishes - Mexican |
| 58100535 | Enchilada with meat, green-chile or | Lo | Mixed Dishes - Mexican |
| 58100560 | Enchilada with ham and cheese, no | Lo | Mixed Dishes - Mexican |
| 58100600 | Enchilada with chicken, tomato-bas | Lo | Mixed Dishes - Mexican |
| 58100610 | Enchilada with chicken and beans, | Lo | Mixed Dishes - Mexican |
| 58100620 | Enchilada with chicken and beans, | Lo | Mixed Dishes - Mexican |
| 58100625 | Enchilada with chicken and beans, | Lo | Mixed Dishes - Mexican |
| 58100630 | Enchilada with chicken, red-chile or | Lo | Mixed Dishes - Mexican |
| 58100635 | Enchilada with chicken, green-chile | Lo | Mixed Dishes - Mexican |
| 58100710 | Enchilada with beans, meatless | Lo | Mixed Dishes - Mexican |
| 58100720 | Enchilada with beans, meatless, re | Lo | Mixed Dishes - Mexican |
| 58100725 | Enchilada with beans, green-chile o | Lo | Mixed Dishes - Mexican |
| 58100800 | Enchilada, just cheese, meatless, n | Lo | Mixed Dishes - Mexican |
| 58100805 | Enchilada, just cheese, meatless, n | Lo | Mixed Dishes - Mexican |
| 58100900 | Enchilada with seafood, tomato-bas | Lo | Mixed Dishes - Mexican |
| 58101200 | Flauta, NFS | Lo | Mixed Dishes - Mexican |
| 58101230 | Flauta with beef | Lo | Mixed Dishes - Mexican |
| 58101240 | Flauta with chicken | Lo | Mixed Dishes - Mexican |
| 58101300 | Taco or tostada with beef, cheese a | Lo | Mixed Dishes - Mexican |
| 58101310 | Taco or tostada with beef, lettuce, t | Lo | Mixed Dishes - Mexican |
| 58101320 | Taco or tostada with meat | Lo | Mixed Dishes - Mexican |
| 58101323 | Taco or tostada with meat, from fas | Lo | Mixed Dishes - Mexican |
| 58101325 | Taco or tostada with meat and sour | Lo | Mixed Dishes - Mexican |
| 58101345 | Soft taco with meat | Lo | Mixed Dishes - Mexican |
| 58101347 | Soft taco with meat, from fast food | Lo | Mixed Dishes - Mexican |
| 58101350 | Soft taco with meat and sour cream | Lo | Mixed Dishes - Mexican |
| 58101357 | Soft taco with meat and sour cream | Lo | Mixed Dishes - Mexican |
| 58101400 | Soft taco with beef, cheese, and let | Lo | Mixed Dishes - Mexican |
| 58101450 | Soft taco with chicken | Lo | Mixed Dishes - Mexican |
| 58101457 | Soft taco with chicken, from fast foo | Lo | Mixed Dishes - Mexican |
| 58101460 | Soft taco with chicken and sour cre | Lo | Mixed Dishes - Mexican |
| 58101510 | Taco or tostada with chicken or turk | Lo | Mixed Dishes - Mexican |

| 58101520 | Taco or tostada with chicken | Lo | Mixed Dishes - Mexican |
| --- | --- | --- | --- |
| 58101525 | Taco or tostada with chicken and s | Lo | Mixed Dishes - Mexican |
| 58101530 | Soft taco with beef, cheese, lettuce | Lo | Mixed Dishes - Mexican |
| 58101540 | Taco or tostada with fish | Lo | Mixed Dishes - Mexican |
| 58101555 | Soft taco with fish | Lo | Mixed Dishes - Mexican |
| 58101600 | Soft taco with bean, cheese, and le | Lo | Mixed Dishes - Mexican |
| 58101610 | Soft taco with beans | Lo | Mixed Dishes - Mexican |
| 58101615 | Soft taco with beans and sour crea | Lo | Mixed Dishes - Mexican |
| 58101620 | Soft taco with meat and beans | Lo | Mixed Dishes - Mexican |
| 58101625 | Soft taco with chicken and beans | Lo | Mixed Dishes - Mexican |
| 58101630 | Soft taco with meat, beans, and so | Lo | Mixed Dishes - Mexican |
| 58101635 | Soft taco with chicken, beans, and | Lo | Mixed Dishes - Mexican |
| 58101710 | Taco or tostada with beans, meatle | Lo | Mixed Dishes - Mexican |
| 58101720 | Taco or tostada with beans | Lo | Mixed Dishes - Mexican |
| 58101725 | Taco or tostada with beans and sou | Lo | Mixed Dishes - Mexican |
| 58101730 | Taco or tostada with meat and bea | Lo | Mixed Dishes - Mexican |
| 58101733 | Taco or tostada with meat and bea | Lo | Mixed Dishes - Mexican |
| 58101735 | Taco or tostada with chicken and b | Lo | Mixed Dishes - Mexican |
| 58101740 | Soft taco with egg and potato | Lo | Mixed Dishes - Sandwiches (single code) |
| 58101745 | Taco or tostada with meat, beans, | Lo | Mixed Dishes - Mexican |
| 58101750 | Taco or tostada with chicken, bean | Lo | Mixed Dishes - Mexican |
| 58101800 | Ground beef with tomato sauce and | Lo | Mixed Dishes - Mexican |
| 58101820 | Mexican casserole made with grou | Lo | Mixed Dishes - Mexican |
| 58101830 | Mexican casserole made with grou | Lo | Mixed Dishes - Mexican |
| 58101910 | Taco or tostada salad with beef and | Lo | Mixed Dishes - Mexican |
| 58101930 | Taco or tostada salad with meat | Lo | Mixed Dishes - Mexican |
| 58101935 | Taco or tostada salad with chicken | Lo | Mixed Dishes - Mexican |
| 58101940 | Taco or tostada salad, meatless | Lo | Mixed Dishes - Mexican |
| 58101945 | Taco or tostada salad with meat an | Lo | Mixed Dishes - Mexican |
| 58101950 | Taco or tostada salad with chicken | Lo | Mixed Dishes - Mexican |
| 58101955 | Taco or tostada salad, meatless wit | Lo | Mixed Dishes - Mexican |
| 58103110 | Tamale with meat and/or poultry | Lo | Mixed Dishes - Mexican |
| 58103120 | Tamale with meat | Lo | Mixed Dishes - Mexican |
| 58103130 | Tamale with chicken | Lo | Mixed Dishes - Mexican |
| 58103200 | Tamale, plain, meatless, no sauce, | Lo | Mixed Dishes - Mexican |
| 58103210 | Tamale, meatless, with sauce, Pue | Lo | Mixed Dishes - Mexican |
| 58103250 | Tamale, plain, meatless, no sauce, | Lo | Mixed Dishes - Mexican |
| 58103310 | Tamale casserole with meat | Lo | Mixed Dishes - Mexican |
| 58104080 | Nachos with beef, beans, cheese, a | Lo | Mixed Dishes - Mexican |
| 58104090 | Nachos with cheese and sour crea | Lo | Mixed Dishes - Mexican |
| 58104100 | Nachos with cheese, meatless, no | Lo | Mixed Dishes - Mexican |
| 58104110 | Nachos with beans, no cheese | Lo | Mixed Dishes - Mexican |
| 58104120 | Nachos with cheese | Lo | Mixed Dishes - Mexican |
| 58104130 | Nachos with meat and cheese | Lo | Mixed Dishes - Mexican |
| 58104140 | Nachos with beef and cheese | Lo | Mixed Dishes - Mexican |
| 58104150 | Nachos with chicken and cheese | Lo | Mixed Dishes - Mexican |
| 58104160 | Nachos with chili | Lo | Mixed Dishes - Mexican |
| 58104180 | Nachos with meat, cheese, and sou | Lo | Mixed Dishes - Mexican |
| 58104190 | Nachos with chicken, cheese, and | Lo | Mixed Dishes - Mexican |
| 58104250 | Nachos with chicken or turkey and | Lo | Mixed Dishes - Mexican |
| 58104260 | Gordita, sope, or chalupa with bean | Lo | Mixed Dishes - Mexican |

| 58104270 | Gordita, sope, or chalupa with bean | Lo | Mixed Dishes - Mexican |
| --- | --- | --- | --- |
| 58104280 | Gordita, sope, or chalupa with mea | Lo | Mixed Dishes - Mexican |
| 58104290 | Gordita, sope, or chalupa with mea | Lo | Mixed Dishes - Mexican |
| 58104310 | Chalupa with beans, chicken, chee | Lo | Mixed Dishes - Mexican |
| 58104320 | Gordita, sope, or chalupa with chick | Lo | Mixed Dishes - Mexican |
| 58104340 | Gordita, sope, or chalupa with chick | Lo | Mixed Dishes - Mexican |
| 58104450 | Chimichanga with beef and tomato | Lo | Mixed Dishes - Mexican |
| 58104500 | Chimichanga with meat | Lo | Mixed Dishes - Mexican |
| 58104510 | Chimichanga with beef, cheese, let | Lo | Mixed Dishes - Mexican |
| 58104520 | Chimichanga, meatless | Lo | Mixed Dishes - Mexican |
| 58104530 | Chimichanga with chicken | Lo | Mixed Dishes - Mexican |
| 58104535 | Chimichanga with meat and sour cr | Lo | Mixed Dishes - Mexican |
| 58104550 | Chimichanga with chicken and sour | Lo | Mixed Dishes - Mexican |
| 58104600 | Chimichanga with beef and rice | Lo | Mixed Dishes - Mexican |
| 58104710 | Quesadilla, just cheese, meatless | Lo | Mixed Dishes - Mexican |
| 58104720 | Quesadilla, just cheese, from fast f | Lo | Mixed Dishes - Mexican |
| 58104730 | Quesadilla with meat | Lo | Mixed Dishes - Mexican |
| 58104740 | Quesadilla with chicken | Lo | Mixed Dishes - Mexican |
| 58104745 | Quesadilla with chicken, from fast f | Lo | Mixed Dishes - Mexican |
| 58104750 | Quesadilla with vegetables | Lo | Mixed Dishes - Mexican |
| 58104760 | Quesadilla with vegetables and me | Lo | Mixed Dishes - Mexican |
| 58104770 | Quesadilla with vegetables and chic | Lo | Mixed Dishes - Mexican |
| 58104800 | Taquito or flauta with cheese | Lo | Mixed Dishes - Mexican |
| 58104810 | Taquitoes | Lo | Mixed Dishes - Mexican |
| 58104820 | Taquito or flauta with meat | Lo | Mixed Dishes - Mexican |
| 58104825 | Taquito or flauta with meat and che | Lo | Mixed Dishes - Mexican |
| 58104830 | Taquito or flauta with chicken | Lo | Mixed Dishes - Mexican |
| 58104835 | Taquito or flauta with chicken and c | Lo | Mixed Dishes - Mexican |
| 58104900 | Taquito or flauta with egg | Lo | Mixed Dishes - Sandwiches (single code) |
| 58104905 | Taquito or flauta with egg and brea | Lo | Mixed Dishes - Sandwiches (single code) |
| 58105000 | Fajita with chicken and vegetables | Lo | Mixed Dishes - Mexican |
| 58105050 | Fajita with meat and vegetables | Lo | Mixed Dishes - Mexican |
| 58105075 | Fajita with vegetables | Lo | Mixed Dishes - Mexican |
| 58105100 | Pupusa, cheese-filled | Lo | Mixed Dishes - Mexican |
| 58105105 | Pupusa, bean-filled | Lo | Mixed Dishes - Mexican |
| 58105110 | Pupusa, meat-filled | Lo | Mixed Dishes - Mexican |
| 58106200 | Pizza, cheese, from frozen, thin cru | Lo | Mixed Dishes - Pizza |
| 58106205 | Pizza, cheese, from frozen, thick cr | Lo | Mixed Dishes - Pizza |
| 58106210 | Pizza, cheese, from restaurant or fa | Lo | Mixed Dishes - Pizza |
| 58106220 | Pizza, cheese, from restaurant or fa | Lo | Mixed Dishes - Pizza |
| 58106225 | Pizza, cheese, from restaurant or fa | Lo | Mixed Dishes - Pizza |
| 58106230 | Pizza, cheese, from restaurant or fa | Lo | Mixed Dishes - Pizza |
| 58106233 | Pizza, cheese, stuffed crust | Lo | Mixed Dishes - Pizza |
| 58106234 | Pizza, cheese, from school lunch, m | Lo | Mixed Dishes - Pizza |
| 58106235 | Pizza, cheese, from school lunch, t | Lo | Mixed Dishes - Pizza |
| 58106236 | Pizza, cheese, from school lunch, t | Lo | Mixed Dishes - Pizza |
| 58106240 | Pizza, extra cheese, NS as to type | Lo | Mixed Dishes - Pizza |
| 58106250 | Pizza, extra cheese, thin crust | Lo | Mixed Dishes - Pizza |
| 58106255 | Pizza, extra cheese, regular crust | Lo | Mixed Dishes - Pizza |
| 58106260 | Pizza, extra cheese, thick crust | Lo | Mixed Dishes - Pizza |
| 58106300 | Pizza, cheese, with vegetables, fro | Lo | Mixed Dishes - Pizza |

| 58106305 | Pizza, cheese with vegetables, from | Lo | Mixed Dishes - Pizza |
| --- | --- | --- | --- |
| 58106310 | Pizza, cheese, with vegetables, NS | Lo | Mixed Dishes - Pizza |
| 58106320 | Pizza, cheese, with vegetables, fro | Lo | Mixed Dishes - Pizza |
| 58106325 | Pizza, cheese, with vegetables, fro | Lo | Mixed Dishes - Pizza |
[truncated: 295,563 more chars]
